# Supplementary material for: Impact of brining and drying processes on the nutritive value of tambaqui fish (Colossoma macropomum)
Source: PLoS One. 2024 Apr 16;19(4):e0299926. doi: 10.1371/journal.pone.0299926 (PMC11020844; doi:10.1371/journal.pone.0299926)
Supplement: S1 Table — (DOCX) [file pone.0299926.s001.docx]

**S1 Table.** Effect of individual drying temperatures on nutrient profiles of tambaqui fish.

| **Nutrients** | **Temperature (^o^C)** | **N** | **Mean** | **SD** | **SEM** | **95% CI for Mean** | | **Min.** | **Max.** |
| --- | --- | --- | --- | --- | --- | --- | --- | --- | --- |
|  |  |  |  |  |  | Lower Bound | Upper Bound |  |  |
| Fe | 30 | 3 | 36.13 | 2.43 | 1.44 | 30.08 | 42.16 | 34.19 | 38.85 |
|  | 35 | 3 | 41.24 | 0.68 | 0.39 | 39.55 | 42.94 | 40.46 | 41.70 |
|  | 40 | 3 | 36.75 | 0.63 | 0.36 | 35.19 | 38.31 | 36.04 | 37.21 |
|  | 45 | 3 | 38.66 | 0.70 | 0.40 | 36.92 | 40.39 | 37.97 | 39.37 |
|  | 50 | 3 | 40.34 | 2.18 | 1.26 | 34.93 | 45.75 | 38.35 | 42.67 |
|  | 55 | 3 | 54.92 | 1.33 | 0.77 | 51.62 | 58.23 | 53.99 | 56.45 |
|  | 60 | 3 | 38.39 | 1.22 | 0.71 | 35.35 | 41.43 | 37.01 | 39.33 |
|  | Total | 21 | 40.91 | 6.23 | 1.36 | 38.08 | 43.75 | 34.19 | 56.45 |
| K | 30 | 3 | 9.16 | 0.14 | 0.08 | 8.80 | 9.51 | 9.07 | 9.32 |
|  | 35 | 3 | 9.82 | 0.15 | 0.09 | 9.45 | 10.20 | 9.70 | 9.99 |
|  | 40 | 3 | 9.90 | 0.16 | 0.09 | 9.51 | 10.29 | 9.72 | 10.00 |
|  | 45 | 3 | 9.49 | 0.34 | 0.20 | 8.64 | 10.33 | 9.13 | 9.80 |
|  | 50 | 3 | 10.92 | 0.47 | 0.27 | 9.75 | 12.09 | 10.43 | 11.37 |
|  | 55 | 3 | 9.27 | 0.31 | 0.18 | 8.50 | 10.05 | 9.07 | 9.63 |
|  | 60 | 3 | 9.60 | 0.34 | 0.20 | 8.74 | 10.45 | 9.22 | 9.89 |
|  | Total | 21 | 9.74 | 0.61 | 0.13 | 9.46 | 10.02 | 9.07 | 11.37 |
| Mg | 30 | 3 | 1.34 | 0.03 | 0.02 | 1.27 | 1.40 | 1.32 | 1.37 |
|  | 35 | 3 | 1.28 | 0.04 | 0.02 | 1.18 | 1.37 | 1.23 | 1.30 |
|  | 40 | 3 | 1.29 | 0.04 | 0.03 | 1.18 | 1.41 | 1.25 | 1.34 |
|  | 45 | 3 | 1.16 | 0.06 | 0.03 | 1.01 | 1.30 | 1.10 | 1.22 |
|  | 50 | 3 | 1.37 | 0.03 | 0.02 | 1.29 | 1.46 | 1.33 | 1.40 |
|  | 55 | 3 | 1.08 | 0.06 | 0.03 | 0.94 | 1.21 | 1.01 | 1.11 |
|  | 60 | 3 | 1.19 | 0.09 | 0.05 | 0.97 | 1.42 | 1.10 | 1.28 |
|  | Total | 21 | 1.24 | 0.11 | 0.02 | 1.19 | 1.29 | 1.01 | 1.40 |
| Mn | 30 | 3 | 15.65 | 0.87 | 0.50 | 13.48 | 17.82 | 14.66 | 16.30 |
|  | 35 | 3 | 10.78 | 0.38 | 0.22 | 9.82 | 11.73 | 10.49 | 11.21 |
|  | 40 | 3 | 10.90 | 0.67 | 0.39 | 9.23 | 12.57 | 10.47 | 11.67 |
|  | 45 | 3 | 8.44 | 0.07 | 0.04 | 8.27 | 8.62 | 8.37 | 8.50 |
|  | 50 | 3 | 10.71 | 0.49 | 0.29 | 9.48 | 11.93 | 10.22 | 11.21 |
|  | 55 | 3 | 7.93 | 0.30 | 0.17 | 7.19 | 8.66 | 7.64 | 8.23 |
|  | 60 | 3 | 10.60 | 0.38 | 0.22 | 9.65 | 11.55 | 10.26 | 11.01 |
|  | Total | 21 | 10.71 | 2.41 | 0.53 | 9.62 | 11.81 | 7.64 | 16.30 |
| Na | 30 | 3 | 2.69 | 0.13 | 0.08 | 2.36 | 3.01 | 2.54 | 2.79 |
|  | 35 | 3 | 2.69 | 0.09 | 0.05 | 2.47 | 2.91 | 2.60 | 2.78 |
|  | 40 | 3 | 2.65 | 0.09 | 0.05 | 2.44 | 2.87 | 2.55 | 2.72 |
|  | 45 | 3 | 2.52 | 0.10 | 0.06 | 2.28 | 2.76 | 2.41 | 2.60 |
|  | 50 | 3 | 2.94 | 0.07 | 0.04 | 2.76 | 3.12 | 2.89 | 3.02 |
|  | 55 | 3 | 2.43 | 0.07 | 0.04 | 2.25 | 2.60 | 2.36 | 2.50 |
|  | 60 | 3 | 2.86 | 0.15 | 0.09 | 2.49 | 3.22 | 2.70 | 3.00 |
|  | Total | 21 | 2.68 | 0.19 | 0.04 | 2.60 | 2.77 | 2.36 | 3.02 |
| P | 30 | 3 | 21.73 | 0.95 | 0.55 | 19.38 | 24.08 | 20.89 | 22.75 |
|  | 35 | 3 | 21.19 | 1.05 | 0.61 | 18.57 | 23.80 | 20.02 | 22.05 |
|  | 40 | 3 | 22.28 | 0.86 | 0.50 | 20.15 | 24.42 | 21.34 | 23.02 |
|  | 45 | 3 | 19.75 | 0.44 | 0.25 | 18.67 | 20.84 | 19.25 | 20.03 |
|  | 50 | 3 | 25.26 | 0.77 | 0.45 | 23.34 | 27.18 | 24.60 | 26.11 |
|  | 55 | 3 | 17.44 | 0.40 | 0.23 | 16.45 | 18.43 | 16.99 | 17.76 |
|  | 60 | 3 | 23.39 | 0.93 | 0.54 | 21.07 | 25.70 | 22.34 | 24.12 |
|  | Total | 21 | 21.58 | 2.48 | 0.54 | 20.45 | 22.71 | 16.99 | 26.11 |
| Zn | 30 | 3 | 54.89 | 0.77 | 0.45 | 52.96 | 56.81 | 54.05 | 55.59 |
|  | 35 | 3 | 55.26 | 1.05 | 0.61 | 52.64 | 57.88 | 54.23 | 56.33 |
|  | 40 | 3 | 48.85 | 1.32 | 0.76 | 45.59 | 52.12 | 47.97 | 50.37 |
|  | 45 | 3 | 38.23 | 0.33 | 0.19 | 37.42 | 39.05 | 38.00 | 38.61 |
|  | 50 | 3 | 50.64 | 0.68 | 0.39 | 48.96 | 52.32 | 49.90 | 51.22 |
|  | 55 | 3 | 44.02 | 1.23 | 0.71 | 40.96 | 47.08 | 42.64 | 45.01 |
|  | 60 | 3 | 46.28 | 1.26 | 0.72 | 43.17 | 49.40 | 45.00 | 47.51 |
|  | Total | 21 | 48.31 | 5.82 | 1.27 | 45.66 | 50.96 | 38.00 | 56.33 |
| Se | 30 | 3 | 153.51 | 2.17 | 1.25 | 148.12 | 158.90 | 151.32 | 155.66 |
|  | 35 | 3 | 166.09 | 0.94 | 0.54 | 163.75 | 168.43 | 165.14 | 167.02 |
|  | 40 | 3 | 152.25 | 0.81 | 0.47 | 150.23 | 154.27 | 151.39 | 153.00 |
|  | 45 | 3 | 121.31 | 0.66 | 0.38 | 119.67 | 122.96 | 120.86 | 122.07 |
|  | 50 | 3 | 165.49 | 1.39 | 0.80 | 162.04 | 168.94 | 164.07 | 166.84 |
|  | 55 | 3 | 130.09 | 1.10 | 0.63 | 127.37 | 132.82 | 129.04 | 131.23 |
|  | 60 | 3 | 156.07 | 2.08 | 1.20 | 150.90 | 161.24 | 154.30 | 158.37 |
|  | Total | 21 | 149.26 | 16.33 | 3.56 | 141.83 | 156.69 | 120.86 | 167.02 |
| I | 30 | 3 | 674.44 | 1.09 | 0.63 | 671.74 | 677.15 | 673.19 | 675.13 |
|  | 35 | 3 | 526.88 | 1.05 | 0.60 | 524.27 | 529.48 | 526.00 | 528.04 |
|  | 40 | 3 | 671.40 | 1.07 | 0.62 | 668.73 | 674.07 | 670.23 | 672.34 |
|  | 45 | 3 | 464.08 | 0.96 | 0.55 | 461.69 | 466.46 | 463.10 | 465.02 |
|  | 50 | 3 | 557.52 | 1.69 | 0.97 | 553.33 | 561.71 | 556.01 | 559.34 |
|  | 55 | 3 | 614.43 | 0.59 | 0.34 | 612.95 | 615.90 | 614.01 | 615.11 |
|  | 60 | 3 | 717.30 | 1.26 | 0.73 | 714.17 | 720.42 | 716.00 | 718.52 |
|  | Total | 21 | 603.72 | 86.73 | 18.92 | 564.24 | 643.20 | 463.10 | 718.52 |
| % DM | 30 | 3 | 100.00 | 0.00 | 0.00 | 100.00 | 100.00 | 100.00 | 100.00 |
|  | 35 | 3 | 100.00 | 0.00 | 0.00 | 100.00 | 100.00 | 100.00 | 100.00 |
|  | 40 | 3 | 100.00 | 0.00 | 0.00 | 100.00 | 100.00 | 100.00 | 100.00 |
|  | 45 | 3 | 100.00 | 0.00 | 0.00 | 100.00 | 100.00 | 100.00 | 100.00 |
|  | 50 | 3 | 100.00 | 0.00 | 0.00 | 100.00 | 100.00 | 100.00 | 100.00 |
|  | 55 | 3 | 100.00 | 0.00 | 0.00 | 100.00 | 100.00 | 100.00 | 100.00 |
|  | 60 | 3 | 100.00 | 0.00 | 0.00 | 100.00 | 100.00 | 100.00 | 100.00 |
|  | Total | 21 | 100.00 | 0.00 | 0.00 | 100.00 | 100.00 | 100.00 | 100.00 |
| %Ash | 30 | 3 | 11.87 | 0.79 | 0.45 | 9.92 | 13.83 | 11.03 | 12.58 |
|  | 35 | 3 | 12.38 | 0.36 | 0.21 | 11.49 | 13.27 | 12.07 | 12.77 |
|  | 40 | 3 | 12.12 | 0.16 | 0.09 | 11.73 | 12.51 | 11.99 | 12.30 |
|  | 45 | 3 | 12.04 | 0.17 | 0.10 | 11.63 | 12.46 | 11.90 | 12.23 |
|  | 50 | 3 | 13.07 | 0.11 | 0.06 | 12.80 | 13.35 | 12.99 | 13.20 |
|  | 55 | 3 | 11.74 | 0.25 | 0.15 | 11.11 | 12.37 | 11.51 | 12.01 |
|  | 60 | 3 | 13.03 | 0.06 | 0.04 | 12.87 | 13.19 | 12.97 | 13.10 |
|  | Total | 21 | 12.32 | 0.59 | 0.13 | 12.05 | 12.59 | 11.03 | 13.20 |
| % Protein | 30 | 3 | 60.75 | 0.37 | 0.21 | 59.82 | 61.67 | 60.33 | 61.03 |
|  | 35 | 3 | 59.48 | 0.76 | 0.44 | 57.59 | 61.37 | 58.78 | 60.29 |
|  | 40 | 3 | 59.54 | 1.60 | 0.92 | 55.56 | 63.51 | 57.75 | 60.85 |
|  | 45 | 3 | 59.61 | 0.68 | 0.39 | 57.92 | 61.29 | 58.89 | 60.24 |
|  | 50 | 3 | 56.99 | 0.65 | 0.37 | 55.39 | 58.60 | 56.35 | 57.64 |
|  | 55 | 3 | 59.53 | 0.51 | 0.29 | 58.27 | 60.79 | 59.00 | 60.02 |
|  | 60 | 3 | 59.34 | 1.85 | 1.07 | 54.74 | 63.93 | 57.67 | 61.33 |
|  | Total | 21 | 59.32 | 1.39 | 0.30 | 58.69 | 59.95 | 56.35 | 61.33 |
| %Fat | 30 | 3 | 27.12 | 1.04 | 0.60 | 24.55 | 29.70 | 26.15 | 28.21 |
|  | 35 | 3 | 28.08 | 0.20 | 0.12 | 27.58 | 28.59 | 27.93 | 28.31 |
|  | 40 | 3 | 29.15 | 0.28 | 0.16 | 28.44 | 29.85 | 28.97 | 29.48 |
|  | 45 | 3 | 28.33 | 0.61 | 0.35 | 26.82 | 29.84 | 27.75 | 28.97 |
|  | 50 | 3 | 30.05 | 0.35 | 0.20 | 29.17 | 30.93 | 29.67 | 30.37 |
|  | 55 | 3 | 28.53 | 0.50 | 0.29 | 27.29 | 29.77 | 27.99 | 28.98 |
|  | 60 | 3 | 28.51 | 0.79 | 0.46 | 26.54 | 30.48 | 27.65 | 29.21 |
|  | Total | 21 | 28.54 | 1.00 | 0.22 | 28.09 | 28.99 | 26.15 | 30.37 |
| Ca | 30 | 3 | 37.75 | 1.04 | 0.60 | 35.17 | 40.33 | 36.60 | 38.62 |
|  | 35 | 3 | 36.76 | 0.37 | 0.21 | 35.85 | 37.67 | 36.38 | 37.12 |
|  | 40 | 3 | 38.19 | 0.65 | 0.37 | 36.58 | 39.80 | 37.66 | 38.91 |
|  | 45 | 3 | 33.66 | 1.41 | 0.81 | 30.16 | 37.16 | 32.30 | 35.11 |
|  | 50 | 3 | 41.85 | 1.65 | 0.96 | 37.74 | 45.96 | 40.12 | 43.42 |
|  | 55 | 3 | 32.31 | 1.45 | 0.84 | 28.71 | 35.91 | 30.66 | 33.34 |
|  | 60 | 3 | 41.48 | 1.01 | 0.58 | 38.96 | 44.00 | 40.52 | 42.54 |
|  | Total | 21 | 37.43 | 3.54 | 0.77 | 35.81 | 39.04 | 30.66 | 43.42 |

*No significant effect (*P > 0.05*) of temperatures on individual nutrient profiles.
