# Supplementary material for: Impact of brining and drying processes on the nutritive value of tambaqui fish (Colossoma macropomum)
Source: PLoS One. 2024 Apr 16;19(4):e0299926. doi: 10.1371/journal.pone.0299926 (PMC11020844; doi:10.1371/journal.pone.0299926)
Supplement: Attachment — Submitted filename: Summary of Comments on PONE-D-22-35077_EA.pdf [file pone.0299926.s007.pdf]

Summary of Comments on PONE-D-22-35077_EA.pdf

Page: 7

Author: USER   Subject: Sticky Note   Date: 8/28/2023 4:49:08 PM

There is no simulation in this study, please change the title to Impact of brining and drying processes...................

Author: USER   Subject: Sticky Note   Date: 8/28/2023 4:52:20 PM

You have mentioned that electricity is a problem to rural communities of most tropical regions, using electric oven for your experiment should not be an accessible preservative strategies.

Author: USER Subject: Highlight Date: 8/28/2023 4:58:13 PM

Author: USER Subject: Sticky Note Date: 8/28/2023 4:55:49 PM

Please add the effect brining on the nutrient profile and at what significant level?

Author: USER Subject: Sticky Note Date: 8/28/2023 5:49:14 PM

I will suggest that you re-write your results section of the abstract: Present the ANOVA results on all the nutrient profile and the interactive effect of brining and drying processes(if it was done).
Follow by the mean separation results for the key nutrient that had significant effect.
PCA result

Author: USER Subject: Sticky Note Date: 8/28/2023 5:03:23 PM

Please add the a sentence on the practical application of your findings.

Author: USER Subject: Sticky Note Date: 8/28/2023 5:13:18 PM

Your introduction is not flowing well.
Following the general introduction on fis. please add the following:
- Explain the importance of studying the nutrient profiles in fish.
- Provide information on the natural habitat and characteristics of the studied fish
- Highlight the nutritional importance of this fish species for human consumption
-Then, Discuss the drying process and its role in enhancing the shelf life of fish
- A brief literature review on various traditional fish preservatives eg, sundrying, smoking, brinning,etc,
Then , Brining as yiu have in Line2s 42 to 60,
Drying as you have it in Lines 61 to 81.

Page: 10

Author: USER Subject: Highlight Date: 8/28/2023 5:14:21 PM

Author: USER Subject: Sticky Note Date: 8/28/2023 5:16:56 PM

This is not true,

There are several studies on brining and drying of fish. What makes your study unique is species you are using.

Please check the following papers as examples:

Kituu, G. M., Shitanda, D., Silayo, V., Odote, P., & Bongyereire, J. (2008). Effect of brining on the drying rate of tilapia (Oreochromis niloticus) in a solar tunnel dryer.

Abraha, B., Admassu, H., Mahmud, A., Tsighe, N., Shui, X. W., & Fang, Y. (2018). Effect of processing methods on nutritional and physico-chemical composition of fish: a review. MOJ Food Process Technol, 6(4), 376-82.

Guizani, N., Rahman, M. S., Al-Ruzeiqi, M. H., Al-Sabahi, J. N., & Sureshchandran, S. (2014). Effects of brine concentration on lipid oxidation and fatty acids profile of hot smoked tuna (Thunnus albacares) stored at refrigerated temperature. Journal of Food Science and Technology, 51, 577-582.

Sampels, S. (2015). The effects of processing technologies and preparation on the final quality of fish products. Trends in Food Science & Technology, 44(2), 131-146.

Doe, P., & Olley, J. (2020). Drying and dried fish products. In Seafood (pp. 125-145). CRC Press.

Author: USER Subject: Highlight Date: 8/28/2023 5:17:54 PM

Author: USER Subject: Sticky Note Date: 8/28/2023 5:22:16 PM

Please you are simulating you are just checking the impact of brining and drying processes. Also, the rural conditions was not simulated using electric oven in the lab.
Rural areas, they used sun drying. If you used sun drying or smoking processes, you will surely get a different result.
I would have suggested you used sun drying, smoking as control.

Author: USER Subject: Sticky Note Date: 8/28/2023 5:23:00 PM

Imported from where?

Author: USER Subject: Highlight Date: 8/28/2023 5:24:34 PM

Author: USER Subject: Highlight Date: 8/28/2023 5:24:16 PM

Author: USER Subject: Sticky Note Date: 8/28/2023 5:24:03 PM

Using which method? Please add more information on the rearing process.

Author: USER Subject: Highlight Date: 8/28/2023 5:24:48 PM

Author: USER Subject: Sticky Note Date: 8/28/2023 5:25:01 PM

Please add reference

Author: USER Subject: Sticky Note Date: 8/28/2023 5:25:53 PM

Author: USER Subject: Sticky Note Date: 8/28/2023 5:26:59 PM

What type of anesthetic agent?, give the brand,manufacturer, country

Author: USER Subject: Highlight Date: 8/28/2023 5:25:58 PM

Page: 12

Author: USER Subject: Highlight Date: 8/28/2023 5:28:27 PM

Author: USER Subject: Sticky Note Date: 8/28/2023 5:28:59 PM
This is repetition. You have given the detail earlier

Author: USER Subject: Sticky Note Date: 8/28/2023 5:32:19 PM
Was it stainless?

Author: USER Subject: Highlight Date: 8/28/2023 5:31:45 PM

Author: USER Subject: Sticky Note Date: 8/28/2023 5:30:32 PM

Author: USER Subject: Sticky Note Date: 8/28/2023 5:31:35 PM
Using tap water? An you carried out Fe and Zn analysis on the samples. How did you avoid contamination?

Author: USER Subject: Highlight Date: 8/28/2023 5:30:30 PM

Author: USER Subject: Sticky Note Date: 8/28/2023 5:36:08 PM
Why 15%?

Author: USER Subject: Sticky Note Date: 8/28/2023 5:35:24 PM
Please add reference

Author: USER Subject: Sticky Note Date: 8/28/2023 5:34:12 PM

Author: USER Subject: Sticky Note Date: 8/28/2023 5:34:01 PM
How did select these temperatures, especially the interval of 5? Any reference?

Author: USER Subject: Highlight Date: 8/28/2023 5:32:43 PM

Author: USER Subject: Sticky Note Date: 8/28/2023 5:36:45 PM
Add reference for this process

Author: amir4 Subject: Highlight Date: 8/24/2023 7:50:11 AM
Please specify the model of oven used and Humidity condition set for laboratory simulation.

Author: amir4 Subject: Note Date: 8/23/2023 8:25:54 AM
Please state the humidity conditions while simulating natural drying environment and specify the model of oven used??

Author: amir4 Subject: Highlight Date: 8/23/2023 8:45:22 AM
This statement looks contradictory with the title "A laboratory simulation of the impact of brining and drying as accessible preservation strategies under tropical rural conditions on the nutritive value of tambaqui fish (Colossoma macropomum)"

Author: USER Subject: Sticky Note Date: 8/28/2023 5:47:15 PM
Please give the particle size or the sieve size used

Author: USER Subject: Highlight Date: 8/28/2023 5:46:54 PM

Author: USER Subject: Sticky Note Date: 8/28/2023 5:38:38 PM
No reference for the Brining and drying processes

Author: USER Subject: Sticky Note Date: 8/28/2023 5:39:50 PM
Please give full meaning of all the acronyms at first mentioned

Author: USER Subject: Highlight Date: 8/28/2023 5:39:12 PM

Page: 14

Author: USER Subject: Sticky Note Date: 8/28/2023 5:44:58 PM

Please add quality control steps observed to avoid contaminations.
Also, please give the Limit of detection, sensitivity, accuracy level etc of the ICP-OES used.
Please give simple steps of estimating the values of the minerals using ICP-OES.

Page: 15

Author: USER Subject: Highlight Date: 8/28/2023 5:45:41 PM

Author: USER Subject: Sticky Note Date: 8/28/2023 5:46:14 PM
How many sampling point?

Author: USER Subject: Sticky Note Date: 8/28/2023 5:48:03 PM
What sieve size?

Author: USER Subject: Highlight Date: 8/28/2023 5:47:47 PM

Author: USER Subject: Sticky Note Date: 8/28/2023 5:51:30 PM

Please present all tables in an acceptable format. Check Journal guidelines

Page: 23

Author: amir4 Subject: Note Date: 8/24/2023 7:42:49 AM

The language of the conclusion must be improved for better understanding of the reader. This section must be sound.

Page: 24

Author: USER Subject: Sticky Note Date: 8/28/2023 5:53:16 PM

Please add the practical application limitation of this study, recommendations to the conclusion.
