# Supplementary material for: Impact of brining and drying processes on the nutritive value of tambaqui fish (Colossoma macropomum)
Source: PLoS One. 2024 Apr 16;19(4):e0299926. doi: 10.1371/journal.pone.0299926 (PMC11020844; doi:10.1371/journal.pone.0299926)

PLOS ONE

A laboratory simulation of the impact of brining and drying as accessible preservation strategies under tropical rural conditions on the nutritive value of tambaqui fish (Colossoma macropomum)

--Manuscript Draft--

**Manuscript Number:**	PONE-D-22-35077
**Article Type:**	Research Article
**Full Title:**	A laboratory simulation of the impact of brining and drying as accessible preservation strategies under tropical rural conditions on the nutritive value of tambaqui fish (Colossoma macropomum)
**Short Title:**	Effect of brining and drying on nutritive value of tambaqui fish.
**Corresponding Author:**	Awot Teklu MEBRATU<br>Universiteit Gent<br>Merelbeke, BELGIUM
**Keywords:**	brining; drying; nutrient profiles; tambaqui; reactive lysine
**Abstract:**	Preservation of fish as diet ingredient is challenging in many tropical conditions due to the absence of freezing facilities, hence warranting other preservation techniques. The present study evaluated the effect of brine salting (15% w/w) prior to drying at different temperatures on the nutrient profiles of tambaqui fish (Colossoma macropomum). Whole fish samples (792 ± 16 g; 8 months old; n = 48) were grouped into two as brine-salted and non-salted, and treated at seven different drying temperatures of 30, 35, 40, 45, 50, 55 and 60°C for a period of 23 h each. To evaluate the impact of Maillard reaction, reactive lysine was also quantified. Drying temperature had no effect on the nutrient profiles of tambaqui fish (P > 0.05). Brining reduced the overall protein concentration by 6% (P < 0.05). Although brining significantly diminished many amino acids (taurine 56%, methionine 17%, cysteine 11%, reactive lysine 11% and histidine 10%), proline and glycine increased with 5 and 6%, respectively, whereas alanine and arginine were not affected. Brining also reduced the concentrations of Se (14%), I (38%), K (42%) and Mg (18%) (P < 0.05) versus an anticipated vast increase in Na (744%) and ash (28%) concentrations (P < 0.05). There was no significant effect of brining on Fe, Zn, Mn, P and Ca concentrations and brining and drying on body fat content of tambaqui fish (P > 0.05). From a nutritional viewpoint, drying without brining is the best approach to maintain the nutritive value of tambaqui fish; neither brining nor drying temperature induced changes in lysine reactivity.
**Order of Authors:**	Awot Teklu MEBRATU
	Yohannes Tekle Asfaw
	Wouter Merckx
	Wouter Hendriks
	Geert Janssens
**Additional Information:**	
**Question**	**Response**
**Financial Disclosure**<br><br>Enter a financial disclosure statement that describes the sources of funding for the work included in this submission. Review the submission guidelines for detailed requirements. View published research articles from *PLOS ONE* for specific examples.	The project was financially supported by the VLIR-UOS TEAM project under the title 'Win-win protein: reconciling fish protein generation and water quality management' [grant number ET2018TEA462A101] through Geert P.J Janssens and Luc De Meester; https://www.vliruos.be/en/home/1. The funders had no role in study design, data collection and analysis, decision to publish, or preparation of the manuscript. ATM received support in form of salary while doing the research activities.

Powered by Editorial Manager® and ProduXion Manager® from Aries Systems Corporation

This statement is required for submission and **will appear in the published article** if the submission is accepted. Please make sure it is accurate.<br><br>**Unfunded studies**<br>Enter: *The author(s) received no specific funding for this work.*<br><br>**Funded studies**<br>Enter a statement with the following details:<br>• Initials of the authors who received each award<br>• Grant numbers awarded to each author<br>• The full name of each funder<br>• URL of each funder website<br>• Did the sponsors or funders play any role in the study design, data collection and analysis, decision to publish, or preparation of the manuscript?<br>• **NO** - Include this sentence at the end of your statement: *The funders had no role in study design, data collection and analysis, decision to publish, or preparation of the manuscript.*<br>• **YES** - Specify the role(s) played.<br><br>* typeset	
**Competing Interests**<br><br>Use the instructions below to enter a competing interest statement for this submission. On behalf of all authors, disclose any competing interests that could be perceived to bias this work—acknowledging all financial support and any other relevant financial or non-financial competing interests.<br><br>This statement is required for submission and **will appear in the published article** if the submission is accepted. Please make sure it is accurate and that any funding sources listed in your Funding Information later in the submission form are also declared in your Financial Disclosure statement.<br><br>View published research articles from	The authors have declared that no competing interests exist.

[*PLOS ONE*]() for specific examples.<br><br>**NO authors have competing interests**<br><br>Enter: *The authors have declared that no competing interests exist.*<br><br>**Authors with competing interests**<br><br>Enter competing interest details beginning with this statement:<br><br>*I have read the journal's policy and the authors of this manuscript have the following competing interests: [insert competing interests here]*<br><br>* typeset	
**Ethics Statement**<br><br>Enter an ethics statement for this submission. This statement is required if the study involved:<br><br>• Human participants<br>• Human specimens or tissue<br>• Vertebrate animals or cephalopods<br>• Vertebrate embryos or tissues<br>• Field research<br><br>Write "N/A" if the submission does not require an ethics statement.<br><br>General guidance is provided below. Consult the submission guidelines for detailed instructions. **Make sure that all information entered here is included in the Methods section of the manuscript.**	All experimental and animal care procedures were realized based upon the current written knowledge of aquaculture technologies and tambaquí fish farming. Fingerlings of Colossoma macropomum were initially imported to TRANSfarm and reared for about 7 months period before being subject to the current study. During their stay, fish were kept in larger aqua-tanks at a density of 1.2 fish/m3 sufficient enough for movements and behavioral expression. Tanks were fixed with individual bio-filters, well aerated with a constant temperature supply, 18:6 h light - dark cycle, and supplying pipes were regularly inspected and cleaned with tap water and water quality parameters were checked twice a week and water renewed accordingly.<br>For our experimental study, after a 12 h fasting, a total of 48 tambaqui fish were randomly selected and kept in a separate tank and were euthanized in a two-step procedure (Lewbart, 2019). Six fish were randomly taken at a time and placed in a tank with a volume of 240 L water into which 50 mL of phenoxyethanol-solution (Anest Fish©) was added. After five minutes, 30 L of water were elevated out of the tanks and put into a large bucket. Another 6 mL of Anest Fish© was added to this bucket. Each group of six unconscious fish was scooped out of the tank and brought to the slaughter room in the bucket with the anesthetic agent. Thereafter, tambaqui were killed with a firm blow to the head, followed by fracturing the brainstem with a knife and then dissected for the experimental use (Lewbart, 2019). The study was conducted in accordance with the Declaration of Helsinki and directive 2010/63/EU of the European parliament and of the council on the protection of animals used for scientific purposes, and the wellbeing of the cultured tambaquí fish before being subjected to the experiment was assessed and approved by the Institutional Review Board of TRANSfarm, KU Leuven, Belgium (TRANSfarm17.09/2019).

Powered by Editorial Manager® and ProduXion Manager® from Aries Systems Corporation

**Format for specific study types**<br><br>**Human Subject Research (involving human participants and/or tissue)**<br>• Give the name of the institutional review board or ethics committee that approved the study<br>• Include the approval number and/or a statement indicating approval of this research<br>• Indicate the form of consent obtained (written/oral) or the reason that consent was not obtained (e.g. the data were analyzed anonymously)<br><br>**Animal Research (involving vertebrate animals, embryos or tissues)**<br>• Provide the name of the Institutional Animal Care and Use Committee (IACUC) or other relevant ethics board that reviewed the study protocol, and indicate whether they approved this research or granted a formal waiver of ethical approval<br>• Include an approval number if one was obtained<br>• If the study involved *non-human primates*, add *additional details* about animal welfare and steps taken to ameliorate suffering<br>• If anesthesia, euthanasia, or any kind of animal sacrifice is part of the study, include briefly which substances and/or methods were applied<br><br>**Field Research**<br><br>Include the following details if this study involves the collection of plant, animal, or other materials from a natural setting:<br>• Field permit number<br>• Name of the institution or relevant body that granted permission	
**Data Availability**<br><br>Authors are required to make all data underlying the findings described fully available, without restriction, and from the time of publication. PLOS allows rare exceptions to address legal and ethical concerns. See the PLOS Data Policy and FAQ for detailed information.	Yes - all data are fully available without restriction

Powered by Editorial Manager® and ProduXion Manager® from Aries Systems Corporation

A Data Availability Statement describing where the data can be found is required at submission. Your answers to this question constitute the Data Availability Statement and **will be published in the article**, if accepted.<br><br>**Important:** Stating 'data available on request from the author' is not sufficient. If your data are only available upon request, select 'No' for the first question and explain your exceptional situation in the text box.<br><br>Do the authors confirm that all data underlying the findings described in their manuscript are fully available without restriction?	
**Describe where the data may be found in full sentences. If you are copying our sample text, replace any instances of XXX with the appropriate details.**<br><br>• If the data are **held or will be held in a public repository**, include URLs, accession numbers or DOIs. If this information will only be available after acceptance, indicate this by ticking the box below. For example: *All XXX files are available from the XXX database (accession number(s) XXX, XXX.)*.<br>• If the data are all contained **within the manuscript and/or Supporting Information files**, enter the following: *All relevant data are within the manuscript and its Supporting Information files.*<br>• If neither of these applies but you are able to provide **details of access elsewhere**, with or without limitations, please do so. For example:<br><br>*Data cannot be shared publicly because of [XXX]. Data are available from the XXX Institutional Data Access / Ethics Committee (contact via XXX) for researchers who meet the criteria for access to confidential data.*<br><br>*The data underlying the results presented in the study are available from (include the name of the third party*	The data underlying the results presented in the study are available from the corresponding author (ATM, awotteklu.mebratu.ugent.be) and will be shared or uploaded without restriction.

and contact information or URL).<br>• This text is appropriate if the data are owned by a third party and authors do not have permission to share the data.<br><br>* typeset	
Additional data availability information:	

Manuscript file

Click here to access/download;Manuscript;Manuscript file_Awot et al.docx

A laboratory simulation of the impact of brining and drying as accessible preservation strategies under tropical rural conditions on the nutritive value of tambaqui fish (*Colossoma macropomum*)

Awot Teklu Mebratu[1,2*], Yohannes Tekle Asfaw[3], Wouter Merckx[4], Wouter H. Hendriks[5], Geert P.J. Janssens[1]

[1]Department of Veterinary and Biosciences, Faculty of Veterinary Medicine, Ghent University, Merelbeke, Belgium

[2]Department of Animal Reproduction and Welfare, College of Veterinary Sciences, Mekelle University, Mekelle, Ethiopia

[3]Department of Veterinary Basic and Diagnostic Sciences, College of Veterinary Sciences, Mekelle University, Mekelle, Ethiopia

[4]TRANSfarm, Engineering and technology group, The Catholic University of Leuven, Lovenjoel, Belgium

[5]Animal Nutrition Group, Wageningen University, Wageningen, The Netherlands

[*]Corresponding author

Email: awotteklu.mebratu@ugent.be (ATM)

Abstract

Preservation of fish as diet ingredient is challenging in many tropical conditions due to the absence of freezing facilities, hence warranting other preservation techniques. The present study evaluated the effect of brine salting (15% *w/w*) prior to drying at different temperatures on the nutrient profiles of tambaqui fish (*Colossoma macropomum*). Whole fish samples (792 ± 16 g; 8 months old; n = 48) were grouped into two as brine-salted and non-salted, and treated at seven different drying temperatures of 30, 35, 40, 45, 50, 55 and 60°C for a period of 23 h each. To evaluate the impact of Maillard reaction, reactive lysine was also quantified. Drying temperature had no effect on the nutrient profiles of tambaqui fish ($P > 0.05$). Brining reduced the overall protein concentration by 6% ($P < 0.05$). Although brining significantly diminished many amino acids (taurine 56%, methionine 17%, cysteine 11%, reactive lysine 11% and histidine 10%), proline and glycine increased with 5 and 6%, respectively, whereas alanine and arginine were not affected. Brining also reduced the concentrations of Se (14%), I (38%), K (42%) and Mg (18%) ($P < 0.05$) versus an anticipated vast increase in Na (744%) and ash (28%) concentrations ($P < 0.05$). There was no significant effect of brining on Fe, Zn, Mn, P and Ca concentrations and brining and drying on body fat content of tambaqui fish ($P > 0.05$). From a nutritional viewpoint, drying without brining is the best approach to maintain the nutritive value of tambaqui fish; neither brining nor drying temperature induced changes in lysine reactivity.

Keywords: Brining; drying; nutrient profiles; tambaqui; reactive lysine.

Introduction

Fish meat is a high-value, perishable protein-rich food requiring appropriate preservation to maintain its organoleptic properties after harvest [1,2]. Freezing is an efficient manner for fish preservation, but in many rural areas of the Global South, freezing fish is not an option

because of poor electricity supply and the unaffordable cost of freezers. Alternative methods for fish preservation are warranted in such conditions. Brine salting is one of the oldest traditional and economically viable preservation methods of edible animal proteins such as fish meat [3-5]. It involves the use of salt (NaCl) that, due to its hygroscopic property, withdraws water from tissues via osmosis and, thereby, reduces water activity to slow bacterial growth [3]. In addition, salt is also reported to improve product flavor, extract proteins, aid in emulsion formation, and reduce proteolytic activities of enzymes [6-8].

The quantity and concentration of salt and treatment time are determining factors in the process that could affect the nutritional quality of the fish [9-11]. A 15% brine concentration for a period of 48 h has been reported to yield optimum protein solubility/availability in a comparative study carried out in cod [5]. Likewise, better mass transfer kinetics of salt to tissues of cod and salmon were observed at 15% (*w/w*) brine concentration [4]. In fact, 10-15% (*w/w*) is the salt concentration recommended for salting whole fish under the food safety and standards regulation [12]. Higher concentrations of up to 25% (*w/w*) denature muscle proteins by causing cell membrane disruption, decreased enzymatic activity, or interaction with myofibrils, and reducing its water-holding capacity [4,5,13]. In general, higher yields of tissue proteins are obtained at lower salt concentrations [5,7,14]. In addition to the salt concentration, the purity of salt, salting method involved, the size, thickness and freshness of the fish, fish-to-salt ratio, and weather conditions are further factors of importance for the quality brine preserved fish [3,4].

Drying involves the exposure of a product to specified temperature and time period to facilitate loss of tissue water to a lower limit of water activity ($a_w < 0.90$) where microbial growth is inhibited [15]. Drying temperature, relative humidity and air velocity are determining the rate of dehydration [2]. In rural areas, temperature-controlled ovens are usually not available, and open-air drying is the only option. Especially high environmental temperatures

during drying may affect the nutritive value of fish, for instance because heat may damage amino acids or may increase the reaction of amino acids with lipids and/or carbohydrates to form biologically unavailable Maillard products [16]. To determine the risk of drying at uncontrolled temperature for the nutritive value of fish, it is warranted to study the effect of a wide enough temperature range for fish drying.

Depending on the type of drying oven and species and size of the fish involved, a temperature range of 65 - 135°C and drying time of 2 - 20 h have been routinely used for drying of fish meat [2,7]. However, due to possible conjoint loss of some non-moisture compounds (e.g. volatile fatty acids and vitamins), lower temperatures with extended drying time are often recommended [17]. For example, drying of salted shark at 35°C reported to yield improved protein solubility, tissue rehydration and water retention capacity and total protein estimates compared to 45, 55 and 60°C [18]. Pacheco [19] also stated that the maximum drying temperature for the majority of the edible fish meat should not exceed 70°C. In fact, drying of fish products at higher temperatures, of more than 60°C, reported to cause fat oxidation and caramelization of sugars [18]. Under tropical "rural" conditions without ovens, temperature will likely not exceed 60°C but may typically remain above 30°C [20].

Tambaqui (*Colossoma macropomum*, Cuvier, 1818) is a tropical freshwater fish most widely cultivated in the Amazon region [21]. The species' higher productivity and hardiness have all increased its global demand [22]. To date, there is no information about the effect of brining and drying temperatures on the nutrient profiles of this fish species. Our study also investigated reactive lysine as a marker for Maillard product formation: lysine is a limiting amino acid in most animal diets and need to be bioavailable to be used in metabolism. Despite its high concentration in tissue deposition, some reducing sugars, fats and their oxidation products and vitamins can bind the ε-amino group of lysine and generate Maillard reaction products during fish processing [16]. Hence, measuring the content of "reactive lysine" as a

percentage of total lysine in tambaqui fish can indicate state of protein damage during processing. The present study therefore simulated rural conditions of brining and a range of drying temperatures to identify how these factors influence macronutrient and mineral composition as well as Maillard reaction that may reduce lysine availability.

Materials and methods

Experimental animal ethics and welfare

All experimental and animal care procedures were realized based upon the current written knowledge of aquaculture technologies and tambaquí fish farming. Fingerlings of *Colossoma macropomum* were initially imported to TRANSfarm and reared for about 7 months period before being subject to the current study. During their stay, fish were kept in larger aqua-tanks at a density of 1.2 fish/m^3 sufficient enough for movements and behavioral expression. Tanks were fixed with individual bio-filters, well aerated with a constant temperature supply, 18:6 h light - dark cycle, and supplying pipes were regularly inspected and cleaned with tap water and water quality parameters were checked twice a week and water renewed accordingly.

For our experimental study, after a 12 h fasting, a total of 48 tambaqui fish were randomly selected and kept in a separate tank and were euthanized in a two-step procedure [23]. Six fish were randomly taken at a time and placed in a tank with a volume of 240 L water into which 50 mL of phenoxyethanol-solution (Anest Fish©) was added. After five minutes, 30 L of water were elevated out of the tanks and put into a large bucket. Another 6 mL of Anest Fish© was added to this bucket. Each group of six unconscious fish was scooped out of the tank and brought to the slaughter room in the bucket with the anesthetic agent. Thereafter, tambaqui were killed with a firm blow to the head, followed by fracturing the brainstem with a knife and then transported in ice cubes for the experiment [23]. The study was conducted in accordance with the Declaration of Helsinki and directive 2010/63/EU of the European parliament and of the council on the protection of animals used for scientific purposes, and the wellbeing of the

cultured tambaquí fish before being subjected to the experiment was assessed and approved by the Institutional Review Board of TRANSfarm, KU Leuven, Belgium (TRANSfarm17.09/2019).

Study materials

A total of 48 tambaqui fish (792 ± 16 g; 8 months old) were collected from TRANSfarm, Lovenjoel, Belgium, and transported to the animal nutrition laboratory of the Department of Veterinary and Biosciences, Ghent University in styrofoam boxes with ice packs (Luxaplast, NORDIC®). Fish were initially starved for 24 h and humanely euthanized in ice cubes before transport. Upon arrival, to facilitate drying whole fish were dissected dorso-ventrally into two equal slices using a post mortem saw ([illegible]-PM/110, 254MM) and scalpel-blades, thoroughly washed using tap water and then individually stored in plastic bags at -20°C for 48 h. All dissected fish slices had an average uniform dimension of 21.2 ± 2.8 cm long and 12.6 ± 2.0 cm wide and average weight of 389 ± 11.5 g. Industrial table salt (99.9%, BDH-PROLABO®, EC) was used for preparing the 15% (*w/w*) brine solution in the present study.

Experimental design and set-up

Uniformly sliced whole fish samples were grouped into two as brine-salted and non-salted, and treated at seven different drying temperatures of [illegible]35, 40, 45, 50, 55 and 60°C for a period of 23 h each. Each temperature treatment was considered as a sampling point and tested in triplicate. Similarly, as a reference point, fresh whole-fish samples were initially evaluated in triplicate for their nutritional profiles.

Brine salting and drying processes

Brine-salted fish samples were immersed in brine solution (15% *w/w*) at a ratio of 3:1 of fish to salt solution. For each sampling unit, samples were totally immersed and brined together in a closable plastic bucket (20 L) for 48 h at 4°C. The brine solution was pre-cooled to 4°C

prior to the immersion of samples. Afterwards, brined samples were drained from salt drips for 3 min using a plastic sieve and randomly distributed into individual aluminium trays (500 mL; 142 mm × 116 mm × 30.4 mm; Boni™, Halle, Belgium), at one fish per try, into which small beads (13 mm diameter, 3.5 g, JVLAB®) were added (10 beads/tray) at the bottom to avoid crusting of samples and optimize moisture loss. Likewise, the non-salted group of fish samples were randomly distributed into identical aluminium trays containing similar beads. To simulate environmental drying temperature in rural conditions, each tray was placed in an electric oven and randomly set and dried with the above-mentioned temperature points. Drying temperature between sampling units were controlled using an oven-fixed thermometer and a consistent oven temperature (± 1°C) was maintained throughout the drying time period. A constant air ventilation was supplied through an oven-fixed ventilator at a steady air velocity of 1.0 m/s. Drying time started after oven closure and the oven returned to its set temperature (~ 5 min). After 23 h, samples were collected and allowed to cool in a desiccator for 5 min to room temperature before weighing. Samples were finally freeze-dried (CoolSafe™, SCANVAC, Denmark) to stable weight, uniformly ground and homogenized using a grinder machine (De´Longhi™, KG48) at 12 × speed and then individually kept in closable plastic containers at 4°C until analyzed.

Nutrient evaluations

Proximate analysis

So as to make legitimate comparisons between the processed samples, the quantitative analysis of the values of macromolecules is a significant nutritional marker and for which case the proximate analysis was used to determine the crude protein (CP), ether extract and ash contents of fish samples. The DM content of freeze-dried samples (1 g) was determined through oven-drying at 105°C for 24 h to constant weight [24]. Ether-extract was determined using the acid hydrolysis method [24], CP using the Kjeldahl method involving a nitrogen analyser

(LECO FP528 Instruments, St. Joseph, Michigan, USA) and N × 6.25 conversion factor [25], and ash content using an automated muffle furnace (Nabertherm™, UK) as per the procedures described by AOAC [26]. A gram of fish sample was used for the analysis of each of the macronutrients and all samples were evaluated in duplicate at the animal nutrition laboratory of the Department of Veterinary and Biosciences, Ghent University, Belgium.

Mineral analysis

The total ash content was determined following the ISO method [27] and mineral (calcium, sodium, potassium, copper, iron, magnesium, manganese, phosphorus and zinc) concentrations of fish were evaluated after dry ashing mineralization according to ISO method (ICP-AES: ISO 11885) as per the procedures described by Gorsuch [28]. In brief, a gram of sample was weighed, placed into a porcelain crucible and oven-dried at 105°C for 3 h. Sample was then burnt on a hot plate and incinerated in a muffle furnace at 450°C for 16 h. Obtained ash was then moistened with a small amount of deionized water first and dissolved in 10 mL of 1:1 (*v/v*) solution of hydrochloric acid (HCl) and deionized water and evaporated to dry. Finally, the mix was re-dissolved in 10 mL of 1:9 (*v/v*) solution of HCl and deionized water, transferred into a 50 mL flask, and then diluted several folds for optimal determination of individual mineral concentration using induction-coupled plasma optical emission spectrometry (ICP-OES) method as per the manufacturer's procedures (Iris Intrepid II XSP, Thermo Fisher Scientific, Aalst, Belgium). Likewise, the concentration of selenium was determined by ISO method (ICP-MS: ISO 11885) and iodine with tetramethylammonium hydroxide extraction and iodine quantification (ICP-MS: ISO 11885) at the Laboratory of Chemical analysis, Ghent University, Belgium.

Amino acid and OMIU-reactive lysine analyses

The total amino acid profiling was done by acid hydrolysis (2 g) at 110°C for 23 h and ion-exchange chromatography with post-column derivatization with ninhydrin method (ISO

13903). Sulphur containing amino acids were measured as cysteic acid and methionine sulfone after oxidation with performic acid; tryptophan was measured by alkaline hydrolysis at 110°C for 20 h and ion-exchange chromatography with fluorescence detection method (ISO 13904) according to procedures described by Hulshof [29].

For analysis of reactive lysine, about 2 g of fish sample were processed from each sampling point for defatting by extraction with light petroleum ether without acid hydrolysis (ISO 6492), finely ground and homogenized using a mixer mill (Retsch MM200, BV) and used for the analysis of reactive lysine via O-methylisourea (OMIU) method according to Hulshof [29]. Briefly, 1 mL of 0.6 M solution of OMIU was prepared as described by Moughan and Rutherfurd [30] that converts lysine with free e-amino group to homoarginine. Four gram of barium hydroxide octahydrate (Sigma-Aldrich, Zwijndrecht, the Netherlands) was added to 16 mL of boiling (10 min) distilled deionized water to remove CO_2, after which 2 g of OMIU sulphate salt was added. The solution was then cooled for 30 min at room temperature, centrifuged (6,400 × g for 10 min at 20°C) and the supernatant was retained and the precipitate was washed with about 2 mL of same water and further centrifuged (6,400 × g for 10 min at 20°C). both supernatants were combined and pH adjusted to 11.5 (for guanidation) by adding 6 M HCl solution. The homoarginine content was analysed in duplicate and the OMIU-reactive lysine was calculated from the homoarginine content. All amino acid profile, including reactive lysine, were determined at Wageningen University & Research, Animal Nutrition laboratory, The Netherlands.

Statistical analysis

Data obtained in the current study were analyzed using SPSS statistical software version 27 (IBM Corporation, Armonk, NY, USA). All data were checked for normality distributions using histogram and QQ-plots. A univariate one-way ANOVA was used with brining as fixed factor and temperature as covariate to test the hypothesis about differences between two or more

mean nutrient values. All data were presented as means of three values and standard error of the means (SEM). Statistical significance was set at $P < 0.05$ for any differences observed between samples. Furthermore, multivariate analysis was computed using principal component analysis (PCA) on the collective factors obtained to appraise the relationships of nutrient profiles based on their responses towards brining and drying methods, with values deemed relevant below -0.5 and above 0.5 of the components' values.

Results

Proximate analysis

Drying temperature had no significant effect on the measured nutrient profiles of the fish in this study and all data were combined across temperature treatments ($P > 0.05$; Fig 1). However, several effects ($P < 0.05$) were observed with the brining method.

Fig 1. The effect of drying temperature on nutrient profiles of tambaqui fish. (a) gram of taurine per kg fish; (b) gram of reactive lysine per kg fish.

Brining resulted in a higher ash concentration with a relative increase of 28% ($P < 0.05$). Moreover, 6% loss on the overall protein concentration was noticed due to brining ($P < 0.05$). The body fat contents of tambaqui fish were not affected by the addition of salt and temperature treatments ($P > 0.05$; Table 1).

Table [illegible] Changes in macronutrient concentrations in tambaqui fish (DM basis) due to brining and drying at seven different temperatures (30, 35, 40, 45, 50, 55 and 60°C).

Concentration (g/100 g DM)	Salt		Total	Relative % Change	SEM	*P*	
	No	Yes				salted	temp
**Ash**	12.4	16.0	14.1	28	0.5	< 0.001	0.793
**Crude protein**	58.8	55.4	57.0	-6	0.6	0.004	0.846

**Crude fat**	28.3	28.7	28.7	1	0.4	0.716	0.657

DM, dry matter; SEM, standard error of the means; temp, temperature.

Values represent mean and standard error of the means based on three replicates per treatment and seven treatments; n = 48.

Minerals and amino acids

The essential microminerals Se and I, and the macrominerals K and Mg were significantly reduced by brining with 14, 38, 42 and 18% percent losses, respectively ($P < 0.05$). Moreover, brining resulted in an obvious enormous significant increase (744%) in Na concentration ($P < 0.05$). Otherwise, all minerals (Fe, Mn, P and Ca) were decreased by brining (Table 2).

Table 2. Changes in mineral concentrations in tambaqui fish due to brining and drying at seven different temperatures (30, 35, 40, 45, 50, 55 and 60˚C).

**Minerals**	**Salt**		**Total**	**Relative % Change**	**SEM**	***P***	
	**No**	**Yes**				**salted**	**temp**
**Macrominerals (g/kg DM)**							
**Ca**	37.4	37.1	37.0	-1	1.1	0.965	0.965
**K**	9.71	5.61	7.71	-42	0.6	<0.001	0.495
**Mg**	1.32	1.10	1.10	-18	0.03	<0.001	0.880
**Na**	2.70	22.9	12.1	744	2.7	<0.001	0.564
**P**	21.7	20.4	21.0	-6	0.6	0.317	0.814
**Microminerals (mg/kg DM)**							
**Fe**	42.0	36.9	39.2	-12	2.4	0.256	0.188
**I***	604	373	489	-38	49	0.020	0.264
**Mn**	9.80	8.81	9.31	-10	0.3	0.115	0.152
**Se***	149	128	137	-14	5	0.020	0.449
**Zn**	48.5	50.6	49.6	4	1.3	0.473	0.588

DM, dry matter; SEM, standard error of the means; temp, temperature.

Values represent mean and standard error of the means based on three replicates per treatment and seven treatments; n = 48.

*Values are presented in µg/kg DM.

Brining negatively affected the overall sum amino acid profile ($P < 0.05$). In particular, taurine, histidine, cysteine, methionine and available (reactive) lysine were affected by brining with the highest percent loss accounted for taurine (56%) followed by methionine (17%), cyteine (11%), and histidine (10%) ($P < 0.05$). In contrast, there was numerical 5 and 6% increase in proline and glycine respectively, whereas alanine and arginine were not affected by brining ($P > 0.05$; Table 3).

Table 3. Changes in amino acid concentrations in tambaqui fish due to brining and drying at seven different temperatures (30, 35, 40, 45, 50, 55 and 60°C).

**Amino acids (g/kg CP)**	**Salt**		**Total**	**Relative % Change**	**SEM**	***P***	
	**No**	**Yes**				**salted**	**temp**
Taurine	7.10	3.10	5.11	-56	0.5	< 0.001	0.324
Asparagine + Aspartic acid	56.8	54.9	55.4	-3	0.9	0.268	0.671
Threonine	27.0	26.3	26.5	-3	0.4	0.311	0.965
Serine	25.0	24.9	24.8	0	0.2	0.807	0.777
Glutamine + Glutamic acid	91.4	88.3	89.2	-3	1.3	0.247	0.851
Glycine	75.7	80.2	79.3	6	2.5	0.361	0.750
Alanine	51.1	51.0	51.2	0	0.7	0.961	0.636
Valine	30.8	29.2	29.8	-5	0.5	0.062	0.642
Isoleucine	26.0	24.6	24.9	-5	0.5	0.162	0.838
Leucine	44.3	41.8	42.5	-6	0.9	0.134	0.855
Tyrosine	17.9	16.5	16.9	-8	0.5	0.098	0.952
Phenylalanine	24.5	23.4	23.8	-5	0.4	0.134	0.874
Histidine	15.5	14.0	14.6	-10	0.4	0.054	0.400
Lysine	51.1	47.4	48.6	-7	1.1	0.070	0.863
Arginine	48.3	48.3	48.5	0	0.5	0.999	0.464
Proline	47.5	49.9	49.5	5	1.3	0.356	0.660
Cysteine	5.10	4.40	4.60	-11	0.1	0.016	0.746
Methionine	14.7	12.1	13.3	-17	0.4	< 0.001	0.675
Tryptophan	5.10	4.60	4.71	-8	0.2	0.229	0.698

Reactive lysine[a]	52.0	46.4	48.5	-11	1.2	0.004	0.851
% Reactive lysine[b]	102.2	97.9	99.9	-4	1.8	0.306	0.760
Sum AA	665	645	653	-3	6.87	0.017	0.621

CP, crude protein; AA, amino acid; SEM, standard error of the means; temp, temperature.

Values represent mean and standard error of the means based on three replicates per treatment and seven treatments; n = 48.

[a]Is the percentage of reactive lysine in the total protein.

[b]Is the percentage of reactive lysine in the total lysine.

Multivariate classification of variables

Multivariate statistical analysis (PCA) was computed on the collective factors obtained to appraise the relationships of nutrient profiles based on their responses towards brining and drying methods. In line, there were no associations between nutrient profiles and drying temperature while several associations were observed with brining. Negative association was seen between brining, Na and ash on one side of component 1 and most other nutrients on the other. However, positive association between the brining proxies (Na, ash) and amino acids prominent in connective tissue (glycine, proline, arginine, alanine) were typically seen clustered together right of the component. The more central position of Zn, Fe, fat, and available Lys point to their low response to brining and temperature. More importantly, temperature itself was positioned centrally in the plot, emphasizing the absence of drying temperature effects on the study parameters (Fig 2).

Fig 2. Multivariate classification of nutrient profiles based on their response to brining and temperature. Analyses represent principal component analysis (PCA) of evaluated nutrient values (expressed as percentages of the sum of total) after brining and drying of whole tambaqui fish samples (n = 48). AvailableLYS, is the percentage of reactive lysine in the total protein; availablelys_A, is the percentage of reactive lysine in the total lysine; saltcode, refers to brining; temp, is the drying temperature.

Discussion

In our study, brining induced pronounced losses of specific amino acids and minerals, irrespective of drying temperature. Salt, a hygroscopic compound, can absorb water molecules from muscle tissues and cause protein damage [5,11]. The causes for protein damage can be related to direct cell membrane disruption, decreased activity of antioxidant enzymes and the increased release of ferric (Fe^{3+}) ions and formation of ferryl-myoglobin and metmyoglobin in damaged tissue [31,32]. Gill et al. [33] found that salting enhanced the aggregation of myosin in cod, yet only above a drying temperature of 50°C. Marínez-Alvarez and Gómez-Guillén [14] indicated that there is a notable loss of soluble muscle proteins (particularly, actin and myosin) by osmosis in salting (NaCl) at pH 6.5 on cod muscle (*Gadus morhua*). Indeed, drying of fish products at higher temperatures, of more than 60°C, resulted in a proportional decrease in protein solubility, and cause fat oxidation and caramelization of sugars [18]. Differences between studies can evolve from a wide range of conditions used in the drying process including the drying type, drying temperature and duration, relative humidity, air velocity and the specific characteristics of the product dried (species and size of the fish). Although comparison with existing literature does not allow us to pinpoint the factors that protected from a drying temperature effect, it is clear that brining completely overruled the drying temperature in its effect on nutrient loss. In the present study, a total of 6% tissue proteins was lost due to the brining stress. In fact, salt concentration and salting duration can modulate the effect of brining on muscular protein loss in fish [9-11]. The solubility of muscle proteins often start to decrease with increasing salt concentration due to protein aggregation [5] and/or altered enzymatic activities [34]. In the present study, however, an acceptable (15% *w/w*) salt was used for brining [12].

The amino acid analysis in our study demonstrated that the salt-induced protein loss was not an overall effect, but reduced specific amino acids, taurine, histidine, cysteine, methionine

and available lysine. Taurine is not involved in protein synthesis but often accumulates in muscle tissues of fish and has osmolytic properties [35,36]. Hence, the loss in taurine seem a logical reaction to the osmotic stress from brining. This taurine loss can be important since its role in health has been demonstrated for instance in infants [37] and it is also the main amino acid to form conjugated bile acids in chickens [38].

The tendency to a lower histidine concentration due to brining can be seen in the same light. Apart from its protein-bound appearance, histidine is stored in muscular tissue as either free histidine (acetylated or not) or bound to mainly beta-alanine (hence constituting dipeptides such as carnosine or anserine); this storage of histidine occurs throughout the animal kingdom [39]. Shiau [40] reported that histidine can easily get released from muscle tissues during stress conditions such as starvation. Significant losses of the free amino acids histidine, taurine and glycine were reported in salted milkfish [13]. Histidine is involved in the regulation and metabolism of essential trace minerals, formation of enzymes, induction of the immune system through the production of histamine and protects brain cells through the formation of metallothionein among others [41].

Cysteine and methionine are sulphur-containing amino acids and quite reactive to processing, in that they could either get directly affected by the ionic brine solution or can possibly make cross-links that reduces their tissue availability [13]. Kumazawa [42] reported that brining may cause cross-linking reactions of muscle myosin heavy chains and form ε-(γ-glutamyl)lysine through transglutaminase. There was no effect on the percentage of reactive lysine (% available-lysine), meaning that both salting and drying did not induce Maillard reactions that affect lysine availability. The relative decrease in the total lysine likely explains the decreased percentage of available lysine in the current study.

Four specific amino acids in the principal component analysis separated from the negative association between brining and the other amino acids: alanine and arginine were not affected

while glycine and proline were even positively associated with brining. These amino acids are prominent in the skeleton and in scales, which is a strong indication that the highest protein losses were from muscle and other soft tissues [43]. Other authors not always report similar changes in amino acid pattern: a sigmoid initial increase (up to 9 h) and final slight decrease (48 h) of alanine, glutamine and lysine was reported in salted milkfish [13]; alanine, glycine, lysine and histidine increased when salted fish was dried [44]. The increase in these amino acid concentrations could be due to the decrease in the proportion of the extracted total lipids present as polyunsaturated fatty acids as depicted by Yankah [44]. Fat is a hydrophobic molecule and can act as a physical barrier to limit salt (NaCl) and water transport across the muscle tissue [4]. In fact, the lipid content was higher for the fish in our study (28.71%) although fat concentration was not affected by both treatments.

The specific loss of soft tissue from the fish is not only supported by the amino acid data, but also witnessed by the change in mineral profile. The considerable loss of the microminerals Se and I and the macrominerals K and Mg following brining reflects the presence of these minerals in soft tissue [45]. Selenium typically occurs as replacement of sulphur in sulphur-containing amino acids (cysteine and methionine) in soft tissues. The decrease in methionine and cysteine seen in our study, therefore, fits the observation of decreased selenium. In contrast, the typical skeletal minerals such as Ca, P and Mg, and those that are deposited in scales and fins such as Zn and Fe, were not affected. Potassium is a strong electrolyte that could disappear in the brine solution or get filtered out in exchange with the added sodium. The loss of water due to salt was explained as the main cause for leaching of these macrominerals in the solution during brining. The decrease in K concentration agrees with Polak-Juszczak [46] and Bakhiet [47] from salted fish. The latter study remarkably reported a salt-induced increase in iodine concentration, most likely evolving from salt impurities as proposed by the authors. It is not clear how these differences with our study can be explained, but we used a purified source of

salt, hence excluding potential unintended supply of microminerals to the fish tissues through brining.

Drying is an energy intensive process which can use about 10% or more of the total energy consumed during processing. In the present study, although our aim was not to quantify the drying efficiency, we conjointly were aiming to consume less energy while drying samples by increasing the sample surface area of exposure, avoiding surface contact by putting beads, improved equipment insulation during and between drying, supply of a constant air ventilation at 1.0 m/s during drying, and minimizing the timing between dryings [48].

Undeniably, brining has substantially increased Na and ash concentrations. This has been observed in most studies on brining of fish [46,47]. This enrichment with salt through brining must be considered when formulating diets but, more importantly, our results demonstrate that brining may not be the best choice to maintain the nutritive value of fish. Evidently, we must acknowledge that nutritive value is not the only criterion to pick a preservation method for fish: for the reduction of the pathogenic load in fish products, salt addition may still be a valuable technique that goes, however, at the expense of nutritive value as shown in our study. A comforting finding is that the wide range in drying temperature evaluated in our study had no noticeable impact on the nutritive value of the fish. This implies that drying of fish under uncontrolled environmental temperature seems a suitable option in rural areas to preserve the nutritive value of (tambaqui) fish.

5. Conclusions

The lack of a drying temperature effect on fish composition in this study is encouraging for drying of fish under – less controlled – rural tropical conditions. Brining, however, reduced some important micronutrients such as Se and I, and lead to important loss of muscle tissue, with the concomitant amino acids. The formation of Maillard products was not promoted by the drying and brining treatments, as demonstrated through unaltered reactive lysine

proportions. Brining also increased the leaching of K, I and taurine. Drying without brining may therefore better preserve the nutritive value of tambaqui fish, although this must be considered in combination with the goal of pathogen reduction.

Acknowledgments

The authors acknowledge Herman De Rycke for the proximate analysis.

References

1. Pawar SM, Sonawane SR. Fish muscle protein highest source of energy. Int J Biodivers Conserv. 2013; 5:433–435. doi: 10.5897/IJBC12.043.
2. di Domenico CNB. Fish drying: A review. Rev Bras Eng Pesca. 2014; 7:1–18. doi: 10.18817/repesca.v7i1.1061.
3. Binici A, Kaya GK. Effect of brine and dry salting methods on the physicochemical and microbial quality of chub (*Squalius cephalus* Linnaeus, 1758). Food Sci Technol. 2018; 38:66–70. doi: 10.1590/1678-457X.15717.
4. Gallart-Jornet L, Barat JM, Rustad T, Erikson U, Escriche I, Fito P. A comparative study of brine salting of Atlantic cod (*Gadus morhua*) and Atlantic salmon (*Salmo salar*). J Food Eng. 2007; 79:261–270. doi: 10.1016/j.jfoodeng.2006.01.053.
5. Nguyen MV, Thorarinsdottir KA, Gudmundsdottir A, Thorkelsson G, Arason S. The effects of salt concentration on conformational changes in cod (*Gadus morhua*) proteins during brine salting. Food Chem. 2011; 125:1013–1019. doi: 10.1016/j.foodchem.2010.09.109.
6. Rust RE, Knipe CL. Sausage casings. In: Devine C, Dikeman M, editors. Encyclopedia of Meat Sciences. Academic Press: Oxford; 2014. pp. 235–240.

7. Thorarinsdottir KA, Arason S, Bogason S, Kristbergsson K. The effects of various salt concentrations during brine curing of cod (Gadus morhua). Int J Food Sci Technol. 2004; 39(1):79–89. doi: 10.1046/j.0950-5423.2003.00757.x.

8. Thorarinsdottir KA, Arason S, Sigurgisladottir S, Valsdottir T, Tornberg E. Effects of different pre-salting methods on protein aggregation during heavy salting of cod fillets. Food Chem. 2011; 124(1):7–14. doi: 10.1016/j.foodchem.2010.05.095.

9. Barat JM, Rodríguez-Barona S, Andrés A, Fito P. Influence of increasing brine concentration in the cod salting process. J Food Sci. 2002; 67(5):1922–1925. doi: 10.1111/j.1365-2621.2002.tb08747.x.

10. Larsen R, Elvevoll EO. Water uptake, drip losses and retention of free amino acids and minerals in cod (*Gadus morhua*) fillet immersed in NaCl or KCl. Food Chem. 2008; 107(1):369–376. doi: 10.1016/j.foodchem.2007.08.031.

11. Thorarinsdottir KA, Arason S, Geirsdottir M, Bogason SG, Kristbergsson K. Changes in myofibrillar proteins during processing of salted cod (*Gadus morhua*) as determined by electrophoresis and differential scanning calorimetry. Food Chem. 2002; 77(3):377–385. doi: 10.1016/S0308-8146(01)00349-1.

12. FSSA. Fish and fish products regulations. In: Gaur VN, editors. Food Safety and Standards Regulation Authority (FSSA). New Delhi: The gazette of India, 2011. pp. 366-369.

13. Sannaveerappa T, Ammu K, Joseph J. Protein-related changes during salting of milkfish (*Chanos chanos*). J Sci Food Agric. 2004; 84(8):863–869. doi: 10.1002/jsfa.1682.

14. Marínez-Alvarez O, Gómez-Guillén MC. The effect of brine composition and pH on the yield and nature of water-soluble proteins extractable from brined muscle of cod (*Gadus morhua*). Food Chem. 2005; 92(1):71–77. doi: 10.1016/j.foodchem.2004.04.049.

15. Ockerman HW, Basu L. Preservation methods of animal products. In: Dikeman M, Devine C, editors. Encyclopedia of Meat Sciences. London: Oxford Elsevier Ltd.; 2014. pp. 78–83. doi: 10.1016/B978-0-12-384731-7.00207-5.

16. Kim JC, Mullan BP, Pluske JR. Prediction of apparent, standardized, and true ileal digestible total and reactive lysine contents in heat-damaged soybean meal samples. J Anim Sci. 2012; 90:137–139. doi: 10.2527/jas.53819.

17. Lantry BF, O'Gorman R. Drying temperature effects on fish dry mass measurements. J Great Lakes Res. 2004; 33:606–616. doi: 10.3394/0380-1330(2007)33[606:DTEOFD]2.0.CO;2.

18. Bastos JR. Influência da secagem sobre algumas propriedades físico-químicas do músculo do cação branco, Carcharhynus porosus Ranzani (in portuguess, with english abstract). Arquivos de Ciências do Mar. 1997; 17:77–87. Available from: http://www.repositorio.ufc.br/handle/riufc/1403

19. Pacheco N, Garnica-Gonzalez M, Gimeno M, Bárzana E, Trombotto S, David L, et al. Structural characterization of chitin and chitosan obtained by biological and chemical methods. Biomacromolecules. 2011; 12:3285–3290. doi: 10.1021/bm200750t.

20. Vose RS, Easterling DR, Gleason B. Maximum and minimum temperature trends for the globe: An update through 2004. Geophys Res Lett. 2005; 32:1–5. doi: 10.1029/2005GL024379.

21. Fiúza LS, Aragão NMS, Ribeiro-Junior HP, Moraes MG, Rocha IRCB, Lustosa-Neto AD, et al. Effects of salinity on the growth, survival, haematological parameters and osmoregulation of Tambaqui Colossoma macropomum juveniles. Aquac Res. 2015; 46:1–9. doi: 10.1111/are.12224.

22. Saint-Paul U. Native Fish species boosting Brazilian's aquaculture development. Acta Fish Aquat Res. 2017; 5:1–9. doi: 10.2312/ActaFish.2017.5.1.1-9.

23. Lewbart GA. Euthanasia of ectotherms. In: Fowler's zoo and wild animal medicine current therapy. Elsevier. 2019; 9:357–363. doi: 10.1016/b978-0-323-55228-8.00051-5.

24. AOAC. Official Methods of Analysis of the Association of Official Analytical Chemists. 16th ed. Arlington VA; 1995.

25. AOAC. Official Methods of Analysis of the Association of Official Analytical Chemists. 17th ed. Washington DC: Gaithersburg publishing; 2001.

26. AOAC. Official Methods of Analysis of the Association of Official Analytical Chemists. 15th ed. Arlington VA; 1990.

27. ISO. Meat and Meat Products - Determination of total ash. In: International Organization for Standardization. Switzerland: ISO/936; 1998. Available from: https://www.iso.org/standard/24783.html

28. Gorsuch TT. The Destruction of organic matter. 1st ed. New York: Oxford pergamon press Ltd; 1970. pp. 40–54.

29. Hulshof TG, Bikker P, Van der Poel AFB, Hendriks WH. Assessment of protein quality of soybean meal and 00-rapeseed meal toasted in the presence of lignosulfonate by amino acid digestibility in growing pigs and Maillard reaction products. J Anim Sci. 2016; 94:1020–1030. doi: 10.2527/jas.2015-9700.

30. Moughan PJ, Gall MPJ, Rutherfurd SM. Absorption of lysine and deoxyketosyllysine in an early Maillard browned casein by the growing pig. J Agric Food Chem. 1996; 44:1520–1525. doi: 10.1021/jf950428v.

31. Li Y, Xue C, Quan W, Qin F, Wang Z, et al. Assessment the influence of salt and polyphosphate on protein oxidation and Nε-(carboxymethyl)lysine and Nε-(carboxyethyl)lysine formation in roasted beef patties. Meat Sci. 2021; 177:108489. doi: 10.1016/j.meatsci.2021.108489.

32. Soladoye OP, Juárez ML, Aalhus JL, Shand P, Estévez M. Protein oxidation in processed meat: Mechanisms and potential implications on human health. Compr Rev Food Sci Food Saf. 2015; 14:106–122. doi: 10.1111/1541-4337.12127.

33. Gill TA, Chan TK, Phozchareon KF, Paulson AT. Effect of salt concentration and temperature on heat-induced aggregation and gelation of fish myosin. Food Res Int. 1992; 25:333–341. doi: 10.1016/0963-9969(92)90108-H.

34. Stoknes IS, Walde PM, Synnes M. Proteolytic activity in cod (*Gadus morhua*) muscle during salt curing. Food Res Int. 2005; 38*:*693–699. doi: 10.1016/j.foodres.2005.02.003.

35. Geda F, Declercq AM, Remø SC, Lourenço RWM, Janssens GPJ. The metabolic response in fish to mildly elevated water temperature relates to species-dependent muscular concentrations of imidazole compounds and free amino acids. J Therm Biol. 2017; 65:57-63. doi: 10.1016/j.jtherbio.2017.02.004.

36. Zhang Y, Dabrowski K, Hliwa P, Gomulka P. Indispensable amino acid concentrations decrease in tissues of stomachless fish, common carp in response to free amino acid- or peptide-based diets. Amino Acids. 2006; 31(2):165–172. doi: 10.1007/s00726-006-0345-7.

37. Chesney RW, Helms RA, Christensen M, Budreau AM, Han X, Sturman JA. The role of taurine in infant nutrition. Adv Exp Med Biol. 1998; 442:463–476. doi: 10.1007/978-1-4899-0117-0_56.

38. Alzawqari MH, Al-Baadani HH, Alhidary IB, Al-Owaimer AN, Abudabos AM. Effect of taurine and bile acid supplementation and their interaction on performance, serum components, ileal viscosity and carcass characteristics of broiler chickens. S Afr J Anim Sci. 2016; 46(4):448–457. doi: 10.4314/sajas.v46i4.13.

39. Boldyrev AA, Aldini G, Derave W. Physiology and pathophysiology of carnosine. Physiol Rev. 2013; 93:1803–1845. doi: 10.1152/physrev.00039.2012.

40. Shiau CY, Pong YJ, Chiou TK, Tin YY. Effect of starvation on free histidine and amino acids in white muscle of milkfish *Chanos chanos*. Comp Biochem Physiol B: Biochem Mol Biol. 2001; 128:501–506. doi: 10.1016/S1096-4959(00)00350-X.

41. Kopeć W, Jamroz D, Wiliczkiewicz A, Biazik E, Pudlo A, Hikawczuk T, et al. Influence of different histidine sources and zinc supplementation of broiler diets on dipeptide content and antioxidant status of blood and meat. Br Poult Sci. 2013; 54:454–465. doi: 10.1080/00071668.2013.793295.

42. Kumazawa K, Seguro K, Takamura M, Motoki M. Formation of ε-(γ-glutamyl)lysine cross-link in cured horse mackerel meat induced by drying. J Food Sci. 1993; 58:1062–1064. doi: 10.1111/j.1365-2621.1993.tb06112.x.

43. Fedina IS, Georgieva K, Grigorova I. Light-dark changes in proline content of barley leaves under salt stress. Biol Plant. 2002; 45:59–63. doi: 10.1023/A:1015175802847.

44. Yankah MV, Ohshima T, Koizumi C. Effects of processing and storage on some chemical characteristics and lipid composition of a Ghanian fermented fish product. J Sci Food Agric. 1993; 63:227–235. doi: 10.1002/jsfa.2740630209.

45. Yamashita Y, Yamashita M, Iida H. Selenium Content in Seafood in Japan. Nutrients. 2013; 5:388–395. doi: 10.3390/nu5020388.

46. Polak-Juszczak L. Effects of processing methods on the content of minerals in fish products. J Elem. 2016; 21:461-470. doi: 10.5601/jelem.2015.20.3.890.

47. Bakhiet HHA, Khogali FAH, Ahmed FAM. Effect of different salt concentrations on mineral content of the fish (*Hydrocynus* spp.). J Life Sci Biomed. 2013; 3(1):01–03.

48. Pask F, Lake P, Yang A, Tokos H, Sadhukhan J. Industrial oven improvement for energy reduction and enhanced process performance. Clean Technol Environ Policy. 2017; 19:215-224. doi: 10.1007/s10098-016-1206-z.

Fig 1.

Click here to access/download;Figure;Fig 1.tif

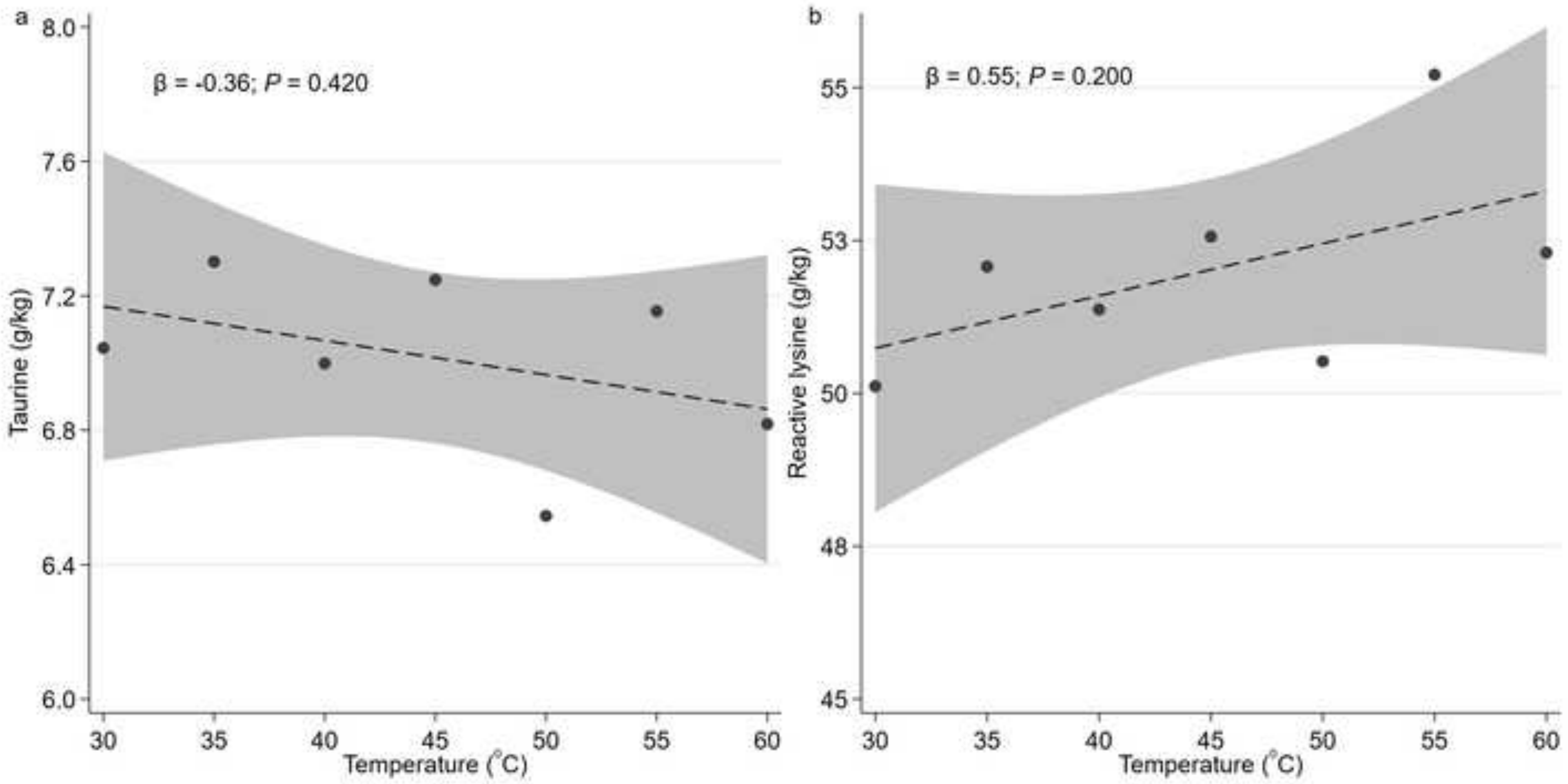

Fig 2.

Click here to access/download;Figure;Fig 2.tif

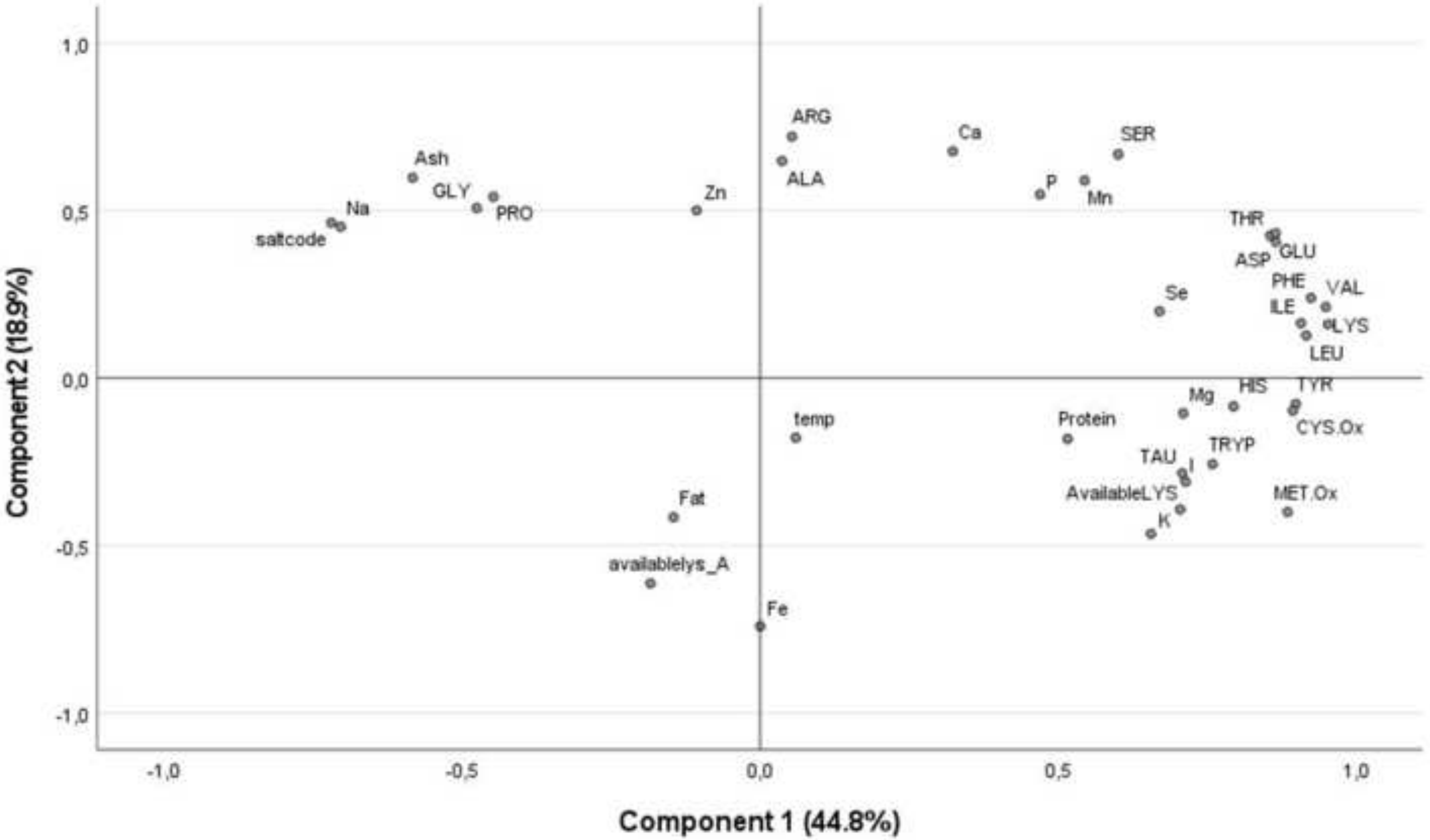

Supplement: Attachment — Submitted filename: PONE-D-22-35077_EA.pdf [file pone.0299926.s008.pdf]
