# Supplementary material for: Impact of brining and drying processes on the nutritive value of tambaqui fish (Colossoma macropomum)
Source: PLoS One. 2024 Apr 16;19(4):e0299926. doi: 10.1371/journal.pone.0299926 (PMC11020844; doi:10.1371/journal.pone.0299926)
Supplement: Attachment — Submitted filename: PONE-D-22-35077_R1.pdf [file pone.0299926.s010.pdf]

PLOS ONE

Impact of brining and drying processes on the nutritive value of tambaqui fish (Colossoma macropomum)

--Manuscript Draft--

**Manuscript Number:**	PONE-D-22-35077R1
**Article Type:**	Research Article
**Full Title:**	Impact of brining and drying processes on the nutritive value of tambaqui fish (Colossoma macropomum)
**Short Title:**	Effect of brining and drying on nutritive value of tambaqui fish.
**Corresponding Author:**	Awot Teklu MEBRATU<br>Universiteit Gent<br>Merelbeke, BELGIUM
**Keywords:**	brining; drying; nutrient profiles; tambaqui; reactive lysine
**Abstract:**	Preservation of fish as diet ingredient is challenging in many tropical conditions due to the absence of freezing facilities, hence warranting other preservation techniques. Brine salting is one of the oldest traditional and economically viable preservation methods. The present study evaluated the effect of brine salting (15% w/w) prior to drying at different temperatures on the nutrient profiles of tambaqui fish (Colossoma macropomum). Whole fish samples (792 ± 16 g; 8 months old; n = 48) were grouped into two as brine-salted and non-salted, and treated at seven different drying temperatures of 30, 35, 40, 45, 50, 55 and 60°C for a period of 23 h each. To evaluate the impact of Maillard reaction, reactive lysine was also quantified. Drying temperature had no effect on the nutrient profiles of tambaqui fish (P > 0.05) while brining reduced the overall protein concentration by 6% (58.8 to 55.4 g/100 g DM; P = 0.004). Brining significantly diminished many amino acids taurine by 56% (7.1 to 3.1 g/kg; P < 0.001), methionine 17% (14.7 to 12.1 g/kg; P < 0.001), cysteine 11% (5.1 to 4.4 g/kg, P = 0.016), and reactive lysine 11% (52.0 to 46.4 g/kg; P = 0.004). However, alanine, arginine, and serine were not affected by brining (P > 0.05). Brining also reduced the concentrations of Se by 14% (149 to 128 µg/kg dry matter; P = 0.020), I 38% (604 to 373 µg/kg DM; P = 0.020), K 42% (9.71 to 5.61 g/kg DM; P < 0.001) and Mg 18% (1.32 to 1.10 g/kg DM; P = < 0.001) versus an anticipated vast increase in Na by 744% (2.70 to 22.90 g/kg DM; P < 0.001) and ash 28% (12.4 to 16.0 g/100g DM; P < 0.001) concentration. Neither brining nor drying temperature induced changes in lysine reactivity and fat content of tambaqui fish (P > 0.05). Agreeably, results of multivariate analysis showed a negative association between brining, Na, and ash on one side of the component and most other nutrients on the other while the lack of effect of temperature can be seen by its presence in the center. Therefore, drying without brining may better preserve the nutritive value of tambaqui fish. However, as a practical to the industry sector, it is recommended that the final products are further evaluated for any pathogen of economic or public health importance.
**Order of Authors:**	Awot Teklu MEBRATU
	Yohannes Tekle Asfaw
	Wouter Merckx
	Wouter Hendriks
	Geert Janssens
**Response to Reviewers:**	Dear Editor and Reviewers,<br>We, the authors, are thankful for your time in reviewing our manuscript. The comments given by the reviewers and editors were highly valuable for the quality of our manuscript. Now, to the best of our knowledge, we have tried to incorporate all the comments given and address them with rebutal letter one-byone.<br>Thank you for your time,
**Additional Information:**	
**Question**	**Response**

Powered by Editorial Manager® and ProduXion Manager® from Aries Systems Corporation

**Financial Disclosure**<br><br>Enter a financial disclosure statement that describes the sources of funding for the work included in this submission. Review the submission guidelines for detailed requirements. View published research articles from *PLOS ONE* for specific examples.<br><br>This statement is required for submission and **will appear in the published article** if the submission is accepted. Please make sure it is accurate.<br><br>**Unfunded studies**<br>Enter: *The author(s) received no specific funding for this work.*<br><br>**Funded studies**<br>Enter a statement with the following details:<br>• Initials of the authors who received each award<br>• Grant numbers awarded to each author<br>• The full name of each funder<br>• URL of each funder website<br>• Did the sponsors or funders play any role in the study design, data collection and analysis, decision to publish, or preparation of the manuscript?<br>• **NO** - Include this sentence at the end of your statement: *The funders had no role in study design, data collection and analysis, decision to publish, or preparation of the manuscript.*<br>• **YES** - Specify the role(s) played.<br><br>* typeset	The project was financially supported by the VLIR-UOS TEAM project under the title 'Win-win protein: reconciling fish protein generation and water quality management' [grant number ET2018TEA462A101] through Geert P.J Janssens and Luc De Meester; https://www.vliruos.be/en/home/1. The funders had no role in study design, data collection and analysis, decision to publish, or preparation of the manuscript. ATM received support in form of salary while doing the research activities.
**Competing Interests**<br><br>Use the instructions below to enter a competing interest statement for this submission. On behalf of all authors, disclose any competing interests that could be perceived to bias this work—acknowledging all financial support and any other relevant financial or non-financial competing interests.<br><br>This statement is required for submission	The authors have declared that no competing interests exist.

and **will appear in the published article** if the submission is accepted. Please make sure it is accurate and that any funding sources listed in your Funding Information later in the submission form are also declared in your Financial Disclosure statement.<br><br>View published research articles from *PLOS ONE* for specific examples.<br><br>**NO authors have competing interests**<br><br>Enter: *The authors have declared that no competing interests exist.*<br><br>**Authors with competing interests**<br><br>Enter competing interest details beginning with this statement:<br><br>*I have read the journal's policy and the authors of this manuscript have the following competing interests: [insert competing interests here]*<br><br>* typeset	
**Ethics Statement**<br><br>Enter an ethics statement for this submission. This statement is required if the study involved:<br><br>• Human participants<br>• Human specimens or tissue<br>• Vertebrate animals or cephalopods<br>• Vertebrate embryos or tissues<br>• Field research<br><br>Write "N/A" if the submission does not require an ethics statement.<br><br>General guidance is provided below. Consult the submission guidelines for detailed instructions. **Make sure that all information entered here is included in the Methods section of the manuscript.**	All experimental and animal care procedures were realized based upon the current written knowledge of aquaculture technologies and tambaquí fish farming. Fingerlings of Colossoma macropomum were initially brought from Aqua-Koi NV farm (http://www.aqua-koi.be/documents/home.xml?lang=nl) to TRANSfarm and reared in a recirculating aquaculture system (RAS) with a weekly basis 30% water renewal (20L/day) for about 7 months period before being subject to the current study. During their stay, fish were kept in larger aqua-tanks at 1.2 fish/m3. Tanks were fixed with individual bio-filters, aerated, temperature supplied, 18:6 h light - dark cycle, and supplying pipes were regularly inspected and cleaned with tap water and water quality parameters were checked twice a week and water renewed accordingly [39].<br>For our experimental study, after a 12 h fasting, a total of 48 tambaqui fish were randomly selected and kept in a separate tank and were euthanized in a two-step procedure [40]. Six fish were randomly taken at a time and placed in a tank with a volume of 240 L water into which 50 mL of phenoxyethanol-solution was added. After five minutes, 30 L of water were elevated out of the tanks and put into a large bucket. Another 6 mL of Anest Fish© was added to this bucket. Each group of six unconscious fish was scooped out of the tank and brought to the slaughter room in the bucket with the anesthetic agent. Thereafter, tambaqui were killed with a firm blow to the head, followed by fracturing the brainstem with a knife and then transported in ice cubes for the experiment [40]. The study was conducted in accordance with the Declaration of Helsinki and directive 2010/63/EU of the European parliament and of the council on the protection of animals used for scientific purposes, and the wellbeing of the cultured tambaqui fish before being subjected to the experiment was assessed and approved

Powered by Editorial Manager® and ProduXion Manager® from Aries Systems Corporation

**Format for specific study types**<br><br>**Human Subject Research (involving human participants and/or tissue)**<br>• Give the name of the institutional review board or ethics committee that approved the study<br>• Include the approval number and/or a statement indicating approval of this research<br>• Indicate the form of consent obtained (written/oral) or the reason that consent was not obtained (e.g. the data were analyzed anonymously)<br><br>**Animal Research (involving vertebrate animals, embryos or tissues)**<br>• Provide the name of the Institutional Animal Care and Use Committee (IACUC) or other relevant ethics board that reviewed the study protocol, and indicate whether they approved this research or granted a formal waiver of ethical approval<br>• Include an approval number if one was obtained<br>• If the study involved *non-human primates*, add *additional details* about animal welfare and steps taken to ameliorate suffering<br>• If anesthesia, euthanasia, or any kind of animal sacrifice is part of the study, include briefly which substances and/or methods were applied<br><br>**Field Research**<br><br>Include the following details if this study involves the collection of plant, animal, or other materials from a natural setting:<br>• Field permit number<br>• Name of the institution or relevant body that granted permission	by the institutional review board of TRANSfarm, KU Leuven, lab accreditation (KU/LA-1210616).
**Data Availability**<br><br>Authors are required to make all data underlying the findings described fully available, without restriction, and from the time of publication. PLOS allows rare exceptions to address legal and ethical concerns. See the PLOS Data Policy and FAQ for detailed information.	Yes - all data are fully available without restriction

Powered by Editorial Manager® and ProduXion Manager® from Aries Systems Corporation

A Data Availability Statement describing where the data can be found is required at submission. Your answers to this question constitute the Data Availability Statement and **will be published in the article**, if accepted.<br><br>**Important:** Stating 'data available on request from the author' is not sufficient. If your data are only available upon request, select 'No' for the first question and explain your exceptional situation in the text box.<br><br>Do the authors confirm that all data underlying the findings described in their manuscript are fully available without restriction?	
**Describe where the data may be found in full sentences. If you are copying our sample text, replace any instances of XXX with the appropriate details.**<br><br>• If the data are **held or will be held in a public repository**, include URLs, accession numbers or DOIs. If this information will only be available after acceptance, indicate this by ticking the box below. For example: *All XXX files are available from the XXX database (accession number(s) XXX, XXX.)*.<br>• If the data are all contained **within the manuscript and/or Supporting Information files**, enter the following: *All relevant data are within the manuscript and its Supporting Information files.*<br>• If neither of these applies but you are able to provide **details of access elsewhere**, with or without limitations, please do so. For example:<br><br>*Data cannot be shared publicly because of [XXX]. Data are available from the XXX Institutional Data Access / Ethics Committee (contact via XXX) for researchers who meet the criteria for access to confidential data.*<br><br>*The data underlying the results presented in the study are available from (include the name of the third party*	The data underlying the results presented in the study are all uploaded as supplementary information files with the manuscript.

and contact information or URL).<br>• This text is appropriate if the data are owned by a third party and authors do not have permission to share the data.<br><br>* typeset	
Additional data availability information:	

Powered by Editorial Manager® and ProduXion Manager® from Aries Systems Corporation

Manuscript Click here to access/download;Manuscript;Manuscript.docx

Impact of brining and drying processes on the nutritive value of tambaqui fish (*Colossoma macropomum*)

Awot Teklu Mebratu[1,2*], Yohannes Tekle Asfaw[3], Wouter Merckx[4], Wouter H. Hendriks[5], Geert P.J. Janssens[1]

[1]Department of Veterinary and Biosciences, Faculty of Veterinary Medicine, Ghent University, Merelbeke, Belgium

[2]Department of Animal Reproduction and Welfare, College of Veterinary Sciences, Mekelle University, Mekelle, Ethiopia

[3]Department of Veterinary Basic and Diagnostic Sciences, College of Veterinary Sciences, Mekelle University, Mekelle, Ethiopia

[4]TRANSfarm, Engineering and technology group, The Catholic University of Leuven, Lovenjoel, Belgium

[5]Animal Nutrition Group, Wageningen University, Wageningen, The Netherlands

[*]Corresponding author

Email: awotteklu.mebratu@ugent.be (ATM)

Abstract

Preservation of fish as diet ingredient is challenging in many tropical conditions due to the absence of freezing facilities, hence warranting other preservation techniques. Brine salting is one of the oldest traditional and economically viable preservation methods. The present study evaluated the effect of brine salting (15% *w/w*) prior to drying at different temperatures on the nutrient profiles of tambaqui fish (*Colossoma macropomum*). Whole fish samples (792 ± 16 g; 8 months old; n = 48) were grouped into two as brine-salted and non-salted, and treated at seven different drying temperatures of 30, 35, 40, 45, 50, 55 and 60°C for a period of 23 h each. To evaluate the impact of Maillard reaction, reactive lysine was also quantified. Drying temperature had no effect on the nutrient profiles of tambaqui fish ($P > 0.05$) while brining reduced the overall protein concentration by 6% (58.8 to 55.4 g/100 g DM; $P = 0.004$). Brining significantly diminished many amino acids taurine by 56% (7.1 to 3.1 g/kg; $P < 0.001$), methionine 17% (14.7 to 12.1 g/kg; $P < 0.001$), cysteine 11% (5.1 to 4.4 g/kg, $P = 0.016$), and reactive lysine 11% (52.0 to 46.4 g/kg; $P = 0.004$). However, alanine, arginine, and serine were not affected by brining ($P > 0.05$). Brining also reduced the concentrations of Se by 14% (149 to 128 µg/kg dry matter; $P = 0.020$), I 38% (604 to 373 µg/kg DM; $P = 0.020$), K 42% (9.71 to 5.61 g/kg DM; $P < 0.001$) and Mg 18% (1.32 to 1.10 g/kg DM; $P = < 0.001$) versus an anticipated vast increase in Na by 744% (2.70 to 22.90 g/kg DM; $P < 0.001$) and ash 28% (12.4 to 16.0 g/100g DM; $P < 0.001$) concentration. Neither brining nor drying temperature induced changes in lysine reactivity and fat content of tambaqui fish ($P > 0.05$). Agreeably, results of multivariate analysis showed a negative association between brining, Na, and ash on one side of the component and most other nutrients on the other while the lack of effect of temperature can be seen by its presence in the center. Therefore, drying without brining may better preserve the nutritive value of tambaqui fish. However, as a practical to the industry sector, it is

recommended that the final products are further evaluated for any pathogen of economic or public health importance.

Keywords: Brining; drying; nutrient profiles; tambaqui; reactive lysine.

Introduction

Fish meat is a high-value, perishable protein-rich food requiring appropriate preservation to maintain its organoleptic properties after harvest [1,2]. Nutritionally, fish meat is a prominent source of high quality essential amino acids [3,4], omega-3 fatty acids such as eicosapentaenoic acid (EPA) and (docosahexaenoic acid (DHA), and trace elements such as iron, selenium, iodine that are beneficial for the human health [5]. Depending on the species of the fish, culture system (farmed versus wild), feeding behavior (herbivores, carnivores, omnivores), and agroecology (temperate versus tropical; freshwater versus marine water) these essential nutrients, and their bioavailability, vary from one to other fish. Hence, a knowledge on the nutrient profiles of fish meat do promote a balanced and healthy diet for the human consumer and endorses consumer preferences on dietary requirements [5,6].

Tambaqui fish (*Colossoma macropomum*), also known as black pacu, is the main native fish species farmed in South and Central American countries [7]. It is mainly found in Brazil, Venezuela, Colombia, Peru, and Bolivia. The species alone accounted for a total production of 142,135 tons in 2016 with Brazil accounting for about 96.4% of the total production [8]. Its life cycle is mainly associated with floody plains of white-water rivers in the amazon basins that are characterized with a temperature range of 25-34°C, low to neutral pH (6-7), and rich in suspended solids [9,10].

Tambaqui is an omnivorous fish that feeds on a variety of natural feeds such as fruits and seeds, and zooplanktons depending upon seasons [8,11]. It is considered to be a nocturnal feeder fish [7]. The higher growth performance, hardiness, and socioeconomic value of tambaqui fish have nowadays led to technology endorsement in its farm level production [9,12,13].

Nutritionally, the tambaqui is also rich in essential amino acids, EPA and DHA, selenium, and vitamins D and B [9]. Furthermore, tambaqui requires a fairly low protein content in its feed, most of which (75-85%) can be provided as plant protein [7,12]. This makes it possible to breed tambaqui with the aid of low-cost feed and feedstuffs, such as forest fruits or seeds of leguminous plants [7].

Preservation is a fundamental requirement of food and food ingredients in maintaining a quality food which is a vital issue in the human health and contributes to profitability [14,15]. Fish meat, for instance, contains several set of nutrients (moisture, proteins, carbohydrates, and vitamins) that can easily get exposed to the ubiquitous environmental microbials (bacteria, moulds…) and chemicals causing degradation. The moisture content can be regarded as the primary nutrient input for fish meat degradation and affecting the nutritive value and shelf life of the product [6]. Generally, fish meat often have higher moisture content averaging 70-84% where bacteria can grow rapidly and cause spoilage [4]. The suppression or killing of such microorganisms can be achieved by either reducing the temperature (e.g. chilling, freezing) or reducing the water content (e.g. drying, salting) [15]. Drying of fish meat products can be achieved by traditionally sun-drying or open air drying and using air driers, or microwaves [5,17]. Salting, on the other hand, involve different processes such as brining (immersion of fish meat into salt solution), kench curing (dry salting of fish fillets), pickling (longer storage of the fish with brine), and the possible injection of brine into the fish muscle all ending up in reducing product water content and hence extended shelf life [5,18-21].

Brine salting is one of the oldest traditional and economically viable preservation methods of edible animal proteins such as fish meat [22-24]. It involves the use of salt (NaCl) that, due to its hygroscopic property, withdraws water from tissues via osmosis and, thereby, reduces water activity to slow bacterial growth [22]. In addition, salt is also reported to improve product

flavor, extract proteins, aid in emulsion formation, and reduce proteolytic activities of enzymes [25-27].

The quantity and concentration of salt and treatment time are determining factors in the process that could affect the nutritional quality of the fish [28-30]. A 15% brine concentration for a period of 48 h has been reported to yield optimum protein solubility/availability in a comparative study carried out in cod [24]. Likewise, better mass transfer kinetics of salt to tissues of cod and salmon were observed at 15% (*w/w*) brine concentration [23]. In fact, 10-15% (*w/w*) is the salt concentration recommended for salting whole fish under the food safety and standards regulation [31]. Higher concentrations of up to 25% (*w/w*) denature muscle proteins by causing cell membrane disruption, decreased enzymatic activity, or interaction with myofibrils, and reducing its water-holding capacity [23,24,32]. In general, higher yields of tissue proteins are obtained at lower salt concentrations [24,26,33]. In addition to the salt concentration, the purity of salt, salting method involved, the size, thickness and freshness of the fish, fish-to-salt ratio, and weather conditions are further factors of importance for the quality brine preserved fish [22,23].

Drying involves the exposure of a product to specified temperature and time period to facilitate loss of tissue water to a lower limit of water activity ($a_w < 0.90$) where microbial growth is inhibited [34]. Drying temperature, relative humidity and air velocity are determining the rate of dehydration [2]. In rural areas, temperature-controlled ovens are usually not available, and open-air drying is the only option. Especially high environmental temperatures during drying may affect the nutritive value of fish, for instance because heat may damage amino acids or may increase the reaction of amino acids with lipids and/or carbohydrates to form biologically unavailable Maillard products [35]. To determine the risk of drying at uncontrolled temperature for the nutritive value of fish, it is warranted to study the effect of a wide enough temperature range for fish drying.

Depending on the type of drying oven and species and size of the fish involved, a temperature range of 65 - 135°C and drying time of 2 - 20 h have been routinely used for drying of fish meat [2,26]. However, due to possible conjoint loss of some non-moisture compounds (e.g. volatile fatty acids and vitamins), lower temperatures with extended drying time are often recommended [36]. For example, drying of salted shark at 35°C reported to yield improved protein solubility, tissue rehydration and water retention capacity and total protein estimates compared to 45, 55 and 60°C [37]. Pacheco [38] also stated that the maximum drying temperature for the majority of the edible fish meat should not exceed 70°C. In fact, drying of fish products at higher temperatures, of more than 60°C, reported to cause fat oxidation and caramelization of sugars [37].

Despite the effect of brining and drying temperatures on the nutrient profiles of different fish species has been investigated, we hypothesized that species differences in nutrient profiles could respond differently to drying and brining and evaluated tambaqui fish. Our study also investigated reactive lysine as a marker for Maillard product formation: lysine is a limiting amino acid in most animal diets and need to be bioavailable to be used in metabolism. Despite its high concentration in tissue deposition, some reducing sugars, fats and their oxidation products and vitamins can bind the ε-amino group of lysine and generate Maillard reaction products during fish processing [35]. Hence, measuring the content of "reactive lysine" as a percentage of total lysine in tambaqui fish can indicate state of protein damage during processing. The present study therefore evaluated the effect of brining and a range of drying temperatures on the macronutrients and micronutrients, and mineral composition as well as on the state of Maillard reaction that may reduce lysine availability.

Materials and methods

Experimental animal ethics and welfare

All experimental and animal care procedures were realized based upon the current written knowledge of aquaculture technologies and tambaquí fish farming. Fingerlings of *Colossoma macropomum* were initially brought from Aqua-Koi NV farm (http://www.aqua-koi.be/documents/home.xml?lang=nl) to TRANSfarm and reared in a recirculating aquaculture system (RAS) with a weekly basis 30% water renewal (20L/day) for about 7 months period before being subject to the current study. During their stay, fish were kept in larger aqua-tanks (100 cm × 120 cm × 40 cm) at a density of 1.2 fish/m^3 sufficient enough for movements and behavioral expression. Tanks were fixed with individual bio-filters, well aerated with a constant temperature supply, 18:6 h light - dark cycle, and supplying pipes were regularly inspected and cleaned with tap water and water quality parameters were checked twice a week and water renewed accordingly [39].

For our experimental study, after a 12 h fasting, a total of 48 tambaqui fish were randomly selected and kept in a separate tank and were euthanized in a two-step procedure [40]. Six fish were randomly taken at a time and placed in a tank with a volume of 240 L water into which 50 mL of phenoxyethanol-solution (Anest Fish©, Germany) was added. After five minutes, 30 L of water were elevated out of the tanks and put into a large bucket. Another 6 mL of Anest Fish© was added to this bucket. Each group of six unconscious fish was scooped out of the tank and brought to the slaughter room in the bucket with the anesthetic agent (1mL:5L, Anest Fish©, Germany). Thereafter, tambaqui were killed with a firm blow to the head, followed by fracturing the brainstem with a knife and then transported in ice cubes for the experiment [40]. The study was conducted in accordance with the Declaration of Helsinki and directive 2010/63/EU of the European parliament and of the council on the protection of animals used for scientific purposes, and the wellbeing of the cultured tambaqui fish before being subjected to the experiment was assessed and approved by the institutional review board of TRANSfarm, KU Leuven, lab accreditation (KU/LA-1210616).

Study materials

A total of 48 tambaqui fish with a mean weight of 792 (± 16) g and 8 months old were collected from TRANSfarm, Lovenjoel, Belgium, and transported to the animal nutrition laboratory of the Department of veterinary and biosciences, Ghent University in styrofoam boxes with ice packs (Luxaplast, NORDIC®). Upon arrival, to facilitate drying whole fish were dissected dorso-ventrally into two equal slices using a stainless post mortem saw (SC-PM/110, 254MM) and scalpel-blades, thoroughly washed using distilled water in pipes and then individually stored in plastic bags at -20°C for 48 h. All dissected fish slices had an average uniform dimension of 21.2 ± 2.8 cm long and 12.6 ± 2.0 cm wide and average weight of 389 ± 11.5 g. Industrial table salt (99.9%, BDH-PROLABO®, EC) was used for preparing the 15% (*w/w*) brine solution in the present study [24].

Experimental design and set-up

Uniformly sliced whole fish samples were grouped into two as brine-salted and non-salted, and treated at seven different drying temperatures of 30, 35, 40, 45, 50, 55 and 60°C for a period of 23 h each [23,24,31,37]. Each temperature treatment was considered as a sampling point and tested in triplicate. Similarly, as a reference point, fresh whole-fish samples were initially evaluated in triplicate for their nutritional profiles.

Brine salting and drying processes

Brine-salted fish samples were immersed in brine solution (15% *w/w*) at a ratio of 3:1 of fish to salt solution [22,27]. For each sampling unit, samples were totally immersed and brined together in a closable plastic bucket (20 L) for 48 h at 4°C. The brine solution was pre-cooled to 4°C prior to the immersion of samples. Afterwards, brined samples were drained from salt drips for 3 min using a plastic sieve and randomly distributed into individual aluminium trays (500 mL; 142 mm × 116 mm × 30.4 mm; Boni™, Halle, Belgium), at one fish per try, into

which small beads (13 mm diameter, 3.5 g, JVLAB®) were added (10 beads/tray) at the bottom to avoid crusting of samples and optimize moisture loss. Likewise, the non-salted group of fish samples were randomly distributed into identical aluminium trays containing similar beads. Each tray was placed in an electric oven (EP66, BEKSO-SA®, Belgium) and randomly set and dried with the above-mentioned temperature points. The laboratory humidity was in a range of 50-60% during the experimental period. Drying temperature between sampling units were controlled using an oven-fixed thermometer and a consistent oven temperature (± 1°C) was maintained throughout the drying time period. A constant air ventilation was supplied through an oven-fixed ventilator at a steady air velocity of 1.0 m/s. Drying time started after oven closure and the oven returned to its set temperature (~ 5 min). After 23 h, samples were collected and allowed to cool in a desiccator for 5 min to room temperature before weighing. Samples were then kept at -21°C overnight and finally freeze-dried (CoolSafe™, SCANVAC, Denmark) for a period of 16 h each to a stable weight, uniformly ground (2.0 mm sieve), and homogenized using a grinder machine (De´Longhi™, KG48) at 12× speed and then individually kept in closable plastic containers at 4°C until analyzed [22].

Nutrient evaluations

Proximate analysis

So as to make legitimate comparisons between the processed samples, the quantitative analysis of the values of macromolecules is a significant nutritional marker and for which case the proximate analysis was used to determine the crude protein (CP), ether extract and ash contents of fish samples. The dry matter (DM) content of freeze-dried samples (1 g) was determined through oven-drying at 105°C for 24 h to constant weight [40]. Ether-extract was determined using the acid hydrolysis method [41], CP using the Kjeldahl method involving a nitrogen analyzer (LECO FP528 Instruments, St. Joseph, Michigan, USA) and N × 6.25 conversion factor [42], and ash content using an automated muffle furnace (Nabertherm™, UK)

as per the procedures described by AOAC [43]. A gram of fish sample was used for the analysis of each of the macronutrients and all samples were evaluated in duplicate at the animal nutrition laboratory of the Department of Veterinary and Biosciences, Ghent University, Belgium.

Mineral analysis

The total ash content was determined following the ISO method [44] and mineral (calcium, sodium, potassium, copper, iron, magnesium, manganese, phosphorus and zinc) concentrations of fish were evaluated after dry ashing mineralization according to ISO method (ICP-AES: ISO 11885) as per the procedures described by Gorsuch [45]. In brief, a gram of sample was weighed, placed into a porcelain crucible and oven-dried at 105°C for 3 h. Sample was then burnt on a hot plate and incinerated in a muffle furnace at 450°C for 16 h. Obtained ash was then moistened with a small amount of deionized water first and dissolved in 10 mL of 1:1 (*v/v*) solution of hydrochloric acid (HCl) and deionized water and evaporated to dry. Finally, the mix was re-dissolved in 10 mL of 1:9 (*v/v*) solution of HCl and deionized water, transferred into a 50 mL flask, and then diluted several folds for optimal determination of individual mineral concentration using induction-coupled plasma optical emission spectrometry (ICP-OES) method as per the manufacturer's procedures (Iris Intrepid II XSP, Thermo Fisher Scientific, Aalst, Belgium). So as to obtain an optimum concentration range for the atomic absorption spectrometric method, the concentrations of certain minerals were determined after fivefold (Mg and Ca), 10-fold (Na and Zn), and 50-fold (K) dilutions. Likewise, the concentration of selenium was determined by ISO method (ICP-MS: ISO 11885) and iodine with tetramethylammonium hydroxide extraction and iodine quantification (ICP-MS: ISO 11885) at the Laboratory of Chemical analysis, Ghent University, Belgium. As quality control protocols, visual inspection of pump tubing at regular intervals and replacing between samples are often made to increase the precision measurements. Similarly, regular inspection and cleaning of the injector tube, spread chamber and mobilizer, and cleaning of the widows are

done to avoid contamination and reduction of light intensity. Blanc solutions with similar matrix were used to improve the measurement accuracy and the detection limit set was 1-100 ppb.

Amino acid and OMIU-reactive lysine analyses

The total amino acid profiling was done by acid hydrolysis (2 g) at 110°C for 23 h and ion-exchange chromatography with post-column derivatization with ninhydrin method (ISO 13903). Sulphur containing amino acids were measured as cysteic acid and methionine sulfone after oxidation with performic acid; tryptophan was measured by alkaline hydrolysis at 110°C for 20 h and ion-exchange chromatography with fluorescence detection method (ISO 13904) according to procedures described by Hulshof [46].

For analysis of reactive lysine, about 2 g of fish sample were processed from each of the seven sampling points for defatting by extraction with light petroleum ether without acid hydrolysis (ISO 6492), finely ground and homogenized (2.0 mm sieve size) using a mixer mill (Retsch MM200, BV) and used for the analysis of reactive lysine via O-methylisourea (OMIU) method according to Hulshof [46]. Briefly, 1 mL of 0.6 M solution of OMIU was prepared as described by Moughan and Rutherfurd [47] that converts lysine with free e-amino group to homoarginine. Four gram of barium hydroxide octahydrate (Sigma-Aldrich, Zwijndrecht, the Netherlands) was added to 16 mL of boiling (10 min) distilled deionized water to remove CO_2, after which 2 g of OMIU sulphate salt was added. The solution was then cooled for 30 min at room temperature, centrifuged (6,400 × g for 10 min at 20°C) and the supernatant was retained and the precipitate was washed with about 2 mL of same water and further centrifuged (6,400 × g for 10 min at 20°C). both supernatants were combined and pH adjusted to 11.5 (for guanidation) by adding 6 M HCl solution. The homoarginine content was analysed in duplicate and the OMIU-reactive lysine was calculated from the homoarginine content. All amino acid profile, including reactive lysine, were determined at Wageningen University & Research, Animal nutrition laboratory, The Netherlands.

Statistical analysis

Data obtained in the current study were analyzed using SPSS statistical software version 27 (IBM Corporation, Armonk, NY, USA). All data were checked for normality distributions using histogram and QQ-plots. A univariate one-way ANOVA was used with brining as fixed factor and temperature as covariate to test the hypothesis about differences between two or more mean nutrient values. Data were presented as means and standard error of the means (SEM). Statistical significance was set at $P < 0.05$ for any differences observed between samples. Furthermore, principal component analysis (PCA) was computed using on the collective factors obtained to appraise the relationships of nutrient profiles based on their responses towards brining and drying methods, with values deemed relevant below -0.5 and above 0.5 of the components' values.

Results

Tambaqui fish samples were grouped into two as brine-salted and non-salted, and treated at seven different drying temperatures for 23 h each. Each temperature treatment was considered as sampling point and tested in triplicate (n = 3) and mean values obtained were statistically computed for all nutrient profiles evaluated.

Proximate analysis

Drying temperature had no significant effect on the measured nutrient profiles of the fish in this study and all data were combined across temperature treatments ($P > 0.05$; Fig 1). However, several effects ($P < 0.05$) were observed with the brining method.

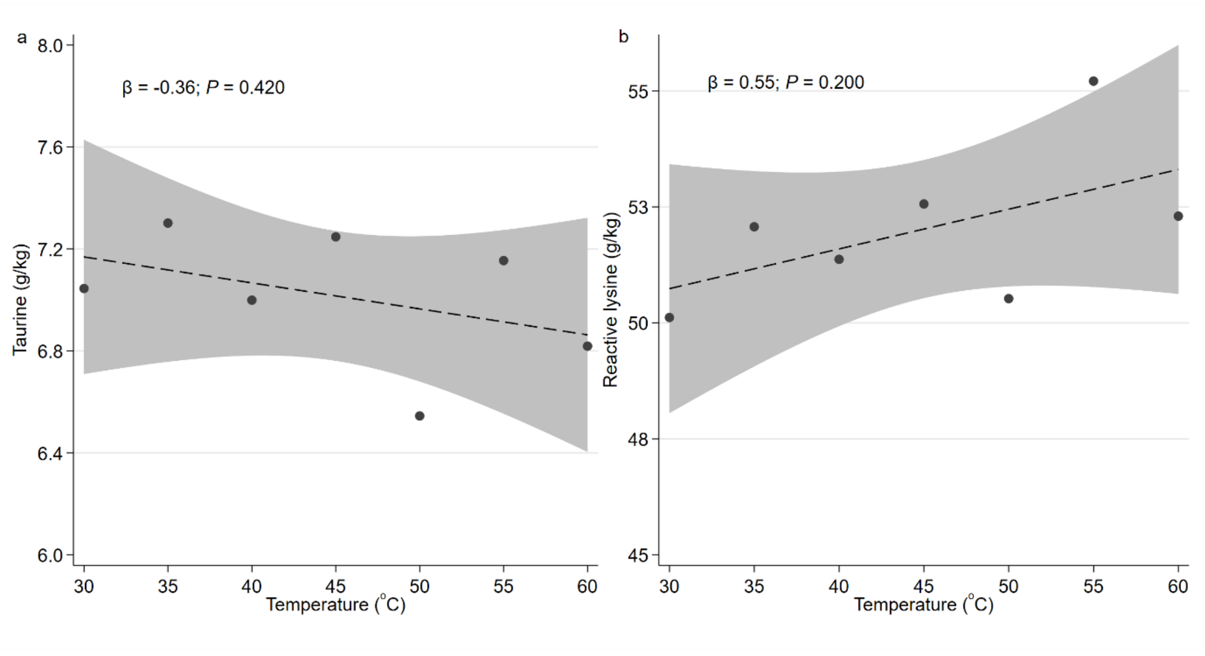

Fig 1. The effect of drying temperature on nutrient profiles of tambaqui fish. (a) gram of taurine per kg fish; (b) gram of reactive lysine per kg fish.

Brining resulted in a higher ash concentration with a relative increase of 28% ($P < 0.05$). Moreover, 6% loss on the overall protein concentration was noticed due to brining ($P < 0.05$). The body fat contents of tambaqui fish were not affected by the addition of salt and temperature treatments ($P > 0.05$; Table 1).

Table 1. Changes in macronutrient concentrations in tambaqui fish (on %DM basis) due to brining and drying at seven different temperatures (30, 35, 40, 45, 50, 55 and 60°C).

Concentration (g/100 g DM)	Not salted	Salted	Total	Relative % Change	SEM	*p*	
						salted	temp
**Ash**	12.4	16.0	14.1	28	0.5	< 0.001	0.793
**Crude protein**	58.8	55.4	57.0	-6	0.6	0.004	0.846
**Crude fat**	28.3	28.7	28.7	1	0.4	0.716	0.657

DM= Dry matter; SEM= standard error of the means; temp= temperature.
Values represent mean and standard error of the means based on three replicates per treatment and seven treatments; n = 48.

Minerals and amino acids

The essential minerals Se and I, and the macrominerals K and Mg were significantly reduced by brining with 14, 38, 42 and 18% percent losses, respectively (*$P < 0.05$*). Moreover, brining resulted in an obvious enormous significant increase (744%) in Na concentration (*$P < 0.05$*). Otherwise, all minerals (Fe, Mn, P and Ca) were decreased by brining (Table 2).

Table 2. Changes in mineral concentrations in tambaqui fish due to brining and drying at seven different temperatures (30, 35, 40, 45, 50, 55 and 60°C).

**Minerals**	**Not salted**	**Salted**	**Total**	**Relative % Change**	**SEM**	***p***	
						**salted**	**temp**
**Macrominerals (g/kg DM)**							
**Ca**	37.4	37.1	37.0	-1	1.1	0.965	0.965
**K**	9.71	5.61	7.71	-42	0.6	<0.001	0.495
**Mg**	1.32	1.10	1.10	-18	0.03	<0.001	0.880
**Na**	2.70	22.9	12.1	744	2.7	<0.001	0.564
**P**	21.7	20.4	21.0	-6	0.6	0.317	0.814
**Microminerals (mg/kg DM)**							
**Fe**	42.0	36.9	39.2	-12	2.4	0.256	0.188
**I***	604	373	489	-38	49	0.020	0.264
**Mn**	9.80	8.81	9.31	-10	0.3	0.115	0.152
**Se***	149	128	137	-14	5	0.020	0.449
**Zn**	48.5	50.6	49.6	4	1.3	0.473	0.588

DM= Dry matter; SEM= standard error of the means; temp= temperature.
Values represent mean and standard error of the means based on three replicates per treatment and seven treatments; n = 48.
*Values are presented in µg/kg DM.

Brining negatively affected the overall sum amino acid profile (*$P < 0.05$*). In particular, taurine, histidine, cysteine, methionine and available (reactive) lysine were affected by brining with the highest percent loss accounted for taurine (56%) followed by methionine (17%), cyteine (11%), and histidine (10%) (*$P < 0.05$*). In contrast, there was numerical 5 and 6%

increase in proline and glycine respectively, whereas alanine and arginine were not affected by brining (*P > 0.05*; Table 3).

Table 3. Changes in amino acid concentrations in tambaqui fish due to brining and drying at seven different temperatures (30, 35, 40, 45, 50, 55 and 60°C).

Amino acids (g/kg CP)	Not salted	salted	Total	Relative % Change	SEM	*p*	
						salted	temp
Taurine	7.10	3.10	5.11	-56	0.5	<0.001	0.324
Asparagine + Aspartic acid	56.8	54.9	55.4	-3	0.9	0.268	0.671
Threonine	27.0	26.3	26.5	-3	0.4	0.311	0.965
Serine	25.0	24.9	24.8	0	0.2	0.807	0.777
Glutamine + Glutamic acid	91.4	88.3	89.2	-3	1.3	0.247	0.851
Glycine	75.7	80.2	79.3	6	2.5	0.361	0.750
Alanine	51.1	51.0	51.2	0	0.7	0.961	0.636
Valine	30.8	29.2	29.8	-5	0.5	0.062	0.642
Isoleucine	26.0	24.6	24.9	-5	0.5	0.162	0.838
Leucine	44.3	41.8	42.5	-6	0.9	0.134	0.855
Tyrosine	17.9	16.5	16.9	-8	0.5	0.098	0.952
Phenylalanine	24.5	23.4	23.8	-5	0.4	0.134	0.874
Histidine	15.5	14.0	14.6	-10	0.4	0.054	0.400
Lysine	51.1	47.4	48.6	-7	1.1	0.070	0.863
Arginine	48.3	48.3	48.5	0	0.5	0.999	0.464
Proline	47.5	49.9	49.5	5	1.3	0.356	0.660
Cysteine	5.10	4.40	4.60	-11	0.1	0.016	0.746
Methionine	14.7	12.1	13.3	-17	0.4	<0.001	0.675
Tryptophan	5.10	4.60	4.71	-8	0.2	0.229	0.698
Reactive lysine	52.0	46.4	48.5	-11	1.2	0.004	0.851
% Reactive lysine	102.2	97.9	99.9	-4	1.8	0.306	0.760
Sum AA	665	645	653	-3	6.87	0.017	0.621

CP= Crude protein; AA= Amino acid; SEM= Standard error of the means; temp= temperature.
Values represent mean and standard error of the means based on three replicates per treatment and seven treatments; n = 48.
[a]Percentage of reactive lysine in the total protein.
[b]Percentage of reactive lysine in the total lysine.

Multivariate classification of variables

Principal component analysis was computed on the collective factors obtained to appraise the relationships of nutrient profiles based on their responses towards brining and drying methods. By examining at the scree plot and eigenvalues and looking for a point at which the proportion of variance explained by each subsequent principal component drops off, an elbow in the screen plot, the most explaining two components 1 (44.8%) and 2 (18.9%) were considered and a biplot PCA generated.

In line, there were no associations between nutrient profiles and drying temperature while several associations were observed with brining. Negative association was seen between brining, Na and ash on one side of component 1 and most other nutrients on the other. However, positive association between the brining proxies (Na, ash) and amino acids prominent in connective tissue (glycine, proline, arginine, alanine) were typically seen clustered together right of the component. The more central position of Zn, Fe, fat, and available Lys point to their low response to brining and temperature. More importantly, temperature itself was positioned centrally in the plot, emphasizing the absence of drying temperature effects on the study parameters (Fig 2).

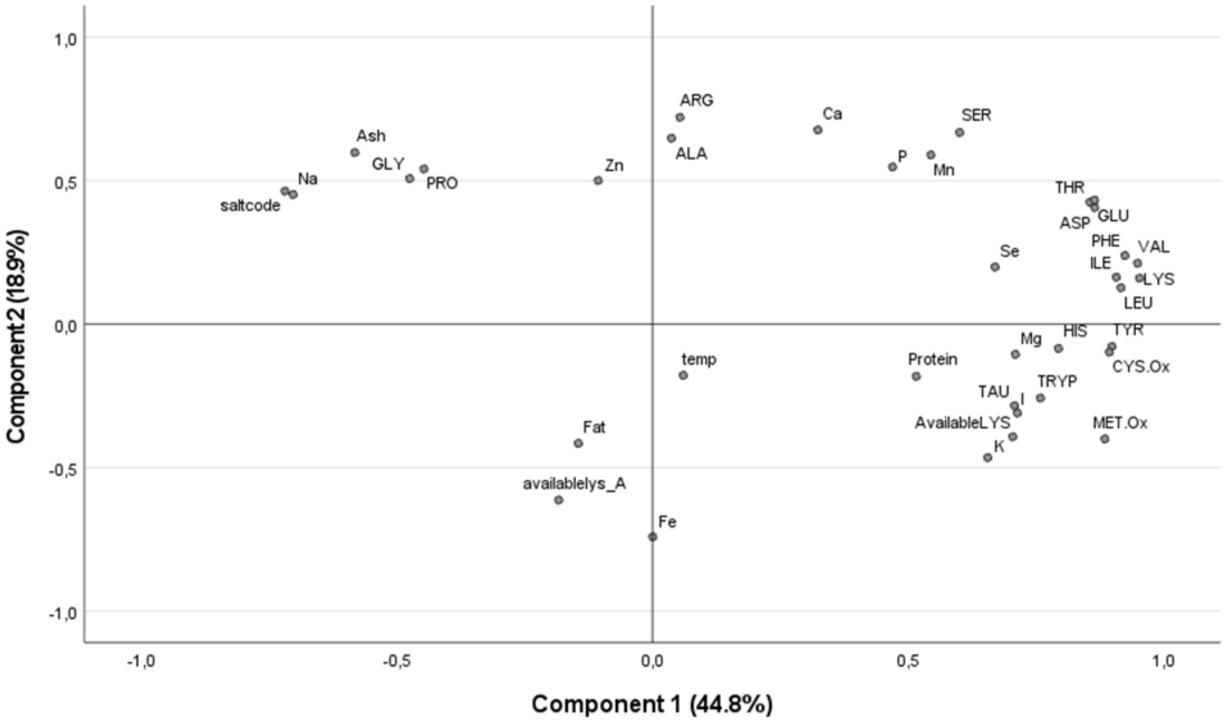

Fig 2. Principal component analysis classification of nutrient profiles based on their response to brining and temperature. Analyses represent principal component analysis (PCA) of evaluated nutrient values (expressed as percentages of the sum of total) after brining and drying of whole tambaqui fish samples (n = 48). AvailableLYS, is the percentage of reactive lysine in the total protein; availablelys_A, is the percentage of reactive lysine in the total lysine; saltcode, refers to brining; temp, is the drying temperature.

Discussion

The present study evaluated the effect of brine salting (15% *w/w*) prior to drying at different temperatures on the nutrient profiles of tambaqui fish. Brining induced a prominent effect on macronutrient and micronutrient profiles of tambaqui fish.

In this study, brining induced pronounced losses of specific amino acids and minerals, irrespective of drying temperature. Gill et al. [48] found that salting enhanced the aggregation of myosin in cod, yet only above a drying temperature of 50°C. Marínez-Alvarez and Gómez-Guillén [33] indicated that there is a notable loss of soluble muscle proteins (particularly, actin and myosin) by osmosis in salting (NaCl) at pH 6.5 on cod muscle (*Gadus morhua*). Differences between studies can evolve from a wide range of conditions used in the drying process including

the drying type, drying temperature and duration, relative humidity, air velocity and the specific characteristics of the product dried (species and size of the fish). Although comparison with existing literature does not allow us to pinpoint the factors that protected from a drying temperature effect, this study suggests that brining is associated with nutrient losses. In the present study, a total of 6% crude protein was lost due to the brining stress. In fact, salt concentration and salting duration can modulate the effect of brining on muscular protein loss in fish [28-30]. The solubility of muscle proteins often start to decrease with increasing salt concentration due to protein aggregation [24] and/or altered enzymatic activities [49]. In the present study, however, an acceptable (15% *w/w*) salt was used for brining [31].

The amino acid analysis in this study demonstrated that the salt-induced protein loss was not an overall effect, but reduced specific amino acids, taurine, histidine, cysteine, methionine and available lysine. Taurine is not involved in protein synthesis but often accumulates in muscle tissues of fish and has osmolytic properties [50,51]. Hence, the loss in taurine seem a logical reaction to the osmotic stress from brining. This taurine loss can be important since its role in health has been demonstrated for instance in infants [52] and it is also the main amino acid to form conjugated bile acids in chickens [53].

The tendency to a lower histidine concentration due to brining can be seen in the same light. Apart from its protein-bound appearance, histidine is stored in muscular tissue as either free histidine (acetylated or not) or bound to mainly beta-alanine (hence constituting dipeptides such as carnosine or anserine); this storage of histidine occurs throughout the animal kingdom [54]. Shiau [55] reported that histidine can easily get released from muscle tissues during stress conditions such as starvation. Significant losses of the free amino acids histidine, taurine and glycine were reported in salted milkfish [32]. Histidine is involved in the regulation and metabolism of essential trace minerals, formation of enzymes, induction of the immune system

through the production of histamine and protects brain cells through the formation of metallothionein among others [56].

Cysteine and methionine are sulphur-containing amino acids and quite reactive to processing, in that they could either get directly affected by the ionic brine solution or can possibly make cross-links that reduces their tissue availability [32]. Kumazawa [57] reported that brining may cause cross-linking reactions of muscle myosin heavy chains and form ε-(γ-glutamyl)lysine through transglutaminase. There was no effect on the percentage of reactive lysine (% available-lysine), meaning that both salting and drying did not induce Maillard reactions that affect lysine availability. The relative decrease in the total lysine likely explains the decreased percentage of available lysine in the current study.

Four specific amino acids in the principal component analysis separated from the negative association between brining and the other amino acids: alanine and arginine were not affected while glycine and proline were even positively associated with brining. These amino acids are prominent in the skeleton and in scales, which is a strong indication that the highest protein losses were from muscle and other soft tissues [58]. Other authors not always report similar changes in amino acid pattern: a sigmoid initial increase (up to 9 h) and final slight decrease (48 h) of alanine, glutamine and lysine was reported in salted milkfish [32]; alanine, glycine, lysine and histidine increased when salted fish was dried [59]. The increase in these amino acid concentrations could be due to the decrease in the proportion of the extracted total lipids present as polyunsaturated fatty acids as depicted by Yankah [59]. Fat is a hydrophobic molecule and can act as a physical barrier to limit salt (NaCl) and water transport across the muscle tissue [23]. In fact, the lipid content was higher for the fish in our study (28.71%) although fat concentration was not affected by both treatments.

The specific loss of soft tissue from the fish is not only supported by the amino acid data, but also witnessed by the change in mineral profile. The considerable loss of the microminerals

Se and I and the macrominerals K and Mg following brining reflects the presence of these minerals in soft tissue [60]. Selenium typically occurs as replacement of sulphur in sulphur-containing amino acids (cysteine and methionine) in soft tissues. The decrease in methionine and cysteine seen in our study, therefore, fits the observation of decreased selenium. In contrast, the typical skeletal minerals such as Ca, P and Mg, and those that are deposited in scales and fins such as Zn and Fe, were not affected. Potassium is a strong electrolyte that could disappear in the brine solution or get filtered out in exchange with the added sodium. The loss of water due to salt was explained as the main cause for leaching of these macrominerals in the solution during brining. The decrease in K concentration agrees with Polak-Juszczak [61] and Bakhiet [62] from salted fish. The latter study remarkably reported a salt-induced increase in iodine concentration, most likely evolving from salt impurities as proposed by the authors. It is not clear how these differences with our study can be explained, but we used a purified source of salt, hence excluding potential unintended supply of microminerals to the fish tissues through brining.

Drying is an energy intensive process which can use about 10% or more of the total energy consumed during processing. In the present study, although our aim was not to quantify the drying efficiency, we conjointly were aiming to consume less energy while drying samples by increasing the sample surface area of exposure, avoiding surface contact by putting beads, improved equipment insulation during and between drying, supply of a constant air ventilation at 1.0 m/s during drying, and minimizing the timing between dryings [63].

Undeniably, brining has substantially increased Na and ash concentrations. This has been observed in most studies on brining of fish [61,62]. This enrichment with salt through brining must be considered when formulating diets but, more importantly, our results demonstrate that brining may not be the best choice to maintain the nutritive value of fish. Evidently, we must acknowledge that nutritive value is not the only criterion to pick a preservation method for fish:

for the reduction of the pathogenic load in fish products, salt addition may still be a valuable technique that goes, however, at the expense of nutritive value as shown in our study. A comforting finding is that the wide range in drying temperature evaluated in our study had no noticeable impact on the nutritive value of the fish. This implies that drying of fish under uncontrolled environmental temperature seems a suitable option in rural areas to preserve the nutritive value of (tambaqui) fish.

5. Conclusions

This research shows that drying temperature has no effect on nutrient composition of the studied fish which could be encouraging for drying of fish under – less controlled – rural tropical conditions. Brining, however, reduced some important trace elements such as Se and I, and lead to loss of muscle crude protein, with the concomitant amino acids. Brining also increased the leaching of K, I, and taurine. The formation of Maillard products was not promoted by both drying and brining treatments, as demonstrated through unaltered reactive lysine proportions. Therefore, drying without brining may better preserve the nutritive value of tambaqui fish. However, as a recommendation to the industry sector, it is suggested that the final products are further evaluated for any pathogen of economic or public health importance.

Acknowledgments

The authors acknowledge Herman De Rycke for the proximate analysis.

References

1. Pawar SM, Sonawane SR. Fish muscle protein highest source of energy. Int J Biodivers Conserv. 2013; 5:433–435. doi: 10.5897/IJBC12.043.
2. di Domenico CNB. Fish drying: A review. Rev Bras Eng Pesca. 2014; 7:1–18. doi: 10.18817/repesca.v7i1.1061.

3. Marta M, Francisca H, Robert S. Fatty acids profile in canned tuna and sardine after retort sterilization and high pressure thermal sterilization treatment. J Food Nutr Res. 2015; 54:171–178. doi: hdl.handle.net/10261/114599.

4. Abraha B, Admassu H, Mahmud A, Tsighe N, Shui XW, Fang Y. Effect of processing methods on nutritional and physico-chemical composition of fish: a review. MOJ Food Process Technol. 2018; 6:376-82. doi: 10.15406/mojfpt.2018.06.00191.

5. Sampels S. The effects of processing technologies and preparation on the final quality of fish products. Trends Food Sci Technol. 2015; 44:131-146. doi: 10.1016/j.tifs.2015.04.003.

6. Kumar MS. Sustainable management of fisheries and aquaculture for food security and nutrition: policies requirements and actions. Agric Res. 2014; 3:97-103.

7. da Silva JAM, Pereira-Filho M, Oliveira-Pereira MI. Seasonal variation of nutrients and energy in tambaquí's (*Colossoma macropomum* Cuvier, 1818) natural food. Braz J Biol. 2000; 60:4. doi: 10.1590/s0034-71082000000400009

8. Woynárovich A, van Anrooy R. Field guide to the culture of tambaquí Colossoma macropomum, Cuvier, 1816. In: FAO Fisheries and Aquaculture Technical Paper. 2019; 624:21-30.

9. Arantes ML, Freitas CEC. Effects of fisheries zoning and environmental characteristics on population parameters of the tambaqui (*Colossoma macropomum*) in managed floodplain lakes in the Central Amazon. Fish Manag Ecol. 2016; 23:133–143. doi: 10.1111/fme.12164

10. Val AL, de Oliveira AM. *Colossoma macropomum*—A tropical fish model for biology and aquaculture. J Exp Zoo Part A: Ecol Integ Phys. 2021; 335:761-70. doi: 10.1002/jez.2536

11. Barat, J.M., Rodríguez-Barona, S., Andrés, A., & Fito, P. (2002). Influence of increasing brine concentration in the cod salting process. J Food Sci. 65(7):1922–1925. doi: 10.1111/j.1365-2621.2002.tb08747.x.

12. Gomes LC, Silva CR. Impact of pond management on tambaquí, *Colossoma macropomum* (Cuvier), production during growth-out phase. Aqua Res. 2009; 40:825–832. doi: 10.1111/j.1365-2109.2009.02170.x

13. Sandre LCG, Buzollo H, Nascimento TMT, Neira LM, Jomori RK, Carneiro DJ. Productive performance and digestibility in the initial growth phase of tambaquí (*Colossoma macropomum*) fed diets with different carbohydrate and lipid levels. Aqua Rep. 2017; 6:28–34. doi: 10.1016/j.aqrep.2017.02.003

14. Heijnen C, Boogaard BVD, Maas van Berkel B. Preservation of fish and meat. Agrodok. 2004; Pp. 26-35.

15. Miladi H, Chaieb K, Bakhrouf A, Elmnasser A, Ammar E. Freezing effects on survival of Listeria monocytogenes in artificially contaminated cold fresh-salmon. Annals Microbiol. 2008; 58:471-476.

16. Mahmoud BSM, Yamazaki K, Miyashita K, Shin II, Suzuki T. A new technology of fish preservation by combined treatment with electrolysed NaCl solutions and essential oil compounds. Food Chem. 2006; 99:656-662. doi: 10.1016/j.foodchem.2005.08.037.

17. Djendoubi N, Boudhrioua N, Bonazzi C, Kechaou N. Drying of sardine muscles: Experimental and mathematical investigations. Food Bioprod Process. 2009; 87:115-123. doi: 10.1016/j.fbp.2008.07.003.

18. Birkeland S, Skåra T, Bjerkeng B, Rørå AMB. Product yield and gaping in cold-smoked Atlantic salmon (*Salmo salar*) fillets as influenced by different injection-salting techniques. J Food Sci. 2003; 68:1743-1748.

19. Guizani N, Rahman MS, Al-Ruzeiqi MH, Al-Sabahi JN, Sureshchandran S. Effects of brine concentration on lipid oxidation and fatty acids profile of hot smoked tuna (*Thunnus albacares*) stored at refrigerated temperature. J Food Sci Technol. 2014; 51:577-582. doi: 10.1007/s13197-011-0528-4.

20. Hall G. Preservation by curing (drying, salting and smoking). In: G. Hall (Ed.), Fish processing sustainability and new opportunities, West Sussex: Wiley-Blackwell. 2011; Pp. 51-76.

21. Kituu GM, Shitanda D, Silayo D, Odote P, Bongyereire J. Effect of brining on the drying rate of tilapia (*Oreochromis niloticus*) in a solar tunnel dryer. Fish Aqua Cluster Proceed. 2000; 88-98. http://ir.mksu.ac.ke/handle/123456780/4889.

22. Binici A, Kaya GK. Effect of brine and dry salting methods on the physicochemical and microbial quality of chub (*Squalius cephalus* Linnaeus, 1758). Food Sci Technol. 2018; 38:66–70. doi: 10.1590/1678-457X.15717.

23. Gallart-Jornet L, Barat JM, Rustad T, Erikson U, Escriche I, Fito P. A comparative study of brine salting of Atlantic cod (*Gadus morhua*) and Atlantic salmon (*Salmo salar*). J Food Eng. 2007; 79:261–270. doi: 10.1016/j.jfoodeng.2006.01.053.

24. Nguyen MV, Thorarinsdottir KA, Gudmundsdottir A, Thorkelsson G, Arason S. The effects of salt concentration on conformational changes in cod (*Gadus morhua*) proteins during brine salting. Food Chem. 2011; 125:1013–1019. doi: 10.1016/j.foodchem.2010.09.109.

25. Rust RE, Knipe CL. Sausage casings. In: Devine C, Dikeman M, editors. Encyclopedia of Meat Sciences. Academic Press: Oxford; 2014. pp. 235–240.

26. Thorarinsdottir KA, Arason S, Bogason S, Kristbergsson K. The effects of various salt concentrations during brine curing of cod (*Gadus morhua*). Int J Food Sci Technol. 2004; 39(1):79–89. doi: 10.1046/j.0950-5423.2003.00757.x.

27. Thorarinsdottir KA, Arason S, Sigurgisladottir S, Valsdottir T, Tornberg E. Effects of different pre-salting methods on protein aggregation during heavy salting of cod fillets. Food Chem. 2011; 124(1):7–14. doi: 10.1016/j.foodchem.2010.05.095.

28. Barat JM, Rodríguez-Barona S, Andrés A, Fito P. Influence of increasing brine concentration in the cod salting process. J Food Sci. 2002; 67(5):1922–1925. doi: 10.1111/j.1365-2621.2002.tb08747.x.

29. Larsen R, Elvevoll EO. Water uptake, drip losses and retention of free amino acids and minerals in cod (*Gadus morhua*) fillet immersed in NaCl or KCl. Food Chem. 2008; 107(1):369–376. doi: 10.1016/j.foodchem.2007.08.031.

30. Thorarinsdottir KA, Arason S, Geirsdottir M, Bogason SG, Kristbergsson K. Changes in myofibrillar proteins during processing of salted cod (*Gadus morhua*) as determined by electrophoresis and differential scanning calorimetry. Food Chem. 2002; 77(3):377–385. doi: 10.1016/S0308-8146(01)00349-1.

31. FSSA. Fish and fish products regulations. In: Gaur VN, editors. Food Safety and Standards Regulation Authority (FSSA). New Delhi: The gazette of India, 2011. pp. 366-369.

32. Sannaveerappa T, Ammu K, Joseph J. Protein-related changes during salting of milkfish (*Chanos chanos*). J Sci Food Agric. 2004; 84(8):863–869. doi: 10.1002/jsfa.1682.

33. Marínez-Alvarez O, Gómez-Guillén MC. The effect of brine composition and pH on the yield and nature of water-soluble proteins extractable from brined muscle of cod (*Gadus morhua*). Food Chem. 2005; 92(1):71–77. doi: 10.1016/j.foodchem.2004.04.049.

34. Ockerman HW, Basu L. Preservation methods of animal products. In: Dikeman M, Devine C, editors. Encyclopedia of Meat Sciences. London: Oxford Elsevier Ltd.; 2014. pp. 78–83. doi: 10.1016/B978-0-12-384731-7.00207-5.

35. Kim JC, Mullan BP, Pluske JR. Prediction of apparent, standardized, and true ileal digestible total and reactive lysine contents in heat-damaged soybean meal samples. J Anim Sci. 2012; 90:137–139. doi: 10.2527/jas.53819.

36. Lantry BF, O'Gorman R. Drying temperature effects on fish dry mass measurements. J Great Lakes Res. 2004; 33:606–616. doi: 10.3394/0380-1330(2007)33[606:DTEOFD]2.0.CO;2.

37. Bastos JR. Influência da secagem sobre algumas propriedades físico-químicas do músculo do cação branco, Carcharhynus porosus Ranzani (in portuguess, with english abstract). Arquivos de Ciências do Mar. 1997; 17:77–87. Available from: http://www.repositorio.ufc.br/handle/riufc/1403

38. Pacheco N, Garnica-Gonzalez M, Gimeno M, Bárzana E, Trombotto S, David L, et al. Structural characterization of chitin and chitosan obtained by biological and chemical methods. Biomacromolecules. 2011; 12:3285–3290. doi: 10.1021/bm200750t.

39. Frisso RM, de Matos FT, Moro GV, de Mattos BO. Stocking density of Amazon fish (*Colossoma macropomum*) farmed in a continental neotropical reservoir with a net cages system. Aquaculture. 2020; 529:p.735702. doi: 10.1016/j.aquaculture.2020.735702

40. Lewbart GA. Euthanasia of ectotherms. In: Fowler's zoo and wild animal medicine current therapy. Elsevier. 2019; 9:357–363. doi: 10.1016/b978-0-323-55228-8.00051-5.

41. AOAC. Official Methods of Analysis of the Association of Official Analytical Chemists. 16th ed. Arlington VA; 1995.

42. AOAC. Official Methods of Analysis of the Association of Official Analytical Chemists. 17th ed. Washington DC: Gaithersburg publishing; 2001.

43. AOAC. Official Methods of Analysis of the Association of Official Analytical Chemists. 15th ed. Arlington VA; 1990.

44. ISO. Meat and Meat Products - Determination of total ash. In: International Organization for Standardization. Switzerland: ISO/936; 1998. Available from: https://www.iso.org/standard/24783.html

45. Gorsuch TT. The Destruction of organic matter. 1st ed. New York: Oxford pergamon press Ltd; 1970. pp. 40–54.

46. Hulshof TG, Bikker P, Van der Poel AFB, Hendriks WH. Assessment of protein quality of soybean meal and 00-rapeseed meal toasted in the presence of lignosulfonate by amino acid digestibility in growing pigs and Maillard reaction products. J Anim Sci. 2016; 94:1020–1030. doi: 10.2527/jas.2015-9700.

47. Moughan PJ, Gall MPJ, Rutherfurd SM. Absorption of lysine and deoxyketosyllysine in an early Maillard browned casein by the growing pig. J Agric Food Chem. 1996; 44:1520–1525. doi: 10.1021/jf950428v.

48. Gill TA, Chan TK, Phozchareon KF, Paulson AT. Effect of salt concentration and temperature on heat-induced aggregation and gelation of fish myosin. Food Res Int. 1992; 25:333–341. doi: 10.1016/0963-9969(92)90108-H.

49. Stoknes IS, Walde PM, Synnes M. Proteolytic activity in cod (*Gadus morhua*) muscle during salt curing. Food Res Int. 2005; 38:693–699. doi: 10.1016/j.foodres.2005.02.003.

50. Geda F, Declercq AM, Remø SC, Lourenço RWM, Janssens GPJ. The metabolic response in fish to mildly elevated water temperature relates to species-dependent muscular concentrations of imidazole compounds and free amino acids. J Therm Biol. 2017; 65:57-63. doi: 10.1016/j.jtherbio.2017.02.004.

51. Zhang Y, Dabrowski K, Hliwa P, Gomulka P. Indispensable amino acid concentrations decrease in tissues of stomachless fish, common carp in response to free amino acid- or peptide-based diets. Amino Acids. 2006; 31(2):165–172. doi: 10.1007/s00726-006-0345-7.

52. Chesney RW, Helms RA, Christensen M, Budreau AM, Han X, Sturman JA. The role of taurine in infant nutrition. Adv Exp Med Biol. 1998; 442:463–476. doi: 10.1007/978-1-4899-0117-0_56.

53. Alzawqari MH, Al-Baadani HH, Alhidary IB, Al-Owaimer AN, Abudabos AM. Effect of taurine and bile acid supplementation and their interaction on performance, serum components, ileal viscosity and carcass characteristics of broiler chickens. S Afr J Anim Sci. 2016; 46(4):448–457. doi: 10.4314/sajas.v46i4.13.

54. Boldyrev AA, Aldini G, Derave W. Physiology and pathophysiology of carnosine. Physiol Rev. 2013; 93:1803–1845. doi: 10.1152/physrev.00039.2012.

55. Shiau CY, Pong YJ, Chiou TK, Tin YY. Effect of starvation on free histidine and amino acids in white muscle of milkfish *Chanos chanos*. Comp Biochem Physiol B: Biochem Mol Biol. 2001; 128:501–506. doi: 10.1016/S1096-4959(00)00350-X.

56. Kopeć W, Jamroz D, Wiliczkiewicz A, Biazik E, Pudlo A, Hikawczuk T, et al. Influence of different histidine sources and zinc supplementation of broiler diets on dipeptide content and antioxidant status of blood and meat. Br Poult Sci. 2013; 54:454–465. doi: 10.1080/00071668.2013.793295.

57. Kumazawa K, Seguro K, Takamura M, Motoki M. Formation of ε-(γ-glutamyl)lysine cross-link in cured horse mackerel meat induced by drying. J Food Sci. 1993; 58:1062–1064. doi: 10.1111/j.1365-2621.1993.tb06112.x.

58. Fedina IS, Georgieva K, Grigorova I. Light-dark changes in proline content of barley leaves under salt stress. Biol Plant. 2002; 45:59–63. doi: 10.1023/A:1015175802847.

59. Yankah MV, Ohshima T, Koizumi C. Effects of processing and storage on some chemical characteristics and lipid composition of a Ghanian fermented fish product. J Sci Food Agric. 1993; 63:227–235. doi: 10.1002/jsfa.2740630209.

60. Yamashita Y, Yamashita M, Iida H. Selenium Content in Seafood in Japan. Nutrients. 2013; 5:388–395. doi: 10.3390/nu5020388.

61. Polak-Juszczak L. Effects of processing methods on the content of minerals in fish products. J Elem. 2016; 21:461-470. doi: 10.5601/jelem.2015.20.3.890.

62. Bakhiet HHA, Khogali FAH, Ahmed FAM. Effect of different salt concentrations on mineral content of the fish (*Hydrocynus* spp.). J Life Sci Biomed. 2013; 3(1):01–03.

63. Pask F, Lake P, Yang A, Tokos H, Sadhukhan J. Industrial oven improvement for energy reduction and enhanced process performance. Clean Technol Environ Policy. 2017; 19:215-224. doi: 10.1007/s10098-016-1206-z.

Click here to access/download
Supporting Information - Compressed/ZIP File Archive
Supporting information files.zip

Revised Manuscript with Track Changes

~~A laboratory simulation of the i~~Impact of brining and drying processes~~as accessible preservation strategies under tropical rural conditions~~ on the nutritive value of tambaqui fish (*Colossoma macropomum*)

Awot Teklu Mebratu[1,2*], Yohannes Tekle Asfaw[3], Wouter Merckx[4], Wouter H. Hendriks[5], Geert P.J. Janssens[1]

[1]Department of Veterinary and Biosciences, Faculty of Veterinary Medicine, Ghent University, Merelbeke, Belgium

[2]Department of Animal Reproduction and Welfare, College of Veterinary Sciences, Mekelle University, Mekelle, Ethiopia

[3]Department of Veterinary Basic and Diagnostic Sciences, College of Veterinary Sciences, Mekelle University, Mekelle, Ethiopia

[4]TRANSfarm, Engineering and technology group, The Catholic University of Leuven, Lovenjoel, Belgium

[5]Animal Nutrition Group, Wageningen University, Wageningen, The Netherlands

[*]Corresponding author

Email: awotteklu.mebratu@ugent.be (ATM)

Abstract

Preservation of fish as diet ingredient is challenging in many tropical conditions due to the absence of freezing facilities, hence warranting other preservation techniques. Brine salting is one of the oldest traditional and economically viable preservation methods. The present study evaluated the effect of brine salting (15% *w/w*) prior to drying at different temperatures on the nutrient profiles of tambaqui fish (*Colossoma macropomum*). Whole fish samples (792 ± 16 g; 8 months old; n = 48) were grouped into two as brine-salted and non-salted, and treated at seven different drying temperatures of 30, 35, 40, 45, 50, 55 and 60°C for a period of 23 h each. To evaluate the impact of Maillard reaction, reactive lysine was also quantified. Drying temperature had no effect on the nutrient profiles of tambaqui fish ($P > 0.05$) while brining reduced the overall protein concentration by 6% (58.8 to 55.4 g/100 g DM; $P = 0.004$). Brining significantly diminished many amino acids taurine by 56% (7.1 to 3.1 g/kg; $P < 0.001$), methionine 17% (14.7 to 12.1 g/kg; $P < 0.001$), cysteine 11% (5.1 to 4.4 g/kg, $P = 0.016$), and reactive lysine 11% (52.0 to 46.4 g/kg; $P = 0.004$). However, alanine, arginine, and serine were not affected by brining ($P > 0.05$). Brining also reduced the concentrations of Se by 14% (149 to 128 µg/kg dry matter; $P = 0.020$), I 38% (604 to 373 µg/kg DM; $P = 0.020$), K 42% (9.71 to 5.61 g/kg DM; $P < 0.001$) and Mg 18% (1.32 to 1.10 g/kg DM; $P = < 0.001$) versus an anticipated vast increase in Na by 744% (2.70 to 22.90 g/kg DM; $P < 0.001$) and ash 28% (12.4 to 16.0 g/100g DM; $P < 0.001$) concentration. Neither brining nor drying temperature induced changes in lysine reactivity and fat content of tambaqui fish ($P > 0.05$). Agreeably, results of multivariate analysis showed a negative association between brining, Na, and ash on one side of the component and most other nutrients on the other while the lack of effect of temperature can be seen by its presence in the center. Therefore, drying without brining may better preserve the nutritive value of tambaqui fish. However, as a practical to the industry sector, it is

Formatted: Line spacing: Double

recommended that the final products are further evaluated for any pathogen of economic or public health importance.

Preservation of fish as diet ingredient is challenging in many tropical conditions due to the absence of freezing facilities, hence warranting other preservation techniques. The present study evaluated the effect of brine salting (15% *w/w*) prior to drying at different temperatures on the nutrient profiles of tambaqui fish (*Colossoma macropomum*). Whole fish samples (792 ± 16 g; 8 months old; n = 48) were grouped into two as brine-salted and non-salted, and treated at seven different drying temperatures of 30, 35, 40, 45, 50, 55 and 60°C for a period of 23 h each. To evaluate the impact of Maillard reaction, reactive lysine was also quantified. Drying temperature had no effect on the nutrient profiles of tambaqui fish ($P > 0.05$). Brining reduced the overall protein concentration by 6% ($P < 0.05$). Although brining significantly diminished many amino acids (taurine 56%, methionine 17%, cysteine 11%, reactive lysine 11% and histidine 10%), proline and glycine increased with 5 and 6%, respectively, whereas alanine and arginine were not affected. Brining also reduced the concentrations of Se (14%), I (38%), K (42%) and Mg (18%) ($P < 0.05$) versus an anticipated vast increase in Na (744%) and ash (28%) concentrations ($P < 0.05$). There was no significant effect of brining on Fe, Zn, Mn, P and Ca concentrations and brining and drying on body fat content of tambaqui fish ($P > 0.05$). From a nutritional viewpoint, drying without brining is the best approach to maintain the nutritive value of tambaqui fish; neither brining nor drying temperature induced changes in lysine reactivity.

Keywords: Brining; drying; nutrient profiles; tambaqui; reactive lysine.

Formatted: MDPI_1.7_abstract

Introduction

Fish meat is a high-value, perishable protein-rich food requiring appropriate preservation to maintain its organoleptic properties after harvest [1,2]. Nutritionally, fish meat is a prominent source of high quality essential amino acids [3,4], omega-3 fatty acids such as eicosapentaenoic

Formatted: Line spacing: Double

acid (EPA) and (docosahexaenoic acid (DHA), and trace elements such as iron, selenium, iodine that are beneficial for the human health [5]. Depending on the species of the fish, culture system (farmed versus wild), feeding behavior (herbivores, carnivores, omnivores), and agroecology (temperate versus tropical; freshwater versus marine water) these essential nutrients, and their bioavailability, vary from one to other fish. Hence, a knowledge on the nutrient profiles of fish meat do promote a balanced and healthy diet for the human consumer and endorses consumer preferences on dietary requirements [5,6].

Tambaqui fish (*Colossoma macropomum*), also known as black pacu, is the main native fish species farmed in South and Central American countries [7]. It is mainly found in Brazil, Venezuela, Colombia, Peru, and Bolivia. The species alone accounted for a total production of 142,135 tons in 2016 with Brazil accounting for about 96.4% of the total production [8]. Its life cycle is mainly associated with floody plains of white-water rivers in the amazon basins that are characterized with a temperature range of 25-34°C, low to neutral pH (6-7), and rich in suspended solids [9,10].

Tambaqui is an omnivorous fish that feeds on a variety of natural feeds such as fruits and seeds, and zooplanktons depending upon seasons [8,11]. It is considered to be a nocturnal feeder fish [7]. The higher growth performance, hardiness, and socioeconomic value of tambaqui fish have nowadays led to technology endorsement in its farm level production [9,12,13]. Nutritionally, the tambaqui is also rich in essential amino acids, EPA and DHA, selenium, and vitamins D and B [9]. Furthermore, tambaqui requires a fairly low protein content in its feed, most of which (75-85%) can be provided as plant protein [7,12]. This makes it possible to breed tambaqui with the aid of low-cost feed and feedstuffs, such as forest fruits or seeds of leguminous plants [7].

Preservation is a fundamental requirement of food and food ingredients in maintaining a quality food which is a vital issue in the human health and contributes to profitability [14,15].

Fish meat, for instance, contains several set of nutrients (moisture, proteins, carbohydrates, and vitamins) that can easily get exposed to the ubiquitous environmental microbials (bacteria, moulds…) and chemicals causing degradation. The moisture content can be regarded as the primary nutrient input for fish meat degradation and affecting the nutritive value and shelf life of the product [6]. Generally, fish meat often have higher moisture content averaging 70-84% where bacteria can grow rapidly and cause spoilage [4]. The suppression or killing of such microorganisms can be achieved by either reducing the temperature (e.g. chilling, freezing) or reducing the water content (e.g. drying, salting) [15]. Drying of fish meat products can be achieved by traditionally sun-drying or open air drying and using air driers, or microwaves [5,17]. Salting, on the other hand, involve different processes such as brining (immersion of fish meat into salt solution), kench curing (dry salting of fish fillets), pickling (longer storage of the fish with brine), and the possible injection of brine into the fish muscle all ending up in reducing product water content and hence extended shelf life [5,18-21].

Brine salting is one of the oldest traditional and economically viable preservation methods of edible animal proteins such as fish meat [22-24]. It involves the use of salt (NaCl) that, due to its hygroscopic property, withdraws water from tissues via osmosis and, thereby, reduces water activity to slow bacterial growth [22]. In addition, salt is also reported to improve product flavor, extract proteins, aid in emulsion formation, and reduce proteolytic activities of enzymes [25-27].

The quantity and concentration of salt and treatment time are determining factors in the process that could affect the nutritional quality of the fish [28-30]. A 15% brine concentration for a period of 48 h has been reported to yield optimum protein solubility/availability in a comparative study carried out in cod [24]. Likewise, better mass transfer kinetics of salt to tissues of cod and salmon were observed at 15% (*w/w*) brine concentration [23]. In fact, 10-15% (*w/w*) is the salt concentration recommended for salting whole fish under the food safety

and standards regulation [31]. Higher concentrations of up to 25% (*w/w*) denature muscle proteins by causing cell membrane disruption, decreased enzymatic activity, or interaction with myofibrils, and reducing its water-holding capacity [23,24,32]. In general, higher yields of tissue proteins are obtained at lower salt concentrations [24,26,33]. In addition to the salt concentration, the purity of salt, salting method involved, the size, thickness and freshness of the fish, fish-to-salt ratio, and weather conditions are further factors of importance for the quality brine preserved fish [22,23].

Drying involves the exposure of a product to specified temperature and time period to facilitate loss of tissue water to a lower limit of water activity (a_w < 0.90) where microbial growth is inhibited [34]. Drying temperature, relative humidity and air velocity are determining the rate of dehydration [2]. In rural areas, temperature-controlled ovens are usually not available, and open-air drying is the only option. Especially high environmental temperatures during drying may affect the nutritive value of fish, for instance because heat may damage amino acids or may increase the reaction of amino acids with lipids and/or carbohydrates to form biologically unavailable Maillard products [35]. To determine the risk of drying at uncontrolled temperature for the nutritive value of fish, it is warranted to study the effect of a wide enough temperature range for fish drying.

Depending on the type of drying oven and species and size of the fish involved, a temperature range of 65 - 135°C and drying time of 2 - 20 h have been routinely used for drying of fish meat [2,26]. However, due to possible conjoint loss of some non-moisture compounds (e.g. volatile fatty acids and vitamins), lower temperatures with extended drying time are often recommended [36]. For example, drying of salted shark at 35°C reported to yield improved protein solubility, tissue rehydration and water retention capacity and total protein estimates compared to 45, 55 and 60°C [37]. Pacheco [38] also stated that the maximum drying temperature for the majority of the edible fish meat should not exceed 70°C. In fact, drying of

fish products at higher temperatures, of more than 60°C, reported to cause fat oxidation and caramelization of sugars [37].

Despite the effect of brining and drying temperatures on the nutrient profiles of different fish species has been investigated, we hypothesized that species differences in nutrient profiles could respond differently to drying and brining and evaluated tambaqui fish. Our study also investigated reactive lysine as a marker for Maillard product formation: lysine is a limiting amino acid in most animal diets and need to be bioavailable to be used in metabolism. Despite its high concentration in tissue deposition, some reducing sugars, fats and their oxidation products and vitamins can bind the ε-amino group of lysine and generate Maillard reaction products during fish processing [35]. Hence, measuring the content of "reactive lysine" as a percentage of total lysine in tambaqui fish can indicate state of protein damage during processing. The present study therefore evaluated the effect of brining and a range of drying temperatures on the macronutrients and micronutrients, and mineral composition as well as on the state of Maillard reaction that may reduce lysine availabilityFish meat is a high-value, perishable protein-rich food requiring appropriate preservation to maintain its organoleptic properties after harvest [1,2]. Freezing is an efficient manner for fish preservation, but in many rural areas of the Global South, freezing fish is not an option because of poor electricity supply and the unaffordable cost of freezers. Alternative methods for fish preservation are warranted in such conditions. Brine salting is one of the oldest traditional and economically viable preservation methods of edible animal proteins such as fish meat [3-5]. It involves the use of salt (NaCl) that, due to its hygroscopic property, withdraws water from tissues via osmosis and, thereby, reduces water activity to slow bacterial growth [3]. In addition, salt is also reported to improve product flavor, extract proteins, aid in emulsion formation, and reduce proteolytic activities of enzymes [6-8].

The quantity and concentration of salt and treatment time are determining factors in the process that could affect the nutritional quality of the fish [9-11]. A 15% brine concentration for a period of 48 h has been reported to yield optimum protein solubility/availability in a comparative study carried out in cod [5]. Likewise, better mass transfer kinetics of salt to tissues of cod and salmon were observed at 15% (*w/w*) brine concentration [4]. In fact, 10-15% (*w/w*) is the salt concentration recommended for salting whole fish under the food safety and standards regulation [12]. Higher concentrations of up to 25% (*w/w*) denature muscle proteins by causing cell membrane disruption, decreased enzymatic activity, or interaction with myofibrils, and reducing its water-holding capacity [4,5,13]. In general, higher yields of tissue proteins are obtained at lower salt concentrations [5,7,14]. In addition to the salt concentration, the purity of salt, salting method involved, the size, thickness and freshness of the fish, fish-to-salt ratio, and weather conditions are further factors of importance for the quality brine preserved fish [3,4].

Drying involves the exposure of a product to specified temperature and time period to facilitate loss of tissue water to a lower limit of water activity (a_w < 0.90) where microbial growth is inhibited [15]. Drying temperature, relative humidity and air velocity are determining the rate of dehydration [2]. In rural areas, temperature-controlled ovens are usually not available, and open-air drying is the only option. Especially high environmental temperatures during drying may affect the nutritive value of fish, for instance because heat may damage amino acids or may increase the reaction of amino acids with lipids and/or carbohydrates to form biologically unavailable Maillard products [16]. To determine the risk of drying at uncontrolled temperature for the nutritive value of fish, it is warranted to study the effect of a wide enough temperature range for fish drying.

Depending on the type of drying oven and species and size of the fish involved, a temperature range of 65 - 135°C and drying time of 2 - 20 h have been routinely used for drying

of fish meat [2,7]. However, due to possible conjoint loss of some non-moisture compounds (e.g. volatile fatty acids and vitamins), lower temperatures with extended drying time are often recommended [17]. For example, drying of salted shark at 35°C reported to yield improved protein solubility, tissue rehydration and water retention capacity and total protein estimates compared to 45, 55 and 60°C [18]. Pacheco [19] also stated that the maximum drying temperature for the majority of the edible fish meat should not exceed 70°C. In fact, drying of fish products at higher temperatures, of more than 60°C, reported to cause fat oxidation and caramelization of sugars [18]. Under tropical "rural" conditions without ovens, temperature will likely not exceed 60°C but may typically remain above 30°C [20].

Tambaqui (*Colossoma macropomum*, Cuvier, 1818) is a tropical freshwater fish most widely cultivated in the Amazon region [21]. The species' higher productivity and hardiness have all increased its global demand [22]. To date, there is no information about the effect of brining and drying temperatures on the nutrient profiles of this fish species. Our study also investigated reactive lysine as a marker for Maillard product formation: lysine is a limiting amino acid in most animal diets and need to be bioavailable to be used in metabolism. Despite its high concentration in tissue deposition, some reducing sugars, fats and their oxidation products and vitamins can bind the ε-amino group of lysine and generate Maillard reaction products during fish processing [16]. Hence, measuring the content of "reactive lysine" as a percentage of total lysine in tambaqui fish can indicate state of protein damage during processing. The present study therefore simulated rural conditions of brining and a range of drying temperatures to identify how these factors influence macronutrient and mineral composition as well as Maillard reaction that may reduce lysine availability.

Materials and methods

Experimental animal ethics and welfare

All experimental and animal care procedures were realized based upon the current written knowledge of aquaculture technologies and tambaquí fish farming. Fingerlings of *Colossoma macropomum* were initially ~~imported~~ brought from Aqua-Koi NV farm (http://www.aqua-koi.be/documents/home.xml?lang=nl) to TRANSfarm and reared in a recirculating aquaculture system (RAS) with a weekly basis 30% water renewal (20L/day) for about 7 months period before being subject to the current study. During their stay, fish were kept in larger aqua-tanks (100 cm × 120 cm × 40 cm) at a density of 1.2 fish/m^3 sufficient enough for movements and behavioral expression. Tanks were fixed with individual bio-filters, well aerated with a constant temperature supply, 18:6 h light - dark cycle, and supplying pipes were regularly inspected and cleaned with tap water and water quality parameters were checked twice a week and water renewed accordingly [39].

For our experimental study, after a 12 h fasting, a total of 48 tambaqui fish were randomly selected and kept in a separate tank and were euthanized in a two-step procedure [~~2~~40~~3~~]. Six fish were randomly taken at a time and placed in a tank with a volume of 240 L water into which 50 mL of phenoxyethanol-solution (Anest Fish©, Germany) was added. After five minutes, 30 L of water were elevated out of the tanks and put into a large bucket. Another 6 mL of Anest Fish© was added to this bucket. Each group of six unconscious fish was scooped out of the tank and brought to the slaughter room in the bucket with the anesthetic agent (1mL:5L, Anest Fish©, Germany). Thereafter, tambaqui were killed with a firm blow to the head, followed by fracturing the brainstem with a knife and then transported in ice cubes for the experiment [40~~23~~]. The study was conducted in accordance with the Declaration of Helsinki and directive 2010/63/EU of the European parliament and of the council on the protection of animals used for scientific purposes, and the wellbeing of the cultured tambaqui~~í~~ fish before being subjected to the experiment was assessed and approved by the i~~I~~nstitutional ~~R~~review b~~B~~oard of

TRANSfarm, KU Leuven, lab accreditation ~~Belgium~~ (KU/LA-1210616)~~(TRANSfarm17.09/2019)~~.

Study materials

A total of 48 tambaqui fish with a mean weight of ~~(~~792 (± 16) g and~~;~~ 8 months old~~)~~ were collected from TRANSfarm, Lovenjoel, Belgium, and transported to the animal nutrition laboratory of the Department of v~~V~~eterinary and b~~B~~iosciences, Ghent University in styrofoam boxes with ice packs (Luxaplast, NORDIC®). ~~Fish were initially starved for 24 h and humanely euthanized in ice cubes before transport.~~ Upon arrival, to facilitate drying whole fish were dissected dorso-ventrally into two equal slices using a stainless post mortem saw (SC-PM/110, 254MM) and scalpel-blades, thoroughly washed using distilled water in pipes~~tap water~~ and then individually stored in plastic bags at -20°C for 48 h. All dissected fish slices had an average uniform dimension of 21.2 ± 2.8 cm long and 12.6 ± 2.0 cm wide and average weight of 389 ± 11.5 g. Industrial table salt (99.9%, BDH-PROLABO®, EC) was used for preparing the 15% (*w/w*) brine solution in the present study [24].

Experimental design and set-up

Uniformly sliced whole fish samples were grouped into two as brine-salted and non-salted, and treated at seven different drying temperatures of 30, 35, 40, 45, 50, 55 and 60°C for a period of 23 h each [23,24,31,37]. Each temperature treatment was considered as a sampling point and tested in triplicate. Similarly, as a reference point, fresh whole-fish samples were initially evaluated in triplicate for their nutritional profiles.

Brine salting and drying processes

Brine-salted fish samples were immersed in brine solution (15% *w/w*) at a ratio of 3:1 of fish to salt solution [22,27]. For each sampling unit, samples were totally immersed and brined together in a closable plastic bucket (20 L) for 48 h at 4°C. The brine solution was pre-cooled

to 4°C prior to the immersion of samples. Afterwards, brined samples were drained from salt drips for 3 min using a plastic sieve and randomly distributed into individual aluminium trays (500 mL; 142 mm × 116 mm × 30.4 mm; Boni™, Halle, Belgium), at one fish per try, into which small beads (13 mm diameter, 3.5 g, JVLAB®) were added (10 beads/tray) at the bottom to avoid crusting of samples and optimize moisture loss. Likewise, the non-salted group of fish samples were randomly distributed into identical aluminium trays containing similar beads. E~~To simulate environmental drying temperature in rural conditions, e~~ach tray was placed in an electric oven (EP66, BEKSO-SA®, Belgium) and randomly set and dried with the above-mentioned temperature points. The laboratory humidity was in a range of 50-60% during the experimental period. Drying temperature between sampling units were controlled using an oven-fixed thermometer and a consistent oven temperature (± 1°C) was maintained throughout the drying time period. A constant air ventilation was supplied through an oven-fixed ventilator at a steady air velocity of 1.0 m/s. Drying time started after oven closure and the oven returned to its set temperature (~ 5 min). After 23 h, samples were collected and allowed to cool in a desiccator for 5 min to room temperature before weighing. Samples were then kept at -21°C overnight and finally freeze-dried (CoolSafe™, SCANVAC, Denmark) for a period of 16 h each to a stable weight, uniformly ground (2.0 mm sieve), and homogenized using a grinder machine (De´Longhi™, KG48) at 12–× speed and then individually kept in closable plastic containers at 4°C until analyzed [22].

Formatted: Superscript

Nutrient evaluations

Proximate analysis

So as to make legitimate comparisons between the processed samples, the quantitative analysis of the values of macromolecules is a significant nutritional marker and for which case the proximate analysis was used to determine the crude protein (CP), ether extract and ash contents of fish samples. The dry matter (–DM) content of freeze-dried samples (1 g) was

determined through oven-drying at 105°C for 24 h to constant weight [40~~24~~]. Ether-extract was determined using the acid hydrolysis method [41~~24~~], CP using the Kjeldahl method involving a nitrogen ~~analyser~~analyzer (LECO FP528 Instruments, St. Joseph, Michigan, USA) and N × 6.25 conversion factor [42~~25~~], and ash content using an automated muffle furnace (Nabertherm™, UK) as per the procedures described by AOAC [43~~26~~]. A gram of fish sample was used for the analysis of each of the macronutrients and all samples were evaluated in duplicate at the animal nutrition laboratory of the Department of Veterinary and Biosciences, Ghent University, Belgium.

Mineral analysis

The total ash content was determined following the ISO method [44~~27~~] and mineral (calcium, sodium, potassium, copper, iron, magnesium, manganese, phosphorus and zinc) concentrations of fish were evaluated after dry ashing mineralization according to ISO method (ICP-AES: ISO 11885) as per the procedures described by Gorsuch [45~~28~~]. In brief, a gram of sample was weighed, placed into a porcelain crucible and oven-dried at 105°C for 3 h. Sample was then burnt on a hot plate and incinerated in a muffle furnace at 450°C for 16 h. Obtained ash was then moistened with a small amount of deionized water first and dissolved in 10 mL of 1:1 (*v/v*) solution of hydrochloric acid (HCl) and deionized water and evaporated to dry. Finally, the mix was re-dissolved in 10 mL of 1:9 (*v/v*) solution of HCl and deionized water, transferred into a 50 mL flask, and then diluted several folds for optimal determination of individual mineral concentration using induction-coupled plasma optical emission spectrometry (ICP-OES) method as per the manufacturer's procedures (Iris Intrepid II XSP, Thermo Fisher Scientific, Aalst, Belgium). So as to obtain an optimum concentration range for the atomic absorption spectrometric method, the concentrations of certain minerals were determined after fivefold (Mg and Ca), 10-fold (Na and Zn), and 50-fold (K) dilutions. Likewise, the concentration of selenium was determined by ISO method (ICP-MS: ISO 11885) and iodine

with tetramethylammonium hydroxide extraction and iodine quantification (ICP-MS: ISO 11885) at the Laboratory of Chemical analysis, Ghent University, Belgium. As quality control protocols, visual inspection of pump tubing at regular intervals and replacing between samples are often made to increase the precision measurements. Similarly, regular inspection and cleaning of the injector tube, spread chamber and mobilizer, and cleaning of the widows are done to avoid contamination and reduction of light intensity. Blanc solutions with similar matrix were used to improve the measurement accuracy and the detection limit set was 1-100 ppb.

Amino acid and OMIU-reactive lysine analyses

The total amino acid profiling was done by acid hydrolysis (2 g) at 110°C for 23 h and ion-exchange chromatography with post-column derivatization with ninhydrin method (ISO 13903). Sulphur containing amino acids were measured as cysteic acid and methionine sulfone after oxidation with performic acid; tryptophan was measured by alkaline hydrolysis at 110°C for 20 h and ion-exchange chromatography with fluorescence detection method (ISO 13904) according to procedures described by Hulshof [4629].

For analysis of reactive lysine, about 2 g of fish sample were processed from each of the seven sampling points for defatting by extraction with light petroleum ether without acid hydrolysis (ISO 6492), finely ground and homogenized (2.0 mm sieve size) using a mixer mill (Retsch MM200, BV) and used for the analysis of reactive lysine via O-methylisourea (OMIU) method according to Hulshof [4629]. Briefly, 1 mL of 0.6 M solution of OMIU was prepared as described by Moughan and Rutherfurd [4730] that converts lysine with free e-amino group to homoarginine. Four gram of barium hydroxide octahydrate (Sigma-Aldrich, Zwijndrecht, the Netherlands) was added to 16 mL of boiling (10 min) distilled deionized water to remove CO_2, after which 2 g of OMIU sulphate salt was added. The solution was then cooled for 30 min at room temperature, centrifuged (6,400 × g for 10 min at 20°C) and the supernatant was retained and the precipitate was washed with about 2 mL of same water and further centrifuged (6,400

× g for 10 min at 20°C). both supernatants were combined and pH adjusted to 11.5 (for guanidation) by adding 6 M HCl solution. The homoarginine content was analysed in duplicate and the OMIU-reactive lysine was calculated from the homoarginine content. All amino acid profile, including reactive lysine, were determined at Wageningen University & Research, Animal n~~N~~utrition laboratory, The Netherlands.

Statistical analysis

Data obtained in the current study were analyzed using SPSS statistical software version 27 (IBM Corporation, Armonk, NY, USA). All data were checked for normality distributions using histogram and QQ-plots. A univariate one-way ANOVA was used with brining as fixed factor and temperature as covariate to test the hypothesis about differences between two or more mean nutrient values. D~~All d~~ata were presented as means ~~of three values~~ and standard error of the means (SEM). Statistical significance was set at $P < 0.05$ for any differences observed between samples. Furthermore, ~~multivariate~~ principal component analysis~~analysis~~ (PCA) was computed using ~~principal component analysis (PCA)~~ on the collective factors obtained to appraise the relationships of nutrient profiles based on their responses towards brining and drying methods, with values deemed relevant below -0.5 and above 0.5 of the components' values.

Results

Tambaqui fish samples were grouped into two as brine-salted and non-salted, and treated at seven different drying temperatures for 23 h each. Each temperature treatment was considered as sampling point and tested in triplicate (n = 3) and mean values obtained were statistically computed for all nutrient profiles evaluated.

Formatted: Normal, Justified

Formatted: Font: 12 pt, Not Bold

Proximate analysis

Drying temperature had no significant effect on the measured nutrient profiles of the fish in this study and all data were combined across temperature treatments ($P > 0.05$; Fig 1). However, several effects ($P < 0.05$) were observed with the brining method.

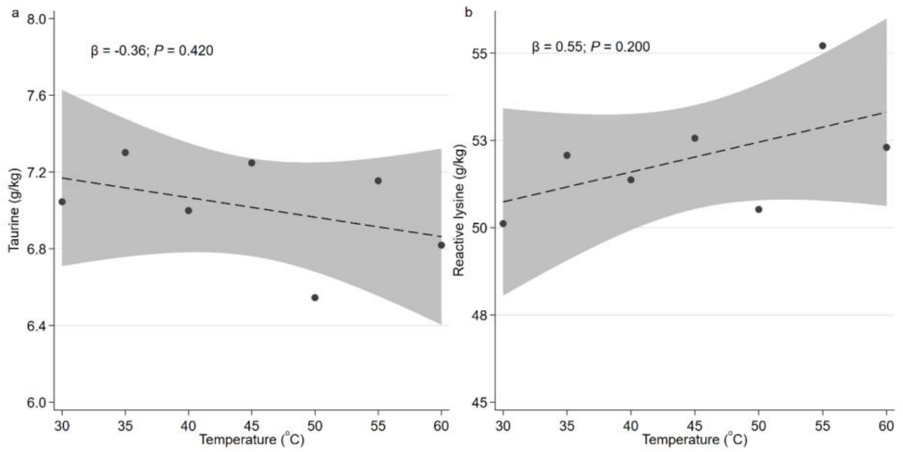

Fig 1. The effect of drying temperature on nutrient profiles of tambaqui fish. (a) gram of taurine per kg fish; (b) gram of reactive lysine per kg fish.

Brining resulted in a higher ash concentration with a relative increase of 28% ($P < 0.05$). Moreover, 6% loss on the overall protein concentration was noticed due to brining ($P < 0.05$). The body fat contents of tambaqui fish were not affected by the addition of salt and temperature treatments ($P > 0.05$; Table 1).

Table 1. Changes in macronutrient concentrations in tambaqui fish (on %DM basis) due to brining and drying at seven different temperatures (30, 35, 40, 45, 50, 55 and 60°C).

Concentration (g/100 g DM)	Not salted	Salted	Total	Relative % Change	SEM	*p*	
						salted	temp
Ash	12.4	16.0	14.1	28	0.5	< 0.001	0.793
Crude protein	58.8	55.4	57.0	-6	0.6	0.004	0.846

Crude fat	28.3	28.7	28.7	1	0.4	0.716	0.657

DM = dry matter; SEM = standard error of the means; temp = temperature.
Values represent mean and standard error of the means based on three replicates per treatment and seven treatments; n = 48.

Minerals and amino acids

The essential minerals Se and I, and the macrominerals K and Mg were significantly reduced by brining with 14, 38, 42 and 18% percent losses, respectively ($P < 0.05$). Moreover, brining resulted in an obvious enormous significant increase (744%) in Na concentration ($P < 0.05$). Otherwise, all minerals (Fe, Mn, P and Ca) were decreased by brining (Table 2).

Table 2. Changes in mineral concentrations in tambaqui fish due to brining and drying at seven different temperatures (30, 35, 40, 45, 50, 55 and 60˚C).

Minerals	Not salted	Salted	Total	Relative % Change	SEM	*p*	
						salted	temp
**Macrominerals (g/kg DM)**							
Ca	37.4	37.1	37.0	-1	1.1	0.965	0.965
K	9.71	5.61	7.71	-42	0.6	<0.001	0.495
Mg	1.32	1.10	1.10	-18	0.03	<0.001	0.880
Na	2.70	22.9	12.1	744	2.7	<0.001	0.564
P	21.7	20.4	21.0	-6	0.6	0.317	0.814
**Microminerals (mg/kg DM)**							
Fe	42.0	36.9	39.2	-12	2.4	0.256	0.188
I*	604	373	489	-38	49	0.020	0.264
Mn	9.80	8.81	9.31	-10	0.3	0.115	0.152
Se*	149	128	137	-14	5	0.020	0.449
Zn	48.5	50.6	49.6	4	1.3	0.473	0.588

DM = dry matter; SEM = standard error of the means; temp = temperature.
Values represent mean and standard error of the means based on three replicates per treatment and seven treatments; n = 48.
*Values are presented in µg/kg DM.

Brining negatively affected the overall sum amino acid profile ($P < 0.05$). In particular, taurine, histidine, cysteine, methionine and available (reactive) lysine were affected by brining

Formatted: Font: 11 pt
Formatted: Font: 11 pt
Formatted: Font: 11 pt
Formatted: Font: 11 pt
Formatted: Line spacing: Multiple 1.15 li
Formatted: Font: 11 pt
Formatted: Font: 11 pt
Formatted: Space After: 12 pt, Line spacing: Multiple 1.15 li
Formatted: Space Before: 2 pt
Formatted: Justified
Formatted: Font: 11 pt
Formatted: Line spacing: Multiple 1.15 li
Formatted: Font: 11 pt
Formatted: Font: 11 pt
Formatted: Font: 11 pt
Formatted: Font: 11 pt
Formatted: Font: 11 pt
Formatted: Font: 11 pt

with the highest percent loss accounted for taurine (56%) followed by methionine (17%), cyteine (11%), and histidine (10%) ($P < 0.05$). In contrast, there was numerical 5 and 6% increase in proline and glycine respectively, whereas alanine and arginine were not affected by brining ($P > 0.05$; Table 3).

Table 3. Changes in amino acid concentrations in tambaqui fish due to brining and drying at seven different temperatures (30, 35, 40, 45, 50, 55 and 60˚C).

Amino acids (g/kg CP)	Not salted	salted	Total	Relative % Change	SEM	*p* salted	*p* temp
Taurine	7.10	3.10	5.11	-56	0.5	<0.001	0.324
Asparagine + Aspartic acid	56.8	54.9	55.4	-3	0.9	0.268	0.671
Threonine	27.0	26.3	26.5	-3	0.4	0.311	0.965
Serine	25.0	24.9	24.8	0	0.2	0.807	0.777
Glutamine + Glutamic acid	91.4	88.3	89.2	-3	1.3	0.247	0.851
Glycine	75.7	80.2	79.3	6	2.5	0.361	0.750
Alanine	51.1	51.0	51.2	0	0.7	0.961	0.636
Valine	30.8	29.2	29.8	-5	0.5	0.062	0.642
Isoleucine	26.0	24.6	24.9	-5	0.5	0.162	0.838
Leucine	44.3	41.8	42.5	-6	0.9	0.134	0.855
Tyrosine	17.9	16.5	16.9	-8	0.5	0.098	0.952
Phenylalanine	24.5	23.4	23.8	-5	0.4	0.134	0.874
Histidine	15.5	14.0	14.6	-10	0.4	0.054	0.400
Lysine	51.1	47.4	48.6	-7	1.1	0.070	0.863
Arginine	48.3	48.3	48.5	0	0.5	0.999	0.464
Proline	47.5	49.9	49.5	5	1.3	0.356	0.660
Cysteine	5.10	4.40	4.60	-11	0.1	0.016	0.746
Methionine	14.7	12.1	13.3	-17	0.4	<0.001	0.675
Tryptophan	5.10	4.60	4.71	-8	0.2	0.229	0.698
Reactive lysine	52.0	46.4	48.5	-11	1.2	0.004	0.851
% Reactive lysine	102.2	97.9	99.9	-4	1.8	0.306	0.760
Sum AA	665	645	653	-3	6.87	0.017	0.621

CP=, Ccrude protein; AA=, Aamino acid; SEM=, s Standard error of the means; temp=, temperature.

Formatted: Portuguese (Brazil)
Formatted: Left
Formatted Table
Formatted Table
Formatted Table
Formatted: Font: 11 pt
Formatted: Line spacing: Multiple 1.15 li

Values represent mean and standard error of the means based on three replicates per treatment and seven treatments; n = 48.

[a]~~Is the p~~Percentage of reactive lysine in the total protein.

[b]~~Is the p~~Percentage of reactive lysine in the total lysine.

Formatted: Space After: 12 pt, Line spacing: Multiple 1.15 li

Multivariate classification of variables

Formatted: Space Before: 2 pt

~~Multivariate statistical analysis (PCA~~Principal component analysis~~)~~ was computed on the collective factors obtained to appraise the relationships of nutrient profiles based on their responses towards brining and drying methods. By examining at the scree plot and eigenvalues and looking for a point at which the proportion of variance explained by each subsequent principal component drops off, an elbow in the screen plot, the most explaining two components 1 (44.8%) and 2 (18.9%) were considered and a biplot PCA generated.

~~-~~In line, there were no associations between nutrient profiles and drying temperature while several associations were observed with brining. Negative association was seen between brining, Na and ash on one side of component 1 and most other nutrients on the other. However, positive association between the brining proxies (Na, ash) and amino acids prominent in connective tissue (glycine, proline, arginine, alanine) were typically seen clustered together right of the component. The more central position of Zn, Fe, fat, and available Lys point to their low response to brining and temperature. More importantly, temperature itself was positioned centrally in the plot, emphasizing the absence of drying temperature effects on the study parameters (Fig 2).

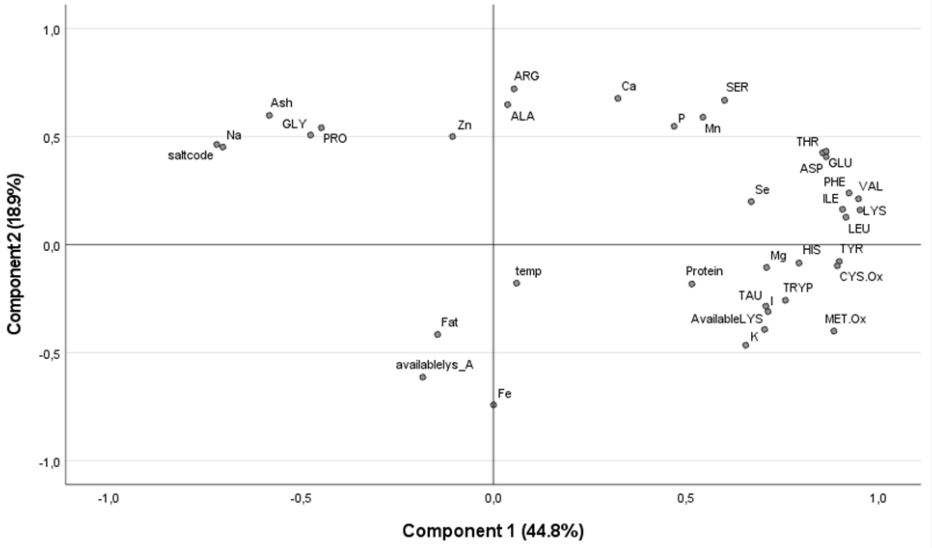

Fig 2. ~~Multivariate~~ Principal component analysis classification of~~classification of~~ nutrient profiles based on their response to brining and temperature. Analyses represent principal component analysis (PCA) of evaluated nutrient values (expressed as percentages of the sum of total) after brining and drying of whole tambaqui fish samples (n = 48). AvailableLYS, is the percentage of reactive lysine in the total protein; availablelys_A, is the percentage of reactive lysine in the total lysine; saltcode, refers to brining; temp, is the drying temperature.

Discussion

The present study evaluated the effect of brine salting (15% *w/w*) prior to drying at different temperatures on the nutrient profiles of tambaqui fish. Brining induced a prominent effect on macronutrient and micronutrient profiles of tambaqui fish.

Formatted: Font: Italic

In this~~our~~ study, brining induced pronounced losses of specific amino acids and minerals, irrespective of drying temperature. ~~Salt, a hygroscopic compound, can absorb water molecules from muscle tissues and cause protein damage [5,11]. The causes for protein damage can be related to direct cell membrane disruption, decreased activity of antioxidant enzymes and the increased release of ferric (Fe^{3+}) ions and formation of ferryl-myoglobin and metmyoglobin in~~

~~damaged tissue [31,32].~~ Gill et al. [48~~33~~] found that salting enhanced the aggregation of myosin in cod, yet only above a drying temperature of 50°C. Marínez-Alvarez and Gómez-Guillén [33~~14~~] indicated that there is a notable loss of soluble muscle proteins (particularly, actin and myosin) by osmosis in salting (NaCl) at pH 6.5 on cod muscle (*Gadus morhua*). ~~Indeed, drying of fish products at higher temperatures, of more than 60°C, resulted in a proportional decrease in protein solubility, and cause fat oxidation and caramelization of sugars [18].~~ Differences between studies can evolve from a wide range of conditions used in the drying process including the drying type, drying temperature and duration, relative humidity, air velocity and the specific characteristics of the product dried (species and size of the fish). Although comparison with existing literature does not allow us to pinpoint the factors that protected from a drying temperature effect, this study suggests ~~it is clear~~ that brining ~~completely overruled the drying temperature in its effect on~~is associated with nutrient losses. In the present study, a total of 6% ~~tissue~~ crude proteins~~s~~ was lost due to the brining stress. In fact, salt concentration and salting duration can modulate the effect of brining on muscular protein loss in fish [28-309~~-11~~]. The solubility of muscle proteins often start to decrease with increasing salt concentration due to protein aggregation [24~~5~~] and/or altered enzymatic activities [49~~34~~]. In the present study, however, an acceptable (15% *w/w*) salt was used for brining [31~~12~~].

The amino acid analysis in this~~our~~ study demonstrated that the salt-induced protein loss was not an overall effect, but reduced specific amino acids, taurine, histidine, cysteine, methionine and available lysine. Taurine is not involved in protein synthesis but often accumulates in muscle tissues of fish and has osmolytic properties [50,51~~35,36~~]. Hence, the loss in taurine seem a logical reaction to the osmotic stress from brining. This taurine loss can be important since its role in health has been demonstrated for instance in infants [52~~37~~] and it is also the main amino acid to form conjugated bile acids in chickens [53~~38~~].

The tendency to a lower histidine concentration due to brining can be seen in the same light. Apart from its protein-bound appearance, histidine is stored in muscular tissue as either free histidine (acetylated or not) or bound to mainly beta-alanine (hence constituting dipeptides such as carnosine or anserine); this storage of histidine occurs throughout the animal kingdom [5439]. Shiau [5540] reported that histidine can easily get released from muscle tissues during stress conditions such as starvation. Significant losses of the free amino acids histidine, taurine and glycine were reported in salted milkfish [3213]. Histidine is involved in the regulation and metabolism of essential trace minerals, formation of enzymes, induction of the immune system through the production of histamine and protects brain cells through the formation of metallothionein among others [5641].

Cysteine and methionine are sulphur-containing amino acids and quite reactive to processing, in that they could either get directly affected by the ionic brine solution or can possibly make cross-links that reduces their tissue availability [3213]. Kumazawa [5742] reported that brining may cause cross-linking reactions of muscle myosin heavy chains and form ε-(γ-glutamyl)lysine through transglutaminase. There was no effect on the percentage of reactive lysine (% available-lysine), meaning that both salting and drying did not induce Maillard reactions that affect lysine availability. The relative decrease in the total lysine likely explains the decreased percentage of available lysine in the current study.

Four specific amino acids in the principal component analysis separated from the negative association between brining and the other amino acids: alanine and arginine were not affected while glycine and proline were even positively associated with brining. These amino acids are prominent in the skeleton and in scales, which is a strong indication that the highest protein losses were from muscle and other soft tissues [5843]. Other authors not always report similar changes in amino acid pattern: a sigmoid initial increase (up to 9 h) and final slight decrease (48 h) of alanine, glutamine and lysine was reported in salted milkfish [3213]; alanine, glycine,

lysine and histidine increased when salted fish was dried [5944]. The increase in these amino acid concentrations could be due to the decrease in the proportion of the extracted total lipids present as polyunsaturated fatty acids as depicted by Yankah [5944]. Fat is a hydrophobic molecule and can act as a physical barrier to limit salt (NaCl) and water transport across the muscle tissue [234]. In fact, the lipid content was higher for the fish in our study (28.71%) although fat concentration was not affected by both treatments.

The specific loss of soft tissue from the fish is not only supported by the amino acid data, but also witnessed by the change in mineral profile. The considerable loss of the microminerals Se and I and the macrominerals K and Mg following brining reflects the presence of these minerals in soft tissue [6045]. Selenium typically occurs as replacement of sulphur in sulphur-containing amino acids (cysteine and methionine) in soft tissues. The decrease in methionine and cysteine seen in our study, therefore, fits the observation of decreased selenium. In contrast, the typical skeletal minerals such as Ca, P and Mg, and those that are deposited in scales and fins such as Zn and Fe, were not affected. Potassium is a strong electrolyte that could disappear in the brine solution or get filtered out in exchange with the added sodium. The loss of water due to salt was explained as the main cause for leaching of these macrominerals in the solution during brining. The decrease in K concentration agrees with Polak-Juszczak [6146] and Bakhiet [6247] from salted fish. The latter study remarkably reported a salt-induced increase in iodine concentration, most likely evolving from salt impurities as proposed by the authors. It is not clear how these differences with our study can be explained, but we used a purified source of salt, hence excluding potential unintended supply of microminerals to the fish tissues through brining.

Drying is an energy intensive process which can use about 10% or more of the total energy consumed during processing. In the present study, although our aim was not to quantify the drying efficiency, we conjointly were aiming to consume less energy while drying samples by

increasing the sample surface area of exposure, avoiding surface contact by putting beads, improved equipment insulation during and between drying, supply of a constant air ventilation at 1.0 m/s during drying, and minimizing the timing between dryings [63~~48~~].

Undeniably, brining has substantially increased Na and ash concentrations. This has been observed in most studies on brining of fish [61,62~~46,47~~]. This enrichment with salt through brining must be considered when formulating diets but, more importantly, our results demonstrate that brining may not be the best choice to maintain the nutritive value of fish. Evidently, we must acknowledge that nutritive value is not the only criterion to pick a preservation method for fish: for the reduction of the pathogenic load in fish products, salt addition may still be a valuable technique that goes, however, at the expense of nutritive value as shown in our study. A comforting finding is that the wide range in drying temperature evaluated in our study had no noticeable impact on the nutritive value of the fish. This implies that drying of fish under uncontrolled environmental temperature seems a suitable option in rural areas to preserve the nutritive value of (tambaqui) fish.

5. Conclusions

This research shows that drying temperature has no effect on nutrient composition of the studied fish which could be encouraging for drying of fish under – less controlled – rural tropical conditions. Brining, however, reduced some important trace elements such as Se and I, and lead to loss of muscle crude protein, with the concomitant amino acids. Brining also increased the leaching of K, I, and taurine. The formation of Maillard products was not promoted by both drying and brining treatments, as demonstrated through unaltered reactive lysine proportions. Therefore, drying without brining may better preserve the nutritive value of tambaqui fish. However, as a recommendation to the industry sector, it is suggested that the final products are further evaluated for any pathogen of economic or public health importance.~~The lack of a drying temperature effect on fish composition in this study is~~

~~encouraging for drying of fish under less controlled rural tropical conditions. Brining, however, reduced some important micronutrients such as Se and I, and lead to important loss of muscle tissue, with the concomitant amino acids. The formation of Maillard products was not promoted by the drying and brining treatments, as demonstrated through unaltered reactive lysine proportions. Brining also increased the leaching of K, I and taurine. Drying without brining may therefore better preserve the nutritive value of tambaqui fish, although this must be considered in combination with the goal of pathogen reduction.~~

Acknowledgments

The authors acknowledge Herman De Rycke for the proximate analysis.

References

1. Pawar SM, Sonawane SR. Fish muscle protein highest source of energy. Int J Biodivers Conserv. 2013; 5:433–435. doi: 10.5897/IJBC12.043.
2. di Domenico CNB. Fish drying: A review. Rev Bras Eng Pesca. 2014; 7:1–18. doi: 10.18817/repesca.v7i1.1061.
3. Marta M, Francisca H, Robert S. Fatty acids profile in canned tuna and sardine after retort sterilization and high pressure thermal sterilization treatment. J Food Nutr Res. 2015; 54:171–178. doi: hdl.handle.net/10261/114599.
4. Abraha B, Admassu H, Mahmud A, Tsighe N, Shui XW, Fang Y. Effect of processing methods on nutritional and physico-chemical composition of fish: a review. MOJ Food Process Technol. 2018; 6:376-82. doi: 10.15406/mojfpt.2018.06.00191.
5. Sampels S. The effects of processing technologies and preparation on the final quality of fish products. Trends Food Sci Technol. 2015; 44:131-146. doi: 10.1016/j.tifs.2015.04.003.

Formatted: Font color: Auto
Formatted: English (United States)
Formatted: Font color: Auto, English (United States)
Formatted: English (United States)
Formatted: Font color: Auto
Formatted: Font color: Auto
Formatted: Font color: Auto
Formatted: English (United States)
Formatted: English (United States)

6. Kumar MS. Sustainable management of fisheries and aquaculture for food security and nutrition: policies requirements and actions. Agric Res. 2014; 3:97-103.

7. da Silva JAM, Pereira-Filho M, Oliveira-Pereira MI. Seasonal variation of nutrients and energy in tambaquí's (*Colossoma macropomum* Cuvier, 1818) natural food. Braz J Biol. 2000; 60:4. doi: 10.1590/s0034-71082000000400009

8. Woynárovich A, van Anrooy R. Field guide to the culture of tambaquí Colossoma macropomum, Cuvier, 1816. In: FAO Fisheries and Aquaculture Technical Paper. 2019; 624:21-30.

9. Arantes ML, Freitas CEC. Effects of fisheries zoning and environmental characteristics on population parameters of the tambaqui (*Colossoma macropomum*) in managed floodplain lakes in the Central Amazon. Fish Manag Ecol. 2016; 23:133–143. doi: 10.1111/fme.12164

10. Val AL, de Oliveira AM. *Colossoma macropomum*—A tropical fish model for biology and aquaculture. J Exp Zoo Part A: Ecol Integ Phys. 2021; 335:761-70. doi: 10.1002/jez.2536

11. Barat, J.M., Rodríguez-Barona, S., Andrés, A., & Fito, P. (2002). Influence of increasing brine concentration in the cod salting process. J Food Sci. 65(7):1922–1925. doi: 10.1111/j.1365-2621.2002.tb08747.x.

12. Gomes LC, Silva CR. Impact of pond management on tambaquí, *Colossoma macropomum* (Cuvier), production during growth-out phase. Aqua Res. 2009; 40:825–832. doi: 10.1111/j.1365-2109.2009.02170.x

13. Sandre LCG, Buzollo H, Nascimento TMT, Neira LM, Jomori RK, Carneiro DJ. Productive performance and digestibility in the initial growth phase of tambaquí (*Colossoma macropomum*) fed diets with different carbohydrate and lipid levels. Aqua Rep. 2017; 6:28–34. doi: 10.1016/j.aqrep.2017.02.003

Formatted: English (United States)
Formatted: Portuguese (Brazil)
Formatted: Font: Italic
Formatted: Font color: Auto
Formatted: English (United States)
Formatted: English (United States)
Formatted: Portuguese (Brazil)
Formatted: Font: Italic
Formatted: Font color: Auto
Formatted: English (United States)
Formatted: Portuguese (Brazil)
Formatted: Font: Italic
Formatted: English (United States)
Formatted: Portuguese (Brazil)
Formatted: English (United States)
Formatted: Font: Italic
Field Code Changed
Formatted: Font color: Auto
Formatted: English (United States)
Formatted: Portuguese (Brazil)
Formatted: Font: Italic
Formatted: Font color: Auto
Formatted: English (United States)

14. Heijnen C, Boogaard BVD, Maas van Berkel B. Preservation of fish and meat. Agrodok. 2004; Pp. 26-35.

15. Miladi H, Chaieb K, Bakhrouf A, Elmnasser A, Ammar E. Freezing effects on survival of Listeria monocytogenes in artificially contaminated cold fresh-salmon. Annals Microbiol. 2008; 58:471-476.

16. Mahmoud BSM, Yamazaki K, Miyashita K, Shin II, Suzuki T. A new technology of fish preservation by combined treatment with electrolysed NaCl solutions and essential oil compounds. Food Chem. 2006; 99:656-662. doi: 10.1016/j.foodchem.2005.08.037.

17. Djendoubi N, Boudhrioua N, Bonazzi C, Kechaou N. Drying of sardine muscles: Experimental and mathematical investigations. Food Bioprod Process. 2009; 87:115-123. doi: 10.1016/j.fbp.2008.07.003.

18. Birkeland S, Skåra T, Bjerkeng B, Rørå AMB. Product yield and gaping in cold-smoked Atlantic salmon (*Salmo salar*) fillets as influenced by different injection-salting techniques. J Food Sci. 2003; 68:1743-1748.

19. Guizani N, Rahman MS, Al-Ruzeiqi MH, Al-Sabahi JN, Sureshchandran S. Effects of brine concentration on lipid oxidation and fatty acids profile of hot smoked tuna (*Thunnus albacares*) stored at refrigerated temperature. J Food Sci Technol. 2014; 51:577-582. doi: 10.1007/s13197-011-0528-4.

20. Hall G. Preservation by curing (drying, salting and smoking). In: G. Hall (Ed.), Fish processing sustainability and new opportunities, West Sussex: Wiley-Blackwell. 2011; Pp. 51-76.

21. Kituu GM, Shitanda D, Silayo D, Odote P, Bongyereire J. Effect of brining on the drying rate of tilapia (*Oreochromis niloticus*) in a solar tunnel dryer. Fish Aqua Cluster Proceed. 2000; 88-98. http://ir.mksu.ac.ke/handle/123456780/4889.

Formatted: Dutch (Netherlands)
Formatted: English (United States)
Formatted: English (United States)
Formatted: English (United States)
Formatted: English (United States)
Formatted: Dutch (Netherlands)
Formatted: Font: Italic
Formatted: English (United States)
Formatted: Font: Italic
Formatted: English (United States)
Formatted: English (United States)
Formatted: Font: Italic
Formatted: Font color: Auto
Formatted: Font color: Auto
Formatted: English (United States)

~~3.~~22. Binici A, Kaya GK. Effect of brine and dry salting methods on the physicochemical and microbial quality of chub (*Squalius cephalus* Linnaeus, 1758). Food Sci Technol. 2018; 38:66–70. doi: 10.1590/1678-457X.15717.

~~4.~~23. Gallart-Jornet L, Barat JM, Rustad T, Erikson U, Escriche I, Fito P. A comparative study of brine salting of Atlantic cod (*Gadus morhua*) and Atlantic salmon (*Salmo salar*). J Food Eng. 2007; 79:261–270. doi: 10.1016/j.jfoodeng.2006.01.053.

~~5.~~24. Nguyen MV, Thorarinsdottir KA, Gudmundsdottir A, Thorkelsson G, Arason S. The effects of salt concentration on conformational changes in cod (*Gadus morhua*) proteins during brine salting. Food Chem. 2011; 125:1013–1019. doi: 10.1016/j.foodchem.2010.09.109.

~~6.~~25. Rust RE, Knipe CL. Sausage casings. In: Devine C, Dikeman M, editors. Encyclopedia of Meat Sciences. Academic Press: Oxford; 2014. pp. 235–240.

~~7.~~26. Thorarinsdottir KA, Arason S, Bogason S, Kristbergsson K. The effects of various salt concentrations during brine curing of cod (*Gadus morhua*). Int J Food Sci Technol. 2004; 39(1):79–89. doi: 10.1046/j.0950-5423.2003.00757.x.

~~8.~~27. Thorarinsdottir KA, Arason S, Sigurgisladottir S, Valsdottir T, Tornberg E. Effects of different pre-salting methods on protein aggregation during heavy salting of cod fillets. Food Chem. 2011; 124(1):7–14. doi: 10.1016/j.foodchem.2010.05.095.

~~9.~~28. Barat JM, Rodríguez-Barona S, Andrés A, Fito P. Influence of increasing brine concentration in the cod salting process. J Food Sci. 2002; 67(5):1922–1925. doi: 10.1111/j.1365-2621.2002.tb08747.x.

~~10.~~29. Larsen R, Elvevoll EO. Water uptake, drip losses and retention of free amino acids and minerals in cod (*Gadus morhua*) fillet immersed in NaCl or KCl. Food Chem. 2008; 107(1):369–376. doi: 10.1016/j.foodchem.2007.08.031.

Formatted: Font: Italic

~~11.~~30. Thorarinsdottir KA, Arason S, Geirsdottir M, Bogason SG, Kristbergsson K. Changes in myofibrillar proteins during processing of salted cod (*Gadus morhua*) as determined by electrophoresis and differential scanning calorimetry. Food Chem. 2002; 77(3):377–385. doi: 10.1016/S0308-8146(01)00349-1.

~~12.~~31. FSSA. Fish and fish products regulations. In: Gaur VN, editors. Food Safety and Standards Regulation Authority (FSSA). New Delhi: The gazette of India, 2011. pp. 366-369.

~~13.~~32. Sannaveerappa T, Ammu K, Joseph J. Protein-related changes during salting of milkfish (*Chanos chanos*). J Sci Food Agric. 2004; 84(8):863–869. doi: 10.1002/jsfa.1682.

~~14.~~33. Marínez-Alvarez O, Gómez-Guillén MC. The effect of brine composition and pH on the yield and nature of water-soluble proteins extractable from brined muscle of cod (*Gadus morhua*). Food Chem. 2005; 92(1):71–77. doi: 10.1016/j.foodchem.2004.04.049.

~~15.~~34. Ockerman HW, Basu L. Preservation methods of animal products. In: Dikeman M, Devine C, editors. Encyclopedia of Meat Sciences. London: Oxford Elsevier Ltd.; 2014. pp. 78–83. doi: 10.1016/B978-0-12-384731-7.00207-5.

~~16.~~35. Kim JC, Mullan BP, Pluske JR. Prediction of apparent, standardized, and true ileal digestible total and reactive lysine contents in heat-damaged soybean meal samples. J Anim Sci. 2012; 90:137–139. doi: 10.2527/jas.53819.

~~17.~~36. Lantry BF, O'Gorman R. Drying temperature effects on fish dry mass measurements. J Great Lakes Res. 2004; 33:606–616. doi: 10.3394/0380-1330(2007)33[606:DTEOFD]2.0.CO;2.

~~18.~~37. Bastos JR. Influência da secagem sobre algumas propriedades físico-químicas do músculo do cação branco, Carcharhynus porosus Ranzani (in portuguess, with

english abstract). Arquivos de Ciências do Mar. 1997; 17:77–87. Available from: http://www.repositorio.ufc.br/handle/riufc/1403

~~19.~~**38.** Pacheco N, Garnica-Gonzalez M, Gimeno M, Bárzana E, Trombotto S, David L, et al. Structural characterization of chitin and chitosan obtained by biological and chemical methods. Biomacromolecules. 2011; 12:3285–3290. doi: 10.1021/bm200750t.

~~**20.** Vose RS, Easterling DR, Gleason B. Maximum and minimum temperature trends for the globe: An update through 2004. Geophys Res Lett. 2005; 32:1–5. doi: 10.1029/2005GL024379.~~

~~**21.** Fiúza LS, Aragão NMS, Ribeiro-Junior HP, Moraes MG, Rocha IRCB, Lustosa-Neto AD, et al. Effects of salinity on the growth, survival, haematological parameters and osmoregulation of Tambaqui Colossoma macropomum juveniles. Aquac Res. 2015; 46:1–9. doi: 10.1111/are.12224.~~

~~**22.** Saint-Paul U. Native Fish species boosting Brazilian's aquaculture development. Acta Fish Aquat Res. 2017; 5:1–9. doi: 10.2312/ActaFish.2017.5.1.1-9.~~

39. Frisso RM, de Matos FT, Moro GV, de Mattos BO. Stocking density of Amazon fish (*Colossoma macropomum*) farmed in a continental neotropical reservoir with a net cages system. Aquaculture. 2020; 529:p.735702. doi: 10.1016/j.aquaculture.2020.735702

~~23.~~**40.** Lewbart GA. Euthanasia of ectotherms. In: Fowler's zoo and wild animal medicine current therapy. Elsevier. 2019; 9:357–363. doi: 10.1016/b978-0-323-55228-8.00051-5.

~~24.~~**41.** AOAC. Official Methods of Analysis of the Association of Official Analytical Chemists. 16th ed. Arlington VA; 1995.

~~25.~~**42.** AOAC. Official Methods of Analysis of the Association of Official Analytical Chemists. 17th ed. Washington DC: Gaithersburg publishing; 2001.

Field Code Changed

Formatted: Portuguese (Brazil)

Formatted: Font: Italic

~~26.~~43. AOAC. Official Methods of Analysis of the Association of Official Analytical Chemists. 15th ed. Arlington VA; 1990.

~~27.~~44. ISO. Meat and Meat Products - Determination of total ash. In: International Organization for Standardization. Switzerland: ISO/936; 1998. Available from: https://www.iso.org/standard/24783.html

~~28.~~45. Gorsuch TT. The Destruction of organic matter. 1st ed. New York: Oxford pergamon press Ltd; 1970. pp. 40–54.

~~29.~~46. Hulshof TG, Bikker P, Van der Poel AFB, Hendriks WH. Assessment of protein quality of soybean meal and 00-rapeseed meal toasted in the presence of lignosulfonate by amino acid digestibility in growing pigs and Maillard reaction products. J Anim Sci. 2016; 94:1020–1030. doi: 10.2527/jas.2015-9700.

~~30.~~47. Moughan PJ, Gall MPJ, Rutherfurd SM. Absorption of lysine and deoxyketosyllysine in an early Maillard browned casein by the growing pig. J Agric Food Chem. 1996; 44:1520–1525. doi: 10.1021/jf950428v.

~~31. Li Y, Xue C, Quan W, Qin F, Wang Z, et al. Assessment the influence of salt and polyphosphate on protein oxidation and Nε-(carboxymethyl)lysine and Nε-(carboxyethyl)lysine formation in roasted beef patties. Meat Sci. 2021; 177:108489. doi: 10.1016/j.meatsci.2021.108489.~~

~~32. Soladoye OP, Juárez ML, Aalhus JL, Shand P, Estévez M. Protein oxidation in processed meat: Mechanisms and potential implications on human health. Compr Rev Food Sci Food Saf. 2015; 14:106–122. doi: 10.1111/1541-4337.12127.~~

~~33.~~48. Gill TA, Chan TK, Phozchareon KF, Paulson AT. Effect of salt concentration and temperature on heat-induced aggregation and gelation of fish myosin. Food Res Int. 1992; 25:333–341. doi: 10.1016/0963-9969(92)90108-H.

~~34.~~49. Stoknes IS, Walde PM, Synnes M. Proteolytic activity in cod (*Gadus morhua*) muscle during salt curing. Food Res Int. 2005; 38:693–699. doi: 10.1016/j.foodres.2005.02.003.

~~35.~~50. Geda F, Declercq AM, Remø SC, Lourenço RWM, Janssens GPJ. The metabolic response in fish to mildly elevated water temperature relates to species-dependent muscular concentrations of imidazole compounds and free amino acids. J Therm Biol. 2017; 65:57-63. doi: 10.1016/j.jtherbio.2017.02.004.

~~36.~~51. Zhang Y, Dabrowski K, Hliwa P, Gomulka P. Indispensable amino acid concentrations decrease in tissues of stomachless fish, common carp in response to free amino acid- or peptide-based diets. Amino Acids. 2006; 31(2):165–172. doi: 10.1007/s00726-006-0345-7.

~~37.~~52. Chesney RW, Helms RA, Christensen M, Budreau AM, Han X, Sturman JA. The role of taurine in infant nutrition. Adv Exp Med Biol. 1998; 442:463–476. doi: 10.1007/978-1-4899-0117-0_56.

~~38.~~53. Alzawqari MH, Al-Baadani HH, Alhidary IB, Al-Owaimer AN, Abudabos AM. Effect of taurine and bile acid supplementation and their interaction on performance, serum components, ileal viscosity and carcass characteristics of broiler chickens. S Afr J Anim Sci. 2016; 46(4):448–457. doi: 10.4314/sajas.v46i4.13.

~~39.~~54. Boldyrev AA, Aldini G, Derave W. Physiology and pathophysiology of carnosine. Physiol Rev. 2013; 93:1803–1845. doi: 10.1152/physrev.00039.2012.

~~40.~~55. Shiau CY, Pong YJ, Chiou TK, Tin YY. Effect of starvation on free histidine and amino acids in white muscle of milkfish *Chanos chanos*. Comp Biochem Physiol B: Biochem Mol Biol. 2001; 128:501–506. doi: 10.1016/S1096-4959(00)00350-X.

~~41.~~56. Kopeć W, Jamroz D, Wiliczkiewicz A, Biazik E, Pudlo A, Hikawczuk T, et al. Influence of different histidine sources and zinc supplementation of broiler diets on

dipeptide content and antioxidant status of blood and meat. Br Poult Sci. 2013; 54:454–465. doi: 10.1080/00071668.2013.793295.

~~42.~~57. Kumazawa K, Seguro K, Takamura M, Motoki M. Formation of ε-(γ-glutamyl)lysine cross-link in cured horse mackerel meat induced by drying. J Food Sci. 1993; 58:1062–1064. doi: 10.1111/j.1365-2621.1993.tb06112.x.

~~43.~~58. Fedina IS, Georgieva K, Grigorova I. Light-dark changes in proline content of barley leaves under salt stress. Biol Plant. 2002; 45:59–63. doi: 10.1023/A:1015175802847.

~~44.~~59. Yankah MV, Ohshima T, Koizumi C. Effects of processing and storage on some chemical characteristics and lipid composition of a Ghanian fermented fish product. J Sci Food Agric. 1993; 63:227–235. doi: 10.1002/jsfa.2740630209.

~~45.~~60. Yamashita Y, Yamashita M, Iida H. Selenium Content in Seafood in Japan. Nutrients. 2013; 5:388–395. doi: 10.3390/nu5020388.

~~46.~~61. Polak-Juszczak L. Effects of processing methods on the content of minerals in fish products. J Elem. 2016; 21:461-470. doi: 10.5601/jelem.2015.20.3.890.

~~47.~~62. Bakhiet HHA, Khogali FAH, Ahmed FAM. Effect of different salt concentrations on mineral content of the fish (*Hydrocynus* spp.). J Life Sci Biomed. 2013; 3(1):01–03.

~~48.~~63. Pask F, Lake P, Yang A, Tokos H, Sadhukhan J. Industrial oven improvement for energy reduction and enhanced process performance. Clean Technol Environ Policy. 2017; 19:215-224. doi: 10.1007/s10098-016-1206-z.

Response to Reviewers

Comment	Response	Adaptation	Line/s
**Reviewer #1**			
1. Is the manuscript technically sound, and do the data support the conclusions?<br>*Yes	Thank you for your comments.	-	-
2. Has the statistical analysis been performed appropriately and rigorously?<br>*No<br>GENERAL COMMENTS<br>The paper is well written with strong justification and very good elaboration in methods. However some of the sections such as data analysis is not well elaborated. The types of statistics used needs to be appropriate to answers the objective of the research.	Comment acknowledged. We now have revised the ‘statistical analysis’ section and narrated it in line with our findings.<br>Further statistical elaborations have also been included in the ‘Results’ section under individual Tables 1, 2, and 3 and with the PCA plots as well.	Data obtained in the current study were analyzed using SPSS statistical software version 27 (IBM Corporation, Armonk, NY, USA). All data were checked for normality distributions using histogram and QQ-plots. A univariate one-way ANOVA was used with brining as fixed factor and temperature as covariate to test the hypothesis about differences between two or more mean nutrient values. Data were presented as means and standard error of the means (SEM). Statistical significance was set at $P < 0.05$ for any differences observed between samples. Furthermore, multivariate analysis was computed using principal component analysis (PCA) on the collective factors obtained to appraise the relationships of nutrient profiles based on their responses towards brining and drying methods, with values deemed relevant	265-274

		below -0.5 and above 0.5 of the components' values.	
		Kindly, see for footnotes under Tables 1, 2, 3 and Figure 2.	293, 303, 315, 337
3. Have the authors made all data underlying the findings in their manuscript fully available?<br>*No	Comment acknowledged. We now have reviewed our data availability statement and managed to include and upload the extra results and the datasets as 'supplementary information' files.	- Kindly, see for 'data availability statement corrected.<br>- Kindly, see for the data and extra results annexed as supplementary files with the journal.	-
4. Is the manuscript presented in an intelligible fashion and written in standard English?<br>*Yes	Thank you for your comments.	-	-
**5. Review Comments to the Author:**			
OTHER COMMENTS:			
Abstract<br>In the abstract introduce the concept of brine salting is what, before "The present study evaluated….."	Comment accepted. We now have addressed this narration in the abstract section.<br>The abstract is also rewritten again to meet other reviewers suggestions as well.	Preservation of fish as diet ingredient is challenging in many tropical conditions due to the absence of freezing facilities, hence warranting other preservation techniques. Brine salting is one of the oldest traditional and economically viable preservation methods. The present study evaluated the effect of…	17-20
**Introduction:**<br>Line 93: You could add "macronutrients and micronutrients"	Comment accepted. We now have corrected the sentence.	The present study therefore evaluated the effect of brining and a range of drying temperatures on the	133-135

		macronutrients and micronutrients, and mineral composition as well as on the state of Maillard reaction that may reduce lysine availability.	
**Materials and Methods:**			
Line 97-104: The paragraph needs to be cited.	Comment accepted. We have now put a suitable referenced citation at the end of the paragraph.	All experimental and animal care procedures were realized based upon the current written knowledge of aquaculture technologies and tambaquí fish farming. Fingerlings of Colossoma macropomum were initially brought from Aqua-Koi NV farm (http://www.aqua-koi.be/documents/home.xml?lang=nl) to TRANSfarm and reared in a recirculating aquaculture system (RAS) with a weekly basis 30% water renewal (20L/day) for about 7 months period before being subject to the current study. During their stay, fish were kept in larger aqua-tanks (100 cm × 120 cm × 40 cm) at a density of 1.2 fish/m3 sufficient enough for movements and behavioral expression. Tanks were fixed with individual bio-filters, well aerated with a constant temperature supply, 18:6 h light - dark cycle, and supplying pipes were regularly inspected and cleaned with tap water and water quality parameters were	138-148

		checked twice a week and water renewed accordingly [39].	
Line 120: A total of 48 tambaqui fish (792 ± 16 g; 8 months old). This number is not clear, whether say it mean weight (sd) or provide unit explanation.	Comment accepted. We now have rewritten the sentence accordingly.	A total of 48 tambaqui fish with a mean weight of 792 (± 16) g and 8 months old were collected from TRANSfarm…	164-165
Statistical analysis:<br>Line 213-215: Univariate analysis is done for each separate variable in a descriptive way. Here did you mean bivariate analysis for comparing variance? Please clarify	Comment acknowledged. Here we took brining as fixed factor and temperature as covariate to test our hypothesis about differences between variables. Hence, univariate was done for each separate variable.	Kindly, see statistical analysis section.	
Line 264: Multivariate statistical analysis (PCA), revise either write PCA on remain with multivariate.<br>Multivariate classification of variables: This section is not well written and confusing. Which association did the author is presenting? PCA is not showing the association between variables. If multivariate analysis was used which type of statistical technique was applied. Just to say the association is not enough, it has to be presented with values. What makes you say this is association/no association? Please clarify.	Comment accepted. We have revised this section and added on the eigenvalues for the components most explaining.<br>Also, added the graph of PCA which now could explain the narrations stated more.	Result section:<br>Principal component analysis was computed on the collective factors obtained to appraise the relationships of nutrient profiles based on their responses towards brining and drying methods. By examining at the scree plot and eigenvalues and looking for a point at which the proportion of variance explained by each subsequent principal component drops off, an elbow in the screen plot, the most explaining two components 1 (44.8%) and 2 (18.9%) were considered and a biplot PCA generated.<br>In line, there were…	320-325

Line 215: "All data were presented as means of three values and standard error of the means (SEM)". Is not clear, What does means of three value imply for?	Comment accepted. By 'means of three values', we meant that there were three fish samples evaluated per treatment group. We now have corrected this in the statistical analysis section, and narrated it clearly under each Table as footnotes.	Data were presented as means and standard error of the means (SEM). Statistical significance…	268-269
		Kindly, see for footnotes under Tables 1, 2, and 3.	293, 303, 315
Where are the results of Principal Component Analysis (PCA)? Why did the researcher opt to use PCA in this research?	Comment acknowledged. The results of PCA are displayed in Figure 2 clearly.<br>We now have included the PCA plot which was initially submitted separately.<br>We carried out PCA so as to evaluate the associations between the nutrient profiles on the basis of their response to brining and drying methods. We have tried to elaborate this under the 'Statistical analysis' and 'Results' as well.	Kindly, see for Figure 2. Principal component analysis classification of nutrient profiles…..	334-341
		Furthermore, principal component analysis (PCA) was computed using on the collective factors obtained to appraise the relationships of nutrient profiles based on their responses towards brining and drying methods, with values deemed relevant below -0.5 and above 0.5 of the components' values.	270-273
		Principal component analysis was computed on the collective factors obtained to appraise the relationships of nutrient profiles based on their responses towards brining and drying methods.	320-325
**Results:**			
You could better start the results section by provide a small descriptive analysis of the samples and general information about your data.	Comment accepted. We now have added a paragraph as a start of the 'Result' section.	Tambaqui fish samples were grouped into two as brine-salted and non-salted, and treated at seven different drying temperatures for 23 h each.	275-278

		Each temperature treatment was considered as sampling point and tested in triplicate (n = 3) and mean values obtained were statistically computed for all nutrient profiles evaluated.	
Line 227: the title of Fig 1 is there, but figure is not shown. Place the figure header below the figure.	Comment accepted. We now have added the Figure and place the figure header below it.	Kindly, see for Figure 1.	282-285
Table 1: The table generalizes the values of macronutrients at different temp, could you try and present at each temp and we could see the variations?	Comment accepted. Such Table was not included in our manuscript as we feel it looks bulky and not easy to follow with seven different values for each preservation method and there was no statistical difference between temperature points.<br>The no significant effect of individual temperatures is represented in Figure 1 by taking representative nutrients and how they look with temperature points.<br>We now have tried to make a separate Table with all the values at different temperature points. This is now addressed as a "Supplementary files" due to larger size of the Table.	Kindly, see for supplementary files annexed.	-
		Kindly, see for Figure 1. **Fig 1.** The effect of drying temperature on nutrient profiles of tambaqui fish. (a) gram of taurine per kg fish; (b) gram of reactive lysine per kg fish.	282-285

<table>
<tr><td rowspan="3">Table 1 needs to be improved, I can only see the values during salting, but not drying temp.</td><td rowspan="3">Comment accepted. In the column values, the terms yes and no refer to salted and drying temp, respectively. We agree that the naming were confusing and now we have corrected this in all Tables 1, 2, and 3 and make it clearer. As mentioned higher, the data combining all salting and drying treatments are presented in Supplementary files because of the complexity.</td><td>Kindly, see for naming corrections made in Tables 1.</td><td>290-294</td></tr>
<tr><td>Kindly, see for naming corrections made in Tables 2.</td><td>300-305</td></tr>
<tr><td>Kindly, see for naming corrections made in Tables 3.</td><td>312-318</td></tr>
<tr><td rowspan="3">Label below the table have to be clearly presented such as DM=Dry matter.</td><td rowspan="3">Comment accepted. We now have corrected the labels under all Tables 1, 2, and 3.</td><td>Table 1 label:
DM= Dry matter; SEM= standard error of the means; temp= temperature.
Values represent mean and standard error of the means based on three replicates per treatment and seven treatments; n = 48.</td><td>292-294</td></tr>
<tr><td>Table 2 label:
DM= Dry matter; SEM= standard error of the means; temp= temperature.
Values represent mean and standard error of the means based on three replicates per treatment and seven treatments; n = 48.
*Values are presented in µg/kg DM.</td><td>302-305</td></tr>
<tr><td>Table 3 label:</td><td>314-318</td></tr>
</table>

		CP= Crude protein; AA= Amino acid; SEM= Standard error of the means; temp= temperature.<br>Values represent mean and standard error of the means based on three replicates per treatment and seven treatments; n = 48.<br>[a]Percentage of reactive lysine in the total protein.<br>[b]Percentage of reactive lysine in the total lysine.	
Line 239: The essential “microminerals”. Just say micronutrients or minerals	Comment accepted. We now corrected the sentence.	The essential minerals Se and I, and the…….	296
Table 2: Similar to table 1, different temp values needs to be presented in table	Comment accepted. We now have added a separate additional Table for proximate and minerals values at individual temperature points as a ‘supplementary files’.	Kindly, see for annexed ‘supplementary files’.	-
**Discussion:**			
Start by providing the objective the research and summarizing the main findings.	Comment accepted. We now have added a paragraph involving the objectives and summary findings as a start.	The present study evaluated the effect of brine salting (15% *w/w*) prior to drying at different temperatures on the nutrient profiles of tambaqui fish. Brining induced a prominent effect on macronutrient and micronutrient profiles of tambaqui fish.<br>In this study, brining induced pronounced losses….	343-345
Line 283: In our study. Use “In this study”		In this study, brining induced…	346

	Comment accepted. Such sentences are now corrected here and across the manuscript.	The amino acid analysis in this study demonstrated…	361
How these literature compared here related to Tambaqui fish? Or related to included sample? Compare and contrast with relevant papers/research.	Comment accepted. We now have removed the unrelated literature contents and their references (two) and focused on the relevant ones only.	In this study, brining induced pronounced losses of specific amino acids and minerals, irrespective of drying temperature. Gill et al. [48] found that salting enhanced the aggregation of myosin in cod, yet only above a drying temperature of 50°C. Marínez-Alvarez and Gómez-Guillén [33] indicated that there is a notable loss of soluble muscle proteins (particularly, actin and myosin) by osmosis in salting (NaCl) at pH 6.5 on cod muscle (*Gadus morhua*).	346-350
Line 292: more than 60C. You don't have that temp in your study. The final temp is 60 Centigrade.	Comment accepted. We now have removed this sentence from the 'discussion' paragraph.	Marínez-Alvarez and Gómez-Guillén [33] indicated that there is a notable loss of soluble muscle proteins (particularly, actin and myosin) by osmosis in salting (NaCl) at pH 6.5 on cod muscle (*Gadus morhua*). Differences between studies can evolve from…	348-351
Line 298: it is clear that brining completely overruled the drying temperature in its effect on nutrient loss. Just use the term such as " This study suggests that brining is associated with nutrients loss…..".	Comment accepted. We now have rewritten the sentence accordingly.	Although comparison with existing literature does not allow us to pinpoint the factors that protected from a drying temperature effect, this study suggests that brining is associated with nutrient losses.	353-355

Line 299: 6% of tissue protein. Focus on what you measure "Crude protein"	Comment accepted and wording now corrected.	In the present study, a total of 6% crude protein was lost due to the brining stress.	355-356
The limitation and strength of this research is very important to be stated in this research.	Comment accepted. We now have clearly addressed the study limitation at the end of the revised 'conclusion' section.	…Therefore, drying without brining may better preserve the nutritive value of tambaqui fish. However, as a recommendation to the industry sector, it is suggested that end products are further evaluated for any pathogen of economic or public health importance.	439-441
**Conclusion:**			
Conclusion should be straight and clear. Please consider revising. Example, this research shows that ……	Comment accepted. By now, we have revised and rewritten the conclusion paragraph and tried to make it clearer and straight.	This research shows that drying temperature has no effect on nutrient composition of the studied fish which could be encouraging for drying of fish under – less controlled – rural tropical conditions. Brining, however, reduced some important trace elements such as Se and I, and lead to loss of muscle crude protein, with the concomitant amino acids. Brining also increased the leaching of K, I, and taurine. The formation of Maillard products was not promoted by both drying and brining treatments, as demonstrated through unaltered reactive lysine proportions. Therefore, drying without brining may better preserve the nutritive value of tambaqui fish. However, as a recommendation to the industry	433-441

		sector, it is suggested that the final products are further evaluated for any pathogen of economic or public health importance.	
**Reviewer #2**			
1. Is the manuscript technically sound, and do the data support the conclusions?<br>*Yes	Comment acknowledged.	-	-
2. Has the statistical analysis been performed appropriately and rigorously?<br>*Yes	Thank you for your comments.	-	-
3. Have the authors made all data underlying the findings in their manuscript fully available?<br>*Yes	Thank you for your comments.	-	-
4. Is the manuscript presented in an intelligible fashion and written in standard English?<br>*Yes	Thank you for your comments.	-	-
**5. Review Comments to the Author:**			
Line 146. Please specify the model of oven used and Humidity condition set for laboratory simulation.	Comment accepted. We now have added the oven model and laboratory humidity as well.	Each tray was placed in an electric oven (EP66, BEKSO-SA®, Belgium) and randomly set and dried with the above-mentioned temperature points. The laboratory humidity was in a range of 50-60% during the experimental period.	190-192
Line 153. The statement (Samples were finally freeze-dried) looks contradictory with the title "A laboratory simulation of the impact of brining and drying as accessible preservation	Comment acknowledged. In fact, it is the drying oven used to simulate the tropical environment. Once all samples	Impact of brining and drying processes on the nutritive value of tambaqui fish (*Colossoma macropomum*)	1-2

strategies under tropical rural conditions on the nutritive value of tambaqui fish (*Colossoma macropomum*)".	has undergone salting and/or drying, they were finally kept at -21ºC until evaluated. As part of a protocol for analyzing nutrients, frozen samples were equally freeze dried, homogenously ground and used to determine nutrient concentrations.<br>However, seeing the importance of the comment, we now have changed the TITLE of the manuscript and elaborated this step clearly in the methodology section as well.	Samples were then kept at -21°C overnight and finally freeze-dried (CoolSafe™, SCANVAC, Denmark) for a period of 16 h each to a stable weight, uniformly ground (2.0 mm sieve), and homogenized using a grinder machine (De´Longhi™, KG48) at 12× speed and then individually kept in closable plastic containers at 4°C until analyzed [22].	197-201
Line 376. The language of the conclusion must be improved for better understanding of the reader. This section must be sound.	Comment accepted. By now, we have tried to rewrite the conclusion paragraph and make it more clear and acceptably sound. We have also addressed the future focus/limitation of the study in response to reviewer 1 and editor comments.	This research shows that drying temperature has no effect on nutrient composition of the studied fish which could be encouraging for drying of fish under – less controlled – rural tropical conditions. Brining, however, reduced some important trace elements such as Se and I, and lead to loss of muscle crude protein, with the concomitant amino acids. Brining also increased the leaching of K, I, and taurine. The formation of Maillard products was not promoted by both drying and brining treatments, as demonstrated through unaltered reactive lysine proportions. Therefore, drying without brining may better	432-440

		preserve the nutritive value of tambaqui fish. However, as a recommendation to the industry sector, it is suggested that the final products are further evaluated for any pathogen of economic or public health importance.	
**Reviewer #3**			
1. Is the manuscript technically sound, and do the data support the conclusions?<br>*Yes	Comment acknowledged.	-	-
2. Has the statistical analysis been performed appropriately and rigorously?<br>*Yes	Thank you for your comments.	-	-
3. Have the authors made all data underlying the findings in their manuscript fully available?<br>*Yes	Thank you for your comments.	-	-
4. Is the manuscript presented in an intelligible fashion and written in standard English?<br>*Yes	Thank you for your comments.	-	-
5. Review Comments to the Author:			
This is a good work and needs attention of the researchers. I must recommend it to be published.	Thank you for your comments.	-	-
Comments to editor:<br>In the current manuscript the authors have studied "A laboratory simulation of the impact of brining and drying as accessible preservation strategies under tropical rural conditions on the nutritive value of tambaqui fish". This is a good research work and need of the hour. I recommend this work to be published in your esteemed journal.	Thank you for the valuable feedback and comments given.	-	-
**Academic Editor (EA):**			

There is no simulation in this study, please change the title to Impact of brining and drying processes...............	Comment accepted. We now have changed the title.	Impact of brining and drying processes on the nutritive value of tambaqui fish (*Colossoma macropomum*)	1-2
You have mentioned that electricity is a problem to rural communities of most tropical regions, using electric oven for your experiment should not be an accessible preservative strategies	Comment accepted. We now have corrected the title and removed this sentence from the 'Introduction' section and the objectives as well.	Removed sentence: Freezing is an efficient manner for fish preservation, but in many rural areas of the Global South, freezing fish is not an option because of poor electricity supply and the unaffordable cost of freezers.	38-48
Please add the effect brining on the nutrient profile and at what significant level?	Comment accepted. We now have added the effect of brining together with that of drying effect.	Drying temperature had no effect on the nutrient profiles of tambaqui fish ($P > 0.05$) while brining reduced the overall protein concentration by 6% (58.8 to 55.4 g/100 g DM; $P = 0.004$).	24-26
I will suggest that you re-write your results section of the abstract: Present the ANOVA results on all the nutrient profile and the interactive effect of brining and drying processes (if it was done).<br>Follow by the mean separation results for the key nutrient that had significant effect.<br>PCA result	Comment accepted. We now have rewritten the ENTIRE abstract with the suggestions given. We apologize that we could not manage to include the interactive effect as stated.	Preservation of fish as diet ingredient is challenging in many tropical conditions due to the absence of freezing facilities, hence warranting other preservation techniques. Brine salting is one of the oldest traditional and economically viable preservation methods. The present study evaluated the effect of brine salting (15% *w/w*) prior to drying at different temperatures on the nutrient profiles of tambaqui fish (*Colossoma macropomum*). Whole fish samples (792 ± 16 g; 8 months old; n = 48) were grouped into two as brine-salted and non-salted, and treated at seven different drying temperatures of 30,	16-41

		35, 40, 45, 50, 55 and 60°C for a period of 23 h each. To evaluate the impact of Maillard reaction, reactive lysine was also quantified. Drying temperature had no effect on the nutrient profiles of tambaqui fish (*P > 0.05*) while brining reduced the overall protein concentration by 6% (58.8 to 55.4 g/100 g DM; *P = 0.004*). Brining significantly diminished many amino acids taurine by 56% (7.1 to 3.1 g/kg; *P < 0.001*), methionine 17% (14.7 to 12.1 g/kg; *P < 0.001*), cysteine 11% (5.1 to 4.4 g/kg, *P = 0.016*), and reactive lysine 11% (52.0 to 46.4 g/kg; *P = 0.004*). However, alanine, arginine, and serine were not affected by brining (*P > 0.05*). Brining also reduced the concentrations of Se by 14% (149 to 128 µg/kg dry matter; *P = 0.020*), I 38% (604 to 373 µg/kg DM; *P = 0.020*), K 42% (9.71 to 5.61 g/kg DM; *P < 0.001*) and Mg 18% (1.32 to 1.10 g/kg DM; *P = < 0.001*) versus an anticipated vast increase in Na by 744% (2.70 to 22.90 g/kg DM; *P < 0.001*) and ash 28% (12.4 to 16.0 g/100g DM; *P < 0.001*) concentration. Neither brining nor drying temperature induced changes in lysine reactivity and fat content of tambaqui	

		fish (*P > 0.05*). Agreeably, results of multivariate analysis showed a negative association between brining, Na, and ash on one side of the component and most other nutrients on the other while the lack of effect of temperature can be seen by its presence in the center. Therefore, drying without brining may better preserve the nutritive value of tambaqui fish. However, as a practical to the industry sector, it is recommended that the final products are further evaluated for any pathogen of economic or public health importance.	
Please add the a sentence on the practical application of your findings.	Comment accepted. We now have addressed this at the end of the 'abstract' section.	However, as a practical to the industry sector, it is recommended that the final products are further evaluated for any pathogen of economic or public health importance.	39-41
Your introduction is not flowing well.<br>Following the general introduction on fish. please add the following:<br>- Explain the importance of studying the nutrient profiles in fish.<br>- Provide information on the natural habitat and characteristics of the studied fish<br>- Highlight the nutritional importance of this fish species for human consumption<br>-Then, Discuss the drying process and its role in enhancing the shelf life of fish	Comment accepted. We now have rewritten the entire 'Introduction' section and made the flow as per the suggestion given here with.	Kindly see for the ENTIRE Introduction section on the revised manuscript with tracked changes.	44-135

- A brief literature review on various traditional fish preservatives eg, sundrying, smoking, brinning,etc,<br>Then , Brining as yiu have in Line2s 42 to 60,<br>Drying as you have it in Lines 61 to 81.			
This is not true, There are several studies on brining and drying of fish. What makes your study unique is species you are using. Please check the following papers as examples:<br>-Kituu, G. M., Shitanda, D., Silayo, V., Odote, P., & Bongyereire, J. (2008). Effect of brining on the drying rate of tilapia (Oreochromis niloticus) in a solar tunnel dryer.<br>-Abraha, B., Admassu, H., Mahmud, A., Tsighe, N., Shui, X. W., & Fang, Y. (2018). Effect of processing methods on nutritional and physico-chemical composition of fish: a review. MOJ Food Process Technol, 6(4), 376-82.<br>-Guizani, N., Rahman, M. S., Al-Ruzeiqi, M. H., Al-Sabahi, J. N., & Sureshchandran, S. (2014). Effects of brine concentration on lipid oxidation and fatty acids profile of hot smoked tuna (Thunnus albacares) stored at refrigerated temperature. Journal of Food Science and Technology, 51, 577-582.<br>-Sampels, S. (2015). The effects of processing technologies and preparation on the final quality of fish products. Trends in Food Science & Technology, 44(2), 131-146.<br>-Doe, P., & Olley, J. (2020). Drying and dried fish products. In Seafood (pp. 125-145). CRC Press	Comment acknowledged. We now have rephrased the sentence in the 'Introduction' section.<br>Also, we have referenced and cited these and other references in the introduction as well.	Despite the effect of brining and drying temperatures on the nutrient profiles of different fish species has been investigated, we hypothesized that species differences in nutrient profiles could respond differently to drying and brining and evaluated tambaqui fish. Our study also…	124-126
		Kindly, see for about 15 extra references cited in the revised 'introduction' section.	44-135
Please you are simulating you are just checking the impact of brining and drying processes. Also, the rural conditions was not simulated using electric oven in the lab. Rural areas, they used sun drying. If you used sun drying or smoking processes, you will surely get a different result. I would have suggested you used sun drying, smoking as control	Comment accepted. We now have removed the term simulation word from this sentence in the introduction and corrected the study objectives in line with the new title. similarly	The present study therefore evaluated the effect of brining and a range of drying temperatures on the macronutrients and micronutrients, and mineral composition as well as on the state of Maillard reaction that may reduce lysine availability.	133-135

	from the methodology as well it has been removed.<br>We agree that results could be different and acknowledge your suggestion on the use of sun drying or smoking as a control, however, the experiment was solely done in Belgium where less sun exposures happen that we manage to use only electric oven.		
Imported from where?	Comment accepted. We now have corrected the wording and included the place fingerlings were brought.	Fingerlings of *Colossoma macropomum* were initially brought from Aqua-Koi NV farm (http://www.aqua-koi.be/documents/home.xml?lang=nl) to TRANSfarm and reared…	139-141
Using which method? Please add more information on the rearing process.	Comment accepted. We now have added details on the rearing process.	Fingerlings of *Colossoma macropomum* were initially brought from Aqua-Koi NV farm (http://www.aqua-koi.be/documents/home.xml?lang=nl) to TRANSfarm and reared in a recirculating aquaculture system (RAS) with a weekly basis 30% water renewal (20L/day) for about 7 months period before being subject to the current study. During their stay, fish were kept in larger aqua-tanks (100 cm × 120 cm × 40 cm) at a density of 1.2 fish/m^3 sufficient enough for movements and behavioral	139-148

		expression. Tanks were fixed with individual bio-filters, well aerated with a constant temperature supply, 18:6 h light - dark cycle, and supplying pipes were regularly inspected and cleaned with tap water and water quality parameters were checked twice a week and water renewed accordingly [39].	
Please add reference	Comment accepted. We now have added a reference to this.	…twice a week and water renewed accordingly [39].	148
What type of anesthetic agent?, give the brand, manufacturer, country	Comment accepted. We now have added this.	Each group of six unconscious fish was scooped out of the tank and brought to the slaughter room in the bucket with the anesthetic agent (1mL:5L, Anest Fish©, Germany).	154-156
This is repetition. You have given the detail earlier	Comment accepted. We now have deleted this sentence.	with ice packs (Luxaplast, NORDIC®). Upon arrival, to facilitate…	167
Was it stainless?	Comment accepted. Indeed, it was, we now have added the word 'stainless' in this sentence.	…using a stainless post mortem saw (SC-PM/110, 254MM) and scalpel-blades…	168-169
Using tap water? An you carried out Fe and Zn analysis on the samples. How did you avoid contamination?	Comment accepted. This was a typo error and apologies for that. We indeed have used distilled water in the lab to wash the sliced fish. Now, we have corrected this.	…thoroughly washed using distilled water in pipes and then individually stored in plastic bags…	169-170
Why 15%?	Comment acknowledged. This was done based on a reference	Industrial table salt (99.9%, BDH-PROLABO®, EC) was used for	172-173

	that we manage to include it at the sentence end by now.	preparing the 15% (*w/w*) brine solution in the present study [24].	
Please add reference	Comment accepted and reference has been added.	Industrial table salt (99.9%, BDH-PROLABO®, EC) was used for preparing the 15% (*w/w*) brine solution in the present study [24].	172-173
How did select these temperatures, especially the interval of 5? Any reference?	Comment acknowledged. The intervals and limit were chosen based on several works done on fish brining/salting with a range of results obtained. We now have cited these references at the sentence end.	Uniformly sliced whole fish samples were grouped into two as brine-salted and non-salted, and treated at seven different drying temperatures of 30, 35, 40, 45, 50, 55 and 60°C for a period of 23 h each [23,24,31,37].	175-177
Add reference for this process	Comment accepted and reference has been added.	Brine-salted fish samples were immersed in brine solution (15% *w/w*) at a ratio of 3:1 of fish to salt solution [22,27]. For each sampling unit…	181-182
Please state the humidity conditions while simulating natural drying environment and specify the model of oven used??	Comment accepted. We now have added the oven model and laboratory humidity as well.	Each tray was placed in an electric oven (EP66, BEKSO-SA®, Belgium) and randomly set and dried with the above-mentioned temperature points. The simulated laboratory humidity was in a range of 50-60% during the experimental period.	190-192
This statement looks contradictory with the title "A laboratory simulation of the impact of brining and drying as accessible preservation strategies under tropical rural conditions on the nutritive value of tambaqui fish (Colossoma macropomum)"	Comment acknowledged. In fact. Once all samples has undergone salting and/or drying, they were finally kept at -21ºC until evaluated. As part of a protocol for analyzing nutrients, frozen samples were equally	Impact of brining and drying processes on the nutritive value of tambaqui fish (*Colossoma macropomum*)	1-2
		Samples were then kept at -21°C overnight and finally freeze-dried (CoolSafe™, SCANVAC, Denmark)	197-201

	freeze dried, homogenously ground and used to determine nutrient concentrations.<br>However, seeing the importance of the comment, we now have changed the TITLE of the manuscript and elaborated this step clearly in the methodology section as well.	for a period of 16 h each to a stable weight, uniformly ground (2.0 mm sieve), and homogenized using a grinder machine (De´Longhi™, KG48) at 12× speed and then individually kept in closable plastic containers at 4°C until analyzed [22].	
Please give the particle size or the sieve size used	Comment accepted. Sieve size used now added.	…a stable weight, uniformly ground (2.0 mm sieve), and homogenized…	199
No reference for the Brining and drying processes	Comment accepted. Reference no has been added.	…speed and then individually kept in closable plastic containers at 4°C until analyzed [22].	200-201
Please give full meaning of all the acronyms at first mentioned	Comment accepted. We have addressed this across the entire manuscript.	… The dry matter (DM) content of freeze-dried samples…	207
Please add quality control steps observed to avoid contaminations. Also, please give the Limit of detection, sensitivity, accuracy level etc of the ICP-OES used. Please give simple steps of estimating the values of the minerals using ICP-OES.	Comment accepted. We now have tried to addressed these comments as shown in the manuscript.	In brief, a gram of sample was weighed, placed into a porcelain crucible and oven-dried at 105°C for 3 h. Sample was then burnt on a hot plate and incinerated in a muffle furnace at 450°C for 16 h. Obtained ash was then moistened with a small amount of deionized water first and dissolved in 10 mL of 1:1 (*v/v*) solution of hydrochloric acid (HCl) and deionized water and evaporated to dry. Finally, the mix was re-dissolved in 10 mL of 1:9 (*v/v*) solution of HCl and deionized water, transferred into a	219-238

		50 mL flask, and then diluted several folds for optimal determination of individual mineral concentration using induction-coupled plasma optical emission spectrometry (ICP-OES) method as per the manufacturer’s procedures (Iris Intrepid II XSP, Thermo Fisher Scientific, Aalst, Belgium). So as to obtain an optimum concentration range for the atomic absorption spectrometric method, the concentrations of certain minerals were determined after fivefold (Mg and Ca), 10-fold (Na and Zn), and 50-fold (K) dilutions. Likewise, the concentration of selenium was determined by ISO method (ICP-MS: ISO 11885) and iodine with tetramethylammonium hydroxide extraction and iodine quantification (ICP-MS: ISO 11885) at the Laboratory of Chemical analysis, Ghent University, Belgium. As quality control protocols, visual inspection of pump tubing at regular intervals and replacing between samples are often made to increase the precision measurements. Similarly, regular inspection and cleaning of the injector tube, spread chamber and mobilizer, and cleaning of the widows	

		are done to avoid contamination and reduction of light intensity. Blank solutions with similar matrix were used to improve the measurement accuracy and the detection limit set was 1-100 ppb.	
How many sampling point?	Comment accepted. We addressed this in the manuscript.	…from each of the seven sampling points for defatting…	2496-247
What sieve size?	Comment accepted. We now have added the sieve size.	finely ground and homogenized (2.0 mm sieve size) using a mixer mill…	248
Please present all tables in an acceptable format. Check Journal guidelines	Comment accepted. We now have corrected the Tables according to the journal's guideline.	Kindly, see for Table 1	289-293
		Kindly, see for Table 2	299-304
		Kindly, see for Table 3	311-317
The language of the conclusion must be improved for better understanding of the reader. This section must be sound.<br>Please add the practical application limitation of this study, recommendations to the conclusion.	Comment accepted. We now have rewritten the conclusion section and added the limitation and future recommendations at the end.	This research shows that drying temperature has no effect on nutrient composition of the studied fish which could be encouraging for drying of fish under – less controlled – rural tropical conditions. Brining, however, reduced some important trace elements such as Se and I, and lead to loss of muscle crude protein, with the concomitant amino acids. Brining also increased the leaching of K, I, and taurine. The formation of Maillard products was not promoted by both drying and brining treatments, as demonstrated through unaltered	432-440

		reactive lysine proportions. Therefore, drying without brining may better preserve the nutritive value of tambaqui fish. However, as a recommendation to the industry sector, it is suggested that the final products are further evaluated for any pathogen of economic or public health importance.	
**Academic Editor:**			
The manuscript has been reviewed and requires a major revision; all Editor's and reviewers' comments should be carefully considered in order to improve its quality.	Thank you for the valuable comments. We have now maximally addressed the comments and concerns given by all the reviewers and editor as well.	Kindly, see detailed answers and replies given point-by-point above under each reviewer and below for the academic editor as well.	-
Please include the following items when submitting your revised manuscript:<br>• A rebuttal letter that responds to each point raised by the academic editor and reviewer(s). You should upload this letter as a separate file labeled 'Response to Reviewers'.<br>• A marked-up copy of your manuscript that highlights changes made to the original version. You should upload this as a separate file labeled 'Revised Manuscript with Track Changes'.<br>• An unmarked version of your revised paper without tracked changes. You should upload this as a separate file labeled 'Manuscript'.	Comment accepted. We now have made all the required amendments and re-submitted the three items as requested.	-	-
If you would like to make changes to your financial disclosure, please include your updated statement in your cover letter.	Comment acknowledged. No changes are made regarding financial disclosure.	-	-

Guidelines for resubmitting your figure files are available below the reviewer comments at the end of this letter.			
If applicable, we recommend that you deposit your laboratory protocols in protocols.io to enhance the reproducibility of your results.	Not applicable.	-	-
**Journal additional requirements:**			
1. Please ensure that your manuscript meets PLOS ONE's style requirements, including those for file naming. The PLOS ONE style templates can be found at: https://journals.plos.org/plosone/s/file?id=wjVg/ PLOSOne_formatting_sample_main_body.pdf and https://journals.plos.org/plosone/s/file?id=ba62/ PLOSOne_formatting_sample_title_authors_affiliations.pdf	Comment acknowledged. We now have re-visited the PLOS ONE style templates and addressed all the journal's requirements to the best of our knowledge in the revised version.	-	-
2. We note that you have indicated that data from this study are available upon request. PLOS only allows data to be available upon request if there are legal or ethical restrictions on sharing data publicly. For more information on unacceptable data access restrictions, please see http://journals.plos.org/plosone/s/data-availability#loc-unacceptabledata-access-restrictions.<br>In your revised cover letter, please address the following prompts:<br>a) If there are ethical or legal restrictions on sharing a de-identified data set, please explain them in detail (e.g., data contain potentially sensitive information, data are owned by a third-party organization, etc.) and who has imposed them (e.g., an ethics committee). Please also provide contact information for a data access committee, ethics committee, or other institutional body to which data requests may be sent.	Comment accepted. In fact, there are no legal or ethical restrictions to the data used in this study. Hence, by now, we have uploaded the dataset, output, and supplementary Table involved in the study as Supporting Information files'.	Kindly, see for uploaded supplementary files separately.	-

b) If there are no restrictions, please upload the minimal anonymized data set necessary to replicate your study findings as either Supporting Information files or to a stable, public repository and provide us with the relevant URLs, DOIs, or accession numbers. For a list of acceptable repositories, please see http://journals.plos.org/plosone/s/dataavailability#loc-recommended-repositories.<br>We will update your Data Availability statement on your behalf to reflect the information you provide.			
Additional Editor Comments:<br>The manuscript has been reviewed and requires a major revision; all Editor's and reviewers' comments should be carefully considered in order to improve its quality. Please check the uploaded files for the comments.	Comment accepted. We hope now have addressed all the comments and concerns raised by the reviewers and editor to the best of our knowledge.	Kindly, see for detailed responses and adaptations made point-by-point above under each reviewer and academic editor as well.	-
