# Supplementary material for: Impact of brining and drying processes on the nutritive value of tambaqui fish (Colossoma macropomum)
Source: PLoS One. 2024 Apr 16;19(4):e0299926. doi: 10.1371/journal.pone.0299926 (PMC11020844; doi:10.1371/journal.pone.0299926)
Supplement: Attachment — Submitted filename: PONE-D-22-35077_R2.pdf [file pone.0299926.s013.pdf]

PLOS ONE

Impact of brining and drying processes on the nutritive value of tambaqui fish (Colossoma macropomum)

--Manuscript Draft--

**Manuscript Number:**	PONE-D-22-35077R2
**Article Type:**	Research Article
**Full Title:**	Impact of brining and drying processes on the nutritive value of tambaqui fish (Colossoma macropomum)
**Short Title:**	Effect of brining and drying on nutritive value of tambaqui fish.
**Corresponding Author:**	Awot Teklu MEBRATU<br>Universiteit Gent<br>Merelbeke, BELGIUM
**Keywords:**	brining; drying; nutrient profiles; tambaqui; reactive lysine
**Abstract:**	Preservation of fish as diet ingredient is challenging in many tropical regions due to poor socioeconomic conditions and lack of freezing facilities. So, alternative preservation techniques could be viable to address the issue. The present study evaluated the effect of brine salting (15% w/w) prior to drying at different temperatures on the nutrient profiles of tambaqui fish (Colossoma macropomum). Whole fish samples (792 ± 16 g; 8 months old; n = 48) were grouped into two as brine-salted and non-salted, and treated at seven different drying temperatures of 30, 35, 40, 45, 50, 55 and 60°C for a period of 23 h each. To evaluate the impact of Maillard reaction, reactive lysine was also quantified. Drying temperature had no effect on the evaluated macro- and micro-nutrients of tambaqui fish (P > 0.05) while brining reduced the overall protein concentration by 6% (58.8 to 55.4 g/100 g DM; P = 0.004). Brining significantly reduced many amino acids: taurine by 56% (7.1 to 3.1 g/kg; P < 0.001), methionine 17% (14.7 to 12.1 g/kg; P < 0.001), cysteine 11% (5.1 to 4.4 g/kg, P = 0.016), and reactive lysine 11% (52.0 to 46.4 g/kg; P = 0.004). However, alanine, arginine, and serine were not affected by brining (P > 0.05). Brining also reduced the concentrations of Se by 14% (149 to 128 µg/kg DM; P = 0.020), iodine 38% (604 to 373 µg/kg DM; P = 0.020), K 42% (9.71 to 5.61 g/kg DM; P < 0.001) and Mg 18% (1.32 to 1.10 g/kg DM; P = < 0.001) versus an anticipated vast increase in Na by 744% (2.70 to 22.90 g/kg DM; P < 0.001) and ash 28% (12.4 to 16.0 g/100g DM; P < 0.001) concentration. Neither brining nor drying temperature induced changes in % lysine reactivity and fat content of tambaqui fish (P > 0.05). Agreeably, results of multivariate analysis showed a negative association between brining, Na, and ash on one side of the component and most other nutrients on the other component. In conclusion, drying without brining may better preserve the nutritive value of tambaqui fish. However, as a practical remark to the industry sector, it is recommended that the final product is further evaluated for any pathogen of economic or public health importance.
**Order of Authors:**	Awot Teklu MEBRATU
	Yohannes Tekle Asfaw
	Wouter Merckx
	Wouter Hendriks
	Geert Janssens
**Response to Reviewers:**	Dear Reviewers,<br>The round-2 comments given by the reviewers were highly valuable for the quality and clarity of our manuscript. Now, to the best of our knowledge, we have tried to incorporate all the comments given so far including gramatical and typographical corrections and we have responded to them point-by-point in a separate rebuttal letter annexed as 'Response to Reviewers'.<br>We really appreciate!
**Additional Information:**	
**Question**	**Response**

Powered by Editorial Manager® and ProduXion Manager® from Aries Systems Corporation

**Financial Disclosure**<br><br>Enter a financial disclosure statement that describes the sources of funding for the work included in this submission. Review the submission guidelines for detailed requirements. View published research articles from *PLOS ONE* for specific examples.<br><br>This statement is required for submission and **will appear in the published article** if the submission is accepted. Please make sure it is accurate.<br><br>**Unfunded studies**<br>Enter: *The author(s) received no specific funding for this work.*<br><br>**Funded studies**<br>Enter a statement with the following details:<br>• Initials of the authors who received each award<br>• Grant numbers awarded to each author<br>• The full name of each funder<br>• URL of each funder website<br>• Did the sponsors or funders play any role in the study design, data collection and analysis, decision to publish, or preparation of the manuscript?<br>• **NO** - Include this sentence at the end of your statement: *The funders had no role in study design, data collection and analysis, decision to publish, or preparation of the manuscript.*<br>• **YES** - Specify the role(s) played.<br><br>* typeset	The project was financially supported by the VLIR-UOS TEAM project under the title 'Win-win protein: reconciling fish protein generation and water quality management' [grant number ET2018TEA462A101] through Geert P.J Janssens and Luc De Meester; https://www.vliruos.be/en/home/1. The funders had no role in study design, data collection and analysis, decision to publish, or preparation of the manuscript. ATM received support in form of salary while doing the research activities.
**Competing Interests**<br><br>Use the instructions below to enter a competing interest statement for this submission. On behalf of all authors, disclose any competing interests that could be perceived to bias this work—acknowledging all financial support and any other relevant financial or non-financial competing interests.<br><br>This statement is required for submission	The authors have declared that no competing interests exist.

Powered by Editorial Manager® and ProduXion Manager® from Aries Systems Corporation

and **will appear in the published article** if the submission is accepted. Please make sure it is accurate and that any funding sources listed in your Funding Information later in the submission form are also declared in your Financial Disclosure statement.<br><br>View published research articles from *PLOS ONE* for specific examples.<br><br>**NO authors have competing interests**<br><br>Enter: *The authors have declared that no competing interests exist.*<br><br>**Authors with competing interests**<br><br>Enter competing interest details beginning with this statement:<br><br>*I have read the journal's policy and the authors of this manuscript have the following competing interests: [insert competing interests here]*<br><br>* typeset	
**Ethics Statement**<br><br>Enter an ethics statement for this submission. This statement is required if the study involved:<br><br>• Human participants<br>• Human specimens or tissue<br>• Vertebrate animals or cephalopods<br>• Vertebrate embryos or tissues<br>• Field research<br><br>Write "N/A" if the submission does not require an ethics statement.<br><br>General guidance is provided below. Consult the submission guidelines for detailed instructions. **Make sure that all information entered here is included in the Methods section of the manuscript.**	All experimental and animal care procedures were realized based upon the current written knowledge of aquaculture technologies and tambaquí fish farming. Fingerlings of Colossoma macropomum were initially brought from Aqua-Koi NV farm (http://www.aqua-koi.be/documents/home.xml?lang=nl) to TRANSfarm and reared in a recirculating aquaculture system (RAS) with a weekly basis 30% water renewal (20L/day) for about 7 months period before being subject to the current study. During their stay, fish were kept in larger aqua-tanks at 1.2 fish/m3. Tanks were fixed with individual bio-filters, aerated, temperature supplied, 18:6 h light - dark cycle, and supplying pipes were regularly inspected and cleaned with tap water and water quality parameters were checked twice a week and water renewed accordingly [39].<br>For our experimental study, after a 12 h fasting, a total of 48 tambaqui fish were randomly selected and kept in a separate tank and were euthanized in a two-step procedure [40]. Six fish were randomly taken at a time and placed in a tank with a volume of 240 L water into which 50 mL of phenoxyethanol-solution was added. After five minutes, 30 L of water were elevated out of the tanks and put into a large bucket. Another 6 mL of Anest Fish© was added to this bucket. Each group of six unconscious fish was scooped out of the tank and brought to the slaughter room in the bucket with the anesthetic agent. Thereafter, tambaqui were killed with a firm blow to the head, followed by fracturing the brainstem with a knife and then transported in ice cubes for the experiment [40]. The study was conducted in accordance with the Declaration of Helsinki and directive 2010/63/EU of the European parliament and of the council on the protection of animals used for scientific purposes, and the wellbeing of the cultured tambaqui fish before being subjected to the experiment was assessed and approved

Powered by Editorial Manager® and ProduXion Manager® from Aries Systems Corporation

**Format for specific study types**<br><br>**Human Subject Research (involving human participants and/or tissue)**<br>• Give the name of the institutional review board or ethics committee that approved the study<br>• Include the approval number and/or a statement indicating approval of this research<br>• Indicate the form of consent obtained (written/oral) or the reason that consent was not obtained (e.g. the data were analyzed anonymously)<br><br>**Animal Research (involving vertebrate animals, embryos or tissues)**<br>• Provide the name of the Institutional Animal Care and Use Committee (IACUC) or other relevant ethics board that reviewed the study protocol, and indicate whether they approved this research or granted a formal waiver of ethical approval<br>• Include an approval number if one was obtained<br>• If the study involved *non-human primates*, add *additional details* about animal welfare and steps taken to ameliorate suffering<br>• If anesthesia, euthanasia, or any kind of animal sacrifice is part of the study, include briefly which substances and/or methods were applied<br><br>**Field Research**<br><br>Include the following details if this study involves the collection of plant, animal, or other materials from a natural setting:<br>• Field permit number<br>• Name of the institution or relevant body that granted permission	by the institutional review board of TRANSfarm, KU Leuven, lab accreditation (KU/LA-1210616).
**Data Availability**<br><br>Authors are required to make all data underlying the findings described fully available, without restriction, and from the time of publication. PLOS allows rare exceptions to address legal and ethical concerns. See the PLOS Data Policy and FAQ for detailed information.	Yes - all data are fully available without restriction

A Data Availability Statement describing where the data can be found is required at submission. Your answers to this question constitute the Data Availability Statement and **will be published in the article**, if accepted.<br><br>**Important:** Stating 'data available on request from the author' is not sufficient. If your data are only available upon request, select 'No' for the first question and explain your exceptional situation in the text box.<br><br>Do the authors confirm that all data underlying the findings described in their manuscript are fully available without restriction?	
**Describe where the data may be found in full sentences. If you are copying our sample text, replace any instances of XXX with the appropriate details.**<br><br>• If the data are **held or will be held in a public repository**, include URLs, accession numbers or DOIs. If this information will only be available after acceptance, indicate this by ticking the box below. For example: *All XXX files are available from the XXX database (accession number(s) XXX, XXX.*).<br>• If the data are all contained **within the manuscript and/or Supporting Information files**, enter the following: *All relevant data are within the manuscript and its Supporting Information files.*<br>• If neither of these applies but you are able to provide **details of access elsewhere**, with or without limitations, please do so. For example:<br><br>*Data cannot be shared publicly because of [XXX]. Data are available from the XXX Institutional Data Access / Ethics Committee (contact via XXX) for researchers who meet the criteria for access to confidential data.*<br><br>*The data underlying the results presented in the study are available from (include the name of the third party*	The data underlying the results presented in the study are all uploaded as supplementary information files with the manuscript.

Powered by Editorial Manager® and ProduXion Manager® from Aries Systems Corporation

and contact information or URL).<br>• This text is appropriate if the data are owned by a third party and authors do not have permission to share the data.<br><br>* typeset	
Additional data availability information:	

Powered by Editorial Manager® and ProduXion Manager® from Aries Systems Corporation

Manuscript Click here to access/download;Manuscript;Manuscript.docx

Impact of brining and drying processes on the nutritive value of tambaqui fish (*Colossoma macropomum*)

Awot Teklu Mebratu[1,2*], Yohannes Tekle Asfaw[3], Wouter Merckx[4], Wouter H. Hendriks[5], Geert P.J. Janssens[1]

[1]Department of Veterinary and Biosciences, Faculty of Veterinary Medicine, Ghent University, Merelbeke, Belgium

[2]Department of Animal Reproduction and Welfare, College of Veterinary Sciences, Mekelle University, Mekelle, Ethiopia

[3]Department of Veterinary Basic and Diagnostic Sciences, College of Veterinary Sciences, Mekelle University, Mekelle, Ethiopia

[4]TRANSfarm, Engineering and technology group, The Catholic University of Leuven, Lovenjoel, Belgium

[5]Animal Nutrition Group, Wageningen University, Wageningen, The Netherlands

[*]Corresponding author

Email: awotteklu.mebratu@ugent.be (ATM)

Abstract

Preservation of fish as diet ingredient is challenging in many tropical regions due to poor socioeconomic conditions and lack of freezing facilities. So, alternative preservation techniques could be viable to address the issue. The present study evaluated the effect of brine salting (15% *w/w*) prior to drying at different temperatures on the nutrient profiles of tambaqui fish (*Colossoma macropomum*). Whole fish samples (792 ± 16 g; 8 months old; n = 48) were grouped into two as brine-salted and non-salted, and treated at seven different drying temperatures of 30, 35, 40, 45, 50, 55 and 60°C for a period of 23 h each. To evaluate the impact of Maillard reaction, reactive lysine was also quantified. Drying temperature had no effect on the evaluated macro- and micro-nutrients of tambaqui fish ($P > 0.05$) while brining reduced the overall protein concentration by 6% (58.8 to 55.4 g/100 g DM; $P = 0.004$). Brining significantly reduced many amino acids: taurine by 56% (7.1 to 3.1 g/kg; $P < 0.001$), methionine 17% (14.7 to 12.1 g/kg; $P < 0.001$), cysteine 11% (5.1 to 4.4 g/kg, $P = 0.016$), and reactive lysine 11% (52.0 to 46.4 g/kg; $P = 0.004$). However, alanine, arginine, and serine were not affected by brining ($P > 0.05$). Brining also reduced the concentrations of Se by 14% (149 to 128 µg/kg DM; $P = 0.020$), iodine 38% (604 to 373 µg/kg DM; $P = 0.020$), K 42% (9.71 to 5.61 g/kg DM; $P < 0.001$) and Mg 18% (1.32 to 1.10 g/kg DM; $P = < 0.001$) versus an anticipated vast increase in Na by 744% (2.70 to 22.90 g/kg DM; $P < 0.001$) and ash 28% (12.4 to 16.0 g/100g DM; $P < 0.001$) concentration. Neither brining nor drying temperature induced changes in % lysine reactivity and fat content of tambaqui fish ($P > 0.05$). Agreeably, results of multivariate analysis showed a negative association between brining, Na, and ash on one side of the component and most other nutrients on the other component. In conclusion, drying without brining may better preserve the nutritive value of tambaqui fish. However, as a practical remark to the industry sector, it is recommended that the final product is further evaluated for any pathogen of economic or public health importance.

Keywords: Brining; drying; nutrient profiles; tambaqui; reactive lysine.

Introduction

Fish meat is a high-value, perishable protein-rich food requiring appropriate preservation to maintain its organoleptic properties after harvest [1,2]. Nutritionally, fish meat is a prominent source of high quality essential amino acids [3,4], omega-3 fatty acids such as eicosapentaenoic acid (EPA) and docosahexaenoic acid (DHA), and trace elements such as iron, selenium, iodine that are beneficial for human health [5]. Hence, good knowledge on the nutrient profiles of fish meat enable a balanced and healthy diet for the human consumer and endorses consumer preferences on dietary requirements [5,6].

Tambaqui fish (*Colossoma macropomum*), also known as black pacu, is the main native fish farmed in South and Central American countries [7]. It is mainly found in Brazil, Venezuela, Colombia, Peru, and Bolivia. Tambaqui alone accounted for a total production of 142,135 tons in 2016 with Brazil accounting for about 96.4% of the total production [8]. Its life cycle is mainly associated with floody plains of white-water rivers in the amazon basins that are characterized with a temperature range of 25-34°C, low to neutral pH (6-7), and rich in suspended solids [9,10]. Tambaqui is an omnivorous fish that feeds on a variety of natural feeds such as fruits and seeds, and zooplanktons depending upon seasons [8,11]. It is considered to be a nocturnal feeder fish [7]. Nutritionally, the tambaqui is also rich in essential amino acids, EPA and DHA, selenium, and vitamins D and B [9]. Furthermore, tambaqui requires a fairly low protein content in its feed, most of which (75-85%) can be provided as plant protein [7,12]. This makes it possible to breed tambaqui with the aid of low-cost feed and feedstuffs, such as forest fruits or seeds of leguminous plants [7,13].

Food preservation is a fundamental requirement in maintaining quality food, a vital issue in human health, and contributes to profitability [14,15]. Fish meat, for instance, contains of nutrients that can easily get exposed to the ubiquitous environmental microbials (bacteria,

moulds,…) and chemicals causing degradation. The moisture content of fish meat predisposes for degradation and affects the nutritive value and shelf life of the product [6]. Fish meat often has a high moisture content averaging 70-84% where bacteria can grow rapidly and cause spoilage [4]. The suppression or killing of such microorganisms can be achieved by either reducing the temperature (e.g. chilling, freezing) or reducing the water content (e.g. drying, salting) [15]. Drying of fish meat products can be achieved by traditionally sun-drying or open air drying and using air driers, or microwaves [5,17]. Salting, on the other hand, involves different processes techniques such as brining (the immersion of fish meat into salt solution), kench curing (dry salting of fish fillets), pickling (longer storage of the fish with brine), and the possible injection of brine into the fish muscle all ending up in extended product shelf life [5,18-21].

Brine salting is one of the oldest traditional and economically viable preservation methods of edible animal proteins such as fish meat [22-24]. It involves the use of salt (NaCl) that, due to its hygroscopic property, withdraws water from tissues via osmosis and, thereby, reduces water activity to slow bacterial growth [22]. In addition, salt is also reported to improve product flavor, extract proteins, aid in emulsion formation, and reduce proteolytic activities of enzymes [25-27]. The quantity and concentration of salt and treatment time are determining factors in the process that could affect the nutritional quality of the fish [28-30]. A 15% brine concentration for a period of 48 h has been reported to yield optimum protein solubility/availability in a comparative study carried out in cod [24]. Likewise, better mass transfer kinetics of salt to tissues of cod and salmon were observed at 15% (*w/w*) brine concentration [23]. In fact, 10-15% (*w/w*) is the salt concentration recommended for salting whole fish under the food safety and standards regulation [31]. Higher concentrations of up to 25% (*w/w*) denature muscle proteins by causing cell membrane disruption, decreased enzymatic

activity, or interaction with myofibrils, and reducing its water-holding capacity [23,24,32]. In general, higher yields of tissue proteins are obtained at lower salt concentrations [24,26,33].

Drying involves the exposure of a product to specified temperature and time period to facilitate loss of tissue water to a lower limit of water activity (a_w < 0.90) where microbial growth is inhibited [34]. Drying temperature, relative humidity and air velocity are determining the rate of dehydration [2]. In rural areas, temperature-controlled ovens are usually not available, and open-air drying is the only option. Especially high environmental temperatures during drying may affect the nutritive value of fish, for instance because heat may damage amino acids or may increase the reaction of amino acids with lipids and/or carbohydrates to form biologically unavailable Maillard products [35]. To determine the risk of drying at uncontrolled temperature for the nutritive value of fish, it is warranted to study the effect of a wide enough temperature range for fish drying.

Depending on the type of drying oven and species and size of the fish involved, a temperature range of 65 - 135°C and drying time of 2 - 20 h have been routinely used for drying of fish meat [2,26]. However, due to possible conjoint loss of some non-moisture compounds (e.g. volatile fatty acids and vitamins), lower temperatures with extended drying time are often recommended [36]. For example, drying of salted shark at 35°C reported to yield improved protein solubility, tissue rehydration and water retention capacity and total protein estimates compared to 45, 55 and 60°C [37]. Pacheco [38] also stated that the maximum drying temperature for the majority of the edible fish meat should not exceed 70°C. In fact, drying of fish products at higher temperatures, of more than 60°C, reported to cause fat oxidation and caramelization of sugars [37].

Despite the effect of brining and drying temperatures on the nutrient profiles of different fish species has been investigated, we hypothesized that species differences in nutrient profiles could respond differently to drying and brining. We here evaluated tambaqui fish. Our study

also investigated reactive lysine as a marker for Maillard product formation: lysine is a limiting amino acid in most diets and needs to be bioavailable to be used in metabolism. Despite its high concentration in tissue deposition, some reducing sugars, fats and their oxidation products and vitamins can bind the ε-amino group of lysine and generate Maillard reaction products during fish processing [35]. Hence, measuring the content of "reactive lysine" as a percentage of total lysine in tambaqui fish can indicate state of protein damage during processing. The present study therefore evaluated the effect of brining and a range of drying temperatures on macronutrients and micronutrients, and mineral composition as well as on the extent of Maillard reaction that may reduce lysine availability.

Materials and methods

Experimental animal ethics and welfare

All experimental and animal care procedures were realized based upon the current written knowledge of aquaculture technologies and tambaquí fish farming. Fingerlings of *Colossoma macropomum* were initially brought from Aqua-Koi NV farm (http://www.aqua-koi.be/documents/home.xml?lang=nl) to TRANSfarm, Belgium. Fingerlings were then raised in a recirculating aquaculture system (RAS) with a weekly basis 30% water renewal (20 L/day) for about 7 months period before being subject to the current study. During their stay, fish were kept in larger aqua-tanks (100 cm × 120 cm × 40 cm) at a density of 1.2 fish/m^3 sufficient enough for movements and behavioral expression. Tanks were fixed with individual bio-filters, well-aerated with a constant temperature supply, 18:6 h light - dark cycle, and supplying pipes were regularly inspected and cleaned with tap water and water quality parameters were checked twice a week and water renewed accordingly [39].

For our experimental study, after a 12-h fasting, a total of 48 tambaqui fish were randomly selected and kept in a separate tank and were euthanized in a two-step procedure [40]. Six fish were randomly taken at a time and placed in a tank with a volume of 240 L water into which

50 mL of phenoxyethanol-solution (Anest Fish©, Germany) was added. After five minutes, 30 L of water was elevated out of the tanks and put into a large bucket. Another 6 mL of Anest Fish© was added to this bucket. Each group of six unconscious fish was scooped out of the tank and brought to the slaughter room in the bucket with an anesthetic agent (1 mL:5 L, Anest Fish©, Germany). Thereafter, tambaqui were killed with a firm blow to the head, followed by fracturing the brainstem with a knife and then transported in ice cubes for the experiment [40]. The study was conducted in accordance with the Declaration of Helsinki and directive 2010/63/EU of the European parliament and of the council on the protection of animals used for scientific purposes. The wellbeing of the cultured tambaqui fish before being subjected to the experiment was assessed and approved by the institutional review board of TRANSfarm, KU Leuven, under laboratory accreditation of KU/LA-1210616.

Study materials

A total of 48 tambaqui fish with a mean weight of 792 (± 16) g and 8 months old were collected from TRANSfarm, Lovenjoel, Belgium, and transported to the animal nutrition laboratory of the Department of Veterinary and Biosciences, Ghent University in styrofoam boxes with ice packs (Luxaplast, NORDIC®). Upon arrival, to facilitate drying whole fish were dissected dorso-ventrally into two equal slices using a stainless post mortem saw (SC-PM/110, 254MM) and scalpel-blades, thoroughly washed using distilled water in pipes and then individually stored in plastic bags at -20°C for 48 h. All dissected fish slices had an average uniform dimension of 21.2 ± 2.8 cm long and 12.6 ± 2.0 cm wide and average weight of 389 ± 11.5 g. Industrial table salt (99.9%, BDH-PROLABO®, EC) was used for preparing the 15% (*w/w*) brine solution in the present study [24].

Experimental design and set-up

Uniformly sliced whole fish samples were grouped into two as brine-salted and non-salted, and treated at seven different drying temperatures of 30, 35, 40, 45, 50, 55 and 60°C for a period of 23 h each [23,24,31,37]. Each temperature treatment was considered as a sampling point and tested in triplicate. Similarly, as a reference point, fresh whole-fish samples were initially evaluated in triplicate for their nutritional profiles.

Brine salting and drying processes

Brine-salted fish samples were immersed in brine solution (15% *w/w*) at a ratio of 3:1 of fish to salt solution [22,27]. For each sampling unit, samples were totally immersed and brined together in a closable plastic bucket (20 L) for 48 h at 4°C. The brine solution was pre-cooled to 4°C prior to the immersion of samples. Afterwards, brined samples were drained from salt drips for 3 min using a plastic sieve and randomly distributed into individual aluminium trays (500 mL; 142 mm × 116 mm × 30.4 mm; Boni™, Halle, Belgium), at one fish per tray, into which small beads (13 mm diameter, 3.5 g, JVLAB®) were added (10 beads/tray) at the bottom to avoid crusting of samples and optimize moisture loss. Likewise, the non-salted group of fish samples were randomly distributed into identical aluminium trays containing similar beads. Each tray was placed in an electric oven (EP66, BEKSO-SA®, Belgium) and randomly set and dried with the above-mentioned temperature points. The laboratory humidity was in a range of 50-60% during the experimental period. Drying temperature between sampling units were controlled using an oven-fixed thermometer and a consistent oven temperature (± 1°C) was maintained throughout the drying time period. A constant air ventilation was supplied through an oven-fixed ventilator at a steady air velocity of 1.0 m/s. Drying time started after oven closure and the oven returned to its set temperature (~ 5 min). After 23 h, samples were collected and allowed to cool in a desiccator for 5 min to room temperature before weighing. Samples were then kept at -21°C overnight and finally freeze-dried (CoolSafe™, SCANVAC, Denmark)

for a period of 16 h each to a stable weight, and uniformly ground (2.0 mm sieve). Subsequently, the samples were homogenized using a grinder machine (De΄Longhi™, KG48) at 12× speed and then individually kept in closable plastic containers at 4°C until analyzed [22].

Nutrient evaluations

Proximate analysis

So as to make legitimate comparisons between the processed samples, the quantitative analysis of the values of macromolecules is a significant nutritional marker and for which case the proximate analysis was used to determine the crude protein (CP), ether extract and ash contents of fish samples. The dry matter (DM) content of freeze-dried samples (1 g) was determined through oven-drying at 105°C for 24 h to constant weight [40]. Ether-extract was determined using the acid hydrolysis method [41], CP using the Kjeldahl method involving a nitrogen analyzer (LECO FP528 Instruments, St. Joseph, Michigan, USA) and N × 6.25 conversion factor [42], and ash content using an automated muffle furnace (Nabertherm™, UK) as per the procedures described by AOAC [43]. A gram of fish sample was used for the analysis of each of the macronutrients and all samples were evaluated in duplicate at the animal nutrition laboratory of the Department of Veterinary and Biosciences, Ghent University, Belgium.

Mineral analysis

The total ash content was determined following the ISO method [44] and mineral (calcium, sodium, potassium, copper, iron, magnesium, manganese, phosphorus and zinc) concentrations of fish were evaluated after dry ashing mineralization according to ISO method (ICP-AES: ISO 11885) as per the procedures described by Gorsuch [45]. In brief, a gram of sample was weighed, placed into a porcelain crucible and oven-dried at 105°C for 3 h. Sample was then burnt on a hot plate and incinerated in a muffle furnace at 450°C for 16 h. Obtained ash was then moistened with a small amount of deionized water first and dissolved in 10 mL of 1:1 (*v/v*) solution of hydrochloric acid (HCl) and deionized water and evaporated to dry. Finally,

the mix was re-dissolved in 10 mL of 1:9 (*v/v*) solution of HCl and deionized water, transferred into a 50 mL flask, and then diluted several folds for optimal determination of individual mineral concentration using induction-coupled plasma optical emission spectrometry (ICP-OES) method as per the manufacturer's procedures (Iris Intrepid II XSP, Thermo Fisher Scientific, Aalst, Belgium). So as to obtain an optimum concentration range for the atomic absorption spectrometric method, the concentrations of certain minerals were determined after fivefold (Mg and Ca), 10-fold (Na and Zn), and 50-fold (K) dilutions. Likewise, the concentration of selenium was determined by ISO method (ICP-MS: ISO 11885) and iodine with tetramethylammonium hydroxide extraction and iodine quantification (ICP-MS: ISO 11885) at the Laboratory of Chemical analysis, Ghent University, Belgium. As quality control protocols, visual inspection of pump tubing at regular intervals (every 30 min) and replacing between samples were often made to increase the precision measurements. Similarly, regular inspection and cleaning of the injector tube, spray chamber and nebulizer, and cleaning of the widows are done to avoid contamination and reduction of light intensity. Blank solutions with similar matrix were used to improve the measurement accuracy and the detection limit set was 1-100 ppb.

Amino acid and OMIU-reactive lysine analyses

The total amino acid profiling was done by acid hydrolysis (2 g) at 110°C for 23 h and ion-exchange chromatography with post-column derivatization with ninhydrin method (ISO 13903). Sulphur-containing amino acids were measured as cysteic acid and methionine sulfone after oxidation with performic acid; tryptophan was measured by alkaline hydrolysis at 110°C for 20 h and ion-exchange chromatography with fluorescence detection method (ISO 13904) according to procedures described by Hulshof [46].

For analysis of reactive lysine, about 2 g of fish sample were processed from each of the seven sampling points for defatting by extraction with light petroleum ether without acid

hydrolysis (ISO 6492), finely ground and homogenized (2.0 mm sieve size) using a mixer mill (Retsch MM200, BV) and used for the analysis of reactive lysine via O-methylisourea (OMIU) method according to Hulshof [46]. Briefly, 1 mL of 0.6 M solution of OMIU was prepared as described by Moughan and Rutherfurd [47] that converts lysine with free e-amino group to homoarginine. Four gram of barium hydroxide octahydrate (Sigma-Aldrich, Zwijndrecht, the Netherlands) was added to 16 mL of boiling (10 min) distilled deionized water to remove CO_2, after which 2 g of OMIU sulphate salt was added. The solution was then cooled for 30 min at room temperature, centrifuged (6,400 × g for 10 min at 20°C) and the supernatant was retained and the precipitate was washed with about 2 mL of same water and further centrifuged (6,400 × g for 10 min at 20°C). both supernatants were combined and pH adjusted to 11.5 (for guanidation) by adding 6 M HCl solution. The homoarginine content was analysed in duplicate and the OMIU-reactive lysine was calculated from the homoarginine content. All amino acid profile, including reactive lysine, were determined at Wageningen University & Research, Animal nutrition laboratory, The Netherlands.

Statistical analysis

Data obtained in the current study were analyzed using SPSS statistical software version 27 (IBM Corporation, Armonk, NY, USA). All data were checked for normality distributions using histogram and QQ-plots. A univariate one-way ANOVA was used with brining as fixed factor and temperature as covariate to test the hypothesis about differences between two or more mean nutrient values. Data were presented as means and standard error of the means (SEM). Statistical significance was set at $P < 0.05$ for any differences observed between samples. Furthermore, principal component analysis (PCA) was computed using on the collective factors obtained to appraise the relationships of nutrient profiles based on their responses towards brining and drying methods, with values deemed relevant below -0.5 and above 0.5 of the components' values.

Results

Tambaqui fish samples were grouped into two as brine-salted and non-salted, and treated at seven different drying temperatures for 23 h each. Each temperature treatment was considered as sampling point and tested in triplicate (n = 3) and mean values obtained were statistically computed for all nutrient profiles evaluated.

Proximate composition

Drying temperature had no significant effect on the measured nutrient profiles of the fish in this study and all data were combined across temperature treatments ($P > 0.05$; Fig 1). However, several effects ($P < 0.05$) were observed with the brining method.

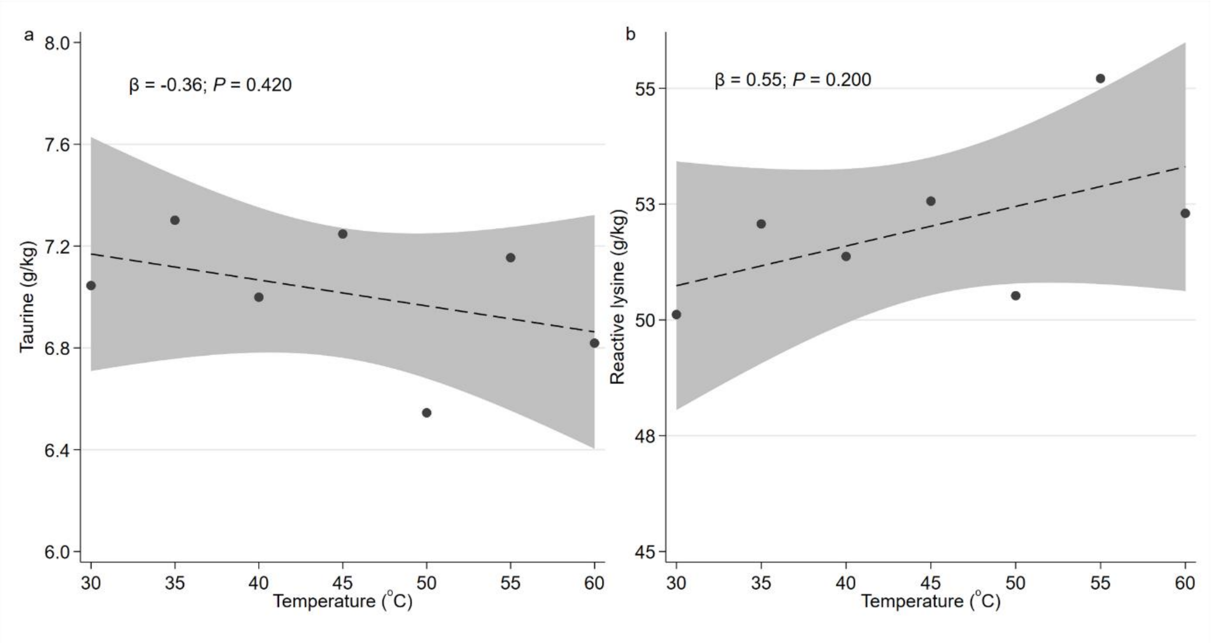

Fig 1. The effect of drying temperature on nutrient profiles of tambaqui fish. (a) gram of taurine per kg fish; (b) gram of reactive lysine per kg fish.

Brining resulted in a higher ash concentration with a relative increase of 28% ($P < 0.05$). Moreover, 6% loss on the overall protein concentration was noticed due to brining ($P < 0.05$). The body fat contents of tambaqui fish were not affected by the addition of salt and temperature treatments ($P > 0.05$; Table 1).

Table 1. Changes in macronutrient concentrations in tambaqui fish (on %DM basis) due to brining and drying at seven different temperatures (30, 35, 40, 45, 50, 55 and 60°C).

Concentration (g/100 g DM)	Not salted	Salted	Total	Relative % Change	SEM	*p*	
						salted	temp
**Ash**	12.4	16.0	14.1	28	0.5	< 0.001	0.793
**Crude protein**	58.8	55.4	57.0	-6	0.6	0.004	0.846
**Crude fat**	28.3	28.7	28.7	1	0.4	0.716	0.657

DM= Dry matter; SEM= standard error of the means; temp= temperature.
Values represent mean and standard error of the means based on three replicates per treatment and seven treatments; n = 48.

Minerals and amino acids

The essential minerals Se and I, and the macrominerals K and Mg were significantly reduced by brining with 14, 38, 42 and 18% percent losses, respectively ($P < 0.05$). Moreover, brining resulted in an obvious enormous significant increase (744%) in Na concentration ($P < 0.05$). Otherwise, all minerals (Fe, Mn, P and Ca) were decreased by brining (Table 2).

Table 2. Changes in mineral concentrations in tambaqui fish due to brining and drying at seven different temperatures (30, 35, 40, 45, 50, 55 and 60°C).

Minerals	Not salted	Salted	Total	Relative % Change	SEM	*p*	
						salted	temp
**Macrominerals (g/kg DM)**							
**Ca**	37.4	37.1	37.0	-1	1.1	0.965	0.965
**K**	9.71	5.61	7.71	-42	0.6	<0.001	0.495
**Mg**	1.32	1.10	1.10	-18	0.03	<0.001	0.880
**Na**	2.70	22.9	12.1	744	2.7	<0.001	0.564
**P**	21.7	20.4	21.0	-6	0.6	0.317	0.814
**Microminerals (mg/kg DM)**							
**Fe**	42.0	36.9	39.2	-12	2.4	0.256	0.188
**I***	604	373	489	-38	49	0.020	0.264
**Mn**	9.80	8.81	9.31	-10	0.3	0.115	0.152
**Se***	149	128	137	-14	5	0.020	0.449

**Zn**	48.5	50.6	49.6	4	1.3	0.473	0.588

DM= Dry matter; SEM= standard error of the means; temp= temperature.
Values represent mean and standard error of the means based on three replicates per treatment and seven treatments; n = 48.
*Values are presented in µg/kg DM.

Brining negatively affected the overall sum amino acid profile (*P < 0.05*). In particular, taurine, histidine, cysteine, methionine and available (reactive) lysine were affected by brining with the highest percent loss accounted for taurine (56%) followed by methionine (17%), cyteine (11%), and histidine (10%) (*P < 0.05*). In contrast, there was numerical 5 and 6% increase in proline and glycine respectively, whereas alanine and arginine were not affected by brining (*P > 0.05*; Table 3).

Table 3. Changes in amino acid concentrations in tambaqui fish due to brining and drying at seven different temperatures (30, 35, 40, 45, 50, 55 and 60°C).

**Amino acids (g/kg CP)**	**Not salted**	**salted**	**Total**	**Relative % Change**	**SEM**	***p***	
						**salted**	**temp**
Taurine	7.10	3.10	5.11	-56	0.5	<0.001	0.324
Asparagine + Aspartic acid	56.8	54.9	55.4	-3	0.9	0.268	0.671
Threonine	27.0	26.3	26.5	-3	0.4	0.311	0.965
Serine	25.0	24.9	24.8	0	0.2	0.807	0.777
Glutamine + Glutamic acid	91.4	88.3	89.2	-3	1.3	0.247	0.851
Glycine	75.7	80.2	79.3	6	2.5	0.361	0.750
Alanine	51.1	51.0	51.2	0	0.7	0.961	0.636
Valine	30.8	29.2	29.8	-5	0.5	0.062	0.642
Isoleucine	26.0	24.6	24.9	-5	0.5	0.162	0.838
Leucine	44.3	41.8	42.5	-6	0.9	0.134	0.855
Tyrosine	17.9	16.5	16.9	-8	0.5	0.098	0.952
Phenylalanine	24.5	23.4	23.8	-5	0.4	0.134	0.874
Histidine	15.5	14.0	14.6	-10	0.4	0.054	0.400
Lysine	51.1	47.4	48.6	-7	1.1	0.070	0.863
Arginine	48.3	48.3	48.5	0	0.5	0.999	0.464
Proline	47.5	49.9	49.5	5	1.3	0.356	0.660

Cysteine	5.10	4.40	4.60	-11	0.1	0.016	0.746
Methionine	14.7	12.1	13.3	-17	0.4	<0.001	0.675
Tryptophan	5.10	4.60	4.71	-8	0.2	0.229	0.698
Reactive lysine	52.0	46.4	48.5	-11	1.2	0.004	0.851
% Reactive lysine	102.2	97.9	99.9	-4	1.8	0.306	0.760
Sum AA	665	645	653	-3	6.87	0.017	0.621

CP= Crude protein; AA= Amino acid; SEM= Standard error of the means; temp= temperature.
Values represent mean and standard error of the means based on three replicates per treatment and seven treatments; n = 48.
[a]Percentage of reactive lysine in the total protein.
[b]Percentage of reactive lysine in the total lysine.

Multivariate classification of variables

Principal component analysis was computed on the collective factors obtained to appraise the relationships of nutrient profiles based on their responses towards brining and drying methods. By examining at the scree plot and eigenvalues and looking for a point at which the proportion of variance explained by each subsequent principal component drops off, an elbow in the screen plot, the most explaining two components 1 (44.8%) and 2 (18.9%) were considered and a biplot PCA generated.

In line, there were no associations between nutrient profiles and drying temperature, while several associations were observed with brining. Negative association was seen between brining, Na and ash on one side of component 1 and most other nutrients on the other. However, positive association between the brining proxies (Na, ash) and amino acids prominent in connective tissue (glycine, proline, arginine, alanine) were typically seen clustered together right of the component. The more central position of Zn, Fe, fat, and available Lys point to their low response to brining and temperature. More importantly, temperature itself was positioned centrally in the plot, emphasizing the absence of drying temperature effects on the study parameters (Fig 2).

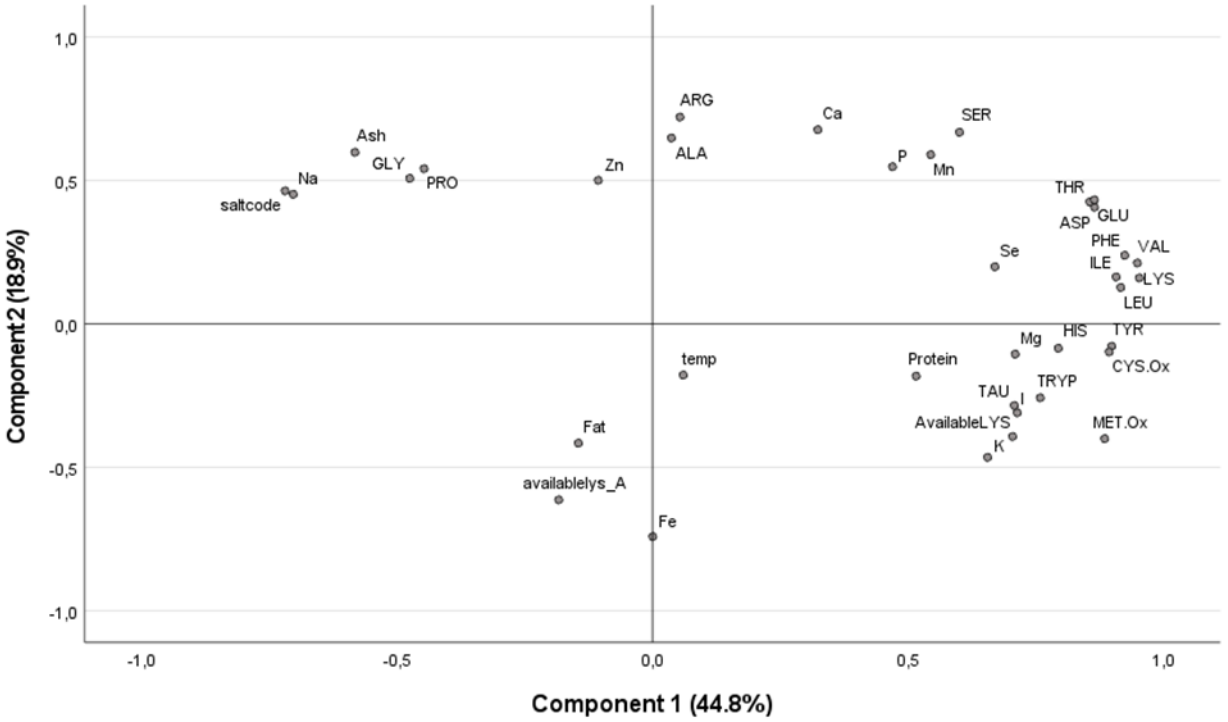

Fig 2. Principal component analysis classification of nutrient profiles based on their response to brining and temperature. Analyses represent principal component analysis (PCA) of evaluated nutrient values (expressed as percentages of the sum of total) after brining and drying of whole tambaqui fish samples (n = 48). AvailableLYS, is the percentage of reactive lysine in the total protein; availablelys_A, is the percentage of reactive lysine in the total lysine; saltcode, refers to brining; temp, is the drying temperature.

Discussion

The present study evaluated the effect of brine salting (15% *w/w*) prior to drying at different temperatures on the nutrient profiles of tambaqui fish. Brining induced a prominent effect on macronutrient and micronutrient profiles of tambaqui fish.

In this study, brining induced pronounced losses of specific amino acids and minerals, irrespective of drying temperature. Gill et al. [48] found that salting enhanced the aggregation of myosin in cod, yet only above a drying temperature of 50°C. Marínez-Alvarez and Gómez-Guillén [33] indicated that there is a notable loss of soluble muscle proteins (particularly, actin and myosin) by osmosis in salting (NaCl) at pH 6.5 on cod muscle (*Gadus morhua*). Differences between studies can evolve from a wide range of conditions used in the drying process,

including the drying type, drying temperature and duration, relative humidity, air velocity and the specific characteristics of the product dried (species and size of the fish). Although comparison with existing literature does not allow us to pinpoint the factors that protected from a drying temperature effect, this study suggests that brining is associated with nutrient losses. In the present study, a total of 6% crude protein was lost due to the brining stress. In fact, salt concentration and salting duration can modulate the effect of brining on muscular protein loss in fish [28-30]. The solubility of muscle proteins often start to decrease with increasing salt concentration due to protein aggregation [24] and/or altered enzymatic activities [49]. In the present study, however, an acceptable (15% *w/w*) salt was used for brining [31].

The amino acid analysis in this study demonstrated that the salt-induced protein loss was not an overall effect, but reduced specific amino acids, taurine, histidine, cysteine, methionine and available lysine. Taurine is not involved in protein synthesis but often accumulates in muscle tissues of fish and has osmolytic properties [50,51]. Hence, the loss in taurine seem a logical reaction to the osmotic stress from brining. This taurine loss can be important since its role in health has been demonstrated for instance in infants [52] and it is also the main amino acid to form conjugated bile acids in chickens [53].

The tendency to a lower histidine concentration due to brining can be seen in the same light. Apart from its protein-bound appearance, histidine is stored in muscular tissue as either free histidine (acetylated or not) or bound to mainly beta-alanine (hence constituting dipeptides, such as carnosine or anserine); this storage of histidine occurs throughout the animal kingdom [54]. Shiau [55] reported that histidine can easily get released from muscle tissues during stress conditions, such as starvation. Significant losses of the free amino acids histidine, taurine and glycine were reported in salted milkfish [32]. Histidine is involved in the regulation and metabolism of essential trace minerals, formation of enzymes, induction of the immune system

through the production of histamine and protects brain cells through the formation of metallothionein among others [56].

Cysteine and methionine are sulphur-containing amino acids and quite reactive to processing, in that they could either get directly affected by the ionic brine solution or can possibly make cross-links that reduce their tissue availability [32]. Kumazawa [57] reported that brining may cause cross-linking reactions of muscle myosin heavy chains and form ε-(γ-glutamyl)lysine through transglutaminase. There was no effect on the percentage of reactive lysine (% available-lysine), meaning that both salting and drying did not induce Maillard reactions that affect lysine availability. The relative decrease in the total lysine likely explains the decreased percentage of available lysine in the current study.

Four specific amino acids in the principal component analysis separated from the negative association between brining and the other amino acids: alanine and arginine were not affected, while glycine and proline were even positively associated with brining. These amino acids are prominent in the skeleton and in scales, which is a strong indication that the highest protein losses were from muscle and other soft tissues [58]. Other authors not always report similar changes in amino acid pattern: a sigmoid initial increase (up to 9 h) and final slight decrease (48 h) of alanine, glutamine and lysine was reported in salted milkfish [32]; alanine, glycine, lysine and histidine increased, when salted fish was dried [59]. The increase in these amino acid concentrations could be due to the decrease in the proportion of the extracted total lipids present as polyunsaturated fatty acids as depicted by Yankah [59]. Fat is a hydrophobic molecule and can act as a physical barrier to limit salt (NaCl) and water transport across the muscle tissue [23]. In fact, the lipid content was higher for the fish in our study (28.71%) although fat concentration was not affected by both treatments. Of the affected amino acids by brining, taurine and lysine are polar hydrophilic where are methionine and cysteine have basic hydrophobic property [47,50,51].

The specific loss of soft tissue from the fish is not only supported by the amino acid data, but also witnessed by the change in mineral profile. The considerable loss of the microminerals Se and I and the macrominerals K and Mg following brining reflects the presence of these minerals in soft tissue [60]. Selenium typically occurs as replacement of sulphur in sulphur-containing amino acids (cysteine and methionine) in soft tissues. The decrease in methionine and cysteine seen in our study, therefore, fits the observation of decreased selenium. In contrast, the typical skeletal minerals, such as Ca, P and Mg, and those that are deposited in scales and fins such as Zn and Fe, were not affected. Potassium is a strong electrolyte that could disappear in the brine solution or get filtered out in exchange with the added sodium. The loss of water due to salt was explained as the main cause for leaching of these macrominerals in the solution during brining. The decrease in K concentration agrees with Polak-Juszczak [61] and Bakhiet [62] from salted fish. The latter study remarkably reported a salt-induced increase in iodine concentration, most likely evolving from salt impurities as proposed by the authors. It is not clear how these differences with our study can be explained, but we used a purified source of salt, hence excluding potential unintended supply of microminerals to the fish tissues through brining.

Drying is an energy intensive process, which can use about 10% or more of the total energy consumed during processing. In the present study, although our aim was not to quantify the drying efficiency, we conjointly were aiming to consume less energy while drying samples. This was achieved by increasing the sample surface area of exposure, avoiding surface contact by putting beads, improved equipment insulation during and between drying, supply of a constant air ventilation at 1.0 m/s during drying, and minimizing the timing between dryings [63].

Undeniably, brining has substantially increased Na and ash concentrations. This has been observed in most studies on brining of fish [61,62]. This enrichment with salt through brining

must be considered when formulating diets but, more importantly, our results demonstrate that brining may not be the best choice to maintain the nutritive value of fish. Evidently, we must acknowledge that nutritive value is not the only criterion to pick a preservation method for fish: for the reduction of the pathogenic load in fish products, salt addition may still be a valuable technique that goes, however, at the expense of nutritive value as shown in our study. A comforting finding is that the wide range in drying temperature evaluated in our study had no noticeable impact on the nutritive value of the fish.

5. Conclusions

This research shows that drying temperature has no effect on nutrient composition of the studied fish which could be encouraging for drying of fish within the drying temperature range used in this study. Brining, however, reduced some important trace elements such as Se and I, and lead to loss of muscle crude protein, with the concomitant amino acids. Brining also increased the leaching of K, I, and taurine. The formation of Maillard products was not promoted by both drying and brining treatments, as demonstrated through unaltered reactive lysine proportions. Therefore, drying without brining may better preserve the nutritive value of tambaqui fish. However, as a recommendation to the industry sector, it is suggested that the final products are further evaluated for any pathogen of economic or public health importance.

Acknowledgments

The authors acknowledge Herman De Rycke for the proximate analysis.

References

1. Pawar SM, Sonawane SR. Fish muscle protein highest source of energy. Int J Biodivers Conserv. 2013; 5:433–435. doi: 10.5897/IJBC12.043.

2. di Domenico CNB. Fish drying: A review. Rev Bras Eng Pesca. 2014; 7:1–18. doi: 10.18817/repesca.v7i1.1061.

3. Marta M, Francisca H, Robert S. Fatty acids profile in canned tuna and sardine after retort sterilization and high pressure thermal sterilization treatment. J Food Nutr Res. 2015; 54:171–178. doi: hdl.handle.net/10261/114599.

4. Abraha B, Admassu H, Mahmud A, Tsighe N, Shui XW, Fang Y. Effect of processing methods on nutritional and physico-chemical composition of fish: a review. MOJ Food Process Technol. 2018; 6:376-82. doi: 10.15406/mojfpt.2018.06.00191.

5. Sampels S. The effects of processing technologies and preparation on the final quality of fish products. Trends Food Sci Technol. 2015; 44:131-146. doi: 10.1016/j.tifs.2015.04.003.

6. Kumar MS. Sustainable management of fisheries and aquaculture for food security and nutrition: policies requirements and actions. Agric Res. 2014; 3:97-103.

7. da Silva JAM, Pereira-Filho M, Oliveira-Pereira MI. Seasonal variation of nutrients and energy in tambaquí's (*Colossoma macropomum* Cuvier, 1818) natural food. Braz J Biol. 2000; 60:4. doi: 10.1590/s0034-71082000000400009

8. Woynárovich A, van Anrooy R. Field guide to the culture of tambaquí Colossoma macropomum, Cuvier, 1816. In: FAO Fisheries and Aquaculture Technical Paper. 2019; 624:21-30.

9. Arantes ML, Freitas CEC. Effects of fisheries zoning and environmental characteristics on population parameters of the tambaqui (*Colossoma macropomum*) in managed floodplain lakes in the Central Amazon. Fish Manag Ecol. 2016; 23:133–143. doi: 10.1111/fme.12164

10. Val AL, de Oliveira AM. *Colossoma macropomum*—A tropical fish model for biology and aquaculture. J Exp Zoo Part A: Ecol Integ Phys. 2021; 335:761-70. doi: 10.1002/jez.2536

11. Barat, J.M., Rodríguez-Barona, S., Andrés, A., & Fito, P. (2002). Influence of increasing brine concentration in the cod salting process. J Food Sci. 65(7):1922–1925. doi: 10.1111/j.1365-2621.2002.tb08747.x.

12. Gomes LC, Silva CR. Impact of pond management on tambaquí, *Colossoma macropomum* (Cuvier), production during growth-out phase. Aqua Res. 2009; 40:825–832. doi: 10.1111/j.1365-2109.2009.02170.x

13. Sandre LCG, Buzollo H, Nascimento TMT, Neira LM, Jomori RK, Carneiro DJ. Productive performance and digestibility in the initial growth phase of tambaquí (*Colossoma macropomum*) fed diets with different carbohydrate and lipid levels. Aqua Rep. 2017; 6:28–34. doi: 10.1016/j.aqrep.2017.02.003

14. Heijnen C, Boogaard BVD, Maas van Berkel B. Preservation of fish and meat. Agrodok. 2004; Pp. 26-35.

15. Miladi H, Chaieb K, Bakhrouf A, Elmnasser A, Ammar E. Freezing effects on survival of Listeria monocytogenes in artificially contaminated cold fresh-salmon. Annals Microbiol. 2008; 58:471-476.

16. Mahmoud BSM, Yamazaki K, Miyashita K, Shin II, Suzuki T. A new technology of fish preservation by combined treatment with electrolysed NaCl solutions and essential oil compounds. Food Chem. 2006; 99:656-662. doi: 10.1016/j.foodchem.2005.08.037.

17. Djendoubi N, Boudhrioua N, Bonazzi C, Kechaou N. Drying of sardine muscles: Experimental and mathematical investigations. Food Bioprod Process. 2009; 87:115-123. doi: 10.1016/j.fbp.2008.07.003.

18. Birkeland S, Skåra T, Bjerkeng B, Rørå AMB. Product yield and gaping in cold-smoked Atlantic salmon (*Salmo salar*) fillets as influenced by different injection-salting techniques. J Food Sci. 2003; 68:1743-1748.

19. Guizani N, Rahman MS, Al-Ruzeiqi MH, Al-Sabahi JN, Sureshchandran S. Effects of brine concentration on lipid oxidation and fatty acids profile of hot smoked tuna (*Thunnus albacares*) stored at refrigerated temperature. J Food Sci Technol. 2014; 51:577-582. doi: 10.1007/s13197-011-0528-4.

20. Hall G. Preservation by curing (drying, salting and smoking). In: G. Hall (Ed.), Fish processing sustainability and new opportunities, West Sussex: Wiley-Blackwell. 2011; Pp. 51-76.

21. Kituu GM, Shitanda D, Silayo D, Odote P, Bongyereire J. Effect of brining on the drying rate of tilapia (*Oreochromis niloticus*) in a solar tunnel dryer. Fish Aqua Cluster Proceed. 2000; 88-98. http://ir.mksu.ac.ke/handle/123456780/4889.

22. Binici A, Kaya GK. Effect of brine and dry salting methods on the physicochemical and microbial quality of chub (*Squalius cephalus* Linnaeus, 1758). Food Sci Technol. 2018; 38:66–70. doi: 10.1590/1678-457X.15717.

23. Gallart-Jornet L, Barat JM, Rustad T, Erikson U, Escriche I, Fito P. A comparative study of brine salting of Atlantic cod (*Gadus morhua*) and Atlantic salmon (*Salmo salar*). J Food Eng. 2007; 79:261–270. doi: 10.1016/j.jfoodeng.2006.01.053.

24. Nguyen MV, Thorarinsdottir KA, Gudmundsdottir A, Thorkelsson G, Arason S. The effects of salt concentration on conformational changes in cod (*Gadus morhua*) proteins during brine salting. Food Chem. 2011; 125:1013–1019. doi: 10.1016/j.foodchem.2010.09.109.

25. Rust RE, Knipe CL. Sausage casings. In: Devine C, Dikeman M, editors. Encyclopedia of Meat Sciences. Academic Press: Oxford; 2014. pp. 235–240.

26. Thorarinsdottir KA, Arason S, Bogason S, Kristbergsson K. The effects of various salt concentrations during brine curing of cod (*Gadus morhua*). Int J Food Sci Technol. 2004; 39(1):79–89. doi: 10.1046/j.0950-5423.2003.00757.x.

27. Thorarinsdottir KA, Arason S, Sigurgisladottir S, Valsdottir T, Tornberg E. Effects of different pre-salting methods on protein aggregation during heavy salting of cod fillets. Food Chem. 2011; 124(1):7–14. doi: 10.1016/j.foodchem.2010.05.095.

28. Barat JM, Rodríguez-Barona S, Andrés A, Fito P. Influence of increasing brine concentration in the cod salting process. J Food Sci. 2002; 67(5):1922–1925. doi: 10.1111/j.1365-2621.2002.tb08747.x.

29. Larsen R, Elvevoll EO. Water uptake, drip losses and retention of free amino acids and minerals in cod (*Gadus morhua*) fillet immersed in NaCl or KCl. Food Chem. 2008; 107(1):369–376. doi: 10.1016/j.foodchem.2007.08.031.

30. Thorarinsdottir KA, Arason S, Geirsdottir M, Bogason SG, Kristbergsson K. Changes in myofibrillar proteins during processing of salted cod (*Gadus morhua*) as determined by electrophoresis and differential scanning calorimetry. Food Chem. 2002; 77(3):377–385. doi: 10.1016/S0308-8146(01)00349-1.

31. FSSA. Fish and fish products regulations. In: Gaur VN, editors. Food Safety and Standards Regulation Authority (FSSA). New Delhi: The gazette of India, 2011. pp. 366-369.

32. Sannaveerappa T, Ammu K, Joseph J. Protein-related changes during salting of milkfish (*Chanos chanos*). J Sci Food Agric. 2004; 84(8):863–869. doi: 10.1002/jsfa.1682.

33. Marínez-Alvarez O, Gómez-Guillén MC. The effect of brine composition and pH on the yield and nature of water-soluble proteins extractable from brined muscle of cod (*Gadus morhua*). Food Chem. 2005; 92(1):71–77. doi: 10.1016/j.foodchem.2004.04.049.

34. Ockerman HW, Basu L. Preservation methods of animal products. In: Dikeman M, Devine C, editors. Encyclopedia of Meat Sciences. London: Oxford Elsevier Ltd.; 2014. pp. 78–83. doi: 10.1016/B978-0-12-384731-7.00207-5.

35. Kim JC, Mullan BP, Pluske JR. Prediction of apparent, standardized, and true ileal digestible total and reactive lysine contents in heat-damaged soybean meal samples. J Anim Sci. 2012; 90:137–139. doi: 10.2527/jas.53819.

36. Lantry BF, O'Gorman R. Drying temperature effects on fish dry mass measurements. J Great Lakes Res. 2004; 33:606–616. doi: 10.3394/0380-1330(2007)33[606:DTEOFD]2.0.CO;2.

37. Bastos JR. Influência da secagem sobre algumas propriedades físico-químicas do músculo do cação branco, Carcharhynus porosus Ranzani (in portuguess, with english abstract). Arquivos de Ciências do Mar. 1997; 17:77–87. Available from: http://www.repositorio.ufc.br/handle/riufc/1403

38. Pacheco N, Garnica-Gonzalez M, Gimeno M, Bárzana E, Trombotto S, David L, et al. Structural characterization of chitin and chitosan obtained by biological and chemical methods. Biomacromolecules. 2011; 12:3285–3290. doi: 10.1021/bm200750t.

39. Frisso RM, de Matos FT, Moro GV, de Mattos BO. Stocking density of Amazon fish (*Colossoma macropomum*) farmed in a continental neotropical reservoir with a net cages system. Aquaculture. 2020; 529:p.735702. doi: 10.1016/j.aquaculture.2020.735702

40. Lewbart GA. Euthanasia of ectotherms. In: Fowler's zoo and wild animal medicine current therapy. Elsevier. 2019; 9:357–363. doi: 10.1016/b978-0-323-55228-8.00051-5.

41. AOAC. Official Methods of Analysis of the Association of Official Analytical Chemists. 16th ed. Arlington VA; 1995.

42. AOAC. Official Methods of Analysis of the Association of Official Analytical Chemists. 17th ed. Washington DC: Gaithersburg publishing; 2001.

43. AOAC. Official Methods of Analysis of the Association of Official Analytical Chemists. 15th ed. Arlington VA; 1990.

44. ISO. Meat and Meat Products - Determination of total ash. In: International Organization for Standardization. Switzerland: ISO/936; 1998. Available from: https://www.iso.org/standard/24783.html

45. Gorsuch TT. The Destruction of organic matter. 1st ed. New York: Oxford pergamon press Ltd; 1970. pp. 40–54.

46. Hulshof TG, Bikker P, Van der Poel AFB, Hendriks WH. Assessment of protein quality of soybean meal and 00-rapeseed meal toasted in the presence of lignosulfonate by amino acid digestibility in growing pigs and Maillard reaction products. J Anim Sci. 2016; 94:1020–1030. doi: 10.2527/jas.2015-9700.

47. Moughan PJ, Gall MPJ, Rutherfurd SM. Absorption of lysine and deoxyketosyllysine in an early Maillard browned casein by the growing pig. J Agric Food Chem. 1996; 44:1520–1525. doi: 10.1021/jf950428v.

48. Gill TA, Chan TK, Phozchareon KF, Paulson AT. Effect of salt concentration and temperature on heat-induced aggregation and gelation of fish myosin. Food Res Int. 1992; 25:333–341. doi: 10.1016/0963-9969(92)90108-H.

49. Stoknes IS, Walde PM, Synnes M. Proteolytic activity in cod (*Gadus morhua*) muscle during salt curing. Food Res Int. 2005; 38*:*693–699. doi: 10.1016/j.foodres.2005.02.003.

50. Geda F, Declercq AM, Remø SC, Lourenço RWM, Janssens GPJ. The metabolic response in fish to mildly elevated water temperature relates to species-dependent muscular concentrations of imidazole compounds and free amino acids. J Therm Biol. 2017; 65:57-63. doi: 10.1016/j.jtherbio.2017.02.004.

51. Zhang Y, Dabrowski K, Hliwa P, Gomulka P. Indispensable amino acid concentrations decrease in tissues of stomachless fish, common carp in response to free amino acid- or peptide-based diets. Amino Acids. 2006; 31(2):165–172. doi: 10.1007/s00726-006-0345-7.

52. Chesney RW, Helms RA, Christensen M, Budreau AM, Han X, Sturman JA. The role of taurine in infant nutrition. Adv Exp Med Biol. 1998; 442:463–476. doi: 10.1007/978-1-4899-0117-0_56.

53. Alzawqari MH, Al-Baadani HH, Alhidary IB, Al-Owaimer AN, Abudabos AM. Effect of taurine and bile acid supplementation and their interaction on performance, serum components, ileal viscosity and carcass characteristics of broiler chickens. S Afr J Anim Sci. 2016; 46(4):448–457. doi: 10.4314/sajas.v46i4.13.

54. Boldyrev AA, Aldini G, Derave W. Physiology and pathophysiology of carnosine. Physiol Rev. 2013; 93:1803–1845. doi: 10.1152/physrev.00039.2012.

55. Shiau CY, Pong YJ, Chiou TK, Tin YY. Effect of starvation on free histidine and amino acids in white muscle of milkfish *Chanos chanos*. Comp Biochem Physiol B: Biochem Mol Biol. 2001; 128:501–506. doi: 10.1016/S1096-4959(00)00350-X.

56. Kopeć W, Jamroz D, Wiliczkiewicz A, Biazik E, Pudlo A, Hikawczuk T, et al. Influence of different histidine sources and zinc supplementation of broiler diets on dipeptide content and antioxidant status of blood and meat. Br Poult Sci. 2013; 54:454–465. doi: 10.1080/00071668.2013.793295.

57. Kumazawa K, Seguro K, Takamura M, Motoki M. Formation of ε-(γ-glutamyl)lysine cross-link in cured horse mackerel meat induced by drying. J Food Sci. 1993; 58:1062–1064. doi: 10.1111/j.1365-2621.1993.tb06112.x.

58. Fedina IS, Georgieva K, Grigorova I. Light-dark changes in proline content of barley leaves under salt stress. Biol Plant. 2002; 45:59–63. doi: 10.1023/A:1015175802847.

59. Yankah MV, Ohshima T, Koizumi C. Effects of processing and storage on some chemical characteristics and lipid composition of a Ghanian fermented fish product. J Sci Food Agric. 1993; 63:227–235. doi: 10.1002/jsfa.2740630209.

60. Yamashita Y, Yamashita M, Iida H. Selenium Content in Seafood in Japan. Nutrients. 2013; 5:388–395. doi: 10.3390/nu5020388.

61. Polak-Juszczak L. Effects of processing methods on the content of minerals in fish products. J Elem. 2016; 21:461-470. doi: 10.5601/jelem.2015.20.3.890.

62. Bakhiet HHA, Khogali FAH, Ahmed FAM. Effect of different salt concentrations on mineral content of the fish (*Hydrocynus* spp.). J Life Sci Biomed. 2013; 3(1):01–03.

63. Pask F, Lake P, Yang A, Tokos H, Sadhukhan J. Industrial oven improvement for energy reduction and enhanced process performance. Clean Technol Environ Policy. 2017; 19:215-224. doi: 10.1007/s10098-016-1206-z.

Figure 1

Click here to access/download;Figure;Fig 1.tif

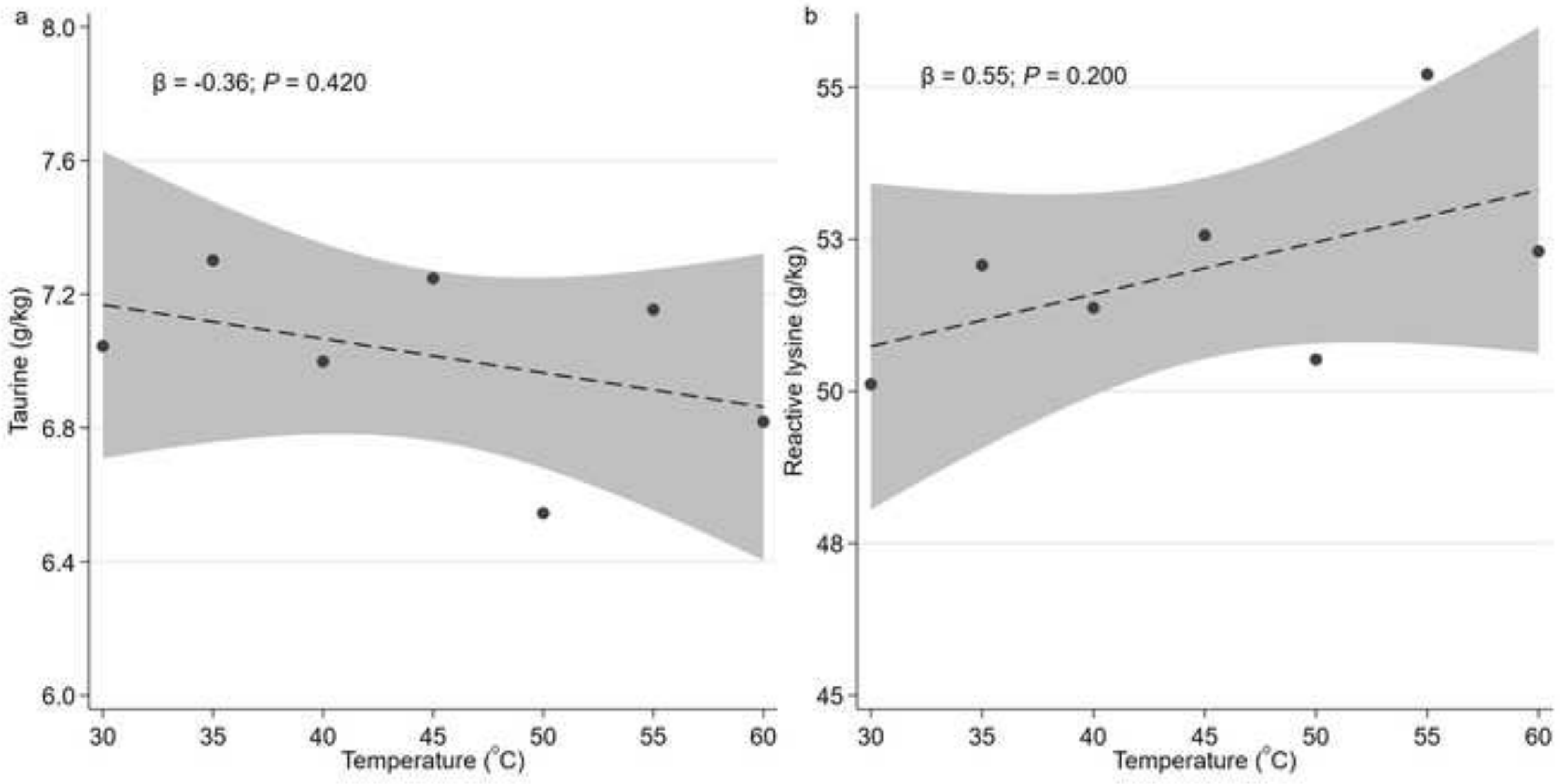

Figure 2

Click here to access/download;Figure;Fig 2.tif

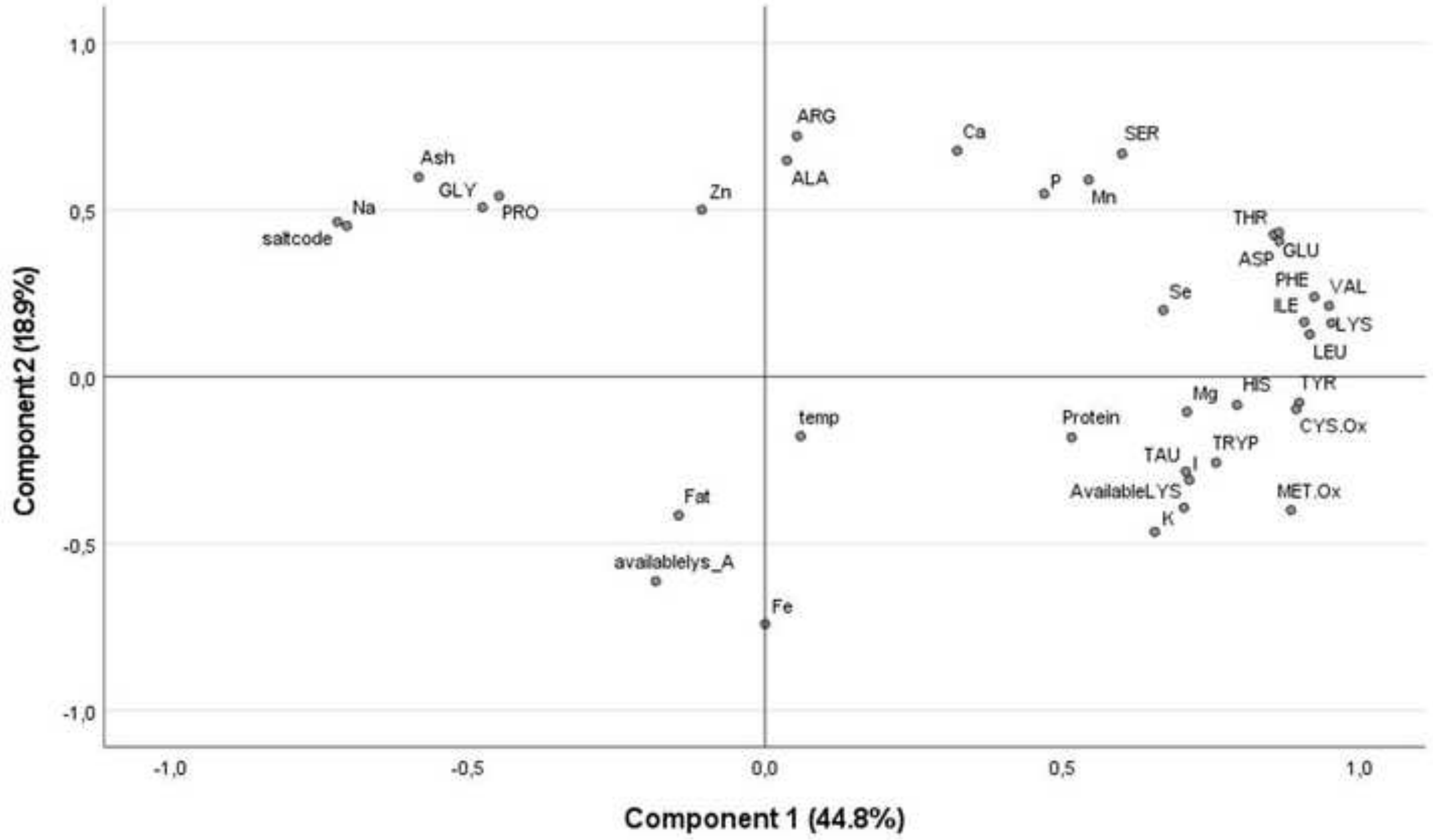

Click here to access/download
Supporting Information - Compressed/ZIP File Archive
Supporting information files.zip

Impact of brining and drying processes on the nutritive value of tambaqui fish (*Colossoma macropomum*)

Awot Teklu Mebratu[1,2*], Yohannes Tekle Asfaw[3], Wouter Merckx[4], Wouter H. Hendriks[5], Geert P.J. Janssens[1]

[1]Department of Veterinary and Biosciences, Faculty of Veterinary Medicine, Ghent University, Merelbeke, Belgium

[2]Department of Animal Reproduction and Welfare, College of Veterinary Sciences, Mekelle University, Mekelle, Ethiopia

[3]Department of Veterinary Basic and Diagnostic Sciences, College of Veterinary Sciences, Mekelle University, Mekelle, Ethiopia

[4]TRANSfarm, Engineering and technology group, The Catholic University of Leuven, Lovenjoel, Belgium

[5]Animal Nutrition Group, Wageningen University, Wageningen, The Netherlands

[*]Corresponding author

Email: awotteklu.mebratu@ugent.be (ATM)

Abstract

Preservation of fish as diet ingredient is challenging in many tropical regions due to poor socioeconomic conditions and lack of freezing facilities. So, alternative preservation techniques could be viable to address the issue~~Preservation of fish as diet ingredient is challenging in many tropical conditions due to the absence of freezing facilities, hence warranting other preservation techniques~~. ~~Brine salting is one of the oldest traditional and economically viable preservation methods.~~ The present study evaluated the effect of brine salting (15% *w/w*) prior to drying at different temperatures on the nutrient profiles of tambaqui fish (*Colossoma macropomum*). Whole fish samples (792 ± 16 g; 8 months old; n = 48) were grouped into two as brine-salted and non-salted, and treated at seven different drying temperatures of 30, 35, 40, 45, 50, 55 and 60°C for a period of 23 h each. To evaluate the impact of Maillard reaction, reactive lysine was also quantified. Drying temperature had no effect on the evaluated macro- and micro-nutrient ~~profile~~s of tambaqui fish ($P > 0.05$) while brining reduced the overall protein concentration by 6% (58.8 to 55.4 g/100 g DM; $P = 0.004$). Brining significantly ~~diminished~~reduced many amino acids: taurine by 56% (7.1 to 3.1 g/kg; $P < 0.001$), methionine 17% (14.7 to 12.1 g/kg; $P < 0.001$), cysteine 11% (5.1 to 4.4 g/kg, $P = 0.016$), and reactive lysine 11% (52.0 to 46.4 g/kg; $P = 0.004$). However, alanine, arginine, and serine were not affected by brining ($P > 0.05$). Brining also reduced the concentrations of Se by 14% (149 to 128 μg/kg ~~dry matter~~DM; $P = 0.020$), iodine~~I~~ 38% (604 to 373 μg/kg DM; $P = 0.020$), K 42% (9.71 to 5.61 g/kg DM; $P < 0.001$) and Mg 18% (1.32 to 1.10 g/kg DM; $P = < 0.001$) versus an anticipated vast increase in Na by 744% (2.70 to 22.90 g/kg DM; $P < 0.001$) and ash 28% (12.4 to 16.0 g/100g DM; $P < 0.001$) concentration. Neither brining nor drying temperature induced changes in % lysine reactivity and fat content of tambaqui fish ($P > 0.05$). ~~Agreeably, results of multivariate analysis showed a negative association between brining, Na, and ash on one side of the component and most other nutrients on the other while the lack of effect of temperature can be seen by its~~

Formatted: Font: 12 pt, Font color: Auto

~~presence in the center.~~ Agreeably, results of multivariate analysis showed a negative association between brining, Na, and ash on one side of the component and most other nutrients on the other component. In conclusion~~Therefore~~, drying without brining may better preserve the nutritive value of tambaqui fish. However, as a practical remark to the industry sector, it is recommended that the final product is~~s are~~ further evaluated for any pathogen of economic or public health importance.

Keywords: Brining; drying; nutrient profiles; tambaqui; reactive lysine.

Introduction

Fish meat is a high-value, perishable protein-rich food requiring appropriate preservation to maintain its organoleptic properties after harvest [1,2]. Nutritionally, fish meat is a prominent source of high quality essential amino acids [3,4], omega-3 fatty acids such as eicosapentaenoic acid (EPA) and ~~(~~docosahexaenoic acid (DHA), and trace elements such as iron, selenium, iodine that are beneficial for ~~the~~ human health [5].~~ Depending on the species of the fish, culture system (farmed versus wild), feeding behavior (herbivores, carnivores, omnivores), and agroecology (temperate versus tropical; freshwater versus marine water) these essential nutrients, and their bioavailability, vary from one to other fish.~~ Hence, good~~a~~ knowledge on the nutrient profiles of fish meat enable~~do promote~~ a balanced and healthy diet for the human consumer and endorses consumer preferences on dietary requirements [5,6].

Tambaqui fish (*Colossoma macropomum*), also known as black pacu, is the main native fish ~~species~~ farmed in South and Central American countries [7]. It is mainly found in Brazil, Venezuela, Colombia, Peru, and Bolivia. Tambaqui~~he species~~ alone accounted for a total production of 142,135 tons in 2016 with Brazil accounting for about 96.4% of the total production [8]. Its life cycle is mainly associated with floody plains of white-water rivers in the amazon basins that are characterized with a temperature range of 25-34ºC, low to neutral pH (6-7), and rich in suspended solids [9,10].

Tambaqui is an omnivorous fish that feeds on a variety of natural feeds such as fruits and seeds, and zooplanktons depending upon seasons [8,11]. It is considered to be a nocturnal feeder fish [7].~~ The higher growth performance, hardiness, and socioeconomic value of tambaqui fish have nowadays led to technology endorsement in its farm level production [9,12,13].~~ Nutritionally, the tambaqui is also rich in essential amino acids, EPA and DHA, selenium, and vitamins D and B [9]. Furthermore, tambaqui requires a fairly low protein content in its feed, most of which (75-85%) can be provided as plant protein [7,12]. This makes it possible to breed tambaqui with the aid of low-cost feed and feedstuffs, such as forest fruits or seeds of leguminous plants [7,13].

Food p~~P~~reservation is a fundamental requirement ~~of food and food ingredients~~ in maintaining ~~a~~ quality food, ~~which is~~ a vital issue in ~~the~~ human health, and contributes to profitability [14,15]. Fish meat, for instance, contains ~~several set~~ of nutrients ~~(moisture, proteins, carbohydrates, and vitamins)~~ that can easily get exposed to the ubiquitous environmental microbials (bacteria, moulds,…) and chemicals causing degradation. The moisture content of ~~can be regarded as the primary nutrient input for~~ fish meat predisposes for degradation and affects~~ing~~ the nutritive value and shelf life of the product [6]. F~~Generally, f~~ish meat often has~~ve~~ a high~~er~~ moisture content averaging 70-84% where bacteria can grow rapidly and cause spoilage [4]. The suppression or killing of such microorganisms can be achieved by either reducing the temperature (e.g. chilling, freezing) or reducing the water content (e.g. drying, salting) [15]. Drying of fish meat products can be achieved by traditionally sun-drying or open air drying and using air driers, or microwaves [5,17]. Salting, on the other hand, involves different processes techniques such as brining (the immersion of fish meat into salt solution), kench curing (dry salting of fish fillets), pickling (longer storage of the fish with brine), and the possible injection of brine into the fish muscle all ending up in ~~reducing product water content and hence~~ extended product shelf life [5,18-21].

Brine salting is one of the oldest traditional and economically viable preservation methods of edible animal proteins such as fish meat [22-24]. It involves the use of salt (NaCl) that, due to its hygroscopic property, withdraws water from tissues via osmosis and, thereby, reduces water activity to slow bacterial growth [22]. In addition, salt is also reported to improve product flavor, extract proteins, aid in emulsion formation, and reduce proteolytic activities of enzymes [25-27].

The quantity and concentration of salt and treatment time are determining factors in the process that could affect the nutritional quality of the fish [28-30]. A 15% brine concentration for a period of 48 h has been reported to yield optimum protein solubility/availability in a comparative study carried out in cod [24]. Likewise, better mass transfer kinetics of salt to tissues of cod and salmon were observed at 15% (*w/w*) brine concentration [23]. In fact, 10-15% (*w/w*) is the salt concentration recommended for salting whole fish under the food safety and standards regulation [31]. Higher concentrations of up to 25% (*w/w*) denature muscle proteins by causing cell membrane disruption, decreased enzymatic activity, or interaction with myofibrils, and reducing its water-holding capacity [23,24,32]. In general, higher yields of tissue proteins are obtained at lower salt concentrations [24,26,33]. ~~In addition to the salt concentration, the purity of salt, salting method involved, the size, thickness and freshness of the fish, fish to salt ratio, and weather conditions are further factors of importance for the quality brine preserved fish [22,23].~~

Drying involves the exposure of a product to specified temperature and time period to facilitate loss of tissue water to a lower limit of water activity ($a_w < 0.90$) where microbial growth is inhibited [34]. Drying temperature, relative humidity and air velocity are determining the rate of dehydration [2]. In rural areas, temperature-controlled ovens are usually not available, and open-air drying is the only option. Especially high environmental temperatures during drying may affect the nutritive value of fish, for instance because heat may damage

amino acids or may increase the reaction of amino acids with lipids and/or carbohydrates to form biologically unavailable Maillard products [35]. To determine the risk of drying at uncontrolled temperature for the nutritive value of fish, it is warranted to study the effect of a wide enough temperature range for fish drying.

Depending on the type of drying oven and species and size of the fish involved, a temperature range of 65 - 135°C and drying time of 2 - 20 h have been routinely used for drying of fish meat [2,26]. However, due to possible conjoint loss of some non-moisture compounds (e.g. volatile fatty acids and vitamins), lower temperatures with extended drying time are often recommended [36]. For example, drying of salted shark at 35°C reported to yield improved protein solubility, tissue rehydration and water retention capacity and total protein estimates compared to 45, 55 and 60°C [37]. Pacheco [38] also stated that the maximum drying temperature for the majority of the edible fish meat should not exceed 70°C. In fact, drying of fish products at higher temperatures, of more than 60°C, reported to cause fat oxidation and caramelization of sugars [37].

Despite the effect of brining and drying temperatures on the nutrient profiles of different fish species has been investigated, we hypothesized that species differences in nutrient profiles could respond differently to drying and brining. We ~~and~~ here evaluated tambaqui fish. Our study also investigated reactive lysine as a marker for Maillard product formation: lysine is a limiting amino acid in most ~~animal~~ diets and needs to be bioavailable to be used in metabolism. Despite its high concentration in tissue deposition, some reducing sugars, fats and their oxidation products and vitamins can bind the ε-amino group of lysine and generate Maillard reaction products during fish processing [35]. Hence, measuring the content of "reactive lysine" as a percentage of total lysine in tambaqui fish can indicate state of protein damage during processing. The present study therefore evaluated the effect of brining and a range of drying

temperatures on ~~the ~~macronutrients and micronutrients, and mineral composition as well as on the extent~~state ~~ of Maillard reaction that may reduce lysine availability.

Materials and methods

Experimental animal ethics and welfare

All experimental and animal care procedures were realized based upon the current written knowledge of aquaculture technologies and tambaquí fish farming. Fingerlings of *Colossoma macropomum* were initially brought from Aqua-Koi NV farm (http://www.aqua-koi.be/documents/home.xml?lang=nl) to TRANSfarm, Belgium. Fingerlings were then~~and~~ ~~reared~~raised in a recirculating aquaculture system (RAS) with a weekly basis 30% water renewal (20 L/day) for about 7 months period before being subject to the current study. During their stay, fish were kept in larger aqua-tanks (100 cm × 120 cm × 40 cm) at a density of 1.2 fish/m^3 sufficient enough for movements and behavioral expression. Tanks were fixed with individual bio-filters, well~~-~~aerated with a constant temperature supply, 18:6 h light - dark cycle, and supplying pipes were regularly inspected and cleaned with tap water and water quality parameters were checked twice a week and water renewed accordingly [39].

For our experimental study, after a 12 ~~-~~h fasting, a total of 48 tambaqui fish were randomly selected and kept in a separate tank and were euthanized in a two-step procedure [40]. Six fish were randomly taken at a time and placed in a tank with a volume of 240 L water into which 50 mL of phenoxyethanol-solution (Anest Fish©, Germany) was added. After five minutes, 30 L of water was~~ere~~ elevated out of the tanks and put into a large bucket. Another 6 mL of Anest Fish© was added to this bucket. Each group of six unconscious fish was scooped out of the tank and brought to the slaughter room in the bucket with an~~the~~ anesthetic agent (1 mL:5 L, Anest Fish©, Germany). Thereafter, tambaqui were killed with a firm blow to the head, followed by fracturing the brainstem with a knife and then transported in ice cubes for the experiment [40]. The study was conducted in accordance with the Declaration of Helsinki and directive

2010/63/EU of the European parliament and of the council on the protection of animals used for scientific purposes. T~~, and t~~he wellbeing of the cultured tambaqui fish before being subjected to the experiment was assessed and approved by the institutional review board of TRANSfarm, KU Leuven, under laboratory accreditation of ~~(~~KU/LA-1210616~~)~~.

Study materials

A total of 48 tambaqui fish with a mean weight of 792 (± 16) g and 8 months old were collected from TRANSfarm, Lovenjoel, Belgium, and transported to the animal nutrition laboratory of the Department of V~~v~~eterinary and B~~b~~iosciences, Ghent University in styrofoam boxes with ice packs (Luxaplast, NORDIC®). Upon arrival, to facilitate drying whole fish were dissected dorso-ventrally into two equal slices using a stainless post mortem saw (SC-PM/110, 254MM) and scalpel-blades, thoroughly washed using distilled water in pipes and then individually stored in plastic bags at -20°C for 48 h. All dissected fish slices had an average uniform dimension of 21.2 ± 2.8 cm long and 12.6 ± 2.0 cm wide and average weight of 389 ± 11.5 g. Industrial table salt (99.9%, BDH-PROLABO®, EC) was used for preparing the 15% (*w/w*) brine solution in the present study [24].

Experimental design and set-up

Uniformly sliced whole fish samples were grouped into two as brine-salted and non-salted, and treated at seven different drying temperatures of 30, 35, 40, 45, 50, 55 and 60°C for a period of 23 h each [23,24,31,37]. Each temperature treatment was considered as a sampling point and tested in triplicate. Similarly, as a reference point, fresh whole-fish samples were initially evaluated in triplicate for their nutritional profiles.

Brine salting and drying processes

Brine-salted fish samples were immersed in brine solution (15% *w/w*) at a ratio of 3:1 of fish to salt solution [22,27]. For each sampling unit, samples were totally immersed and brined

together in a closable plastic bucket (20 L) for 48 h at 4°C. The brine solution was pre-cooled to 4°C prior to the immersion of samples. Afterwards, brined samples were drained from salt drips for 3 min using a plastic sieve and randomly distributed into individual aluminium trays (500 mL; 142 mm × 116 mm × 30.4 mm; Boni™, Halle, Belgium), at one fish per tray, into which small beads (13 mm diameter, 3.5 g, JVLAB®) were added (10 beads/tray) at the bottom to avoid crusting of samples and optimize moisture loss. Likewise, the non-salted group of fish samples were randomly distributed into identical aluminium trays containing similar beads. Each tray was placed in an electric oven (EP66, BEKSO-SA®, Belgium) and randomly set and dried with the above-mentioned temperature points. The laboratory humidity was in a range of 50-60% during the experimental period. Drying temperature between sampling units were controlled using an oven-fixed thermometer and a consistent oven temperature (± 1°C) was maintained throughout the drying time period. A constant air ventilation was supplied through an oven-fixed ventilator at a steady air velocity of 1.0 m/s. Drying time started after oven closure and the oven returned to its set temperature (~ 5 min). After 23 h, samples were collected and allowed to cool in a desiccator for 5 min to room temperature before weighing. Samples were then kept at -21°C overnight and finally freeze-dried (CoolSafe™, SCANVAC, Denmark) for a period of 16 h each to a stable weight, and uniformly ground (2.0 mm sieve). Subsequently, the samples were~~and~~ homogenized using a grinder machine (De´Longhi™, KG48) at 12× speed and then individually kept in closable plastic containers at 4°C until analyzed [22].

Nutrient evaluations

Proximate analysis

So as to make legitimate comparisons between the processed samples, the quantitative analysis of the values of macromolecules is a significant nutritional marker and for which case the proximate analysis was used to determine the crude protein (CP), ether extract and ash contents of fish samples. The dry matter (DM) content of freeze-dried samples (1 g) was

determined through oven-drying at 105°C for 24 h to constant weight [40]. Ether-extract was determined using the acid hydrolysis method [41], CP using the Kjeldahl method involving a nitrogen analyzer (LECO FP528 Instruments, St. Joseph, Michigan, USA) and N × 6.25 conversion factor [42], and ash content using an automated muffle furnace (Nabertherm™, UK) as per the procedures described by AOAC [43]. A gram of fish sample was used for the analysis of each of the macronutrients and all samples were evaluated in duplicate at the animal nutrition laboratory of the Department of Veterinary and Biosciences, Ghent University, Belgium.

Mineral analysis

The total ash content was determined following the ISO method [44] and mineral (calcium, sodium, potassium, copper, iron, magnesium, manganese, phosphorus and zinc) concentrations of fish were evaluated after dry ashing mineralization according to ISO method (ICP-AES: ISO 11885) as per the procedures described by Gorsuch [45]. In brief, a gram of sample was weighed, placed into a porcelain crucible and oven-dried at 105°C for 3 h. Sample was then burnt on a hot plate and incinerated in a muffle furnace at 450°C for 16 h. Obtained ash was then moistened with a small amount of deionized water first and dissolved in 10 mL of 1:1 (*v/v*) solution of hydrochloric acid (HCl) and deionized water and evaporated to dry. Finally, the mix was re-dissolved in 10 mL of 1:9 (*v/v*) solution of HCl and deionized water, transferred into a 50 mL flask, and then diluted several folds for optimal determination of individual mineral concentration using induction-coupled plasma optical emission spectrometry (ICP-OES) method as per the manufacturer's procedures (Iris Intrepid II XSP, Thermo Fisher Scientific, Aalst, Belgium). So as to obtain an optimum concentration range for the atomic absorption spectrometric method, the concentrations of certain minerals were determined after fivefold (Mg and Ca), 10-fold (Na and Zn), and 50-fold (K) dilutions. Likewise, the concentration of selenium was determined by ISO method (ICP-MS: ISO 11885) and iodine with tetramethylammonium hydroxide extraction and iodine quantification (ICP-MS: ISO

11885) at the Laboratory of Chemical analysis, Ghent University, Belgium. As quality control protocols, visual inspection of pump tubing at regular intervals (every 30 min) and replacing between samples were~~are~~ often made to increase the precision measurements. Similarly, regular inspection and cleaning of the injector tube, spray~~ead~~ chamber and ~~mobilizer~~nebulizer, and cleaning of the widows are done to avoid contamination and reduction of light intensity. Blank~~e~~ solutions with similar matrix were used to improve the measurement accuracy and the detection limit set was 1-100 ppb.

Amino acid and OMIU-reactive lysine analyses

The total amino acid profiling was done by acid hydrolysis (2 g) at 110°C for 23 h and ion-exchange chromatography with post-column derivatization with ninhydrin method (ISO 13903). Sulphur-~~-~~containing amino acids were measured as cysteic acid and methionine sulfone after oxidation with performic acid; tryptophan was measured by alkaline hydrolysis at 110°C for 20 h and ion-exchange chromatography with fluorescence detection method (ISO 13904) according to procedures described by Hulshof [46].

For analysis of reactive lysine, about 2 g of fish sample were processed from each of the seven sampling points for defatting by extraction with light petroleum ether without acid hydrolysis (ISO 6492), finely ground and homogenized (2.0 mm sieve size) using a mixer mill (Retsch MM200, BV) and used for the analysis of reactive lysine via O-methylisourea (OMIU) method according to Hulshof [46]. Briefly, 1 mL of 0.6 M solution of OMIU was prepared as described by Moughan and Rutherfurd [47] that converts lysine with free e-amino group to homoarginine. Four gram of barium hydroxide octahydrate (Sigma-Aldrich, Zwijndrecht, the Netherlands) was added to 16 mL of boiling (10 min) distilled deionized water to remove CO_2, after which 2 g of OMIU sulphate salt was added. The solution was then cooled for 30 min at room temperature, centrifuged (6,400 × g for 10 min at 20°C) and the supernatant was retained and the precipitate was washed with about 2 mL of same water and further centrifuged (6,400

× g for 10 min at 20°C). both supernatants were combined and pH adjusted to 11.5 (for guanidation) by adding 6 M HCl solution. The homoarginine content was analysed in duplicate and the OMIU-reactive lysine was calculated from the homoarginine content. All amino acid profile, including reactive lysine, were determined at Wageningen University & Research, Animal nutrition laboratory, The Netherlands.

Statistical analysis

Data obtained in the current study were analyzed using SPSS statistical software version 27 (IBM Corporation, Armonk, NY, USA). All data were checked for normality distributions using histogram and QQ-plots. A univariate one-way ANOVA was used with brining as fixed factor and temperature as covariate to test the hypothesis about differences between two or more mean nutrient values. Data were presented as means and standard error of the means (SEM). Statistical significance was set at $P < 0.05$ for any differences observed between samples. Furthermore, principal component analysis (PCA) was computed using on the collective factors obtained to appraise the relationships of nutrient profiles based on their responses towards brining and drying methods, with values deemed relevant below -0.5 and above 0.5 of the components' values.

Results

Tambaqui fish samples were grouped into two as brine-salted and non-salted, and treated at seven different drying temperatures for 23 h each. Each temperature treatment was considered as sampling point and tested in triplicate (n = 3) and mean values obtained were statistically computed for all nutrient profiles evaluated.

Proximate compositionanalysis

Drying temperature had no significant effect on the measured nutrient profiles of the fish in this study and all data were combined across temperature treatments ($P > 0.05$; Fig 1). However, several effects ($P < 0.05$) were observed with the brining method.

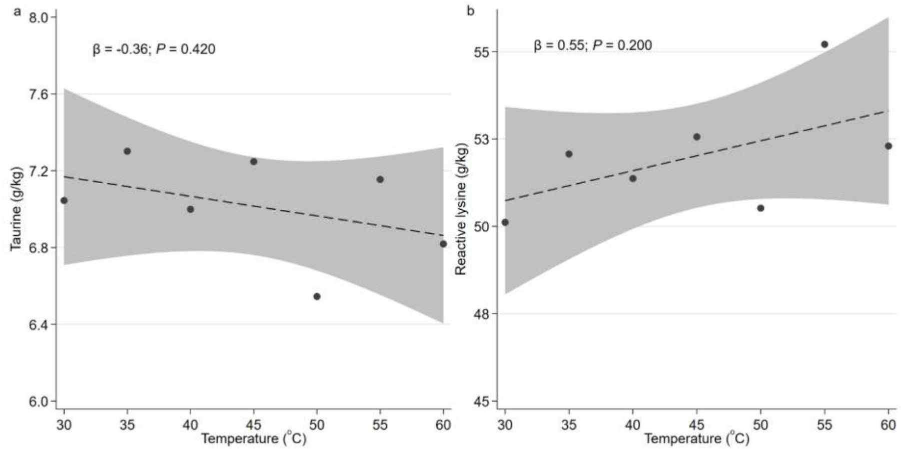

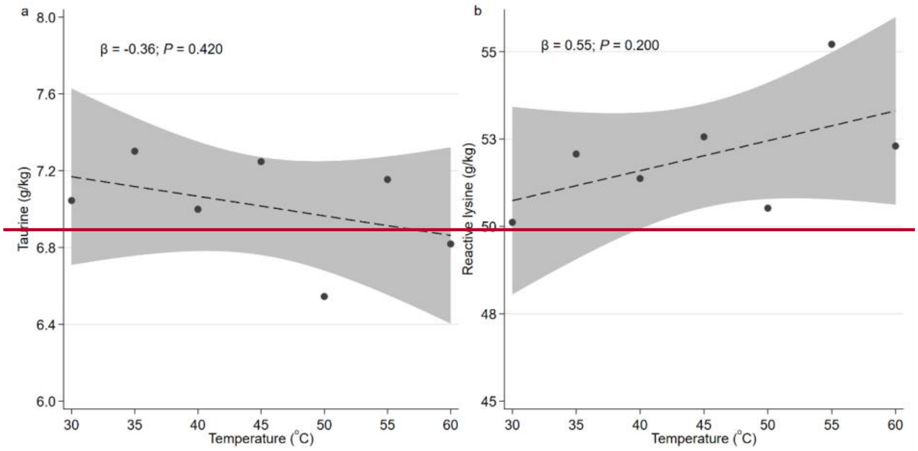

Fig 1. The effect of drying temperature on nutrient profiles of tambaqui fish. (a) gram of taurine per kg fish; (b) gram of reactive lysine per kg fish.

Brining resulted in a higher ash concentration with a relative increase of 28% ($P < 0.05$). Moreover, 6% loss on the overall protein concentration was noticed due to brining ($P < 0.05$). The body fat contents of tambaqui fish were not affected by the addition of salt and temperature treatments ($P > 0.05$; Table 1).

Table 1. Changes in macronutrient concentrations in tambaqui fish (on %DM basis) due to brining and drying at seven different temperatures (30, 35, 40, 45, 50, 55 and 60˚C).

Concentration (g/100 g DM)	Not salted	Salted	Total	Relative % Change	SEM	*p*	
						salted	temp
**Ash**	12.4	16.0	14.1	28	0.5	< 0.001	0.793
**Crude protein**	58.8	55.4	57.0	-6	0.6	0.004	0.846
**Crude fat**	28.3	28.7	28.7	1	0.4	0.716	0.657

DM= Dry matter; SEM= standard error of the means; temp= temperature.
Values represent mean and standard error of the means based on three replicates per treatment and seven treatments; n = 48.

Minerals and amino acids

The essential minerals Se and I, and the macrominerals K and Mg were significantly reduced by brining with 14, 38, 42 and 18% percent losses, respectively ($P < 0.05$). Moreover, brining resulted in an obvious enormous significant increase (744%) in Na concentration ($P < 0.05$). Otherwise, all minerals (Fe, Mn, P and Ca) were decreased by brining (Table 2).

Table 2. Changes in mineral concentrations in tambaqui fish due to brining and drying at seven different temperatures (30, 35, 40, 45, 50, 55 and 60˚C).

Minerals	Not salted	Salted	Total	Relative % Change	SEM	*p*	
						salted	temp
**Macrominerals (g/kg DM)**							
**Ca**	37.4	37.1	37.0	-1	1.1	0.965	0.965
**K**	9.71	5.61	7.71	-42	0.6	<0.001	0.495
**Mg**	1.32	1.10	1.10	-18	0.03	<0.001	0.880
**Na**	2.70	22.9	12.1	744	2.7	<0.001	0.564

**P**	21.7	20.4	21.0	-6	0.6	0.317	0.814
**Microminerals (mg/kg DM)**							
**Fe**	42.0	36.9	39.2	-12	2.4	0.256	0.188
**I***	604	373	489	-38	49	0.020	0.264
**Mn**	9.80	8.81	9.31	-10	0.3	0.115	0.152
**Se***	149	128	137	-14	5	0.020	0.449
**Zn**	48.5	50.6	49.6	4	1.3	0.473	0.588

DM= Dry matter; SEM= standard error of the means; temp= temperature.
Values represent mean and standard error of the means based on three replicates per treatment and seven treatments; n = 48.
*Values are presented in µg/kg DM.

Brining negatively affected the overall sum amino acid profile ($P < 0.05$). In particular, taurine, histidine, cysteine, methionine and available (reactive) lysine were affected by brining with the highest percent loss accounted for taurine (56%) followed by methionine (17%), cyteine (11%), and histidine (10%) ($P < 0.05$). In contrast, there was numerical 5 and 6% increase in proline and glycine respectively, whereas alanine and arginine were not affected by brining ($P > 0.05$; Table 3).

Table 3. Changes in amino acid concentrations in tambaqui fish due to brining and drying at seven different temperatures (30, 35, 40, 45, 50, 55 and 60°C).

**Amino acids (g/kg CP)**	**Not salted**	**salted**	**Total**	**Relative % Change**	**SEM**	***p***	
						**salted**	**temp**
Taurine	7.10	3.10	5.11	-56	0.5	<0.001	0.324
Asparagine + Aspartic acid	56.8	54.9	55.4	-3	0.9	0.268	0.671
Threonine	27.0	26.3	26.5	-3	0.4	0.311	0.965
Serine	25.0	24.9	24.8	0	0.2	0.807	0.777
Glutamine + Glutamic acid	91.4	88.3	89.2	-3	1.3	0.247	0.851
Glycine	75.7	80.2	79.3	6	2.5	0.361	0.750
Alanine	51.1	51.0	51.2	0	0.7	0.961	0.636
Valine	30.8	29.2	29.8	-5	0.5	0.062	0.642
Isoleucine	26.0	24.6	24.9	-5	0.5	0.162	0.838
Leucine	44.3	41.8	42.5	-6	0.9	0.134	0.855

Tyrosine	17.9	16.5	16.9	-8	0.5	0.098	0.952
Phenylalanine	24.5	23.4	23.8	-5	0.4	0.134	0.874
Histidine	15.5	14.0	14.6	-10	0.4	0.054	0.400
Lysine	51.1	47.4	48.6	-7	1.1	0.070	0.863
Arginine	48.3	48.3	48.5	0	0.5	0.999	0.464
Proline	47.5	49.9	49.5	5	1.3	0.356	0.660
Cysteine	5.10	4.40	4.60	-11	0.1	0.016	0.746
Methionine	14.7	12.1	13.3	-17	0.4	<0.001	0.675
Tryptophan	5.10	4.60	4.71	-8	0.2	0.229	0.698
Reactive lysine	52.0	46.4	48.5	-11	1.2	0.004	0.851
% Reactive lysine	102.2	97.9	99.9	-4	1.8	0.306	0.760
Sum AA	665	645	653	-3	6.87	0.017	0.621

CP= Crude protein; AA= Amino acid; SEM= Standard error of the means; temp= temperature.
Values represent mean and standard error of the means based on three replicates per treatment and seven treatments; n = 48.
[a]Percentage of reactive lysine in the total protein.
[b]Percentage of reactive lysine in the total lysine.

Multivariate classification of variables

Principal component analysis was computed on the collective factors obtained to appraise the relationships of nutrient profiles based on their responses towards brining and drying methods. By examining at the scree plot and eigenvalues and looking for a point at which the proportion of variance explained by each subsequent principal component drops off, an elbow in the screen plot, the most explaining two components 1 (44.8%) and 2 (18.9%) were considered and a biplot PCA generated.

In line, there were no associations between nutrient profiles and drying temperature, while several associations were observed with brining. Negative association was seen between brining, Na and ash on one side of component 1 and most other nutrients on the other. However, positive association between the brining proxies (Na, ash) and amino acids prominent in connective tissue (glycine, proline, arginine, alanine) were typically seen clustered together right of the component. The more central position of Zn, Fe, fat, and available Lys point to their

low response to brining and temperature. More importantly, temperature itself was positioned centrally in the plot, emphasizing the absence of drying temperature effects on the study parameters (Fig 2).

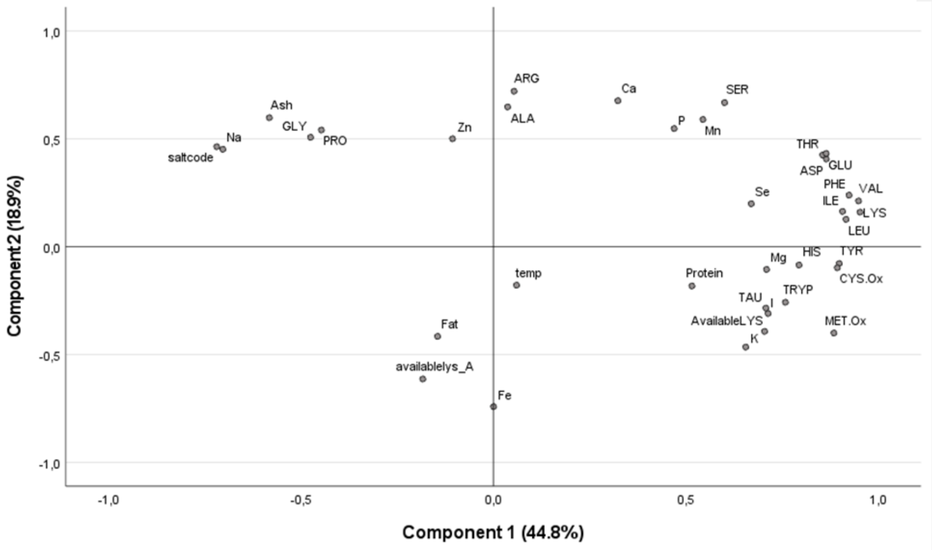

Fig 2. Principal component analysis classification of nutrient profiles based on their response to brining and temperature. Analyses represent principal component analysis (PCA) of evaluated nutrient values (expressed as percentages of the sum of total) after brining and drying of whole tambaqui fish samples (n = 48). AvailableLYS, is the percentage of reactive lysine in the total protein; availablelys_A, is the percentage of reactive lysine in the total lysine; saltcode, refers to brining; temp, is the drying temperature.

Discussion

The present study evaluated the effect of brine salting (15% *w/w*) prior to drying at different temperatures on the nutrient profiles of tambaqui fish. Brining induced a prominent effect on macronutrient and micronutrient profiles of tambaqui fish.

In this study, brining induced pronounced losses of specific amino acids and minerals, irrespective of drying temperature. Gill et al. [48] found that salting enhanced the aggregation of myosin in cod, yet only above a drying temperature of 50°C. Marínez-Alvarez and Gómez-

Guillén [33] indicated that there is a notable loss of soluble muscle proteins (particularly, actin and myosin) by osmosis in salting (NaCl) at pH 6.5 on cod muscle (*Gadus morhua*). Differences between studies can evolve from a wide range of conditions used in the drying process, including the drying type, drying temperature and duration, relative humidity, air velocity and the specific characteristics of the product dried (species and size of the fish). Although comparison with existing literature does not allow us to pinpoint the factors that protected from a drying temperature effect, this study suggests that brining is associated with nutrient losses. In the present study, a total of 6% crude protein was lost due to the brining stress. In fact, salt concentration and salting duration can modulate the effect of brining on muscular protein loss in fish [28-30]. The solubility of muscle proteins often start to decrease with increasing salt concentration due to protein aggregation [24] and/or altered enzymatic activities [49]. In the present study, however, an acceptable (15% *w/w*) salt was used for brining [31].

The amino acid analysis in this study demonstrated that the salt-induced protein loss was not an overall effect, but reduced specific amino acids, taurine, histidine, cysteine, methionine and available lysine. Taurine is not involved in protein synthesis but often accumulates in muscle tissues of fish and has osmolytic properties [50,51]. Hence, the loss in taurine seem a logical reaction to the osmotic stress from brining. This taurine loss can be important since its role in health has been demonstrated for instance in infants [52] and it is also the main amino acid to form conjugated bile acids in chickens [53].

The tendency to a lower histidine concentration due to brining can be seen in the same light. Apart from its protein-bound appearance, histidine is stored in muscular tissue as either free histidine (acetylated or not) or bound to mainly beta-alanine (hence constituting dipeptides, such as carnosine or anserine); this storage of histidine occurs throughout the animal kingdom [54]. Shiau [55] reported that histidine can easily get released from muscle tissues during stress conditions, such as starvation. Significant losses of the free amino acids histidine, taurine and

glycine were reported in salted milkfish [32]. Histidine is involved in the regulation and metabolism of essential trace minerals, formation of enzymes, induction of the immune system through the production of histamine and protects brain cells through the formation of metallothionein among others [56].

Cysteine and methionine are sulphur-containing amino acids and quite reactive to processing, in that they could either get directly affected by the ionic brine solution or can possibly make cross-links that reduces their tissue availability [32]. Kumazawa [57] reported that brining may cause cross-linking reactions of muscle myosin heavy chains and form ε-(γ-glutamyl)lysine through transglutaminase. There was no effect on the percentage of reactive lysine (% available-lysine), meaning that both salting and drying did not induce Maillard reactions that affect lysine availability. The relative decrease in the total lysine likely explains the decreased percentage of available lysine in the current study.

Four specific amino acids in the principal component analysis separated from the negative association between brining and the other amino acids: alanine and arginine were not affected, while glycine and proline were even positively associated with brining. These amino acids are prominent in the skeleton and in scales, which is a strong indication that the highest protein losses were from muscle and other soft tissues [58]. Other authors not always report similar changes in amino acid pattern: a sigmoid initial increase (up to 9 h) and final slight decrease (48 h) of alanine, glutamine and lysine was reported in salted milkfish [32]; alanine, glycine, lysine and histidine increased, when salted fish was dried [59]. The increase in these amino acid concentrations could be due to the decrease in the proportion of the extracted total lipids present as polyunsaturated fatty acids as depicted by Yankah [59]. Fat is a hydrophobic molecule and can act as a physical barrier to limit salt (NaCl) and water transport across the muscle tissue [23]. In fact, the lipid content was higher for the fish in our study (28.71%) although fat concentration was not affected by both treatments. Of the affected amino acids by brining,

taurine and lysine are polar hydrophilic where are methionine and cysteine have basic hydrophobic property [47,50,51].

The specific loss of soft tissue from the fish is not only supported by the amino acid data, but also witnessed by the change in mineral profile. The considerable loss of the microminerals Se and I and the macrominerals K and Mg following brining reflects the presence of these minerals in soft tissue [60]. Selenium typically occurs as replacement of sulphur in sulphur-containing amino acids (cysteine and methionine) in soft tissues. The decrease in methionine and cysteine seen in our study, therefore, fits the observation of decreased selenium. In contrast, the typical skeletal minerals, such as Ca, P and Mg, and those that are deposited in scales and fins such as Zn and Fe, were not affected. Potassium is a strong electrolyte that could disappear in the brine solution or get filtered out in exchange with the added sodium. The loss of water due to salt was explained as the main cause for leaching of these macrominerals in the solution during brining. The decrease in K concentration agrees with Polak-Juszczak [61] and Bakhiet [62] from salted fish. The latter study remarkably reported a salt-induced increase in iodine concentration, most likely evolving from salt impurities as proposed by the authors. It is not clear how these differences with our study can be explained, but we used a purified source of salt, hence excluding potential unintended supply of microminerals to the fish tissues through brining.

Drying is an energy intensive process, which can use about 10% or more of the total energy consumed during processing. In the present study, although our aim was not to quantify the drying efficiency, we conjointly were aiming to consume less energy while drying samples. This was achieved by increasing the sample surface area of exposure, avoiding surface contact by putting beads, improved equipment insulation during and between drying, supply of a constant air ventilation at 1.0 m/s during drying, and minimizing the timing between dryings [63].

Undeniably, brining has substantially increased Na and ash concentrations. This has been observed in most studies on brining of fish [61,62]. This enrichment with salt through brining must be considered when formulating diets but, more importantly, our results demonstrate that brining may not be the best choice to maintain the nutritive value of fish. Evidently, we must acknowledge that nutritive value is not the only criterion to pick a preservation method for fish: for the reduction of the pathogenic load in fish products, salt addition may still be a valuable technique that goes, however, at the expense of nutritive value as shown in our study. A comforting finding is that the wide range in drying temperature evaluated in our study had no noticeable impact on the nutritive value of the fish. This implies that drying of fish under uncontrolled environmental temperature seems a suitable option in rural areas to preserve the nutritive value of (tambaqui) fish.

5. Conclusions

This research shows that drying temperature has no effect on nutrient composition of the studied fish which could be encouraging for drying of fish within the drying temperature range used in this studyunder – less controlled – rural tropical conditions. Brining, however, reduced some important trace elements such as Se and I, and lead to loss of muscle crude protein, with the concomitant amino acids. Brining also increased the leaching of K, I, and taurine. The formation of Maillard products was not promoted by both drying and brining treatments, as demonstrated through unaltered reactive lysine proportions. Therefore, drying without brining may better preserve the nutritive value of tambaqui fish. However, as a recommendation to the industry sector, it is suggested that the final products are further evaluated for any pathogen of economic or public health importance.

Acknowledgments

The authors acknowledge Herman De Rycke for the proximate analysis.

References

1. Pawar SM, Sonawane SR. Fish muscle protein highest source of energy. Int J Biodivers Conserv. 2013; 5:433–435. doi: 10.5897/IJBC12.043.
2. di Domenico CNB. Fish drying: A review. Rev Bras Eng Pesca. 2014; 7:1–18. doi: 10.18817/repesca.v7i1.1061.
3. Marta M, Francisca H, Robert S. Fatty acids profile in canned tuna and sardine after retort sterilization and high pressure thermal sterilization treatment. J Food Nutr Res. 2015; 54:171–178. doi: hdl.handle.net/10261/114599.
4. Abraha B, Admassu H, Mahmud A, Tsighe N, Shui XW, Fang Y. Effect of processing methods on nutritional and physico-chemical composition of fish: a review. MOJ Food Process Technol. 2018; 6:376-82. doi: 10.15406/mojfpt.2018.06.00191.
5. Sampels S. The effects of processing technologies and preparation on the final quality of fish products. Trends Food Sci Technol. 2015; 44:131-146. doi: 10.1016/j.tifs.2015.04.003.
6. Kumar MS. Sustainable management of fisheries and aquaculture for food security and nutrition: policies requirements and actions. Agric Res. 2014; 3:97-103.
7. da Silva JAM, Pereira-Filho M, Oliveira-Pereira MI. Seasonal variation of nutrients and energy in tambaquí's (*Colossoma macropomum* Cuvier, 1818) natural food. Braz J Biol. 2000; 60:4. doi: 10.1590/s0034-71082000000400009
8. Woynárovich A, van Anrooy R. Field guide to the culture of tambaquí Colossoma macropomum, Cuvier, 1816. In: FAO Fisheries and Aquaculture Technical Paper. 2019; 624:21-30.
9. Arantes ML, Freitas CEC. Effects of fisheries zoning and environmental characteristics on population parameters of the tambaqui (*Colossoma macropomum*) in managed

floodplain lakes in the Central Amazon. Fish Manag Ecol. 2016; 23:133–143. doi: 10.1111/fme.12164

10. Val AL, de Oliveira AM. *Colossoma macropomum*—A tropical fish model for biology and aquaculture. J Exp Zoo Part A: Ecol Integ Phys. 2021; 335:761-70. doi: 10.1002/jez.2536

11. Barat, J.M., Rodríguez-Barona, S., Andrés, A., & Fito, P. (2002). Influence of increasing brine concentration in the cod salting process. J Food Sci. 65(7):1922–1925. doi: 10.1111/j.1365-2621.2002.tb08747.x.

12. Gomes LC, Silva CR. Impact of pond management on tambaquí, *Colossoma macropomum* (Cuvier), production during growth-out phase. Aqua Res. 2009; 40:825–832. doi: 10.1111/j.1365-2109.2009.02170.x

13. Sandre LCG, Buzollo H, Nascimento TMT, Neira LM, Jomori RK, Carneiro DJ. Productive performance and digestibility in the initial growth phase of tambaquí (*Colossoma macropomum*) fed diets with different carbohydrate and lipid levels. Aqua Rep. 2017; 6:28–34. doi: 10.1016/j.aqrep.2017.02.003

14. Heijnen C, Boogaard BVD, Maas van Berkel B. Preservation of fish and meat. Agrodok. 2004; Pp. 26-35.

15. Miladi H, Chaieb K, Bakhrouf A, Elmnasser A, Ammar E. Freezing effects on survival of Listeria monocytogenes in artificially contaminated cold fresh-salmon. Annals Microbiol. 2008; 58:471-476.

16. Mahmoud BSM, Yamazaki K, Miyashita K, Shin II, Suzuki T. A new technology of fish preservation by combined treatment with electrolysed NaCl solutions and essential oil compounds. Food Chem. 2006; 99:656-662. doi: 10.1016/j.foodchem.2005.08.037.

17. Djendoubi N, Boudhrioua N, Bonazzi C, Kechaou N. Drying of sardine muscles: Experimental and mathematical investigations. Food Bioprod Process. 2009; 87:115-123. doi: 10.1016/j.fbp.2008.07.003.

18. Birkeland S, Skåra T, Bjerkeng B, Rørå AMB. Product yield and gaping in cold-smoked Atlantic salmon (*Salmo salar*) fillets as influenced by different injection-salting techniques. J Food Sci. 2003; 68:1743-1748.

19. Guizani N, Rahman MS, Al-Ruzeiqi MH, Al-Sabahi JN, Sureshchandran S. Effects of brine concentration on lipid oxidation and fatty acids profile of hot smoked tuna (*Thunnus albacares*) stored at refrigerated temperature. J Food Sci Technol. 2014; 51:577-582. doi: 10.1007/s13197-011-0528-4.

20. Hall G. Preservation by curing (drying, salting and smoking). In: G. Hall (Ed.), Fish processing sustainability and new opportunities, West Sussex: Wiley-Blackwell. 2011; Pp. 51-76.

21. Kituu GM, Shitanda D, Silayo D, Odote P, Bongyereire J. Effect of brining on the drying rate of tilapia (*Oreochromis niloticus*) in a solar tunnel dryer. Fish Aqua Cluster Proceed. 2000; 88-98. http://ir.mksu.ac.ke/handle/123456780/4889.

22. Binici A, Kaya GK. Effect of brine and dry salting methods on the physicochemical and microbial quality of chub (*Squalius cephalus* Linnaeus, 1758). Food Sci Technol. 2018; 38:66–70. doi: 10.1590/1678-457X.15717.

23. Gallart-Jornet L, Barat JM, Rustad T, Erikson U, Escriche I, Fito P. A comparative study of brine salting of Atlantic cod (*Gadus morhua*) and Atlantic salmon (*Salmo salar*). J Food Eng. 2007; 79:261–270. doi: 10.1016/j.jfoodeng.2006.01.053.

24. Nguyen MV, Thorarinsdottir KA, Gudmundsdottir A, Thorkelsson G, Arason S. The effects of salt concentration on conformational changes in cod (*Gadus morhua*) proteins

during brine salting. Food Chem. 2011; 125:1013–1019. doi: 10.1016/j.foodchem.2010.09.109.

25. Rust RE, Knipe CL. Sausage casings. In: Devine C, Dikeman M, editors. Encyclopedia of Meat Sciences. Academic Press: Oxford; 2014. pp. 235–240.

26. Thorarinsdottir KA, Arason S, Bogason S, Kristbergsson K. The effects of various salt concentrations during brine curing of cod (*Gadus morhua*). Int J Food Sci Technol. 2004; 39(1):79–89. doi: 10.1046/j.0950-5423.2003.00757.x.

27. Thorarinsdottir KA, Arason S, Sigurgisladottir S, Valsdottir T, Tornberg E. Effects of different pre-salting methods on protein aggregation during heavy salting of cod fillets. Food Chem. 2011; 124(1):7–14. doi: 10.1016/j.foodchem.2010.05.095.

28. Barat JM, Rodríguez-Barona S, Andrés A, Fito P. Influence of increasing brine concentration in the cod salting process. J Food Sci. 2002; 67(5):1922–1925. doi: 10.1111/j.1365-2621.2002.tb08747.x.

29. Larsen R, Elvevoll EO. Water uptake, drip losses and retention of free amino acids and minerals in cod (*Gadus morhua*) fillet immersed in NaCl or KCl. Food Chem. 2008; 107(1):369–376. doi: 10.1016/j.foodchem.2007.08.031.

30. Thorarinsdottir KA, Arason S, Geirsdottir M, Bogason SG, Kristbergsson K. Changes in myofibrillar proteins during processing of salted cod (*Gadus morhua*) as determined by electrophoresis and differential scanning calorimetry. Food Chem. 2002; 77(3):377–385. doi: 10.1016/S0308-8146(01)00349-1.

31. FSSA. Fish and fish products regulations. In: Gaur VN, editors. Food Safety and Standards Regulation Authority (FSSA). New Delhi: The gazette of India, 2011. pp. 366-369.

32. Sannaveerappa T, Ammu K, Joseph J. Protein-related changes during salting of milkfish (*Chanos chanos*). J Sci Food Agric. 2004; 84(8):863–869. doi: 10.1002/jsfa.1682.

33. Marínez-Alvarez O, Gómez-Guillén MC. The effect of brine composition and pH on the yield and nature of water-soluble proteins extractable from brined muscle of cod (*Gadus morhua*). Food Chem. 2005; 92(1):71–77. doi: 10.1016/j.foodchem.2004.04.049.

34. Ockerman HW, Basu L. Preservation methods of animal products. In: Dikeman M, Devine C, editors. Encyclopedia of Meat Sciences. London: Oxford Elsevier Ltd.; 2014. pp. 78–83. doi: 10.1016/B978-0-12-384731-7.00207-5.

35. Kim JC, Mullan BP, Pluske JR. Prediction of apparent, standardized, and true ileal digestible total and reactive lysine contents in heat-damaged soybean meal samples. J Anim Sci. 2012; 90:137–139. doi: 10.2527/jas.53819.

36. Lantry BF, O'Gorman R. Drying temperature effects on fish dry mass measurements. J Great Lakes Res. 2004; 33:606–616. doi: 10.3394/0380-1330(2007)33[606:DTEOFD]2.0.CO;2.

37. Bastos JR. Influência da secagem sobre algumas propriedades físico-químicas do músculo do cação branco, Carcharhynus porosus Ranzani (in portuguess, with english abstract). Arquivos de Ciências do Mar. 1997; 17:77–87. Available from: http://www.repositorio.ufc.br/handle/riufc/1403

38. Pacheco N, Garnica-Gonzalez M, Gimeno M, Bárzana E, Trombotto S, David L, et al. Structural characterization of chitin and chitosan obtained by biological and chemical methods. Biomacromolecules. 2011; 12:3285–3290. doi: 10.1021/bm200750t.

39. Frisso RM, de Matos FT, Moro GV, de Mattos BO. Stocking density of Amazon fish (*Colossoma macropomum*) farmed in a continental neotropical reservoir with a net cages system. Aquaculture. 2020; 529:p.735702. doi: 10.1016/j.aquaculture.2020.735702

40. Lewbart GA. Euthanasia of ectotherms. In: Fowler's zoo and wild animal medicine current therapy. Elsevier. 2019; 9:357–363. doi: 10.1016/b978-0-323-55228-8.00051-5.

Field Code Changed

41. AOAC. Official Methods of Analysis of the Association of Official Analytical Chemists. 16th ed. Arlington VA; 1995.

42. AOAC. Official Methods of Analysis of the Association of Official Analytical Chemists. 17th ed. Washington DC: Gaithersburg publishing; 2001.

43. AOAC. Official Methods of Analysis of the Association of Official Analytical Chemists. 15th ed. Arlington VA; 1990.

44. ISO. Meat and Meat Products - Determination of total ash. In: International Organization for Standardization. Switzerland: ISO/936; 1998. Available from: https://www.iso.org/standard/24783.html

45. Gorsuch TT. The Destruction of organic matter. 1st ed. New York: Oxford pergamon press Ltd; 1970. pp. 40–54.

46. Hulshof TG, Bikker P, Van der Poel AFB, Hendriks WH. Assessment of protein quality of soybean meal and 00-rapeseed meal toasted in the presence of lignosulfonate by amino acid digestibility in growing pigs and Maillard reaction products. J Anim Sci. 2016; 94:1020–1030. doi: 10.2527/jas.2015-9700.

47. Moughan PJ, Gall MPJ, Rutherfurd SM. Absorption of lysine and deoxyketosyllysine in an early Maillard browned casein by the growing pig. J Agric Food Chem. 1996; 44:1520–1525. doi: 10.1021/jf950428v.

48. Gill TA, Chan TK, Phozchareon KF, Paulson AT. Effect of salt concentration and temperature on heat-induced aggregation and gelation of fish myosin. Food Res Int. 1992; 25:333–341. doi: 10.1016/0963-9969(92)90108-H.

49. Stoknes IS, Walde PM, Synnes M. Proteolytic activity in cod (*Gadus morhua*) muscle during salt curing. Food Res Int. 2005; 38*:*693–699. doi: 10.1016/j.foodres.2005.02.003.

50. Geda F, Declercq AM, Remø SC, Lourenço RWM, Janssens GPJ. The metabolic response in fish to mildly elevated water temperature relates to species-dependent

muscular concentrations of imidazole compounds and free amino acids. J Therm Biol. 2017; 65:57-63. doi: 10.1016/j.jtherbio.2017.02.004.

51. Zhang Y, Dabrowski K, Hliwa P, Gomulka P. Indispensable amino acid concentrations decrease in tissues of stomachless fish, common carp in response to free amino acid- or peptide-based diets. Amino Acids. 2006; 31(2):165–172. doi: 10.1007/s00726-006-0345-7.

52. Chesney RW, Helms RA, Christensen M, Budreau AM, Han X, Sturman JA. The role of taurine in infant nutrition. Adv Exp Med Biol. 1998; 442:463–476. doi: 10.1007/978-1-4899-0117-0_56.

53. Alzawqari MH, Al-Baadani HH, Alhidary IB, Al-Owaimer AN, Abudabos AM. Effect of taurine and bile acid supplementation and their interaction on performance, serum components, ileal viscosity and carcass characteristics of broiler chickens. S Afr J Anim Sci. 2016; 46(4):448–457. doi: 10.4314/sajas.v46i4.13.

54. Boldyrev AA, Aldini G, Derave W. Physiology and pathophysiology of carnosine. Physiol Rev. 2013; 93:1803–1845. doi: 10.1152/physrev.00039.2012.

55. Shiau CY, Pong YJ, Chiou TK, Tin YY. Effect of starvation on free histidine and amino acids in white muscle of milkfish *Chanos chanos*. Comp Biochem Physiol B: Biochem Mol Biol. 2001; 128:501–506. doi: 10.1016/S1096-4959(00)00350-X.

56. Kopeć W, Jamroz D, Wiliczkiewicz A, Biazik E, Pudlo A, Hikawczuk T, et al. Influence of different histidine sources and zinc supplementation of broiler diets on dipeptide content and antioxidant status of blood and meat. Br Poult Sci. 2013; 54:454–465. doi: 10.1080/00071668.2013.793295.

57. Kumazawa K, Seguro K, Takamura M, Motoki M. Formation of ε-(γ-glutamyl)lysine cross-link in cured horse mackerel meat induced by drying. J Food Sci. 1993; 58:1062–1064. doi: 10.1111/j.1365-2621.1993.tb06112.x.

58. Fedina IS, Georgieva K, Grigorova I. Light-dark changes in proline content of barley leaves under salt stress. Biol Plant. 2002; 45:59–63. doi: 10.1023/A:1015175802847.

59. Yankah MV, Ohshima T, Koizumi C. Effects of processing and storage on some chemical characteristics and lipid composition of a Ghanian fermented fish product. J Sci Food Agric. 1993; 63:227–235. doi: 10.1002/jsfa.2740630209.

60. Yamashita Y, Yamashita M, Iida H. Selenium Content in Seafood in Japan. Nutrients. 2013; 5:388–395. doi: 10.3390/nu5020388.

61. Polak-Juszczak L. Effects of processing methods on the content of minerals in fish products. J Elem. 2016; 21:461-470. doi: 10.5601/jelem.2015.20.3.890.

62. Bakhiet HHA, Khogali FAH, Ahmed FAM. Effect of different salt concentrations on mineral content of the fish (*Hydrocynus* spp.). J Life Sci Biomed. 2013; 3(1):01–03.

63. Pask F, Lake P, Yang A, Tokos H, Sadhukhan J. Industrial oven improvement for energy reduction and enhanced process performance. Clean Technol Environ Policy. 2017; 19:215-224. doi: 10.1007/s10098-016-1206-z.

Response to Reviewers

Comment	Response	Adaptation	Line/s
**Reviewer** #2			
Comments to the author:			
1. If the authors have adequately addressed your comments raised in a previous round of review and you feel that this manuscript is now acceptable for publication, you may indicate that here to bypass the "Comments to the Author" section, enter your conflict of interest statement in the "Confidential to Editor" section, and submit your "Accept" recommendation.<br>*All comments have been addressed.	Thank you for your feedback in relation to our previous responses.<br>We still have attempted to address all the further comments given as discussed here below.	-	-
2. Is the manuscript technically sound, and do the data support the conclusions?<br>The manuscript must describe a technically sound piece of scientific research with data that supports the conclusions. Experiments must have been conducted rigorously, with appropriate controls, replication, and sample sizes. The conclusions must be drawn appropriately based on the data presented.<br>*Yes	Thank you for your feedback in relation to our previous responses.	-	-
3. Has the statistical analysis been performed appropriately and rigorously?<br>*Yes	Thank you for your feedback in relation to our previous responses.	-	-
4. Have the authors made all data underlying the findings in their manuscript fully available?<br>The PLOS Data policy requires authors to make all data underlying the findings described in their manuscript fully available without restriction, with rare exception (please refer to the Data Availability Statement in the	Thank you for your feedback in relation to our previous responses.	-	-

manuscript PDF file). The data should be provided as part of the manuscript or its supporting information, or deposited to a public repository. For example, in addition to summary statistics, the data points behind means, medians and variance measures should be available. If there are restrictions on publicly sharing data—e.g. participant privacy or use of data from a third party—those must be specified.<br>*Yes			
5. Is the manuscript presented in an intelligible fashion and written in standard English?<br>PLOS ONE does not copyedit accepted manuscripts, so the language in submitted articles must be clear, correct, and unambiguous. Any typographical or grammatical errors should be corrected at revision, so please note any specific errors here.<br>*No	Thank you for the feedback given. We now have tried to go through the entire manuscript and correct any typographical/grammatical errors including, but not limited to, the below stated points.	-	-
6. Review Comments to the Author:			
1. The abstract lacks clarity and brevity and has ambiguity in presenting the problem statement and results.	Comment accepted. We now have rewritten the entire abstract and tried to make it more clear and understandable. As indicated in the adapted abstract, we tried to address all the points raised by the reviewer one-by-one as shown below.	Preservation of fish as diet ingredient is challenging in many tropical regions due to poor socioeconomic conditions and lack of freezing facilities. So, alternative preservation techniques could be viable to address the issue. The present study evaluated the effect of brine salting (15% *w/w*) prior to drying at different temperatures on the nutrient profiles of tambaqui fish (*Colossoma macropomum*).	17-40

		Whole fish samples (792 ± 16 g; 8 months old; n = 48) were grouped into two as brine-salted and non-salted, and treated at seven different drying temperatures of 30, 35, 40, 45, 50, 55 and 60°C for a period of 23 h each. To evaluate the impact of Maillard reaction, reactive lysine was also quantified. Drying temperature had no effect on the evaluated macro- and micro-nutrients of tambaqui fish ($P > 0.05$) while brining reduced the overall protein concentration by 6% (58.8 to 55.4 g/100 g DM; $P = 0.004$). Brining significantly reduced many amino acids: taurine by 56% (7.1 to 3.1 g/kg; $P < 0.001$), methionine 17% (14.7 to 12.1 g/kg; $P < 0.001$), cysteine 11% (5.1 to 4.4 g/kg, $P = 0.016$), and reactive lysine 11% (52.0 to 46.4 g/kg; $P = 0.004$). However, alanine, arginine, and serine were not affected by brining ($P > 0.05$). Brining also reduced the concentrations of Se by 14% (149 to 128 µg/kg DM; $P = 0.020$), iodine 38% (604 to 373 µg/kg DM; $P = 0.020$), K 42% (9.71 to 5.61 g/kg DM; $P < 0.001$) and Mg 18% (1.32 to 1.10 g/kg DM; $P = <$	

		0.001) versus an anticipated vast increase in Na by 744% (2.70 to 22.90 g/kg DM; $P < 0.001$) and ash 28% (12.4 to 16.0 g/100g DM; $P < 0.001$) concentration. Neither brining nor drying temperature induced changes in % lysine reactivity and fat content of tambaqui fish ($P > 0.05$). Agreeably, results of multivariate analysis showed a negative association between brining, Na, and ash on one side of the component and most other nutrients on the other component. In conclusion, drying without brining may better preserve the nutritive value of tambaqui fish. However, as a practical remark to the industry sector, it is recommended that the final product is further evaluated for any pathogen of economic or public health importance.	
Abstract:<br>Line 17-18: The opening sentences lack clarity and brevity and be improved to make it more catchy. The author may follow it as "Preservation of fish as diet ingredient is challenging in many tropical regions due to poor socioeconomic conditions and lack of freezing facilities. So, alternative preservation techniques could be viable to address the issue.	Comment accepted. We now have corrected the abstract's opening sentence as per the feedback given and make it more clear.	Preservation of fish as diet ingredient is challenging in many tropical regions due to poor socioeconomic conditions and lack of freezing facilities. So, alternative preservation	17-19

		techniques could be viable to address the issue.	
Abstract:<br>Line 25: ‘Profiles’?	Comment accepted. We now have removed and elaborated the word ‘profiles’.	Drying temperature had no effect on the evaluated macro- and micro-nutrients of tambaqui fish	24-25
Abstract:<br>Line 27: I acknowledge the efforts made by author to improve the article but some sound mistakes as mentioned here are major concerns to readers understanding.	Comment accepted. We now have corrected the wording in this sentence particularly.	Brining significantly reduced many amino acids: taurine…	26-27
Abstract:<br>Line 31: This type of writing (I 38%) may sounds improper.	Comment accepted. We now have extended the abbreviated iodine (I) and makes it cleared.	iodine 38%...	31
Abstract:<br>Line 35-38: 1e. The sentence can be split to make it more understandable.	Comment accepted. We now have shortened the sentence into one simple and understandable.	Agreeably, results of multivariate analysis showed a negative association between brining, Na, and ash on one side of the component and most other nutrients on the other component.	35-37
Abstract:<br>Line 35-38: ‘final products are further evaluated’ seems improper.	Comment accepted. We now have corrected the rewording.	However, as a practical remark to the industry sector, it is recommended that the final product is further evaluated…	38-39
2. The introduction section is too long, with a lot of vague sentences, and is poor in its construction.<br>*Introduction section is too lengthy and lack clarity. Please make it concise and clear.	Comment accepted. We now have gone through the entire ‘Introduction’ section, removed some sentences we believe are redundant and/or vague, and attempted to make it clearer and shorter.	Kinly, see for tracked reduced contents in the introduction section with these line numbers.	46-49<br>57-58<br>66-67<br>91-92

	We, kindly, believe that we now have tried to find the balance between the different reviewers, where one asked to have a more extensive introduction, and another that asks to reduce it.<br>Here below are the further comments given addressed in the introduction section.		
Introduction:<br>Line 48-51: Must be improved to make it understandable.	Comment accepted. To minimize the 'introduction' length and make it with flow, we have now removed this sentence and briefly stated it below paragraph.	Nutritionally, fish meat is a prominent source of high quality essential amino acids [3,4], omega-3 fatty acids such as eicosapentaenoic acid (EPA) and docosahexaenoic acid (DHA), and trace elements such as iron, selenium, iodine that are beneficial for human health [5]. Hence, good knowledge on the nutrient…	44-47
Introduction:<br>Line 54: is and species seems to be contradictory.	Comment accepted. We now have removed 'species' and corrected the sentence.	Tambaqui fish (*Colossoma macropomum*), also known as black pacu, is the main native fish farmed in…	50-51
Introduction:<br>Line 56: "species???"	Comment accepted. Now, we have corrected the wording by replacing 'tambaqui' for 'species'.	Tambaqui alone accounted for a total production…	52
Introduction:<br>Line 70-71: "Preservation is a fundamental requirement of food and food ingredients in maintaining a quality food which is a vital issue in the human health and contributes to profitability" is grammatically too poor to be understandable.	Comment accepted. The sentence has now been improved.	Food preservation is a fundamental requirement in maintaining quality food, a vital issue in human health, and contributes to profitability [14,15].	63-64

Introduction:<br>Line 81-84: Sentence needs attention ”Salting, on the other hand, involve different processes such as brining (immersion of fish meat into salt solution), kench curing (dry salting of fish fillets), pickling (longer storage of the fish with brine), and the possible injection of brine into the fish muscle all ending up in reducing product water content and hence extended shelf life [5,18-21].”	Comment accepted. We have made some amendment in this sentence and made it flow well with its succeeding paragraph “Brine salting…)	Salting, on the other hand, involves different processes techniques such as brining (the immersion of fish meat into salt solution), kench curing (dry salting of fish fillets), pickling (longer storage of the fish with brine), and the possible injection of brine into the fish muscle all ending up in extended product shelf life [5,18-21].<br>Brine salting is one of the oldest traditional and economically…	72-77
3. The material and method are against the problem statement, i.e., the use of freeze drying.	Comment acknowledged. We understand that the concern of the reviewer that freeze-drying of the final samples may have overruled the temperature effect during oven-drying. We could have analyzed the samples without freeze-drying at the end, but to evaluate eventual differences in residual moisture content, we wanted to include freeze-drying in the final step. It is well-established that freeze-drying is a suitable method to obtain moist-free samples without noticeable effects on any of the studied nutrients. That is the reason why freeze-drying is used in many industrial food processing methods. This is elaborated on in detail in Handbook of Nutritive Value of	-	-

	Processed Foods (ed. Miloslav Rechcigl) https://books.google.be/books?hl=nl&lr=&id=BRemDwAAQBAJ&oi=fnd&pg=PA45&dq=freezedrying+and+nutritive+quality&ots=AVbkHiMmA7&sig=hzN_HgTO3t72adCedyWfy2OAjdk#v=onepage&q=freezedrying%20and%20nutritive%20quality&f=false		
Materials and methods:<br>Line 139-143: Too lengthy sentences may be avoided; and also 'reared' may need correction.	Comment accepted. We now have split the long sentence into two. Also, replaced 'reared' by 'raised'.	Fingerlings of *Colossoma macropomum* were initially brought from Aqua-Koi NV farm (http://www.aqua-koi.be/documents/home.xml?lang=nl) to TRANSfarm, Belgium. Fingerlings were then raised in a recirculating aquaculture system (RAS) with a weekly basis 30% water renewal (20L/day) for about 7 months period before being subject to the current study.	127-131
Materials and methods:<br>Line 158-162: May be divided into parts to make it more clear: "The study was conducted in accordance with the Declaration of Helsinki and directive 2010/63/EU of the European parliament and of the council on the protection of animals used for scientific purposes, and the wellbeing of the cultured tambaqui fish before being subjected to the experiment was assessed and approved by the institutional review board of TRANSfarm, KU Leuven, lab accreditation (KU/LA-1210616)."	Comment accepted. We now have split the long sentence into two and make it more clear.	The study was conducted in accordance with the Declaration of Helsinki and directive 2010/63/EU of the European parliament and of the council on the protection of animals used for scientific purposes. The wellbeing of the cultured tambaqui fish before being subjected to the experiment was assessed and approved by the	146-150

		institutional review board of TRANSfarm, KU Leuven, under laboratory accreditation of KU/LA-1210616.	
Materials and methods:<br>Line 170: The study is interesting and have a role in managing food security but some contradictions exists like storing at -20 degrees needs a lot of energy or chemical refrigerant while this was planed as community based practice.	Comment is highly acknowledged. We agree that energy is being costing in this case. We use it as part of the study protocols and taking the time required to process the drying of the entire 96 fish samples.<br>Seeing this important, we have previously acknowledged this 'energy costs and efficiency in the discussion' section further.	Drying is an energy intensive process, which can use about 10% or more of the total energy consumed during processing. In the present study, although our aim was not to quantify the drying efficiency, we conjointly were aiming to consume less energy while drying samples. This was achieved by increasing the sample surface area of exposure, avoiding surface contact by putting beads, improved equipment insulation during and between drying, supply of a constant air ventilation at 1.0 m/s during drying, and minimizing the timing between dryings [63].	403-409
4. The result and discussion are well presented and have clarity.	Thank you for your feedback in relation to our previous responses.	-	-
5. The conclusion is improved compared to previous one and addresses the problem statement.	Thank you for your feedback in relation to our previous responses.	-	-
**Reviewer #4**			
1. If the authors have adequately addressed your comments raised in a previous round of review and you feel that this manuscript is now acceptable for	Thank you for your feedback in relation to our previous responses.	-	-

publication, you may indicate that here to bypass the “Comments to the Author” section, enter your conflict of interest statement in the “Confidential to Editor” section, and submit your "Accept" recommendation.<br>*No response.	We now have tried to further go through the entire manuscript and address further comments given including any typographical or grammatical errors as discussed here below.		
2. Is the manuscript technically sound, and do the data support the conclusions?<br>The manuscript must describe a technically sound piece of scientific research with data that supports the conclusions. Experiments must have been conducted rigorously, with appropriate controls, replication, and sample sizes. The conclusions must be drawn appropriately based on the data presented.<br>*Yes	Thank you for your feedback in relation to our previous responses.	-	-
3. Has the statistical analysis been performed appropriately and rigorously?<br>*Yes	Thank you for your feedback in relation to our previous responses.	-	-
4. Have the authors made all data underlying the findings in their manuscript fully available?<br>The PLOS Data policy requires authors to make all data underlying the findings described in their manuscript fully available without restriction, with rare exception (please refer to the Data Availability Statement in the manuscript PDF file). The data should be provided as part of the manuscript or its supporting information, or deposited to a public repository. For example, in addition to summary statistics, the data points behind means, medians and variance measures should be available. If there are restrictions on publicly sharing data—e.g.	Thank you for your feedback in relation to our previous responses.	-	-

participant privacy or use of data from a third party—those must be specified.<br>*Yes			
5. Is the manuscript presented in an intelligible fashion and written in standard English?<br>PLOS ONE does not copyedit accepted manuscripts, so the language in submitted articles must be clear, correct, and unambiguous. Any typographical or grammatical errors should be corrected at revision, so please note any specific errors here.<br>*Yes	Thank you for your feedback in relation to our previous responses.	-	-
6. Review Comments to the Author:			
Line 27: Please change “other while” to “other, while”.	Comment accepted. We now have corrected this sentence and make short as well in line to other reviewers comment.	…component and most other nutrients on the other component.	37
Line 142: Please change “20L/day” to 20 L/day”. Kindly, note that there is a space between a figure and its unit.	Comment accepted. We have tried to address this here and throughout the manuscript.	water renewal (20 L/day) for…	130
Line 145: Please change “well aerated” to “well-aerated”.	Comment accepted and corrected.	bio-filters, well-aerated with a constant temperature	133-134
Line 149: Please change “a 12 h” to “a 12-h”.	Comment accepted and corrected.	after a 12-h fasting…	137
Line 153: Please change “were” to “was”.	Comment accepted and corrected.	water was elevated out of the tanks…	141
Line 155: Please change “the” to “an”.	Comment accepted and corrected.	in the bucket with an anesthetic agent…	143
Line 155: Please change “1mL:5L” to “1 mL:5 L”.	Comment accepted and spaces added between figure and unit.	(1 mL:5 L, Anest Fish$^{©}$, Germany).	143-144

Line 166: Please change “veterinary and biosciences” to “Veterinary and Biosciences”.	Comment accepted and wording now corrected.	the Department of Veterinary and Biosciences, Ghent University	154
Line 186: Please change "try" to "tray".	Comment accepted. We now have corrected the wording.	…at one fish per tray, into…	174
Lines 197-201: This highlighted sentence in these lines is too long. Please separate it into two thus "Samples were then kept at -21°C overnight and finally freeze-dried (CoolSafe™, SCANVAC, Denmark) for a period of 16 h each to a stable weight, uniformly ground (2.0 mm sieve). Subsequently, the samples were homogenized using a grinding machine (De´Longhi™, KG48) at 12× speed and then individually kept in closable plastic containers at 4°C until analyzed [22]."	Comment accepted. By now, we have split the long sentence into two separate sentences.	Samples were then kept at -21°C overnight and finally freeze-dried (CoolSafe™, SCANVAC, Denmark) for a period of 16 h each to a stable weight, and uniformly ground (2.0 mm sieve). Subsequently, the samples were homogenized using a grinder machine (De´Longhi™, KG48) at 12× speed and then individually kept in closable plastic containers at 4°C until analyzed [22].	185-189
Line 235: Please use a past tense for "are often" and specify the number of times the visual inspection was performed.	Comment accepted. We now have addressed this.	As quality control protocols, visual inspection of pump tubing at regular intervals (every 30 min) and replacing between samples were often made to increase the precision measurements.	221-223
Line 236: Please change "spread chamber and mobilizer" to "spray chamber and nebulizer".	Comment accepted. We now have changed the wording.	Similarly, regular inspection and cleaning of the injector tube, spray chamber and nebulizer, and cleaning…	223-224
Line 237: Please change "Blanc" to "Blank".	Comment accepted. We now have changed the wording.	Blank solutions with similar matrix…	225-226
Line 242: Please change "Sulphur containing" to "Sulphur-containing".	Comment accepted. We now have changed the wording.	Sulphur-containing amino acids were measured…	231

Line 278: Please change "Proximate analysis" to "Proximate composition".	Comment accepted. We now have changed the wording.	Proximate composition	267
Line 325: Please change “temperature while” to “temperature, while”.	Comment accepted. We now have added the comma.	drying temperature, while several associations were…	314-315
Line 350: Please change "process including" to "process, including".	Comment accepted. We now have added the comma.	the drying process, including the drying type…	339-340
Line 369: Please change “dipeptides such as” to “dipeptides, such as”.	Comment accepted. We now have added the comma.	dipeptides, such as carnosine or anserine…	358-359
Line 372: Please change “conditions such as” to “conditions, such as”.	Comment accepted. We now have added the comma.	during stress conditions, such as starvation	360-361
Line 379: Please change "reduces" to "reduce".	Comment accepted. We now have changed the wording.	make cross-links that reduce their…	368
Line 386: Please change "affected while" to "affected, while".	Comment accepted. We now have added the comma.	were not affected, while glycine and proline…	375-376
Line 392: Please change "increased when" to "increased, when".	Comment accepted. We now have added the comma.	lysine and histidine increased, when salted fish was dried [59].	381
Lines 394-396: Sequel to the highlighted statement in these lines, the authors can further explain which class of amino acids (whether hydrophobic, polar, acidic, basic, neutral) was most affected by brining.	Comment accepted. We now have addressed this.	Fat is a hydrophobic molecule and can act as a physical barrier to limit salt (NaCl) and water transport across the muscle tissue [23]. In fact, the lipid content was higher for the fish in our study (28.71%) although fat concentration was not affected by both treatments. Of the affected amino acids by brining, taurine and lysine are polar hydrophilic where are methionine and cysteine have basic hydrophobic property [47,50,51].	383-388
Line 404: Please change "minerals such as" to "minerals, such as".	Comment accepted. We now have added the comma.	the typical skeletal minerals, such as Ca…	393

Line 414: Please change "process which" to "process, which".	Comment accepted. We now have added the comma.	Drying is an energy intensive process, which can use…	403
Lines 415-419: The sentence in lines 415-419 is too long. Please split it into two.	Comment accepted. We now have split this sentence into two sentences.	In the present study, although our aim was not to quantify the drying efficiency, we conjointly were aiming to consume less energy while drying samples. This was achieved by increasing the sample surface area of exposure, avoiding surface contact by putting beads, improved equipment insulation during and between drying, supply of a constant air ventilation at 1.0 m/s during drying, and minimizing the timing between dryings [63].	404-409
Lines 428-430: The authors of this study, used controlled temperatures. So, how can they claim that "This implies that drying of fish under uncontrolled environmental temperature seems a suitable option in rural areas to preserve the nutritive value of (tambaqui) fish"? If Maillard reaction products are formed between the amino acid residues and reducing sugars in the fish due to a high "uncontrolled environmental temperature", can this claim (that is, the preservation of the nutritive value of the fish) still hold?<br>Please note that Maillard reaction products' formation can increase with increasing drying temperature. You may refer to https://doi.org/10.1016/j.fshw.2019.03.012	Comment accepted. We absolutely agree with the comments given here. Hence, we have now removed the entire sentence from the paragraph.	… A comforting finding is that the wide range in drying temperature evaluated in our study had no noticeable impact on the nutritive value of the fish.	416-418
Lines 432-434: Sequel to the preceding point (line: 428-430) of reasoning, please be cautious in the first sentence	Comment accepted. Following the removal of the above stated sentence (428-430), We now have corrected the	This research shows that drying temperature has no effect on nutrient composition of the studied	420-422

(lines 432-435) in the conclusion. I suggest adding "within the drying temperature range used in this study".	sentence by adding the suggested phrase.	fish which could be encouraging for drying of fish within the drying temperature range used in this study.	
**Academic Editor:**			
Please include the following items when submitting your revised manuscript:<br>• A rebuttal letter that responds to each point raised by the academic editor and reviewer(s). You should upload this letter as a separate file labeled 'Response to Reviewers'.<br>• A marked-up copy of your manuscript that highlights changes made to the original version. You should upload this as a separate file labeled 'Revised Manuscript with Track Changes'.<br>• An unmarked version of your revised paper without tracked changes. You should upload this as a separate file labeled 'Manuscript'.	Comment accepted. We now have addressed the second round reviews and re-submitted the three items accordingly.	Kindly, see attachments.	-
If you would like to make changes to your financial disclosure, please include your updated statement in your cover letter.	Comment acknowledged. No changes are made regarding financial disclosure.	-	-
If applicable, we recommend that you deposit your laboratory protocols in protocols.io to enhance the reproducibility of your results.	Not applicable.	-	-
Journal requirements:			
Please review your reference list to ensure that it is complete and correct. If you have cited papers that have been retracted, please include the rationale for doing so in the manuscript text, or remove these references and replace them with relevant current references. Any changes to the reference list should be mentioned in the	Comment acknowledged. We now have rechecked the reference and make sure its correct and complete.<br>No changes have been made to the reference list.		

rebuttal letter that accompanies your revised manuscript. If you need to cite a retracted article, indicate the article's retracted status in the References list and also include a citation and full reference for the retraction notice.	No paper was retracted among the listed references.		
Additional editor comments:			
The paper in its current state is unsuitable for publication; it requires minor revisions in accordance with the reviewers' suggestions. Please carefully attend to the reviewers' comments and ensure you do thorough grammar checks, as there are a few errors.	Comment acknowledged. We now have, to the best of our knowledge, tried to address the reviewers' comments including grammar checks and errors as well.	Kinly, see for the detailed point-by-point reflections given above.	-
While revising your submission, please upload your figure files to the Preflight Analysis and Conversion Engine (PACE) digital diagnostic tool, https://pacev2.apexcovantage.com/. PACE helps ensure that figures meet PLOS requirements. To use PACE, you must first register as a user. Registration is free. Then, login and navigate to the UPLOAD tab, where you will find detailed instructions on how to use the tool. If you encounter any issues or have any questions when using PACE, please email PLOS at figures@plos.org. Please note that Supporting Information files do not need this step.	Comment accepted. We now have opened a PACE account and uploaded the figures and checked for the PLOS figures fulfilling the requirements.		
