# Supplementary material for: Impact of brining and drying processes on the nutritive value of tambaqui fish (Colossoma macropomum)
Source: PLoS One. 2024 Apr 16;19(4):e0299926. doi: 10.1371/journal.pone.0299926 (PMC11020844; doi:10.1371/journal.pone.0299926)
Supplement: Attachment — Submitted filename: Comments.docx [file pone.0299926.s006.docx]

**Comments to editor**

Hi Editor Journal

In the current manuscript the authors have studied "A laboratory simulation of the impact of brining and drying as accessible preservation strategies under tropical rural conditions on the nutritive value of tambaqui fish". This is a good research work and need of the hour. I recommend this work to be published in your esteemed journal.

Thanks
