# Supplementary material for: Impact of brining and drying processes on the nutritive value of tambaqui fish (Colossoma macropomum)
Source: PLoS One. 2024 Apr 16;19(4):e0299926. doi: 10.1371/journal.pone.0299926 (PMC11020844; doi:10.1371/journal.pone.0299926)
Supplement: Attachment — Submitted filename: Response to Reviewers.docx [file pone.0299926.s009.docx]

**Response to Reviewers**

| **Comment** | **Response** | **Adaptation** | **Line/s** |
| --- | --- | --- | --- |
| **Reviewer** #**1** | | | |
| 1. Is the manuscript technically sound, and do the data support the conclusions?  *Yes | Thank you for your comments. | - | - |
| 2. Has the statistical analysis been performed appropriately and rigorously?  *No  GENERAL COMMENTS  The paper is well written with strong justification and very good elaboration in methods. However some of the sections such as data analysis is not well elaborated. The types of statistics used needs to be appropriate to answers the objective of the research. | Comment acknowledged. We now have revised the ‘statistical analysis’ section and narrated it in line with our findings.  Further statistical elaborations have also been included in the ‘Results’ section under individual Tables 1, 2, and 3 and with the PCA plots as well. | Data obtained in the current study were analyzed using SPSS statistical software version 27 (IBM Corporation, Armonk, NY, USA). All data were checked for normality distributions using histogram and QQ-plots. A univariate one-way ANOVA was used with brining as fixed factor and temperature as covariate to test the hypothesis about differences between two or more mean nutrient values. Data were presented as means and standard error of the means (SEM). Statistical significance was set at P < 0.05 for any differences observed between samples. Furthermore, multivariate analysis was computed using principal component analysis (PCA) on the collective factors obtained to appraise the relationships of nutrient profiles based on their responses towards brining and drying methods, with values deemed relevant below -0.5 and above 0.5 of the componentsˈ values. | 265-274 |
|  |  | Kindly, see for footnotes under Tables 1, 2, 3 and Figure 2. | 293,  303,  315,  337 |
| 3. Have the authors made all data underlying the findings in their manuscript fully available?  *No | Comment acknowledged. We now have reviewed our data availability statement and managed to include and upload the extra results and the datasets as ‘supplementary information’ files. | - Kindly, see for ‘data availability statement corrected.  - Kindly, see for the data and extra results annexed as supplementary files with the journal. | - |
| 4. Is the manuscript presented in an intelligible fashion and written in standard English?  *Yes | Thank you for your comments. | - | - |
| **5. Review Comments to the Author:** |  |  |  |
| OTHER COMMENTS: |  |  |  |
| Abstract  In the abstract introduce the concept of brine salting is what, before “The present study evaluated…..” | Comment accepted. We now have addressed this narration in the abstract section.  The abstract is also rewritten again to meet other reviewers suggestions as well. | Preservation of fish as diet ingredient is challenging in many tropical conditions due to the absence of freezing facilities, hence warranting other preservation techniques. Brine salting is one of the oldest traditional and economically viable preservation methods. The present study evaluated the effect of… | 17-20 |
| **Introduction:**  Line 93: You could add “macronutrients and micronutrients” | Comment accepted. We now have corrected the sentence. | The present study therefore evaluated the effect of brining and a range of drying temperatures on the macronutrients and micronutrients, and mineral composition as well as on the state of Maillard reaction that may reduce lysine availability. | 133-135 |
| **Materials and Methods:** |  |  |  |
| Line 97-104: The paragraph needs to be cited. | Comment accepted. We have now put a suitable referenced citation at the end of the paragraph. | All experimental and animal care procedures were realized based upon the current written knowledge of aquaculture technologies and tambaquí fish farming. Fingerlings of Colossoma macropomum were initially brought from Aqua-Koi NV farm (http://www.aqua-koi.be/documents/home.xml?lang=nl) to TRANSfarm and reared in a recirculating aquaculture system (RAS) with a weekly basis 30% water renewal (20L/day) for about 7 months period before being subject to the current study. During their stay, fish were kept in larger aqua-tanks (100 cm × 120 cm × 40 cm) at a density of 1.2 fish/m3 sufficient enough for movements and behavioral expression. Tanks were fixed with individual bio-filters, well aerated with a constant temperature supply, 18:6 h light - dark cycle, and supplying pipes were regularly inspected and cleaned with tap water and water quality parameters were checked twice a week and water renewed accordingly [39]. | 138-148 |
| Line 120: A total of 48 tambaqui fish (792 ± 16 g; 8 months old). This number is not clear, whether say it mean weight (sd) or provide unit explanation. | Comment accepted. We now have rewritten the sentence accordingly. | A total of 48 tambaqui fish with a mean weight of 792 (± 16) g and 8 months old were collected from TRANSfarm… | 164-165 |
| Statistical analysis:  Line 213-215: Univariate analysis is done for each separate variable in a descriptive way. Here did you mean bivariate analysis for comparing variance? Please clarify | Comment acknowledged. Here we took brining as fixed factor and temperature as covariate to test our hypothesis about differences between variables. Hence, univariate was done for each separate variable. | Kindly, see statistical analysis section. |  |
| Line 264: Multivariate statistical analysis (PCA), revise either write PCA on remain with multivariate.  Multivariate classification of variables: This section is not well written and confusing. Which association did the author is presenting? PCA is not showing the association between variables. If multivariate analysis was used which type of statistical technique was applied. Just to say the association is not enough, it has to be presented with values. What makes you say this is association/no association? Please clarify. | Comment accepted. We have revised this section and added on the eigenvalues for the components most explaining.  Also, added the graph of PCA which now could explain the narrations stated more. | Result section:  Principal component analysis was computed on the collective factors obtained to appraise the relationships of nutrient profiles based on their responses towards brining and drying methods. By examining at the scree plot and eigenvalues and looking for a point at which the proportion of variance explained by each subsequent principal component drops off, an elbow in the screen plot, the most explaining two components 1 (44.8%) and 2 (18.9%) were considered and a biplot PCA generated.  In line, there were… | 320-325 |
| Line 215: “All data were presented as means of three values and standard error of the means (SEM)”. Is not clear, What does means of three value imply for? | Comment accepted. By ‘means of three values’, we meant that there were three fish samples evaluated per treatment group. We now have corrected this in the statistical analysis section, and narrated it clearly under each Table as footnotes. | Data were presented as means and standard error of the means (SEM). Statistical significance… | 268-269 |
|  |  | Kindly, see for footnotes under Tables 1, 2, and 3. | 293,  303,  315 |
| Where are the results of Principal Component Analysis (PCA)? Why did the researcher opt to use PCA in this research? | Comment acknowledged. The results of PCA are displayed in Figure 2 clearly.  We now have included the PCA plot which was initially submitted separately.  We carried out PCA so as to evaluate the associations between the nutrient profiles on the basis of their response to brining and drying methods. We have tried to elaborate this under the ‘Statistical analysis’ and ‘Results’ as well. | Kindly, see for Figure 2. Principal component analysis classification of nutrient profiles….. | 334-341 |
|  |  | Furthermore, principal component analysis (PCA) was computed using on the collective factors obtained to appraise the relationships of nutrient profiles based on their responses towards brining and drying methods, with values deemed relevant below -0.5 and above 0.5 of the componentsˈ values. | 270-273 |
|  |  | Principal component analysis was computed on the collective factors obtained to appraise the relationships of nutrient profiles based on their responses towards brining and drying methods. | 320-325 |
| **Results:** |  |  |  |
| You could better start the results section by provide a small descriptive analysis of the samples and general information about your data. | Comment accepted. We now have added a paragraph as a start of the ‘Result’ section. | Tambaqui fish samples were grouped into two as brine-salted and non-salted, and treated at seven different drying temperatures for 23 h each. Each temperature treatment was considered as sampling point and tested in triplicate (n = 3) and mean values obtained were statistically computed for all nutrient profiles evaluated. | 275-278 |
| Line 227: the title of Fig 1 is there, but figure is not shown. Place the figure header below the figure. | Comment accepted. We now have added the Figure and place the figure header below it. | Kindly, see for Figure 1. | 282-285 |
| Table 1: The table generalizes the values of macronutrients at different temp, could you try and present at each temp and we could see the variations? | Comment accepted. Such Table was not included in our manuscript as we feel it looks bulky and not easy to follow with seven different values for each preservation method and there was no statistical difference between temperature points.  The no significant effect of individual temperatures is represented in Figure 1 by taking representative nutrients and how they look with temperature points.  We now have tried to make a separate Table with all the values at different temperature points. This is now addressed as a “Supplementary files” due to larger size of the Table. | Kindly, see for supplementary files annexed. | - |
|  |  | Kindly, see for Figure 1. **Fig 1.** The effect of drying temperature on nutrient profiles of tambaqui fish. (a) gram of taurine per kg fish; (b) gram of reactive lysine per kg fish. | 282-285 |
| Table 1 needs to be improved, I can only see the values during salting, but not drying temp. | Comment accepted. In the column values, the terms **yes** and **no** refer to salted and drying temp, respectively. We agree that the naming were confusing and now we have corrected this in all Tables 1, 2, and 3 and make it clearer. As mentioned higher, the data combining all salting and drying treatments are presented in Supplementary files because of the complexity. | Kindly, see for naming corrections made in Tables 1. | 290-294 |
|  |  | Kindly, see for naming corrections made in Tables 2. | 300-305 |
|  |  | Kindly, see for naming corrections made in Tables 3. | 312-318 |
| Label below the table have to be clearly presented such as DM=Dry matter. | Comment accepted. We now have corrected the labels under all Tables 1, 2, and 3. | Table 1 label:  DM= Dry matter; SEM= standard error of the means; temp= temperature.  Values represent mean and standard error of the means based on three replicates per treatment and seven treatments; n = 48. | 292-294 |
|  |  | Table 2 label:  DM= Dry matter; SEM= standard error of the means; temp= temperature.  Values represent mean and standard error of the means based on three replicates per treatment and seven treatments; n = 48.  ^*^Values are presented in µg/kg DM. | 302-305 |
|  |  | Table 3 label:  CP= Crude protein; AA= Amino acid; SEM= Standard error of the means; temp= temperature.  Values represent mean and standard error of the means based on three replicates per treatment and seven treatments; n = 48.  ^a^Percentage of reactive lysine in the total protein.  ^b^Percentage of reactive lysine in the total lysine. | 314-318 |
| Line 239: The essential “microminerals”. Just say micronutrients or minerals | Comment accepted. We now corrected the sentence. | The essential minerals Se and I, and the……. | 296 |
| Table 2: Similar to table 1, different temp values needs to be presented in table | Comment accepted. We now have added a separate additional Table for proximate and minerals values at individual temperature points as a ‘supplementary files’. | Kindly, see for annexed ‘supplementary files’. | - |
| **Discussion:** |  |  |  |
| Start by providing the objective the research and summarizing the main findings. | Comment accepted. We now have added a paragraph involving the objectives and summary findings as a start. | The present study evaluated the effect of brine salting (15% *w/w*) prior to drying at different temperatures on the nutrient profiles of tambaqui fish. Brining induced a prominent effect on macronutrient and micronutrient profiles of tambaqui fish.  In this study, brining induced pronounced losses…. | 343-345 |
| Line 283: In our study. Use “In this study” | Comment accepted. Such sentences are now corrected here and across the manuscript. | In this study, brining induced… | 346 |
|  |  | The amino acid analysis in this study demonstrated… | 361 |
| How these literature compared here related to Tambaqui fish? Or related to included sample? Compare and contrast with relevant papers/research. | Comment accepted. We now have removed the unrelated literature contents and their references (two) and focused on the relevant ones only. | In this study, brining induced pronounced losses of specific amino acids and minerals, irrespective of drying temperature. Gill et al. [48] found that salting enhanced the aggregation of myosin in cod, yet only above a drying temperature of 50°C. Marínez-Alvarez and Gómez-Guillén [33] indicated that there is a notable loss of soluble muscle proteins (particularly, actin and myosin) by osmosis in salting (NaCl) at pH 6.5 on cod muscle (*Gadus morhua*). | 346-350 |
| Line 292: more than 60C. You don’t have that temp in your study. The final temp is 60 Centigrade. | Comment accepted. We now have removed this sentence from the ‘discussion’ paragraph. | Marínez-Alvarez and Gómez-Guillén [33] indicated that there is a notable loss of soluble muscle proteins (particularly, actin and myosin) by osmosis in salting (NaCl) at pH 6.5 on cod muscle (*Gadus morhua*). Differences between studies can evolve from… | 348-351 |
| Line 298: it is clear that brining completely overruled the drying temperature in its effect on nutrient loss. Just use the term such as “ This study suggests that brining is associated with nutrients loss…..”. | Comment accepted. We now have rewritten the sentence accordingly. | Although comparison with existing literature does not allow us to pinpoint the factors that protected from a drying temperature effect, this study suggests that brining is associated with nutrient losses. | 353-355 |
| Line 299: 6% of tissue protein. Focus on what you measure “Crude protein” | Comment accepted and wording now corrected. | In the present study, a total of 6% crude protein was lost due to the brining stress. | 355-356 |
| The limitation and strength of this research is very important to be stated in this research. | Comment accepted. We now have clearly addressed the study limitation at the end of the revised ‘conclusion’ section. | …Therefore, drying without brining may better preserve the nutritive value of tambaqui fish. However, as a recommendation to the industry sector, it is suggested that end products are further evaluated for any pathogen of economic or public health importance. | 439-441 |
| **Conclusion:** |  |  |  |
| Conclusion should be straight and clear. Please consider revising. Example, this research shows that …… | Comment accepted. By now, we have revised and rewritten the conclusion paragraph and tried to make it clearer and straight. | This research shows that drying temperature has no effect on nutrient composition of the studied fish which could be encouraging for drying of fish under – less controlled – rural tropical conditions. Brining, however, reduced some important trace elements such as Se and I, and lead to loss of muscle crude protein, with the concomitant amino acids. Brining also increased the leaching of K, I, and taurine. The formation of Maillard products was not promoted by both drying and brining treatments, as demonstrated through unaltered reactive lysine proportions. Therefore, drying without brining may better preserve the nutritive value of tambaqui fish. However, as a recommendation to the industry sector, it is suggested that the final products are further evaluated for any pathogen of economic or public health importance. | 433-441 |
| **Reviewer** #**2** | | | |
| 1. Is the manuscript technically sound, and do the data support the conclusions?  *Yes | Comment acknowledged. | - | - |
| 2. Has the statistical analysis been performed appropriately and rigorously?  *Yes | Thank you for your comments. | - | - |
| 3. Have the authors made all data underlying the findings in their manuscript fully available?  *Yes | Thank you for your comments. | - | - |
| 4. Is the manuscript presented in an intelligible fashion and written in standard English?  *Yes | Thank you for your comments. | - | - |
| **5. Review Comments to the Author:** |  |  |  |
| Line 146. Please specify the model of oven used and Humidity condition set for laboratory simulation. | Comment accepted. We now have added the oven model and laboratory humidity as well. | Each tray was placed in an electric oven (EP66, BEKSO-SA®, Belgium) and randomly set and dried with the above-mentioned temperature points. The laboratory humidity was in a range of 50-60% during the experimental period. | 190-192 |
| Line 153. The statement (Samples were finally freeze-dried) looks contradictory with the title "A laboratory simulation of the impact of brining and drying as accessible preservation strategies under tropical rural conditions on the nutritive value of tambaqui fish (*Colossoma macropomum*)". | Comment acknowledged. In fact, it is the drying oven used to simulate the tropical environment. Once all samples has undergone salting and/or drying, they were finally kept at -21^o^C until evaluated. As part of a protocol for analyzing nutrients, frozen samples were equally freeze dried, homogenously ground and used to determine nutrient concentrations.  However, seeing the importance of the comment, we now have changed the TITLE of the manuscript and elaborated this step clearly in the methodology section as well. | Impact of brining and drying processes on the nutritive value of tambaqui fish (*Colossoma macropomum*) | 1-2 |
|  |  | Samples were then kept at -21°C overnight and finally freeze-dried (CoolSafe™, SCANVAC, Denmark) for a period of 16 h each to a stable weight, uniformly ground (2.0 mm sieve), and homogenized using a grinder machine (DeˊLonghi™, KG48) at 12× speed and then individually kept in closable plastic containers at 4°C until analyzed [22]. | 197-201 |
| Line 376. The language of the conclusion must be improved for better understanding of the reader. This section must be sound. | Comment accepted. By now, we have tried to rewrite the conclusion paragraph and make it more clear and acceptably sound. We have also addressed the future focus/limitation of the study in response to reviewer 1 and editor comments. | This research shows that drying temperature has no effect on nutrient composition of the studied fish which could be encouraging for drying of fish under – less controlled – rural tropical conditions. Brining, however, reduced some important trace elements such as Se and I, and lead to loss of muscle crude protein, with the concomitant amino acids. Brining also increased the leaching of K, I, and taurine. The formation of Maillard products was not promoted by both drying and brining treatments, as demonstrated through unaltered reactive lysine proportions. Therefore, drying without brining may better preserve the nutritive value of tambaqui fish. However, as a recommendation to the industry sector, it is suggested that the final products are further evaluated for any pathogen of economic or public health importance. | 432-440 |
| **Reviewer** #**3** | | | |
| 1. Is the manuscript technically sound, and do the data support the conclusions?  *Yes | Comment acknowledged. | - | - |
| 2. Has the statistical analysis been performed appropriately and rigorously?  *Yes | Thank you for your comments. | - | - |
| 3. Have the authors made all data underlying the findings in their manuscript fully available?  *Yes | Thank you for your comments. | - | - |
| 4. Is the manuscript presented in an intelligible fashion and written in standard English?  *Yes | Thank you for your comments. | - | - |
| 5. Review Comments to the Author: |  |  |  |
| This is a good work and needs attention of the researchers. I must recommend it to be published. | Thank you for your comments. | - | - |
| Comments to editor:  In the current manuscript the authors have studied "A laboratory simulation of the impact of brining and drying as accessible preservation strategies under tropical rural conditions on the nutritive value of tambaqui fish". This is a good research work and need of the hour. I recommend this work to be published in your esteemed journal. | Thank you for the valuable feedback and comments given. | - | - |
| **Academic Editor (EA):** | | | |
| There is no simulation in this study, please change the title to Impact of brining and drying processes............... | Comment accepted. We now have changed the title. | Impact of brining and drying processes on the nutritive value of tambaqui fish (*Colossoma macropomum*) | 1-2 |
| You have mentioned that electricity is a problem to rural communities of most tropical regions, using electric oven for your experiment should not be an accessible preservative strategies | Comment accepted. We now have corrected the title and removed this sentence from the ‘Introduction’ section and the objectives as well. | Removed sentence: Freezing is an efficient manner for fish preservation, but in many rural areas of the Global South, freezing fish is not an option because of poor electricity supply and the unaffordable cost of freezers. | 38-48 |
| Please add the effect brining on the nutrient profile and at what significant level? | Comment accepted. We now have added the effect of brining together with that of drying effect. | Drying temperature had no effect on the nutrient profiles of tambaqui fish (*P > 0.05*) while brining reduced the overall protein concentration by 6% (58.8 to 55.4 g/100 g DM; *P = 0.004*). | 24-26 |
| I will suggest that you re-write your results section of the abstract: Present the ANOVA results on all the nutrient profile and the interactive effect of brining and drying processes (if it was done).  Follow by the mean separation results for the key nutrient that had significant effect.  PCA result | Comment accepted. We now have rewritten the ENTIRE abstract with the suggestions given. We apologize that we could not manage to include the interactive effect as stated. | Preservation of fish as diet ingredient is challenging in many tropical conditions due to the absence of freezing facilities, hence warranting other preservation techniques. Brine salting is one of the oldest traditional and economically viable preservation methods. The present study evaluated the effect of brine salting (15% *w/w*) prior to drying at different temperatures on the nutrient profiles of tambaqui fish (*Colossoma macropomum*). Whole fish samples (792 ± 16 g; 8 months old; n = 48) were grouped into two as brine-salted and non-salted, and treated at seven different drying temperatures of 30, 35, 40, 45, 50, 55 and 60°C for a period of 23 h each. To evaluate the impact of Maillard reaction, reactive lysine was also quantified. Drying temperature had no effect on the nutrient profiles of tambaqui fish (*P > 0.05*) while brining reduced the overall protein concentration by 6% (58.8 to 55.4 g/100 g DM; *P = 0.004*). Brining significantly diminished many amino acids taurine by 56% (7.1 to 3.1 g/kg; *P < 0.001*), methionine 17% (14.7 to 12.1 g/kg; *P < 0.001*), cysteine 11% (5.1 to 4.4 g/kg, *P = 0.016*), and reactive lysine 11% (52.0 to 46.4 g/kg; *P = 0.004*). However, alanine, arginine, and serine were not affected by brining (*P > 0.05*). Brining also reduced the concentrations of Se by 14% (149 to 128 µg/kg dry matter; *P = 0.020*), I 38% (604 to 373 µg/kg DM; *P = 0.020*), K 42% (9.71 to 5.61 g/kg DM; *P < 0.001*) and Mg 18% (1.32 to 1.10 g/kg DM; *P = < 0.001*) versus an anticipated vast increase in Na by 744% (2.70 to 22.90 g/kg DM; *P < 0.001*) and ash 28% (12.4 to 16.0 g/100g DM; *P < 0.001*) concentration. Neither brining nor drying temperature induced changes in lysine reactivity and fat content of tambaqui fish (*P > 0.05*). Agreeably, results of multivariate analysis showed a negative association between brining, Na, and ash on one side of the component and most other nutrients on the other while the lack of effect of temperature can be seen by its presence in the center. Therefore, drying without brining may better preserve the nutritive value of tambaqui fish. However, as a practical to the industry sector, it is recommended that the final products are further evaluated for any pathogen of economic or public health importance. | 16-41 |
| Please add the a sentence on the practical application of your findings. | Comment accepted. We now have addressed this at the end of the ‘abstract’ section. | However, as a practical to the industry sector, it is recommended that the final products are further evaluated for any pathogen of economic or public health importance. | 39-41 |
| Your introduction is not flowing well.  Following the general introduction on fish. please add the following:  - Explain the importance of studying the nutrient profiles in fish.  - Provide information on the natural habitat and characteristics of the studied fish  - Highlight the nutritional importance of this fish species for human consumption  -Then, Discuss the drying process and its role in enhancing the shelf life of fish  - A brief literature review on various traditional fish preservatives eg, sundrying, smoking, brinning,etc,  Then , Brining as yiu have in Line2s 42 to 60,  Drying as you have it in Lines 61 to 81. | Comment accepted. We now have rewritten the entire ‘Introduction’ section and made the flow as per the suggestion given here with. | Kindly see for the ENTIRE Introduction section on the revised manuscript with tracked changes. | 44-135 |
| This is not true, There are several studies on brining and drying of fish. What makes your study unique is species you are using. Please check the following papers as examples:  -Kituu, G. M., Shitanda, D., Silayo, V., Odote, P., & Bongyereire, J. (2008). Effect of brining on the drying rate of tilapia (Oreochromis niloticus) in a solar tunnel dryer.  -Abraha, B., Admassu, H., Mahmud, A., Tsighe, N., Shui, X. W., & Fang, Y. (2018). Effect of processing methods on nutritional and physico-chemical composition of fish: a review. MOJ Food Process Technol, 6(4), 376-82.  -Guizani, N., Rahman, M. S., Al-Ruzeiqi, M. H., Al-Sabahi, J. N., & Sureshchandran, S. (2014). Effects of brine concentration on lipid oxidation and fatty acids profile of hot smoked tuna (Thunnus albacares) stored at refrigerated temperature. Journal of Food Science and Technology, 51, 577-582.  -Sampels, S. (2015). The effects of processing technologies and preparation on the final quality of fish products. Trends in Food Science & Technology, 44(2), 131-146.  -Doe, P., & Olley, J. (2020). Drying and dried fish products. In Seafood (pp. 125-145). CRC Press | Comment acknowledged. We now have rephrased the sentence in the ‘Introduction’ section.  Also, we have referenced and cited these and other references in the introduction as well. | Despite the effect of brining and drying temperatures on the nutrient profiles of different fish species has been investigated, we hypothesized that species differences in nutrient profiles could respond differently to drying and brining and evaluated tambaqui fish. Our study also… | 124-126 |
|  |  | Kindly, see for about 15 extra references cited in the revised ‘introduction’ section. | 44-135 |
| Please you are simulating you are just checking the impact of brining and drying processes. Also, the rural conditions was not simulated using electric oven in the lab. Rural areas, they used sun drying. If you used sun drying or smoking processes, you will surely get a different result. I would have suggested you used sun drying, smoking as control | Comment accepted. We now have removed the term simulation word from this sentence in the introduction and corrected the study objectives in line with the new title. similarly from the methodology as well it has been removed.  We agree that results could be different and acknowledge your suggestion on the use of sun drying or smoking as a control, however, the experiment was solely done in Belgium where less sun exposures happen that we manage to use only electric oven. | The present study therefore evaluated the effect of brining and a range of drying temperatures on the macronutrients and micronutrients, and mineral composition as well as on the state of Maillard reaction that may reduce lysine availability. | 133-135 |
| Imported from where? | Comment accepted. We now have corrected the wording and included the place fingerlings were brought. | Fingerlings of *Colossoma macropomum* were initially brought from Aqua-Koi NV farm (http://www.aqua-koi.be/documents/home.xml?lang=nl) to TRANSfarm and reared… | 139-141 |
| Using which method? Please add more information on the rearing process. | Comment accepted. We now have added details on the rearing process. | Fingerlings of *Colossoma macropomum* were initially brought from Aqua-Koi NV farm (http://www.aqua-koi.be/documents/home.xml?lang=nl) to TRANSfarm and reared in a recirculating aquaculture system (RAS) with a weekly basis 30% water renewal (20L/day) for about 7 months period before being subject to the current study. During their stay, fish were kept in larger aqua-tanks (100 cm × 120 cm × 40 cm) at a density of 1.2 fish/m^3^ sufficient enough for movements and behavioral expression. Tanks were fixed with individual bio-filters, well aerated with a constant temperature supply, 18:6 h light - dark cycle, and supplying pipes were regularly inspected and cleaned with tap water and water quality parameters were checked twice a week and water renewed accordingly [39]. | 139-148 |
| Please add reference | Comment accepted. We now have added a reference to this. | …twice a week and water renewed accordingly [39]. | 148 |
| What type of anesthetic agent?, give the brand, manufacturer, country | Comment accepted. We now have added this. | Each group of six unconscious fish was scooped out of the tank and brought to the slaughter room in the bucket with the anesthetic agent (1mL:5L, Anest Fish^©^, Germany). | 154-156 |
| This is repetition. You have given the detail earlier | Comment accepted. We now have deleted this sentence. | with ice packs (Luxaplast, NORDIC^®^). Upon arrival, to facilitate… | 167 |
| Was it stainless? | Comment accepted. Indeed, it was, we now have added the word ‘stainless’ in this sentence. | …using a stainless post mortem saw (SC-PM/110, 254MM) and scalpel-blades… | 168-169 |
| Using tap water? An you carried out Fe and Zn analysis on the samples. How did you avoid contamination? | Comment accepted. This was a typo error and apologies for that. We indeed have used distilled water in the lab to wash the sliced fish. Now, we have corrected this. | …thoroughly washed using distilled water in pipes and then individually stored in plastic bags… | 169-170 |
| Why 15%? | Comment acknowledged. This was done based on a reference that we manage to include it at the sentence end by now. | Industrial table salt (99.9%, BDH-PROLABO®, EC) was used for preparing the 15% (*w/w*) brine solution in the present study [24]. | 172-173 |
| Please add reference | Comment accepted and reference has been added. | Industrial table salt (99.9%, BDH-PROLABO^®^, EC) was used for preparing the 15% (*w/w*) brine solution in the present study [24]. | 172-173 |
| How did select these temperatures, especially the interval of 5? Any reference? | Comment acknowledged. The intervals and limit were chosen based on several works done on fish brining/salting with a range of results obtained. We now have cited these references at the sentence end. | Uniformly sliced whole fish samples were grouped into two as brine-salted and non-salted, and treated at seven different drying temperatures of 30, 35, 40, 45, 50, 55 and 60°C for a period of 23 h each [23,24,31,37]. | 175-177 |
| Add reference for this process | Comment accepted and reference has been added. | Brine-salted fish samples were immersed in brine solution (15% *w/w*) at a ratio of 3:1 of fish to salt solution [22,27]. For each sampling unit… | 181-182 |
| Please state the humidity conditions while simulating natural drying environment and specify the model of oven used?? | Comment accepted. We now have added the oven model and laboratory humidity as well. | Each tray was placed in an electric oven (EP66, BEKSO-SA^®^, Belgium) and randomly set and dried with the above-mentioned temperature points. The simulated laboratory humidity was in a range of 50-60% during the experimental period. | 190-192 |
| This statement looks contradictory with the title "A laboratory simulation of the impact of brining and drying as accessible preservation strategies under tropical rural conditions on the nutritive value of tambaqui fish (Colossoma macropomum)" | Comment acknowledged. In fact. Once all samples has undergone salting and/or drying, they were finally kept at -21^o^C until evaluated. As part of a protocol for analyzing nutrients, frozen samples were equally freeze dried, homogenously ground and used to determine nutrient concentrations.  However, seeing the importance of the comment, we now have changed the TITLE of the manuscript and elaborated this step clearly in the methodology section as well. | Impact of brining and drying processes on the nutritive value of tambaqui fish (*Colossoma macropomum*) | 1-2 |
|  |  | Samples were then kept at -21°C overnight and finally freeze-dried (CoolSafe™, SCANVAC, Denmark) for a period of 16 h each to a stable weight, uniformly ground (2.0 mm sieve), and homogenized using a grinder machine (DeˊLonghi™, KG48) at 12× speed and then individually kept in closable plastic containers at 4°C until analyzed [22]. | 197-201 |
| Please give the particle size or the sieve size used | Comment accepted. Sieve size used now added. | …a stable weight, uniformly ground (2.0 mm sieve), and homogenized… | 199 |
| No reference for the Brining and drying processes | Comment accepted. Reference no has been added. | …speed and then individually kept in closable plastic containers at 4°C until analyzed [22]. | 200-201 |
| Please give full meaning of all the acronyms at first mentioned | Comment accepted. We have addressed this across the entire manuscript. | … The dry matter (DM) content of freeze-dried samples… | 207 |
| Please add quality control steps observed to avoid contaminations. Also, please give the Limit of detection, sensitivity, accuracy level etc of the ICP-OES used. Please give simple steps of estimating the values of the minerals using ICP-OES. | Comment accepted. We now have tried to addressed these comments as shown in the manuscript. | In brief, a gram of sample was weighed, placed into a porcelain crucible and oven-dried at 105°C for 3 h. Sample was then burnt on a hot plate and incinerated in a muffle furnace at 450°C for 16 h. Obtained ash was then moistened with a small amount of deionized water first and dissolved in 10 mL of 1:1 (*v/v*) solution of hydrochloric acid (HCl) and deionized water and evaporated to dry. Finally, the mix was re-dissolved in 10 mL of 1:9 (*v/v*) solution of HCl and deionized water, transferred into a 50 mL flask, and then diluted several folds for optimal determination of individual mineral concentration using induction-coupled plasma optical emission spectrometry (ICP-OES) method as per the manufacturer’s procedures (Iris Intrepid II XSP, Thermo Fisher Scientific, Aalst, Belgium). So as to obtain an optimum concentration range for the atomic absorption spectrometric method, the concentrations of certain minerals were determined after fivefold (Mg and Ca), 10-fold (Na and Zn), and 50-fold (K) dilutions. Likewise, the concentration of selenium was determined by ISO method (ICP-MS: ISO 11885) and iodine with tetramethylammonium hydroxide extraction and iodine quantification (ICP-MS: ISO 11885) at the Laboratory of Chemical analysis, Ghent University, Belgium. As quality control protocols, visual inspection of pump tubing at regular intervals and replacing between samples are often made to increase the precision measurements. Similarly, regular inspection and cleaning of the injector tube, spread chamber and mobilizer, and cleaning of the widows are done to avoid contamination and reduction of light intensity. Blank solutions with similar matrix were used to improve the measurement accuracy and the detection limit set was 1-100 ppb. | 219-238 |
| How many sampling point? | Comment accepted. We addressed this in the manuscript. | …from each of the seven sampling points for defatting… | 2496-247 |
| What sieve size? | Comment accepted. We now have added the sieve size. | finely ground and homogenized (2.0 mm sieve size) using a mixer mill… | 248 |
| Please present all tables in an acceptable format. Check Journal guidelines | Comment accepted. We now have corrected the Tables according to the journal’s guideline. | Kindly, see for Table 1 | 289-293 |
|  |  | Kindly, see for Table 2 | 299-304 |
|  |  | Kindly, see for Table 3 | 311-317 |
| The language of the conclusion must be improved for better understanding of the reader. This section must be sound. | Comment accepted. We now have rewritten the conclusion section and added the limitation and future recommendations at the end. | This research shows that drying temperature has no effect on nutrient composition of the studied fish which could be encouraging for drying of fish under – less controlled – rural tropical conditions. Brining, however, reduced some important trace elements such as Se and I, and lead to loss of muscle crude protein, with the concomitant amino acids. Brining also increased the leaching of K, I, and taurine. The formation of Maillard products was not promoted by both drying and brining treatments, as demonstrated through unaltered reactive lysine proportions. Therefore, drying without brining may better preserve the nutritive value of tambaqui fish. However, as a recommendation to the industry sector, it is suggested that the final products are further evaluated for any pathogen of economic or public health importance. | 432-440 |
| Please add the practical application limitation of this study, recommendations to the conclusion. |  |  |  |
| **Academic Editor:** |  |  |  |
| The manuscript has been reviewed and requires a major revision; all Editor's and reviewers' comments should be carefully considered in order to improve its quality. | Thank you for the valuable comments. We have now maximally addressed the comments and concerns given by all the reviewers and editor as well. | Kindly, see detailed answers and replies given point-by-point above under each reviewer and below for the academic editor as well. | - |
| Please include the following items when submitting your revised manuscript:   - A rebuttal letter that responds to each point raised by the academic editor and reviewer(s). You should upload this letter as a separate file labeled 'Response to Reviewers'. - A marked-up copy of your manuscript that highlights changes made to the original version. You should upload this as a separate file labeled 'Revised Manuscript with Track Changes'. - An unmarked version of your revised paper without tracked changes. You should upload this as a separate file labeled 'Manuscript'. | Comment accepted. We now have made all the required amendments and re-submitted the three items as requested. | - | - |
| If you would like to make changes to your financial disclosure, please include your updated statement in your cover letter.  Guidelines for resubmitting your figure files are available below the reviewer comments at the end of this letter. | Comment acknowledged. No changes are made regarding financial disclosure. | - | - |
| If applicable, we recommend that you deposit your laboratory protocols in protocols.io to enhance the reproducibility of your results. | Not applicable. | - | - |
| **Journal additional requirements:** |  |  |  |
| 1. Please ensure that your manuscript meets PLOS ONE's style requirements, including those for file naming. The PLOS ONE style templates can be found at: <https://journals.plos.org/plosone/s/file?id=wjVg/> PLOSOne_formatting_sample_main_body.pdf and  <https://journals.plos.org/plosone/s/file?id=ba62/> PLOSOne_formatting_sample_title_authors_affiliations.pdf | Comment acknowledged. We now have re-visited the PLOS ONE style templates and addressed all the journal’s requirements to the best of our knowledge in the revised version. | - | - |
| 2. We note that you have indicated that data from this study are available upon request. PLOS only allows data to be available upon request if there are legal or ethical restrictions on sharing data publicly. For more information on unacceptable data access restrictions, please see http://journals.plos.org/plosone/s/data-availability#loc-unacceptabledata-access-restrictions.  In your revised cover letter, please address the following prompts:  a) If there are ethical or legal restrictions on sharing a de-identified data set, please explain them in detail (e.g., data contain potentially sensitive information, data are owned by a third-party organization, etc.) and who has imposed them (e.g., an ethics committee). Please also provide contact information for a data access committee, ethics committee, or other institutional body to which data requests may be sent.  b) If there are no restrictions, please upload the minimal anonymized data set necessary to replicate your study findings as either Supporting Information files or to a stable, public repository and provide us with the relevant URLs, DOIs, or accession numbers. For a list of acceptable repositories, please see http://journals.plos.org/plosone/s/dataavailability#loc-recommended-repositories.  We will update your Data Availability statement on your behalf to reflect the information you provide. | Comment accepted. In fact, there are no legal or ethical restrictions to the data used in this study. Hence, by now, we have uploaded the dataset, output, and supplementary Table involved in the study as Supporting Information files’. | Kindly, see for uploaded supplementary files separately. | - |
| Additional Editor Comments:  The manuscript has been reviewed and requires a major revision; all Editor's and reviewers' comments should be carefully considered in order to improve its quality. Please check the uploaded files for the comments. | Comment accepted. We hope now have addressed all the comments and concerns raised by the reviewers and editor to the best of our knowledge. | Kindly, see for detailed responses and adaptations made point-by-point above under each reviewer and academic editor as well. | - |
