# Supplementary material for: Impact of brining and drying processes on the nutritive value of tambaqui fish (Colossoma macropomum)
Source: PLoS One. 2024 Apr 16;19(4):e0299926. doi: 10.1371/journal.pone.0299926 (PMC11020844; doi:10.1371/journal.pone.0299926)
Supplement: Attachment — Submitted filename: Response to Reviewers.docx [file pone.0299926.s012.docx]

**Response to Reviewers**

| **Comment** | **Response** | **Adaptation** | **Line/s** |
| --- | --- | --- | --- |
| **Reviewer** #2 | | | |
| Comments to the author: | | | |
| 1. If the authors have adequately addressed your comments raised in a previous round of review and you feel that this manuscript is now acceptable for publication, you may indicate that here to bypass the “Comments to the Author” section, enter your conflict of interest statement in the “Confidential to Editor” section, and submit your "Accept" recommendation.  *All comments have been addressed. | Thank you for your feedback in relation to our previous responses.  We still have attempted to address all the further comments given as discussed here below. | - | - |
| 2. Is the manuscript technically sound, and do the data support the conclusions?  The manuscript must describe a technically sound piece of scientific research with data that supports the conclusions. Experiments must have been conducted rigorously, with appropriate controls, replication, and sample sizes. The conclusions must be drawn appropriately based on the data presented.  *Yes | Thank you for your feedback in relation to our previous responses. | - | - |
| 3. Has the statistical analysis been performed appropriately and rigorously?  *Yes | Thank you for your feedback in relation to our previous responses. | - | - |
| 4. Have the authors made all data underlying the findings in their manuscript fully available?  The PLOS Data policy requires authors to make all data underlying the findings described in their manuscript fully available without restriction, with rare exception (please refer to the Data Availability Statement in the manuscript PDF file). The data should be provided as part of the manuscript or its supporting information, or deposited to a public repository. For example, in addition to summary statistics, the data points behind means, medians and variance measures should be available. If there are restrictions on publicly sharing data—e.g. participant privacy or use of data from a third party—those must be specified.  *Yes | Thank you for your feedback in relation to our previous responses. | - | - |
| 5. Is the manuscript presented in an intelligible fashion and written in standard English?  PLOS ONE does not copyedit accepted manuscripts, so the language in submitted articles must be clear, correct, and unambiguous. Any typographical or grammatical errors should be corrected at revision, so please note any specific errors here.  *No | Thank you for the feedback given. We now have tried to go through the entire manuscript and correct any typographical/grammatical errors including, but not limited to, the below stated points. | - | - |
| 6. Review Comments to the Author: |  |  |  |
| 1. The abstract lacks clarity and brevity and has ambiguity in presenting the problem statement and results. | Comment accepted. We now have rewritten the entire abstract and tried to make it more clear and understandable.  As indicated in the adapted abstract, we tried to address all the points raised by the reviewer one-by-one as shown below. | Preservation of fish as diet ingredient is challenging in many tropical regions due to poor socioeconomic conditions and lack of freezing facilities. So, alternative preservation techniques could be viable to address the issue. The present study evaluated the effect of brine salting (15% *w/w*) prior to drying at different temperatures on the nutrient profiles of tambaqui fish (*Colossoma macropomum*). Whole fish samples (792 ± 16 g; 8 months old; n = 48) were grouped into two as brine-salted and non-salted, and treated at seven different drying temperatures of 30, 35, 40, 45, 50, 55 and 60°C for a period of 23 h each. To evaluate the impact of Maillard reaction, reactive lysine was also quantified. Drying temperature had no effect on the evaluated macro- and micro-nutrients of tambaqui fish (*P > 0.05*) while brining reduced the overall protein concentration by 6% (58.8 to 55.4 g/100 g DM; *P = 0.004*). Brining significantly reduced many amino acids: taurine by 56% (7.1 to 3.1 g/kg; *P < 0.001*), methionine 17% (14.7 to 12.1 g/kg; *P < 0.001*), cysteine 11% (5.1 to 4.4 g/kg, *P = 0.016*), and reactive lysine 11% (52.0 to 46.4 g/kg; *P = 0.004*). However, alanine, arginine, and serine were not affected by brining (*P > 0.05*). Brining also reduced the concentrations of Se by 14% (149 to 128 µg/kg DM; *P = 0.020*), iodine 38% (604 to 373 µg/kg DM; *P = 0.020*), K 42% (9.71 to 5.61 g/kg DM; *P < 0.001*) and Mg 18% (1.32 to 1.10 g/kg DM; *P = < 0.001*) versus an anticipated vast increase in Na by 744% (2.70 to 22.90 g/kg DM; *P < 0.001*) and ash 28% (12.4 to 16.0 g/100g DM; *P < 0.001*) concentration. Neither brining nor drying temperature induced changes in % lysine reactivity and fat content of tambaqui fish (*P > 0.05*). Agreeably, results of multivariate analysis showed a negative association between brining, Na, and ash on one side of the component and most other nutrients on the other component. In conclusion, drying without brining may better preserve the nutritive value of tambaqui fish. However, as a practical remark to the industry sector, it is recommended that the final product is further evaluated for any pathogen of economic or public health importance. | 17-40 |
| Abstract:  Line 17-18: The opening sentences lack clarity and brevity and be improved to make it more catchy. The author may follow it as "Preservation of fish as diet ingredient is challenging in many tropical regions due to poor socioeconomic conditions and lack of freezing facilities. So, alternative preservation techniques could be viable to address the issue. | Comment accepted. We now have corrected the abstract’s opening sentence as per the feedback given and make it more clear. | Preservation of fish as diet ingredient is challenging in many tropical regions due to poor socioeconomic conditions and lack of freezing facilities. So, alternative preservation techniques could be viable to address the issue. | 17-19 |
| Abstract:  Line 25: ‘Profiles’? | Comment accepted. We now have removed and elaborated the word ‘profiles’. | Drying temperature had no effect on the evaluated macro- and micro-nutrients of tambaqui fish | 24-25 |
| Abstract:  Line 27: I acknowledge the efforts made by author to improve the article but some sound mistakes as mentioned here are major concerns to readers understanding. | Comment accepted. We now have corrected the wording in this sentence particularly. | Brining significantly reduced many amino acids: taurine… | 26-27 |
| Abstract:  Line 31: This type of writing (I 38%) may sounds improper. | Comment accepted. We now have extended the abbreviated iodine (I) and makes it cleared. | iodine 38%... | 31 |
| Abstract:  Line 35-38: 1e. The sentence can be split to make it more understandable. | Comment accepted. We now have shortened the sentence into one simple and understandable. | Agreeably, results of multivariate analysis showed a negative association between brining, Na, and ash on one side of the component and most other nutrients on the other component. | 35-37 |
| Abstract:  Line 35-38: ‘final products are further evaluated’ seems improper. | Comment accepted. We now have corrected the rewording. | However, as a practical remark to the industry sector, it is recommended that the final product is further evaluated… | 38-39 |
| 2. The introduction section is too long, with a lot of vague sentences, and is poor in its construction.  *Introduction section is too lengthy and lack clarity. Please make it concise and clear. | Comment accepted. We now have gone through the entire ‘Introduction’ section, removed some sentences we believe are redundant and/or vague, and attempted to make it clearer and shorter.  We, kindly, believe that we now have tried to find the balance between the different reviewers, where one asked to have a more extensive introduction, and another that asks to reduce it.  Here below are the further comments given addressed in the introduction section. | Kinly, see for tracked reduced contents in the introduction section with these line numbers. | 46-49  57-58  66-67  91-92 |
| Introduction:  Line 48-51: Must be improved to make it understandable. | Comment accepted. To minimize the ‘introduction’ length and make it with flow, we have now removed this sentence and briefly stated it below paragraph. | Nutritionally, fish meat is a prominent source of high quality essential amino acids [3,4], omega-3 fatty acids such as eicosapentaenoic acid (EPA) and docosahexaenoic acid (DHA), and trace elements such as iron, selenium, iodine that are beneficial for human health [5]. Hence, good knowledge on the nutrient… | 44-47 |
| Introduction:  Line 54: is and species seems to be contradictory. | Comment accepted. We now have removed ‘species’ and corrected the sentence. | Tambaqui fish (*Colossoma macropomum*), also known as black pacu, is the main native fish farmed in… | 50-51 |
| Introduction:  Line 56: “species???” | Comment accepted. Now, we have corrected the wording by replacing ’tambaqui’ for ‘species’. | Tambaqui alone accounted for a total production… | 52 |
| Introduction:  Line 70-71: “Preservation is a fundamental requirement of food and food ingredients in maintaining a quality food which is a vital issue in the human health and contributes to profitability” is grammatically too poor to be understandable. | Comment accepted. The sentence has now been improved. | Food preservation is a fundamental requirement in maintaining quality food, a vital issue in human health, and contributes to profitability [14,15]. | 63-64 |
| Introduction:  Line 81-84: Sentence needs attention ”Salting, on the other hand, involve different processes such as brining (immersion of fish meat into salt solution), kench curing (dry salting of fish fillets), pickling (longer storage of the fish with brine), and the possible injection of brine into the fish muscle all ending up in reducing product water content and hence extended shelf life [5,18-21].” | Comment accepted. We have made some amendment in this sentence and made it flow well with its succeeding paragraph “Brine salting…) | Salting, on the other hand, involves different processes techniques such as brining (the immersion of fish meat into salt solution), kench curing (dry salting of fish fillets), pickling (longer storage of the fish with brine), and the possible injection of brine into the fish muscle all ending up in extended product shelf life [5,18-21].  Brine salting is one of the oldest traditional and economically… | 72-77 |
| 3. The material and method are against the problem statement, i.e., the use of freeze drying. | Comment acknowledged. We understand that the concern of the reviewer that freeze-drying of the final samples may have overruled the temperature effect during oven-drying. We could have analyzed the samples without freeze-drying at the end, but to evaluate eventual differences in residual moisture content, we wanted to include freeze-drying in the final step. It is well-established that freeze-drying is a suitable method to obtain moist-free samples without noticeable effects on any of the studied nutrients. That is the reason why freeze-drying is used in many industrial food processing methods. This is elaborated on in detail in Handbook of Nutritive Value of Processed Foods (ed. Miloslav Rechcigl)  https://books.google.be/books?hl=nl&lr= &id=BRemDwAAQBAJ&oi=fnd&pg= PA45&dq=freezedrying+and+nutritive+ quality&ots=AVbkHiMmA7&sig=hzN _HgTO3t72adCedyWfy2OAjdk#v=one page&q=freezedrying%20and%20nutritive %20quality&f=false | - | - |
| Materials and methods:  Line 139-143: Too lengthy sentences may be avoided; and also ‘reared’ may need correction. | Comment accepted. We now have split the long sentence into two. Also, replaced ‘reared’ by ‘raised’. | Fingerlings of *Colossoma macropomum* were initially brought from Aqua-Koi NV farm (http://www.aqua-koi.be/documents/ home.xml?lang=nl) to TRANSfarm, Belgium. Fingerlings were then raised in a recirculating aquaculture system (RAS) with a weekly basis 30% water renewal (20L/day) for about 7 months period before being subject to the current study. | 127-131 |
| Materials and methods:  Line 158-162: May be divided into parts to make it more clear: “The study was conducted in accordance with the Declaration of Helsinki and directive 2010/63/EU of the European parliament and of the council on the protection of animals used for scientific purposes, and the wellbeing of the cultured tambaqui fish before being subjected to the experiment was assessed and approved by the institutional review board of TRANSfarm, KU Leuven, lab accreditation (KU/LA-1210616).” | Comment accepted. We now have split the long sentence into two and make it more clear. | The study was conducted in accordance with the Declaration of Helsinki and directive 2010/63/EU of the European parliament and of the council on the protection of animals used for scientific purposes. The wellbeing of the cultured tambaqui fish before being subjected to the experiment was assessed and approved by the institutional review board of TRANSfarm, KU Leuven, under laboratory accreditation of KU/LA-1210616. | 146-150 |
| Materials and methods:  Line 170: The study is interesting and have a role in managing food security but some contradictions exists like storing at -20 degrees needs a lot of energy or chemical refrigerant while this was planed as community based practice. | Comment is highly acknowledged. We agree that energy is being costing in this case. We use it as part of the study protocols and taking the time required to process the drying of the entire 96 fish samples.  Seeing this important, we have previously acknowledged this ‘energy costs and efficiency in the discussion’ section further. | Drying is an energy intensive process, which can use about 10% or more of the total energy consumed during processing. In the present study, although our aim was not to quantify the drying efficiency, we conjointly were aiming to consume less energy while drying samples. This was achieved by increasing the sample surface area of exposure, avoiding surface contact by putting beads, improved equipment insulation during and between drying, supply of a constant air ventilation at 1.0 m/s during drying, and minimizing the timing between dryings [63]. | 403-409 |
| 4. The result and discussion are well presented and have clarity. | Thank you for your feedback in relation to our previous responses. | - | - |
| 5. The conclusion is improved compared to previous one and addresses the problem statement. | Thank you for your feedback in relation to our previous responses. | - | - |
| **Reviewer #4** | | | |
| 1. If the authors have adequately addressed your comments raised in a previous round of review and you feel that this manuscript is now acceptable for publication, you may indicate that here to bypass the “Comments to the Author” section, enter your conflict of interest statement in the “Confidential to Editor” section, and submit your "Accept" recommendation.  *No response. | Thank you for your feedback in relation to our previous responses.  We now have tried to further go through the entire manuscript and address further comments given including any typographical or grammatical errors as discussed here below. | - | - |
| 2. Is the manuscript technically sound, and do the data support the conclusions?  The manuscript must describe a technically sound piece of scientific research with data that supports the conclusions. Experiments must have been conducted rigorously, with appropriate controls, replication, and sample sizes. The conclusions must be drawn appropriately based on the data presented.  *Yes | Thank you for your feedback in relation to our previous responses. | - | - |
| 3. Has the statistical analysis been performed appropriately and rigorously?  *Yes | Thank you for your feedback in relation to our previous responses. | - | - |
| 4. Have the authors made all data underlying the findings in their manuscript fully available?  The PLOS Data policy requires authors to make all data underlying the findings described in their manuscript fully available without restriction, with rare exception (please refer to the Data Availability Statement in the manuscript PDF file). The data should be provided as part of the manuscript or its supporting information, or deposited to a public repository. For example, in addition to summary statistics, the data points behind means, medians and variance measures should be available. If there are restrictions on publicly sharing data—e.g. participant privacy or use of data from a third party—those must be specified.  *Yes | Thank you for your feedback in relation to our previous responses. | - | - |
| 5. Is the manuscript presented in an intelligible fashion and written in standard English?  PLOS ONE does not copyedit accepted manuscripts, so the language in submitted articles must be clear, correct, and unambiguous. Any typographical or grammatical errors should be corrected at revision, so please note any specific errors here.  *Yes | Thank you for your feedback in relation to our previous responses. | - | - |
| 6. Review Comments to the Author: |  |  |  |
| Line 27: Please change “other while” to “other, while”. | Comment accepted. We now have corrected this sentence and make short as well in line to other reviewers comment. | …component and most other nutrients on the other component. | 37 |
| Line 142: Please change “20L/day” to 20 L/day”. Kindly, note that there is a space between a figure and its unit. | Comment accepted. We have tried to address this here and throughout the manuscript. | water renewal (20 L/day) for… | 130 |
| Line 145: Please change “well aerated” to “well-aerated”. | Comment accepted and corrected. | bio-filters, well-aerated with a constant temperature | 133-134 |
| Line 149: Please change “a 12 h” to “a 12-h”. | Comment accepted and corrected. | after a 12-h fasting… | 137 |
| Line 153: Please change “were” to “was”. | Comment accepted and corrected. | water was elevated out of the tanks… | 141 |
| Line 155: Please change “the” to “an”. | Comment accepted and corrected. | in the bucket with an anesthetic agent… | 143 |
| Line 155: Please change “1mL:5L” to “1 mL:5 L”. | Comment accepted and spaces added between figure and unit. | (1 mL:5 L, Anest Fish^©^, Germany). | 143-144 |
| Line 166: Please change “veterinary and biosciences” to “Veterinary and Biosciences”. | Comment accepted and wording now corrected. | the Department of Veterinary and Biosciences, Ghent University | 154 |
| Line 186: Please change "try" to "tray". | Comment accepted. We now have corrected the wording. | …at one fish per tray, into… | 174 |
| Lines 197-201: This highlighted sentence in these lines is too long. Please separate it into two thus "Samples were then kept at -21°C overnight and finally freeze-dried (CoolSafe™, SCANVAC, Denmark) for a period of 16 h each to a stable weight, uniformly ground (2.0 mm sieve). Subsequently, the samples were homogenized using a grinding machine (DeˊLonghi™, KG48) at 12× speed and then individually kept in closable plastic containers at 4°C until analyzed [22]." | Comment accepted. By now, we have split the long sentence into two separate sentences. | Samples were then kept at -21°C overnight and finally freeze-dried (CoolSafe™, SCANVAC, Denmark) for a period of 16 h each to a stable weight, and uniformly ground (2.0 mm sieve). Subsequently, the samples were homogenized using a grinder machine (DeˊLonghi™, KG48) at 12× speed and then individually kept in closable plastic containers at 4°C until analyzed [22]. | 185-189 |
| Line 235: Please use a past tense for "are often" and specify the number of times the visual inspection was performed. | Comment accepted. We now have addressed this. | As quality control protocols, visual inspection of pump tubing at regular intervals (every 30 min) and replacing between samples were often made to increase the precision measurements. | 221-223 |
| Line 236: Please change "spread chamber and mobilizer" to "spray chamber and nebulizer". | Comment accepted. We now have changed the wording. | Similarly, regular inspection and cleaning of the injector tube, spray chamber and nebulizer, and cleaning… | 223-224 |
| Line 237: Please change "Blanc" to "Blank". | Comment accepted. We now have changed the wording. | Blank solutions with similar matrix… | 225-226 |
| Line 242: Please change "Sulphur containing" to "Sulphur-containing". | Comment accepted. We now have changed the wording. | Sulphur-containing amino acids were measured… | 231 |
| Line 278: Please change "Proximate analysis" to "Proximate composition". | Comment accepted. We now have changed the wording. | Proximate composition | 267 |
| Line 325: Please change “temperature while” to “temperature, while”. | Comment accepted. We now have added the comma. | drying temperature, while several associations were… | 314-315 |
| Line 350: Please change "process including" to "process, including". | Comment accepted. We now have added the comma. | the drying process, including the drying type… | 339-340 |
| Line 369: Please change “dipeptides such as” to “dipeptides, such as”. | Comment accepted. We now have added the comma. | dipeptides, such as carnosine or anserine… | 358-359 |
| Line 372: Please change “conditions such as” to “conditions, such as”. | Comment accepted. We now have added the comma. | during stress conditions, such as starvation | 360-361 |
| Line 379: Please change "reduces" to "reduce". | Comment accepted. We now have changed the wording. | make cross-links that reduce their… | 368 |
| Line 386: Please change "affected while" to "affected, while". | Comment accepted. We now have added the comma. | were not affected, while glycine and proline… | 375-376 |
| Line 392: Please change "increased when" to "increased, when". | Comment accepted. We now have added the comma. | lysine and histidine increased, when salted fish was dried [59]. | 381 |
| Lines 394-396: Sequel to the highlighted statement in these lines, the authors can further explain which class of amino acids (whether hydrophobic, polar, acidic, basic, neutral) was most affected by brining. | Comment accepted. We now have addressed this. | Fat is a hydrophobic molecule and can act as a physical barrier to limit salt (NaCl) and water transport across the muscle tissue [23]. In fact, the lipid content was higher for the fish in our study (28.71%) although fat concentration was not affected by both treatments. Of the affected amino acids by brining, taurine and lysine are polar hydrophilic where are methionine and cysteine have basic hydrophobic property [47,50,51]. | 383-388 |
| Line 404: Please change "minerals such as" to "minerals, such as". | Comment accepted. We now have added the comma. | the typical skeletal minerals, such as Ca… | 393 |
| Line 414: Please change "process which" to "process, which". | Comment accepted. We now have added the comma. | Drying is an energy intensive process, which can use… | 403 |
| Lines 415-419: The sentence in lines 415-419 is too long. Please split it into two. | Comment accepted. We now have split this sentence into two sentences. | In the present study, although our aim was not to quantify the drying efficiency, we conjointly were aiming to consume less energy while drying samples. This was achieved by increasing the sample surface area of exposure, avoiding surface contact by putting beads, improved equipment insulation during and between drying, supply of a constant air ventilation at 1.0 m/s during drying, and minimizing the timing between dryings [63]. | 404-409 |
| Lines 428-430: The authors of this study, used controlled temperatures. So, how can they claim that "This implies that drying of fish under uncontrolled environmental temperature seems a suitable option in rural areas to preserve the nutritive value of (tambaqui) fish"? If Maillard reaction products are formed between the amino acid residues and reducing sugars in the fish due to a high "uncontrolled environmental temperature", can this claim (that is, the preservation of the nutritive value of the fish) still hold?  Please note that Maillard reaction products' formation can increase with increasing drying temperature. You may refer to https://doi.org/10.1016/j.fshw.2019.03.012 | Comment accepted. We absolutely agree with the comments given here. Hence, we have now removed the entire sentence from the paragraph. | … A comforting finding is that the wide range in drying temperature evaluated in our study had no noticeable impact on the nutritive value of the fish. | 416-418 |
| Lines 432-434: Sequel to the preceding point (line: 428-430) of reasoning, please be cautious in the first sentence (lines 432-435) in the conclusion. I suggest adding "within the drying temperature range used in this study". | Comment accepted. Following the removal of the above stated sentence (428-430), We now have corrected the sentence by adding the suggested phrase. | This research shows that drying temperature has no effect on nutrient composition of the studied fish which could be encouraging for drying of fish within the drying temperature range used in this study. | 420-422 |
| **Academic Editor:** |  |  |  |
| Please include the following items when submitting your revised manuscript:   - A rebuttal letter that responds to each point raised by the academic editor and reviewer(s). You should upload this letter as a separate file labeled 'Response to Reviewers'. - A marked-up copy of your manuscript that highlights changes made to the original version. You should upload this as a separate file labeled 'Revised Manuscript with Track Changes'. - An unmarked version of your revised paper without tracked changes. You should upload this as a separate file labeled 'Manuscript'. | Comment accepted. We now have addressed the second round reviews and re-submitted the three items accordingly. | Kindly, see attachments. | - |
| If you would like to make changes to your financial disclosure, please include your updated statement in your cover letter. | Comment acknowledged. No changes are made regarding financial disclosure. | - | - |
| If applicable, we recommend that you deposit your laboratory protocols in protocols.io to enhance the reproducibility of your results. | Not applicable. | - | - |
| Journal requirements: |  |  |  |
| Please review your reference list to ensure that it is complete and correct. If you have cited papers that have been retracted, please include the rationale for doing so in the manuscript text, or remove these references and replace them with relevant current references. Any changes to the reference list should be mentioned in the rebuttal letter that accompanies your revised manuscript. If you need to cite a retracted article, indicate the article’s retracted status in the References list and also include a citation and full reference for the retraction notice. | Comment acknowledged. We now have rechecked the reference and make sure its correct and complete.  No changes have been made to the reference list.  No paper was retracted among the listed references. |  |  |
| Additional editor comments: |  |  |  |
| The paper in its current state is unsuitable for publication; it requires minor revisions in accordance with the reviewers' suggestions. Please carefully attend to the reviewers' comments and ensure you do thorough grammar checks, as there are a few errors. | Comment acknowledged. We now have, to the best of our knowledge, tried to address the reviewers’ comments including grammar checks and errors as well. | Kinly, see for the detailed point-by-point reflections given above. | - |
| While revising your submission, please upload your figure files to the Preflight Analysis and Conversion Engine (PACE) digital diagnostic tool, https://pacev2.apexcovantage.com/. PACE helps ensure that figures meet PLOS requirements. To use PACE, you must first register as a user. Registration is free. Then, login and navigate to the UPLOAD tab, where you will find detailed instructions on how to use the tool. If you encounter any issues or have any questions when using PACE, please email PLOS at figures@plos.org. Please note that Supporting Information files do not need this step. | Comment accepted. We now have opened a PACE account and uploaded the figures and checked for the PLOS figures fulfilling the requirements. |  |  |
