# Supplementary material for: Impact of brining and drying processes on the nutritive value of tambaqui fish (Colossoma macropomum)
Source: PLoS One. 2024 Apr 16;19(4):e0299926. doi: 10.1371/journal.pone.0299926 (PMC11020844; doi:10.1371/journal.pone.0299926)
Supplement: Attachment — Submitted filename: Response to Reviewers.docx [file pone.0299926.s014.docx]

**Response to Reviewers**

| **Comment** | **Response** | **Adaptation** | **Line/s** |
| --- | --- | --- | --- |
| **Reviewer** #2 | | | |
| Comments to the author: | | | |
| 1. If the authors have adequately addressed your comments raised in a previous round of review and you feel that this manuscript is now acceptable for publication, you may indicate that here to bypass the “Comments to the Author” section, enter your conflict of interest statement in the “Confidential to Editor” section, and submit your "Accept" recommendation.  *All comments have been addressed | Thank you for your feedback in relation to our previous responses. | - | - |
| 2. Is the manuscript technically sound, and do the data support the conclusions?  The manuscript must describe a technically sound piece of scientific research with data that supports the conclusions. Experiments must have been conducted rigorously, with appropriate controls, replication, and sample sizes. The conclusions must be drawn appropriately based on the data presented.  *Yes | Thank you for your feedback in relation to our previous responses. | - | - |
| 3. Has the statistical analysis been performed appropriately and rigorously?  *Yes | Thank you for your feedback in relation to our previous responses. | - | - |
| 4. Have the authors made all data underlying the findings in their manuscript fully available?  The PLOS Data policy requires authors to make all data underlying the findings described in their manuscript fully available without restriction, with rare exception (please refer to the Data Availability Statement in the manuscript PDF file). The data should be provided as part of the manuscript or its supporting information, or deposited to a public repository. For example, in addition to summary statistics, the data points behind means, medians and variance measures should be available. If there are restrictions on publicly sharing data—e.g. participant privacy or use of data from a third party—those must be specified.  *Yes | Thank you for your feedback in relation to our previous responses. | - | - |
| 5. Is the manuscript presented in an intelligible fashion and written in standard English?  PLOS ONE does not copyedit accepted manuscripts, so the language in submitted articles must be clear, correct, and unambiguous. Any typographical or grammatical errors should be corrected at revision, so please note any specific errors here.  *Yes | Thank you for your feedback in relation to our previous responses. | - | - |
| **6. Review Comments to the Author:** |  |  |  |
| The author addressed all the comments, and the data presented is technically sound, have no conflict with ethical policy. | Thank you for the feedback given that has improved the manuscript’s quality.  Here below, we have tried to address the minor further comments given. | **-** | **-** |
| Line 21: To improve clarity, here mention the number of fish first then weight and age | Comment accepted. We now have corrected this in the abstract. | Whole fish samples (n = 48; 792 ± 16 g; 8 months old) were… | 21 |
| Line 39: change “is” to “may”. | Comment accepted. We now have corrected this in the abstract. | the final product may further evaluated… | 39 |
| Line 145: change “ice cubes” to “Ice buckets”. | Comment accepted. We now have corrected this in the revised manuscript. | …transported in Ice buckets for the experiment… | 145 |
| Line 161: Preparation of *w/w* solution is controversial. In all the above statements water was used in liters. | Comment accepted. With apologies, this was a typo. We agree, it was salt (g) that we dilute in water (L) to make our solution. We now have corrected this here and in the entire manuscript as well with those related to our study. | … for preparing the 15% (*w/v*) brine solution in the present study [24]. | 160-161 |
|  |  | The present study evaluated the effect of brine salting (15% *w/v*) prior to drying… | 19-20 |
|  |  | Brine-salted fish samples were immersed in brine solution (15% *w/v*) at a ratio of… | 169 |
|  |  | The present study evaluated the effect of brine salting (15% *w/v*) prior to drying… | 331 |
|  |  | In the present study, however, an acceptable (15% *w/v*) salt was used… | 347-348 |
| Line 200: “method number” for AOAC? | Comment accepted. We now have added the method number for AOAC. | …described by AOAC Method 938.08 [43]. | 200 |
| Line 204: Its controversial to the above statement given in line no. 200. | Comment accepted. We now have corrected this reference with its method number. We also have properly addressed the Iso method reference below it. | The total ash content was determined following the AOAC Method 938.08 [43] and mineral (calcium, sodium, potassium, copper, iron, magnesium, manganese, phosphorus and zinc) concentrations of fish were evaluated after dry ashing mineralization according to ISO (ICP-AES: ISO 11885) method [44] as per the procedures described by Gorsuch [45]. | 205-208 |
| Line 213: Its not a technical term so please mention quantitatively. | Comment accepted. Indeed, the ‘several folds’ briefed here were quantitatively explained in the next sentence. We now have corrected this. | Finally, the mix was re-dissolved in 10 mL of 1:9 (*v/v*) solution of HCl and deionized water, transferred into a 50 mL flask, and diluted to volume with deionized water. The measurement of individual mineral concentration was done using induction-coupled plasma optical emission spectrometry (ICP-OES) method as per the manufacturer’s procedures (Iris Intrepid II XSP, Thermo Fisher Scientific, Aalst, Belgium). So as to obtain an optimum concentration range for the atomic absorption spectrometric method, the concentrations of certain minerals were determined after fivefold (Mg and Ca), 10-fold (Na and Zn), and 50-fold (K) dilutions. | 213-220 |
| Line 430: change “are” to “may”. | Comment accepted. We now have changed the wording. | final products may further evaluated for any pathogen of economic or public health importance. | 431 |
| **Reviewer #4** | | | |
| The authors have satisfactorily revised the paper in line with the recommendation made earlier, improving it quality. | Thank you for your feedback given in relation to our previous responses. This has improved the quality of our manuscript. | - | - |
| 1. If the authors have adequately addressed your comments raised in a previous round of review and you feel that this manuscript is now acceptable for publication, you may indicate that here to bypass the “Comments to the Author” section, enter your conflict of interest statement in the “Confidential to Editor” section, and submit your "Accept" recommendation.  *Yes | Thank you for your feedback in relation to our previous responses. | - | - |
| 2. Is the manuscript technically sound, and do the data support the conclusions?  The manuscript must describe a technically sound piece of scientific research with data that supports the conclusions. Experiments must have been conducted rigorously, with appropriate controls, replication, and sample sizes. The conclusions must be drawn appropriately based on the data presented.  *Yes | Thank you for your feedback in relation to our previous responses. | - | - |
| 3. Has the statistical analysis been performed appropriately and rigorously?  *Yes | Thank you for your feedback in relation to our previous responses. | - | - |
| 4. Have the authors made all data underlying the findings in their manuscript fully available?  The PLOS Data policy requires authors to make all data underlying the findings described in their manuscript fully available without restriction, with rare exception (please refer to the Data Availability Statement in the manuscript PDF file). The data should be provided as part of the manuscript or its supporting information, or deposited to a public repository. For example, in addition to summary statistics, the data points behind means, medians and variance measures should be available. If there are restrictions on publicly sharing data—e.g. participant privacy or use of data from a third party—those must be specified.  *Yes | Thank you for your feedback in relation to our previous responses. | - | - |
| 5. Is the manuscript presented in an intelligible fashion and written in standard English?  PLOS ONE does not copyedit accepted manuscripts, so the language in submitted articles must be clear, correct, and unambiguous. Any typographical or grammatical errors should be corrected at revision, so please note any specific errors here.  *Yes | Thank you for your feedback in relation to our previous responses. | - | - |
| **Academic Editor:** |  |  |  |
| Please include the following items when submitting your revised manuscript:   - A rebuttal letter that responds to each point raised by the academic editor and reviewer(s). You should upload this letter as a separate file labeled 'Response to Reviewers'. - A marked-up copy of your manuscript that highlights changes made to the original version. You should upload this as a separate file labeled 'Revised Manuscript with Track Changes'. - An unmarked version of your revised paper without tracked changes. You should upload this as a separate file labeled 'Manuscript'. | Comment accepted. We now have addressed the comments raised by Revier-2 and re-submitted the three items accordingly. | Kindly, see attachments. | - |
| If you would like to make changes to your financial disclosure, please include your updated statement in your cover letter. | No changes are made with regard to financial disclosure. | - | - |
| If applicable, we recommend that you deposit your laboratory protocols in protocols.io to enhance the reproducibility of your results. | Not applicable. | - | - |
| **Journal requirements:** |  |  |  |
| Please review your reference list to ensure that it is complete and correct. If you have cited papers that have been retracted, please include the rationale for doing so in the manuscript text, or remove these references and replace them with relevant current references. Any changes to the reference list should be mentioned in the rebuttal letter that accompanies your revised manuscript. If you need to cite a retracted article, indicate the article’s retracted status in the References list and also include a citation and full reference for the retraction notice. | Comment acknowledged. We now have rechecked the reference and make sure its correct and complete.  No changes have been made to the reference list.  No paper was retracted among the listed references. | - | - |
| Additional editor comments: |  |  |  |
| Thank you for the good work, and the paper is looking good now. However, as Reviewer 2 pointed out, there are a few minor corrections that are necessary. Please attend to these corrections as quickly as possible. | Comment accepted. We now have, to the best of our knowledge, tried to address Reviewer-2 minor corrections as stated. | Kinly, see for the detailed point-by-point reflections given above. | - |
| While revising your submission, please upload your figure files to the Preflight Analysis and Conversion Engine (PACE) digital diagnostic tool, https://pacev2.apexcovantage.com/. PACE helps ensure that figures meet PLOS requirements. To use PACE, you must first register as a user. Registration is free. Then, login and navigate to the UPLOAD tab, where you will find detailed instructions on how to use the tool. If you encounter any issues or have any questions when using PACE, please email PLOS at figures@plos.org. Please note that Supporting Information files do not need this step. | Comment accepted. We now have opened a PACE account and uploaded the figures and checked for the PLOS figures fulfilling the requirements. |  |  |
